# SVM-Prot 2016: A Web-Server for Machine Learning Prediction of Protein Functional Families from Sequence Irrespective of Similarity

**DOI:** 10.1371/journal.pone.0155290

**Published:** 2016-08-15

**Authors:** Ying Hong Li, Jing Yu Xu, Lin Tao, Xiao Feng Li, Shuang Li, Xian Zeng, Shang Ying Chen, Peng Zhang, Chu Qin, Cheng Zhang, Zhe Chen, Feng Zhu, Yu Zong Chen

**Affiliations:** 1 Innovative Drug Research and Bioinformatics Group, Innovative Drug Research Centre and School of Pharmaceutical Sciences, Chongqing University, Chongqing, 401331, China; 2 Bioinformatics and Drug Discovery group, Department of Pharmacy, National University of Singapore, Singapore, 117543, Singapore; 3 Zhejiang Key Laboratory of Gastro-intestinal Pathophysiology, Zhejiang Hospital of Traditional Chinese Medicine, Zhejiang Chinese Medical University, Hangzhou, P. R. China; 4 School of Mathematics and Statistics, Beijing Institute of Technology, Beijing, China; Harbin Institute of Technology Shenzhen Graduate School, CHINA

## Abstract

Knowledge of protein function is important for biological, medical and therapeutic studies, but many proteins are still unknown in function. There is a need for more improved functional prediction methods. Our SVM-Prot web-server employed a machine learning method for predicting protein functional families from protein sequences irrespective of similarity, which complemented those similarity-based and other methods in predicting diverse classes of proteins including the distantly-related proteins and homologous proteins of different functions. Since its publication in 2003, we made major improvements to SVM-Prot with (1) expanded coverage from 54 to 192 functional families, (2) more diverse protein descriptors protein representation, (3) improved predictive performances due to the use of more enriched training datasets and more variety of protein descriptors, (4) newly integrated BLAST analysis option for assessing proteins in the SVM-Prot predicted functional families that were similar in sequence to a query protein, and (5) newly added batch submission option for supporting the classification of multiple proteins. Moreover, 2 more machine learning approaches, K nearest neighbor and probabilistic neural networks, were added for facilitating collective assessment of protein functions by multiple methods. SVM-Prot can be accessed at *http://bidd2.nus.edu.sg/cgi-bin/svmprot/svmprot.cgi*.

## Introduction

The knowledge of protein function is essential for studying biological processes [1], understanding disease mechanisms [2], and exploring novel therapeutic targets [3,4]. Apart from experimental methods, a number of *in-silico* approaches have been developed and extensively used for protein function prediction. These methods include sequence similarity [5], sequence clustering [6], evolutionary analysis [7], gene fusion [8], protein interaction [9], protein remote homology detection [10,11], protein functional family classification based on sequence-derived [12,13] or domain [1] features, and the integrated approaches that combine multiple methods, algorithms and/or data sources for enhanced functional predictions [5,14–16]. A protein functional family is a group of proteins with specific type of molecular functions (e.g. proteases [17]), binding activities (e.g. RNA-binding [18]), or involved in specific biological processes defined by the Gene Ontology [19] (e.g. DNA repair [20]). Moreover, models of protein function prediction have been constructed for more broadly-defined functional families such as transmembrane [21], virulent [22] and secretory [23] proteins, and a large-scale community-based critical assessment of protein function annotation (CAFA) revealed that the improvements of current protein function prediction tools were in urgent need [24]. Despite the development and extensive exploration of these methods, there is still a huge gap between proteins with and without functional characterizations. Continuous efforts are therefore needed for developing new methods and improving existing methods. These efforts have been made possible by the rapidly expanding knowledge of protein sequence [25], structural [26], functional [19] and other [27–30] data.

The uncharacterized proteins comprise a substantial percentage of the predicted proteins in many genomes, and some of these proteins are of no clear sequence or structural similarity to a protein of known function [31,32]. A particular challenge is to predict the function of these proteins from their sequence without the knowledge of similarity, clustering or interaction relationship with a known protein. As part of the collective efforts in developing such prediction methods, we have developed a web-based software SVM-Prot that employs a machine learning method, support vector machines (SVM), for predicting protein functional families from protein sequences irrespective of sequence or structural similarity [12], which have shown good predictive performances [33–40] to complement other methods or as part of the integrated approaches in predicting the function of diverse classes of proteins including the distantly-related proteins and homologous proteins of different functions.

The previous version of SVM-Prot covered 54 functional families. Its predictive accuracies of these families were ranging from 53.03% to 99.26% in sensitivity and from 82.06% to 99.92% in specificity [12]. Since the early 2000s, the number of proteins with sequence information had dramatically expanded from 2 million to more than 48.7 million entries in the UniProt database, and the number of annotated functional families with more than 100 sequence entries had significantly increased from 54 to 192 [25]. Our analysis on all “reviewed” protein entries in the UniProt database revealed that the overwhelming majority (80.23%) of these entries were from those 192 families. The enriched protein sequence data could be employed to expand the coverage and improve the predictive performance of SVM-Prot. Moreover, our earlier study suggested that the prediction performance of SVM could be substantially enhanced by the use of a more diverse set of protein descriptors for representing more comprehensive classes of proteins [41]. Thus, SVM-Prot was upgraded by using the enriched protein data and more diverse protein descriptors to train models for all 192 functional families and to improve the predictive performance of SVM-Prot. The prediction models for an additional set of Gene Ontology [19] functional families will be developed and added into SVM-Prot in the near future.

To facilitate the analysis of specific proteins of the SVM-Prot predicted functional families that might be relevant to a query protein, a new option conducting BLAST sequence alignment was provided to [42] search proteins of the SVM-Prot predicted functional families that were similar to the query protein. Moreover, a batch submission option for loading multiple protein sequences was also included. Given that the functional prediction capacity could be enhanced by the integration of multiple methods [5,14,15], two machine learning prediction tools, K nearest neighbor (kNN) and probabilistic neural networks (PNN), were integrated into this version of SVM-Prot to facilitate the collective assessment of protein functional families. These two tools had been explored for functional prediction of proteins [43–46] and other biomolecules [47]. Since these two tools had been extensively used for developing over 39 protein functional family prediction models (**S1 Table**), and because of their potential utility in complementing SVM from the nearest neighbor and neural network perspectives, SVM-Port could serve the community by providing the alternative protein functional family prediction tools based on these and other machine learning methods.

## Results and Discussion

To evaluate the predictive performance of models in the SVM-Prot, the sensitivity (*SE*), precision (*PR*), and specificity (*SP*) of the independent evaluation datasets were calculated and demonstrated in **Table 1**and **S2 Table**. *SE*, *PR* and *SP* of the SVM model were in the range of 50.00~99.99%, 5.31~99.99% and 82.06~99.99%, respectively. In the kNN model, the performances were 51.06~99.99% for *SE*, 17.86~94.49% for *PR* and 90.19~99.99% for *SP*. Moreover, *SE*, *PR* and *SP* of the PNN model were in the range of 60.49~99.99%, 25.00~99.75% and 97.34~99.99%, respectively. The *SEs* and *PRs* of the SVM classifier were generally lower and with larger variations than the *SPs*. This was partly due to the imbalanced training sets with the numbers of non-members greatly surpassing those of the members. Imbalanced training sets were known to adversely affect the machine learning prediction performance, particularly the minority class [48,49]. Moreover, not all functional families were sufficiently covered by the known proteins, particularly those with < 100 known protein members, the inadequate coverage of the respective training sets likely affect *SE*s to varying degrees.

**Table 1:** Partial list of the protein functional families covered by SVM-Prot and the prediction performance of the SVM, kNN and PNN models on the independent testing sets. The complete list is provided in **S2 Table**. The predicted results are given in Sensitivity SE = TP/(TP+FN), Specificity SP = TN/(TN+FP), Precision PR = TP/(TP + FP), where TP = true positive, FN = false negative, TN = true negative, and FP = false positive respectively.

Family Name	GO Id	Training Dataset		Testing Dataset		Independent Dataset		SVM			KNN			PNN		
		Positive	Negative	Positive	Negative	Positive	Negative	SE (%)	SP (%)	PR (%)	SE (%)	SP (%)	PR (%)	SE (%)	SP (%)	PR (%)
Actin capping	GO:0051693	652	41797	128	39584	102	36797	95.1	99.99	93.3	73.3	99.9	55.0	91.2	99.9	71.0
Calmodulin-binding	GO:0005516	465	41405	223	39198	164	36421	87.2	99.99	90.5	70.0	99.4	41.6	82.9	99.9	84.0
DNA recombination	GO:0006310	1678	10614	3382	18224	2391	13763	85.7	97.4	92.1	67.5	99.3	80.3	77.6	98.9	77.0
DNA repair	GO:0006281	2142	10643	1179	17646	1438	13544	88.7	96.8	85.9	67.6	96.8	68.0	64.3	99.3	90.4
DNA-directed DNA polymerase	GO:0003887	825	9588	963	19524	869	13900	81.9	98.7	88.5	51.1	99.4	41.2	80.2	99.7	66.4
EC1.5 Oxidoreductases (CH-NH donors)	GO:0016645	276	8755	59	15283	70	12006	58.6	99.6	66.1	84.5	95.8	76.0	64.2	99.2	92.6
EC2.9 Transferases (selenium-containing)	GO:0016785	693	41834	620	39620	617	36835	96.0	99.99	99.3	83.7	99.7	81.4	92.4	99.9	92.5
EC3.7 Acting on carbon-carbon bonds	GO:0016822	1429	41786	760	39543	738	36786	96.5	99.9	94.6	84.4	99.4	78.0	91.2	99.9	95.2
EC4.4 Carbon-sulfur lyases	GO:0016846	182	8999	76	15086	58	12031	60.3	99.9	83.3	77.0	99.0	82.7	83.8	99.2	86.8
EC5.1 Racemases and Epimerases	GO:0016854	379	8796	95	15268	66	12020	53.0	99.4	53.9	80.7	93.8	80.0	69.3	98.7	94.5
EC6.6 Forming nitrogen-metal bonds	GO:0051002	1590	41758	348	39529	336	36762	89.3	99.9	91.7	88.4	98.7	55.7	79.5	99.99	94.0
Elongation factor activity	GO:0003746	1069	41788	938	39570	914	36788	97.5	99.99	98.8	95.8	99.6	83.7	84.1	99.9	94.0
G protein coupled receptors	GO:0004930	927	8320	4998	20216	2532	14244	95.6	98.1	94.5	96.6	98.9	64.1	94.1	99.9	93.4
Growth factor activity	GO:0008083	423	41680	301	39458	243	36696	88.9	99.9	88.5	76.7	99.9	81.9	86.0	99.9	86.7
GTPase activation	GO:0005096	429	41584	207	39359	113	36597	92.9	99.9	83.3	61.8	99.6	42.2	86.7	99.9	78.4
Heparin-binding	GO:0008201	182	41591	123	39344	92	36600	89.1	99.9	73.9	70.7	99.9	75.0	90.2	99.9	61.0
Lipid degradation	GO:0016042	403	8775	233	20635	237	14701	78.9	99.9	97.4	64.8	99.8	72.0	75.1	99.9	89.6
Lipid-binding	GO:0008289	274	8530	166	20926	167	14724	84.4	99.9	93.4	72.8	99.6	71.2	66.9	99.7	72.1
rRNA-binding protein	GO:0019843	708	7972	1245	16044	101	11997	94.1	98.7	59.0	96.5	98.3	91.4	95.8	98.7	93.6
Sigma factor activity	GO:0016987	101	41835	60	39616	54	36835	87.0	99.99	85.5	68.3	99.9	50.6	83.3	99.99	81.8

To further evaluate the capability of SVM-Prot in predicting the functional families of novel proteins, a comprehensive literature search for recently reported novel proteins was conducted using the keyword “novel” in combination with “protein”, “enzyme”, “transporter”, “DNA binding”, “RNA-binding”, “viral”, or “bacterial”. As a result, 42 novel proteins published in 2015 or 2014 that had been explicitly described as novel in the literature were identified. These proteins were not in the SVM-Prot training datasets but with available sequence in the literature or public databases.

**S3 Table** summarized the prediction results of those 42 novel proteins by SVM-Prot, FFPred 3 [50] and NCBI BLAST [51], and the detailed prediction results were further provided in **S4 Table**. The function of a novel protein was considered as matched to a computer identified functional family when these two exactly matched at a specifically defined class level. Take the formate-nitrite transporter as an example, it belongs to the formate transporter family, the major intrinsic protein superfamily and the transporter TC1.A class. This families or classes are considered as specifically defined class levels, but the transporter family is too broadly defined. Overall, the number of functional families predicted or outputted by SVM-Prot for each novel protein was in the range of 3~18, and that by FFPred was in the range of 16~55 (if predictions of low reliability were included, the number should change to 45~101). Moreover, the function of 13 out of those 42 novel proteins was correctly assigned to one functional family predicted by SVM-Prot, and 7 (if prediction results of low reliability were included, the number should change to 12) were correctly matched by FFPred (**S3 Table**). In particular, amongst those 13 proteins predicted by SVM-Prot, 7 were ranked as top-1 in the list of predicted functional families, 2 were ranked as top-2, and 4 were ranked as top-5. However, for FFPred, only one protein was ranked as top-1, another one was ranked as top-2, and 2 more proteins were ranked as top-10. The majority (8 proteins) of the predicted proteins by FFPred were ranked within the range of top-27 to top-70. Thus, SVM-Prot is capable of predicting the functional families of novel proteins at comparable yield and reduced false hit rates with respect to FFPred. It should be strongly cautioned that these two servers for protein function prediction were upgraded at different times with varying coverage of training datasets, so the difference in the prediction results may not reflect the true prediction capability of these servers.

As a further comparison, the performance of BLAST on those 42 novel proteins was also evaluated. The number of similarity proteins with E-value < 0.05 for each novel protein was in the range of 0~112, and the function of 30 out of those 42 novel proteins were correctly matched to one of the BLAST identified similarity proteins (20, 2, 6, 2 are ranked as top-1, top-2, top-4 and top-10, respectively) (**S3 Table**). However, caution needs to be raised about the straightforward comparison of the BLAST results with those of the SVM-Prot and FFPred. BLAST searched proteins may cover the previously or recently deposited similarity proteins that are of the same or similar functions with respect to our tested novel proteins, while some of these similarity proteins may not be in the training set of both SVM-Prot and FFPred. Nonetheless, the better prediction performance of BLAST on these novel proteins suggests a need for more frequent upgrade of the SVM-Prot and FFPred by enriched up-to-date training datasets.

One useful strategy for overcoming the imbalanced datasets problem is to re-construct the training sets into more balanced ones by either over sampling the minority class [48] or under sampling the majority one [49], which might compromise the training datasets by introducing noises to the minority class or reducing the diversity of the majority one. In SVM-Prot, the training sets of the non-members were constructed from the minimal set of representative proteins from the Pfam domain families. Our study showed that further reduction of the training sets by one protein per Pfam family significantly reduced the *SPs* without much improvement of the *SEs*. Therefore, no further reduction of the training data was made. Another effective strategy for reducing the negative influence of imbalanced data is to separately optimize the pair of cost parameters of SVM models at the same time [52], particularly the cost for the errors on the positive samples compared to negative ones. In the development of SVM models, due to the very high diversity of each training dataset (containing 7613~46,223 proteins), both the separate and uniform cost parameter optimization scheme led to very high cost parameters for both positive and negative samples that achieve similar levels of prediction performance.

The capability of protein function prediction can be affected by multiple factors, including insufficient diversity of proteins in some functional families, inadequate coverage or representation of certain important structural and/or physicochemical features by the current datasets and protein descriptors, deficiency of the computational algorithms and parameter optimization procedures. The capability of the machine learning functional prediction tools has been enhanced by the expanded protein data, improvement of computational algorithms and exploration of integrated prediction strategies using multiple methods [53]. In addition to the employment of the continuously expanding protein data, SVM-Prot may be improved by exploring the newly developed computational methods. In particular, there have been new progresses in the development and the use of a new machine learning method, deep learning, for predicting protein secondary structure and other local structural properties [54–56], which may be potentially extended for protein function prediction. SVM-Prot can also be improved by integrating multiple methods and algorithms for enhanced functional predictions [5,14,15].

As an effective ensemble classifier, LibD3C [57] was widely cited by the recent publications aiming at identifying the DNA-binding proteins [58,59], predicting the cytokine-receptor interactions [60] and discovering immunoglobulins [61]. **S5 Table** summarized the prediction performances of the SVM, LibD3C, kNN and PNN on the independent testing sets of 10 randomly selected representative families covered by the SVM-Prot. These 10 protein families included 4 enzyme families (EC1.5, EC2.9, EC4.4 and EC5.1), actin capping family, DNA recombination family, DNA repair family, elongation factor activity family, GPCR family and lipid-binding protein family. *PR*, *SE* and *SP* of the SVM model were in the range of 53.9~99.3%, 53.00~97.5% and 96.8~99.99%, respectively. In the LibD3C models the corresponding performances were 52.39~90.51% for *PR*, 79.23~99.03% for *SE* and 96.86~99.89% for *SP*. The kNN method resulted in the performances of 55.0~83.7% for *PR*, 67.5~96.6% for *SE* and 93.8~99.9% for *SP*. Moreover, *PR*, *SE* and *SP* of the PNN model were in the range of 71.0~94.5%, 64.2~94.1% and 98.7~99.9%, respectively. As demonstrated in **S5 Table**, prediction performances (*PR*, *SE* and *SP*) were comparable among SVM, LibD3C, kNN and PNN, indicating that each method was an effective complement to other methods. It should be strongly cautioned that those 10 randomly tested families may not be enough in representing the prediction performances of all protein families covered by the current SVM-Prot. Therefore, a comprehensive analysis on all SVM-Prot families using above classifiers is needed for the next update of the SVM-Prot.

## Methods

Instead of direct alignment or clustering of sequences, the SVM-Prot classification models classifies a protein into functional families based on the analysis of sequence-derived structural and physicochemical properties [33,34]. Proteins known to be in a functional family (e.g., proteases) and those outside the family (e.g., representatives of all non-protease proteins) are used to train a classification model, which recognizes specific sequence-derived features for classifying proteins either into or outside the functional family. Proteins of specific functional family share common structural and physicochemical features [62,63], which may be recognized by a machine learning classification model given the availability of sufficiently diverse training datasets [64].

### Data collection

**Table 1**and **S2 Table** provided a partial and complete list of the protein functional families covered by the upgraded SVM-Prot and the predictive performances of the SVM, kNN and PNN models. These families included G-protein coupled receptor family from GPCRDB [63], nuclear receptor family from NucleaRDB [63], 50 enzyme families from BRENDA [62], 20 transporter families from TCDB [65], 1 channel family from LGICdb [66], 24 molecular binding families (e.g. DNA-binding, RNA-binding, iron-binding), 67 Gene Ontology (30 molecular function and 37 biological process) families, and 28 broadly defined functional families from the UniProt database [25]. The 19 broadly defined functional families were selected on the following basis: either the prediction models for these families have been developed (e.g. allergen proteins [47]), or the relevant functions have some common features exploitable for developing prediction models (e.g.. cAMP binding). As illustrated above, the reason why protein functional families were derived from multiple sources was partly because of their complementary coverage and different functional perspectives. For instance, 122 functional families predictable in SVM-Prot were not covered by FFPred [50], while 391 functional families provided in FFPred were not covered by SVM-Prot. Thus, SVM-Prot may serve to complement other prediction servers by providing different coverage of protein functional families.

### Datasets construction

To prepare datasets for constructing the model of each functional family, the training, testing and independent datasets were carefully prepared by following a strict procedure. Firstly, protein names of members in each family were collected from the UniProt [25], and protein members of the same name but different species origin were grouped together. Secondly, protein members in each group were iteratively selected and put into the training, testing, and independent datasets as positive samples. Thirdly, to generate negative samples, protein members in each functional family were mapped into the pfam [67] protein families. The pfam families with at least one member of the functional family were named as “positive family”, while the rest of the pfam families were named as “negative family”. Fourthly, 3 representative proteins from each “negative family” were randomly selected and iteratively put into the training, testing, and independent datasets as negative samples.

During the model construction, the parameter optimization for each training set was tested by testing set. When the optimized parameter was found, the training and testing sets were combined together to form a new training set, and the optimized parameter was further applied to train a new model. Then, independent dataset was used to evaluate the performance of the newly constructed model and to detect the overfitting problem. Once the optimized parameter passed the evaluation, it was used to train a final model by integrating training, testing, and independent datasets. All duplicated proteins in each training, testing, independent evaluation dataset or among them were removed before the model construction.

### Protein representation

Extensive efforts were applied to the exploration of web-based or stand-alone tools for extracting the features from protein sequences [68,69]. For example, the Pse-in-One is a server for generating various modes of pseudo components of DNA, RNA, and protein sequences [68]. In this work, each sequence is represented by various physicochemical properties including 9 properties of the early version SVM-Prot (amino acid composition, polarity, hydrophobicity, surface tension, charge, normalized Van der Waals volume, polarizability, secondary structure and solvent accessibility) and 4 additional properties in this version SVM-Prot (molecular weight, solubility, number of hydrogen bond donor in side chain, and number of hydrogen bond acceptor in side chain) [69]. All properties are encoded in 3 descriptors, named as composition (C), transition (T), and distribution (D) [70]. C is the fraction of amino acids with a particular property. T characterizes the percent frequency of amino acids of a particular property neighbored by amino acids of another specific property. D measures the fractional chain length within which the first, 25%, 50%, 75% and 100% of the amino acids of a particular property is located.

Take a hypothetical protein (AEAAAEAEEAAAAAEAEEEAAEEAEEEAAE) with 16 alanines (*n*_1_ = 16) and 14 glutamic acids (*n*_2_ = 14) as an example. The composition (C) for these two amino acids are *n*_1_/(*n*_1_ + *n*_2_) = 0.53 and *n*_2_/(*n*_1_ + *n*_2_) = 0.47, respectively. Moreover, this protein contains 15 A-to-E and E-to-A transitions (T) with percent frequency of 15/29 = 0.52. Furthermore, the first, 25%, 50%, 75% and 100% of amino acid A are located within the first, 5, 12, 20, and 29 residues, respectively. Therefore, the distribution (D) for amino acid A can be calculated as (1/30 = 0.03, 5/30 = 0.17, 12/30 = 0.40, 20/30 = 0.67, 29/30 = 0.97, and that for amino acid E can also be calculated in the same way. Overall, the amino acid descriptors for this sequence are C = (0.53, 0.47), T = (0.51), and D = (0.03, 0.17, 0.40, 0.67, 0.97, 0.07, 0.27, 0.60, 0.77, 1.00), respectively. In most studies, amino acids are divided into three classes for each property. The combined descriptors for each property consist of 21 elements (3 for C, 3 for T and 15 for D). Moreover, the Moreau-Broto autocorrelation [71] of amino acid index and Pseudo-amino acid composition [72] are added for presenting correlation of the structural and physicochemical properties within each protein sequence.

### Protein functional family prediction models

Three types of classification models were developed for predicting protein functional families in SVM-Prot. The first model is SVM, which is based on the structural risk minimization (SRM) principle from statistical learning theory [64]. In linearly separable cases, SVM constructs a hyperplane to separate two different classes of feature vectors with a maximum margin. A feature vector *x*_*i*_ is composed of protein descriptors which were described in the previous section. The hyperplane is constructed by finding another vector *w* and a parameter *b* that minimizes ‖*w*‖^2^ and satisfies the following conditions:

$$w*x_{i}+b\leq +1\;for\;y_{i}=+1\;(in\;the\;functional\;family)$$ (1)

$$w*x_{i}+b\leq -1\;for\;y_{i}=-1\;(outside\;the\;functional\;family)$$ (2)

where *y*_*i*_ is the class index, *w* is a vector normal to the hyperplane, |*b*|/‖*w*‖ is the perpendicular distance from the hyperplane to the origin and ‖*w*‖^2^ is the Euclidean norm of *w*. After the determination of *w* and *b*, a feature vector *x* can be classified by:

sign[(w*x)+b] (3)

In non-linearly separable cases, SVM maps the input variable into a high dimensional feature space using a kernel function *K*(*x*_*i*_,*x*_*j*_). In SVM-Prot, Libsvm-3.20 [73] was used for developing the SVM models using the Gaussian kernel:

$$K(x_{i},x_{j})=e^{-{\left\|x_{j}-x_{i}\right\|}^{2}/2\sigma^{2}}$$ (4)

The second model is kNN [74], which computes the Euclidean distance $D=\sqrt{{\left||x-x_{i}|\right|}^{2}}$ between the query vector *x* of a query protein and the vector *x*_*i*_ of every protein in the training set, then selects k vectors nearest to the query vector *x*, and predicts the class of the query vector *x* based on the class of the majority of the k nearest neighbors.

The third model is PNN, which is a form of neural network that uses Bayes optimal decision rule *h*_*i*_*c*_*i*_*f*_*i*_(*x*) > *h*_*j*_*c*_*j*_*f*_*j*_(*x*) for classification [75], where *h*_*i*_ and *h*_*j*_ are the prior probabilities, *c*_*i*_ and *c*_*j*_ are the costs of misclassification and *f*_*i*_(*x*) and *f*_*j*_(*x*) are the probability density function for class *i* and *j* respectively. A query vector *x* is classified into class *i* if the product of all the three terms is greater for class *i* than for any other class *j* (*j* ≠ *i*). The probability density function for each class can be estimated by using the Parzen’s nonparametric estimator:

$$g(x)=\frac{1}{n}\sum_{i=1}^{n}exp(-\sum_{j=1}^{p}{\left(\frac{x_{j}-x_{ij}}{\sigma_{j}}\right)}^{2})$$ (5)

where *n* is the number of proteins in a class, *p* is the number of features, *x*_*j*_ is the *j*^*th*^ feature of a query protein, *x*_*ij*_ is the *j*^*th*^ feature of the *i*^*th*^ protein in the class, and *σ*_*j*_ is the smoothing factor of this feature. PNN uses a single adjustable parameter, a smoothing factor *σ* for the radial basis function in the Parzen’s nonparameteric estimator, to speed-up the training process orders of magnitude faster than the traditional neural networks.

**Fig 1:**
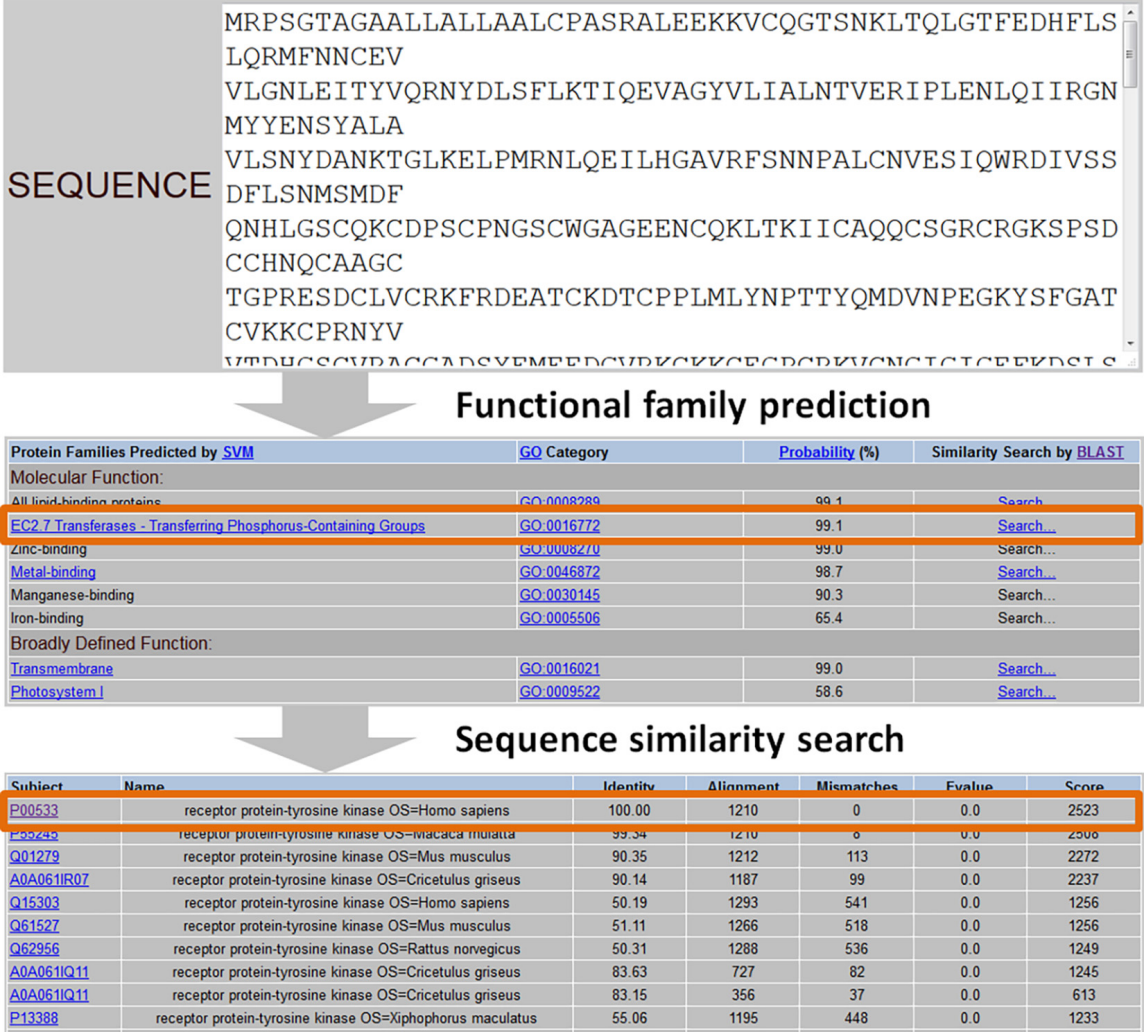
An example of SVM-Prot prediction of an EGFR protein sequence and the its subsequent BLAST sequence alignment analysis of the similarity proteins of the SVM-Prot predicted functional family.

After the prediction of the functional families of a query protein, an option is provided for the user to align their query protein sequence with the sequences of the seed proteins in the SVM-Prot predicted functional families by using the BLAST sequence alignment program obtained from NCBI [42]. The top-ranked proteins (up to 20 sequences) of each SVM-Prot predicted family that are with the highest sequence similarity (the lowest E-values) to the query protein are provided in a separate output page. As the knowledge of protein functional family may not be specific enough to analyze the function of a query protein, this option facilitates the convenient and quick assessment of potential specific functions of a query protein. **Fig 1**illustrates an example of SVM-Prot prediction of an EGFR protein sequence, which predicted EC2.7 Transferases transferring phosphorus-containing group family as the top family for this protein, a click of the BLAST search further indicated that this protein is a receptor protein-tyrosine kinase.

### Performance measurement

The performance of SVM, kNN and PNN models were assessed by three different measurements. The first one is using sensitivity (*SE*), specificity (*SP*, also known as recall) and precision (*PR*) to evaluate the predictive performance of the independent validation datasets, which are defined as below:

SE=TP/(TP+FN) (6)

SP=TN/(TN+FP) (7)

PR=TP/(TP+FN) (8)

where *TP*, *TN*, *FP* and *FN* are the number of true positives, true negatives, false positives and false negatives, respectively. In the real world, the number of proteins outside a specific functional family should significantly surpass that within the family. Thus, a slight decline of specificity (*SP*) would induce tremendous false positive prediction results, which reminds us to primarily focus on the *SP* when evaluating the model’s prediction performance.

The second measurement is the use of platt’s posterior class probability [50,76] for scoring the predicted functional families of a query protein. This probability has been used for scoring the machine learning classification of protein functional families [50], fold classes [77], transmembrane topology [78], secondary structures [79], and the effect of missense mutations on protein function [80]. It has also been built into such popular machine learning software as LibSVM [73], in which the posterior probability takes the form of a sigmoid function:

$$Pr(y=1|f)\approx P_{AB}(f)\equiv \frac{1}{1+exp(Af+B)}$$ (9)

where *f* = *f*(*x*) is the output of the SVM and the parameters *A* and *B* are optimized via cross validation of the training sets.

The last measurement is the test of these models by a set of newly published novel proteins (reported in 2014 and 2015) with their functions reported in the respective publications, and a comparative analysis between SVM-Prot and two popular protein function prediction tools were provided.

## Supporting Information

S1 Table List of literature-reported protein functional family prediction models developed by using kNN and PNN methods. (DOCX)

S2 Table Complete list of the protein functional families covered by SVMProt and the prediction performance of the SVM, kNN and PNN models on the independent testing sets. (DOCX)

S3 Table List of the novel proteins published in 2015 and 2014 that are not in the SVMProt training sets and have available sequence in the literature or public databases. (DOCX)

S4 Table The detailed results of the prediction of the functional families of the 42 novel proteins by SVMProt, FFPred and NCBI BLAST. (DOCX)

S5 Table 10 representative protein functional families covered by SVM-Prot and the prediction performance of the LibD3C, SVM, kNN and PNN models on the independent testing sets. (DOCX)
