# Supplementary material for: SVM-Prot 2016: A Web-Server for Machine Learning Prediction of Protein Functional Families from Sequence Irrespective of Similarity
Source: PLoS One. 2016 Aug 15;11(8):e0155290. doi: 10.1371/journal.pone.0155290 (PMC4985167; doi:10.1371/journal.pone.0155290)
Supplement: S1 Table — (DOCX) [file pone.0155290.s001.docx]

**Table S1.** List of literature-reported protein functional family prediction models developed by using kNN and PNN methods.

| **Prediction Problem** | **Method** | **Ref** |
| --- | --- | --- |
| Gene Ontology protein functonal class | downward random walk kNN | [[1](#_ENREF_1)] |
| Gene Ontology protein functonal class | Multi-Source kNN | [[2](#_ENREF_2)] |
| Enzyme family | kNN | [[3](#_ENREF_3)] |
| Enzyme class | Mulan Binary Relevance kNN | [[4](#_ENREF_4)] |
| Enzyme subfamily class | Adaptive fuzzy kNN | [[5](#_ENREF_5)] |
| Enzyme/non-Enzyme classification | kNN | [[6](#_ENREF_6)] |
| Acidic and alkaline enzyme discrimination | PNN | [[7](#_ENREF_7)] |
| GPCRs and their types | kNN | [[8](#_ENREF_8)] |
| GPCRs and their types | PNN | [[8](#_ENREF_8)] |
| GPCR | kNN | [[9](#_ENREF_9)] |
| GPCR | PNN | [[9](#_ENREF_9)] |
| Heat Shock Protein families and J-protein types | PNN | [[10](#_ENREF_10)] |
| Gene function based on three Escherichia coli classification schemes suggested by biologists | Regression-based kNN | [[11](#_ENREF_11)] |
| Allergens | kNN | [[12](#_ENREF_12)] |
| Food protein allergenicity | kNN | [[13](#_ENREF_13)] |
| Membrane proteins | Optimized Evidence-Theoretic kNN | [[14](#_ENREF_14)] |
| Membrane proteins | Multilabel KNN | [[15](#_ENREF_15)] |
| Membrane protein types | kNN | [[16](#_ENREF_16)] |
| Outer membrane proteins | kNN | [[17](#_ENREF_17)] |
| Outer membrane proteins | Fuzzy kNN | [[18](#_ENREF_18)] |
| Transmembrane helices | PNN | [[19](#_ENREF_19)] |
| Transmembrane beta-barrel proteins | Modified kNN | [[20](#_ENREF_20)] |
| Transmembrane beta-barrel proteins | kNN | [[21](#_ENREF_21)] |
| Lysosomal membrane protein types | PNN | [[22](#_ENREF_22)] |
| Gene ontology protein subcellular location | kNN | [[23](#_ENREF_23)] |
| Gene ontology protein subcellular location | kNN | [[24](#_ENREF_24)] |
| Gene ontology protein subcellular multi-localization | KNN-SVM ensemble classifier | [[25](#_ENREF_25)] |
| Protein subcellular location | kNN | [[26](#_ENREF_26)] |
| Protein subcellular location | PNN | [[26](#_ENREF_26)] |
| Protein subcellular location | kNN | [[27](#_ENREF_27)] |
| Protein subcellular location | PNN | [[27](#_ENREF_27)] |
| Protein subcellular location | kNN | [[28](#_ENREF_28)] |
| Protein subcellular location | PNN | [[28](#_ENREF_28)] |
| Protein subcellular location | PNN | [[29](#_ENREF_29)] |
| Protein subcellular location | PNN | [[30](#_ENREF_30)] |
| Protein subcellular location | kNN | [[31](#_ENREF_31)] |
| Protein subcellular location | Fuzzy kNN | [[32](#_ENREF_32)] |
| Protein subcellular location | Fuzzy kNN | [[33](#_ENREF_33)] |
| Protein subcellular location | Optimally weighted fuzzy kNN | [[34](#_ENREF_34)] |

**References**

1. Yu G, Zhu H, Domeniconi C, Liu J (2015) Predicting protein function via downward random walks on a gene ontology. BMC Bioinformatics 16: 271.

2. Lan L, Djuric N, Guo Y, Vucetic S (2013) MS-kNN: protein function prediction by integrating multiple data sources. BMC Bioinformatics 14 Suppl 3: S8.

3. Nasibov E, Kandemir-Cavas C (2009) Efficiency analysis of KNN and minimum distance-based classifiers in enzyme family prediction. Comput Biol Chem 33: 461-464.

4. De Ferrari L, Aitken S, van Hemert J, Goryanin I (2012) EnzML: multi-label prediction of enzyme classes using InterPro signatures. BMC Bioinformatics 13: 61.

5. Huang WL, Chen HM, Hwang SF, Ho SY (2007) Accurate prediction of enzyme subfamily class using an adaptive fuzzy k-nearest neighbor method. Biosystems 90: 405-413.

6. Davidson NJ, Wang X (2010) Non-Alignment Features Based Enzyme/Non-Enzyme Classification Using an Ensemble Method. Proc Int Conf Mach Learn Appl: 546-551.

7. Khan ZU, Hayat M, Khan MA (2015) Discrimination of acidic and alkaline enzyme using Chou's pseudo amino acid composition in conjunction with probabilistic neural network model. J Theor Biol 365: 197-203.

8. Zia Ur R, Khan A (2012) Identifying GPCRs and their types with Chou's pseudo amino acid composition: an approach from multi-scale energy representation and position specific scoring matrix. Protein Pept Lett 19: 890-903.

9. Ur-Rehman Z, Khan A (2011) G-protein-coupled receptor prediction using pseudo-amino-acid composition and multiscale energy representation of different physiochemical properties. Anal Biochem 412: 173-182.

10. Ahmad S, Kabir M, Hayat M (2015) Identification of Heat Shock Protein families and J-protein types by incorporating Dipeptide Composition into Chou's general PseAAC. Comput Methods Programs Biomed.

11. Yao Z, Ruzzo WL (2006) A regression-based K nearest neighbor algorithm for gene function prediction from heterogeneous data. BMC Bioinformatics 7 Suppl 1: S11.

12. Dimitrov I, Flower DR, Doytchinova I (2013) AllerTOP--a server for in silico prediction of allergens. BMC Bioinformatics 14 Suppl 6: S4.

13. Zorzet A, Gustafsson M, Hammerling U (2002) Prediction of food protein allergenicity: a bioinformatic learning systems approach. In Silico Biol 2: 525-534.

14. Chou KC, Shen HB (2007) MemType-2L: a web server for predicting membrane proteins and their types by incorporating evolution information through Pse-PSSM. Biochem Biophys Res Commun 360: 339-345.

15. Huang C, Yuan JQ (2013) A multilabel model based on Chou's pseudo-amino acid composition for identifying membrane proteins with both single and multiple functional types. J Membr Biol 246: 327-334.

16. Wang T, Xia T, Hu XM (2010) Geometry preserving projections algorithm for predicting membrane protein types. J Theor Biol 262: 208-213.

17. Yan C, Hu J, Wang Y (2008) Discrimination of outer membrane proteins using a K-nearest neighbor method. Amino Acids 35: 65-73.

18. Hayat M, Khan A (2012) Discriminating outer membrane proteins with Fuzzy K-nearest Neighbor algorithms based on the general form of Chou's PseAAC. Protein Pept Lett 19: 411-421.

19. Yu DJ, Shen HB, Yang JY (2012) SOMPNN: an efficient non-parametric model for predicting transmembrane helices. Amino Acids 42: 2195-2205.

20. Garrow AG, Agnew A, Westhead DR (2005) TMB-Hunt: a web server to screen sequence sets for transmembrane beta-barrel proteins. Nucleic Acids Res 33: W188-192.

21. Hu J, Yan C (2008) A method for discovering transmembrane beta-barrel proteins in Gram-negative bacterial proteomes. Comput Biol Chem 32: 298-301.

22. Tripathi V, Gupta DK (2014) Discriminating lysosomal membrane protein types using dynamic neural network. J Biomol Struct Dyn 32: 1575-1582.

23. Shen HB, Yang J, Chou KC (2007) Euk-PLoc: an ensemble classifier for large-scale eukaryotic protein subcellular location prediction. Amino Acids 33: 57-67.

24. Li L, Zhang Y, Zou L, Li C, Yu B, et al. (2012) An ensemble classifier for eukaryotic protein subcellular location prediction using gene ontology categories and amino acid hydrophobicity. PLoS One 7: e31057.

25. Li LQ, Zhang Y, Zou LY, Zhou Y, Zheng XQ (2012) Prediction of protein subcellular multi-localization based on the general form of Chou's pseudo amino acid composition. Protein Pept Lett 19: 375-387.

26. Garg P, Sharma V, Chaudhari P, Roy N (2009) SubCellProt: predicting protein subcellular localization using machine learning approaches. In Silico Biol 9: 35-44.

27. Khan A, Majid A, Hayat M (2011) CE-PLoc: an ensemble classifier for predicting protein subcellular locations by fusing different modes of pseudo amino acid composition. Comput Biol Chem 35: 218-229.

28. Hayat M, Khan A, Yeasin M (2012) Prediction of membrane proteins using split amino acid and ensemble classification. Amino Acids 42: 2447-2460.

29. Guo J, Pu X, Lin Y, Leung H (2006) Protein subcellular localization based on PSI-BLAST and machine learning. J Bioinform Comput Biol 4: 1181-1195.

30. Guo J, Lin Y, Liu X (2006) GNBSL: a new integrative system to predict the subcellular location for Gram-negative bacteria proteins. Proteomics 6: 5099-5105.

31. Wang T, Yang J (2010) Predicting subcellular localization of gram-negative bacterial proteins by linear dimensionality reduction method. Protein Pept Lett 17: 32-37.

32. Huang Y, Li Y (2004) Prediction of protein subcellular locations using fuzzy k-NN method. Bioinformatics 20: 21-28.

33. Tung TQ, Lee D (2009) A method to improve protein subcellular localization prediction by integrating various biological data sources. BMC Bioinformatics 10 Suppl 1: S43.

34. Nasibov E, Kandemir-Cavas C (2008) Protein subcellular location prediction using optimally weighted fuzzy k-NN algorithm. Comput Biol Chem 32: 448-451.
