# Supplementary material for: SVM-Prot 2016: A Web-Server for Machine Learning Prediction of Protein Functional Families from Sequence Irrespective of Similarity
Source: PLoS One. 2016 Aug 15;11(8):e0155290. doi: 10.1371/journal.pone.0155290 (PMC4985167; doi:10.1371/journal.pone.0155290)
Supplement: S2 Table — (DOCX) [file pone.0155290.s002.docx]

**Table S2.** Complete list of the protein functional families covered by SVMProt and the prediction performance of the SVM, kNN and PNN models on the independent testing sets. The prediction results are given in Sensitivity SE=TP/(TP+FN), Specificity SP=TN/(TN+FP), Precision PR=TP/(TP + FP), where TP=true positive, FN=false negative, TN=true negative, and FP=false positive respectively.

| **Family Name** | **GO Id** | **Training Dataset** | | **Testing Dataset** | | **Independent Dataset** | | **SVM** | | | **KNN** | | | **PNN** | | |
| --- | --- | --- | --- | --- | --- | --- | --- | --- | --- | --- | --- | --- | --- | --- | --- | --- |
|  |  | **Pos** | **Neg** | **Pos** | **Neg** | **Pos** | **Neg** | **SE (%)** | **SP (%)** | **PR (%)** | **SE (%)** | **SP (%)** | **PR (%)** | **SE (%)** | **SP (%)** | **PR (%)** |
| EC1.1 Oxidoreductases acting on the CH-OH group of donors | GO:0016614 | 1164 | 9324 | 1805 | 14608 | 599 | 11952 | 82.5 | 96.1 | 72.0 | - | - | - | - | - | - |
| EC1.10 Oxidoreductases acting on diphenols and related substances as donors | GO:0016679 | 219 | 8348 | 88 | 15732 | 85 | 12026 | 76.5 | 99.4 | 68.4 | 88.5 | 99.5 | 90.9 | 85.0 | 99.5 | 90.5 |
| EC1.11 Oxidoreductases acting on a peroxide as acceptor | GO:0016684 | 344 | 8416 | 345 | 15669 | 168 | 12031 | 86.9 | 99.6 | 86.9 | 82.1 | 97.7 | 80.4 | 82.3 | 98.8 | 88.9 |
| EC1.13 Oxidoreductases acting on single donors with incorporation of molecular oxygen (oxygenases) | GO:0016701 | 152 | 8232 | 97 | 15836 | 52 | 12022 | 55.8 | 99.7 | 69.1 | 79.4 | 99.0 | 82.8 | - | - | - |
| EC1.14 Oxidoreductases acting on paired donors with incorporation reduction of molecular oxygen | GO:0016705 | 566 | 8896 | 786 | 15128 | 131 | 11998 | 71.0 | 98.9 | 62.0 | 76.7 | 95.5 | 69.2 | 73.5 | 99.2 | 92.5 |
| EC1.15 Oxidoreductases acting on superoxide as acceptor | GO:0016721 | 259 | 7881 | 418 | 16221 | 240 | 12039 | 92.5 | 99.6 | 91.7 | 92.6 | 99.6 | 94.5 | 94.0 | 99.8 | 97.1 |
| EC1.17 Oxidoreductases acting on CH2 groups | GO:0016725 | 100 | 8308 | 113 | 15787 | 55 | 12035 | 78.2 | 99.8 | 82.7 | - | - | - | 82.3 | 99.2 | 95.3 |
| EC1.18 Oxidoreductases acting on iron-sulfur proteins as donors | GO:0016730 | 244 | 8229 | 233 | 15850 | 85 | 12027 | 91.8 | 99.6 | 78.0 | 83.7 | 99.2 | 83.5 | 87.7 | 99.6 | 92.4 |
| EC1.2 Oxidoreductases acting on the aldehyde or oxo group of donors | GO:0016903 | 665 | 8920 | 719 | 15076 | 328 | 11985 | 79.0 | 98.5 | 77.1 | - | - | - | - | - | - |
| EC1.3 Oxidoreductases acting on the CH-CH group of donors | GO:0016627 | 491 | 8917 | 134 | 15107 | 110 | 11998 | 66.4 | 98.9 | 56.2 | 86.8 | 91.1 | 75.5 | 74.9 | 99.1 | 96.4 |
| EC1.4 Oxidoreductases acting on the CH-NH2 group of donors | GO:0016638 | 307 | 8869 | 94 | 15187 | 76 | 12016 | 65.8 | 99.5 | 65.8 | 92.5 | 93.8 | 70.2 | 77.3 | 99.2 | 94.2 |
| EC1.5 Oxidoreductases acting on the CH-NH group of donors | GO:0016645 | 276 | 8755 | 59 | 15283 | 70 | 12006 | 58.6 | 99.6 | 66.1 | 84.5 | 95.8 | 76.0 | 64.2 | 99.2 | 92.6 |
| EC1.6 Oxidoreductases acting on NADH or NADPH | GO:0016651 | 1333 | 9132 | 2210 | 14876 | 1183 | 11989 | 94.5 | 98.2 | 92.7 | - | - | - | - | - | - |
| EC1.7 Oxidoreductases acting on other nitrogenous compounds as donors | GO:0016661 | 170 | 8356 | 86 | 15705 | 44 | 12018 | 65.9 | 99.7 | 69.1 | - | - | - | - | - | - |
| EC1.8 Oxidoreductases acting on a sulfur group of donors | GO:0016667 | 299 | 8531 | 116 | 15513 | 68 | 12009 | 58.8 | 99.6 | 66.7 | 90.4 | 95.1 | 82.0 | - | - | - |
| EC1.9 Oxidoreductases acting on a heme group of donors | GO:0016675 | 561 | 7807 | 9515 | 16270 | 4841 | 12026 | 99.3 | 99.0 | 99.0 | - | - | - | - | - | - |
| EC2.1 Transferases transferring one-carbon groups | GO:0016741 | 1509 | 9991 | 800 | 13905 | 279 | 11934 | 68.1 | 85.0 | 20.4 | - | - | - | - | - | - |
| EC2.2 Transferases transferring aldehyde or ketone residues | GO:0016744 | 35 | 8197 | 32 | 8121 | 31 | 8008 | 83.9 | 99.7 | 89.7 | - | - | - | - | - | - |
| EC2.3 Acyltransferases | GO:0016764 | 302 | 8001 | 246 | 8288 | 240 | 7993 | 81.7 | 97.3 | 87.9 | - | - | - | - | - | - |
| EC2.4 Glycosyltransferases | GO:0042123 | 945 | 8896 | 1236 | 14981 | 288 | 11926 | 70.5 | 94.2 | 41.5 | - | - | - | - | - | - |
| EC2.5 Transferases transferring alkyl or aryl groups, other than methyl groups | GO:0016765 | 764 | 9174 | 543 | 14865 | 195 | 12008 | 70.3 | 98.1 | 59.6 | - | - | - | - | - | - |
| EC2.6 Transferases transferring nitrogenous groups | GO:0016769 | 343 | 8684 | 306 | 15401 | 107 | 12031 | 70.1 | 99.0 | 60.5 | - | - | - | - | - | - |
| EC2.7 Transferases transferring phosphorus-containing groups | GO:0016772 | 3892 | 12324 | 3765 | 13146 | 3016 | 12707 | 81.7 | 89.1 | 79.8 | - | - | - | - | - | - |
| EC2.8 Transferases transferring sulfur-containing groups | GO:0016782 | 203 | 8549 | 43 | 15538 | 30 | 12032 | 66.7 | 99.8 | 64.5 | 92.5 | 94.3 | 79.5 | 89.3 | 98.8 | 94.9 |
| EC2.9 Transferring selenium-containing groups | GO:0016785 | 693 | 41834 | 620 | 39620 | 617 | 36835 | 96.0 | 99.99 | 99.3 | 83.7 | 99.7 | 81.4 | 92.4 | 99.9 | 92.5 |
| EC3.1 Hydrolases acting on ester bonds | GO:0016788 | 2482 | 10859 | 1557 | 12777 | 533 | 11807 | 71.1 | 90.6 | 45.6 | - | - | - | - | - | - |
| EC3.2 Glycosylases | GO:0016798 | 337 | 7867 | 381 | 8410 | 317 | 7990 | 84.5 | 94.9 | 84.0 | - | - | - | - | - | - |
| EC3.3 Hydrolases acting on ether bonds | GO:0016801 | 97 | 8999 | 66 | 15102 | 54 | 12039 | 59.3 | 99.4 | 50.0 | 84.0 | 99.4 | 84.3 | 85.2 | 99.9 | 97.2 |
| EC3.4 Hydrolases acting on peptide bonds (Peptidases) | GO:0008233 | 2011 | 10402 | 1557 | 13236 | 354 | 11807 | 74.6 | 94.2 | 48.6 | - | - | - | - | - | - |
| EC3.5 Hydrolases acting on carbon-nitrogen bonds, other than peptide bonds | GO:0016810 | 1020 | 9498 | 442 | 14450 | 215 | 11959 | 60.5 | 97.8 | 54.2 | - | - | - | - | - | - |
| EC3.6 Hydrolases acting on acid anhydrides | GO:0016817 | 2195 | 9504 | 1450 | 14439 | 750 | 11962 | 91.6 | 95.6 | 75.7 | - | - | - | - | - | - |
| EC3.7 Acting on carbon-carbon bonds | GO:0016822 | 1429 | 41786 | 760 | 39543 | 738 | 36786 | 96.5 | 99.9 | 94.6 | 84.4 | 99.4 | 78.0 | 91.2 | 99.9 | 95.2 |
| EC3.8 Acting on halide bonds | GO:0016824 | 959 | 41792 | 215 | 39536 | 199 | 36792 | 89.5 | 99.9 | 82.8 | 51.1 | 99.8 | 77.5 | 74.4 | 99.9 | 87.6 |
| EC4.1 Carbon-carbon lyases | GO:0016830 | 546 | 8145 | 781 | 8130 | 609 | 7986 | 89.8 | 89.4 | 83.9 | - | - | - | - | - | - |
| EC4.2 Carbon-oxygen lyases | GO:0016835 | 505 | 8231 | 383 | 8049 | 403 | 7992 | 80.4 | 92.2 | 80.8 | - | - | - | - | - | - |
| EC4.3 Carbon-nitrogen lyases | GO:0016840 | 218 | 8068 | 196 | 16017 | 39 | 12031 | 74.4 | 99.3 | 43.9 | 83.0 | 98.2 | 74.1 | 85.1 | 98.8 | 82.6 |
| EC4.4 Carbon-sulfur lyases | GO:0016846 | 182 | 8999 | 76 | 15086 | 58 | 12031 | 60.3 | 99.9 | 83.3 | 77.0 | 99.0 | 82.7 | 83.8 | 99.2 | 86.8 |
| EC4.6 Phosphorus-oxygen lyases | GO:0016849 | 200 | 8789 | 77 | 15257 | 82 | 12011 | 67.1 | 82.1 | 5.8 | 72.7 | 98.0 | 66.8 | 60.5 | 98.8 | 73.9 |
| EC5.1 Racemases and Epimerases | GO:0016854 | 379 | 8796 | 95 | 15268 | 66 | 12020 | 53.0 | 99.4 | 53.9 | 80.7 | 93.8 | 80.0 | 69.3 | 98.7 | 94.5 |
| EC5.2 Cis-trans-Isomerases | GO:0016859 | 35 | 8404 | 115 | 15682 | 108 | 12032 | 66.7 | 99.5 | 75.0 | 89.9 | 97.2 | 87.0 | 88.4 | 98.7 | 93.5 |
| EC5.3 Intramolecular oxidoreductases | GO:0016860 | 461 | 8122 | 95 | 8062 | 178 | 12009 | 75.8 | 98.0 | 57.7 | - | - | - | - | - | - |
| EC5.4 Intramolecular transferases | GO:0016866 | 329 | 8714 | 147 | 15353 | 77 | 12022 | 54.6 | 99.4 | 57.5 | - | - | - | - | - | - |
| EC5.5 Intramolecular lyases | GO:0016872 | 47 | 7909 | 24 | 16196 | 107 | 12031 | 70.1 | 99.0 | 60.5 | 62.4 | 99.8 | 76.7 | 75.4 | 99.9 | 82.6 |
| EC5.99 Other Isomerases | GO:0016853 | 163 | 8038 | 401 | 16045 | 166 | 12029 | 92.2 | 99.6 | 87.4 | 96.6 | 94.8 | 80.5 | 94.5 | 99.6 | 98.3 |
| EC6.1 Ligases forming carbon-oxygen bonds | GO:0016875 | 281 | 8115 | 382 | 8198 | 315 | 8007 | 90.8 | 97.3 | 91.4 | - | - | - | - | - | - |
| EC6.2 Ligases forming carbon-sulfur bonds | GO:0016877 | 149 | 8233 | 158 | 15862 | 64 | 12216 | 79.7 | 99.8 | 79.7 | 92.0 | 95.7 | 75.6 | 89.5 | 98.9 | 92.3 |
| EC6.3 Ligases forming carbon-nitrogen bonds | GO:0016879 | 381 | 8133 | 360 | 8151 | 351 | 7991 | 83.8 | 95.5 | 86.7 | - | - | - | - | - | - |
| EC6.4 Ligases forming carbon-carbon bonds | GO:0016885 | 99 | 8543 | 45 | 15556 | 44 | 12037 | 63.6 | 99.9 | 87.5 | 94.8 | 96.4 | 72.6 | 86.7 | 98.9 | 89.4 |
| EC6.5 Ligases forming phosphoric ester bonds | GO:0016886 | 94 | 8679 | 38 | 15411 | 31 | 12033 | 71.0 | 99.9 | 78.6 | 92.0 | 96.3 | 66.3 | 88.5 | 99.8 | 96.9 |
| EC6.6 Forming nitrogen-metal bonds | GO:0051002 | 1590 | 41758 | 348 | 39529 | 336 | 36762 | 89.3 | 99.9 | 91.7 | 88.4 | 98.7 | 55.7 | 79.5 | 100 | 94.0 |
| TC1.A alpha-type channels | GO:0005216 | 381 | 8786 | 280 | 17432 | 189 | 13081 | 86.8 | 99.3 | 78.9 | - | - | - | - | - | - |
| TC1.B beta-barrel porins | GO:0015288 | 221 | 9008 | 58 | 19454 | 92 | 14207 | 70.7 | 99.6 | 69.2 | - | - | - | - | - | - |
| TC1.C Pore-forming toxins (proteins and peptides) | GO:0005216 | 357 | 9007 | 47 | 19371 | 127 | 13467 | 78.7 | 99.8 | 87.0 | - | - | - | - | - | - |
| TC1.E Holins | GO:0034290 | 100 | 7513 | 60 | 18851 | 70 | 13155 | 78.6 | 99.9 | 93.2 | - | - | - | - | - | - |
| TC2.A porters (symporters, uniporters, antiporters) | GO:0015291 | 629 | 8175 | 785 | 17951 | 424 | 13035 | 87.3 | 98.5 | 80.4 | - | - | - | - | - | - |
| TC2.A.1 Major facilitator family (MFS) | GO:0005215 | 248 | 14260 | 54 | 14236 | 81 | 14194 | 76.5 | 97.9 | 28.7 | - | - | - | - | - | - |
| TC2.A.3 Amino acid-polyamine-organocation (APC) | GO:0005215 | 91 | 14284 | 32 | 14264 | 33 | 14217 | 93.9 | 99.4 | 40.3 | - | - | - | - | - | - |
| TC2.A.6 Resistance-nodulation-cell division (RND) family | GO:0005215 | 65 | 14296 | 22 | 14281 | 32 | 14234 | 71.9 | 99.4 | 34.9 | - | - | - | - | - | - |
| TC2.C Ion-gradient-driven energizers | GO:0015291 | 166 | 8014 | 88 | 18335 | 119 | 13153 | 76.5 | 99.8 | 87.5 | - | - | - | - | - | - |
| TC3.A P-P-bond-hydrolysis-driven transporters | GO:0015405 | 1220 | 9549 | 1321 | 16583 | 1140 | 13038 | 78.7 | 97.6 | 86.3 | - | - | - | - | - | - |
| TC3.A.1 ATP-binding cassette (ABC) family | GO:0043190 | 641 | 9008 | 150 | 19554 | 373 | 14257 | 94.1 | 91.6 | 36.4 | - | - | - | - | - | - |
| TC3.A.15 The Outer Membrane Protein Secreting Main Terminal Branch (MTB) family | GO:0015405 | 84 | 8997 | 9 | 19605 | 33 | 14277 | 60.6 | 98.1 | 12.4 | - | - | - | - | - | - |
| TC3.A.3 P-type ATPase (P-ATPase) family | GO:0015662 | 123 | 8999 | 44 | 19585 | 68 | 14268 | 97.1 | 95.7 | 17.4 | - | - | - | - | - | - |
| TC3.A.5 Type II (general) secretory pathway (IISP) family | GO:0015405 | 254 | 8997 | 29 | 19587 | 128 | 14268 | 85.9 | 91.5 | 15.2 | - | - | - | - | - | - |
| TC3.D Oxidoreduction-driven transporters | GO:0015453 | 435 | 8529 | 982 | 19985 | 677 | 14233 | 91.1 | 99.5 | 94.5 | - | - | - | - | - | - |
| TC3.E Light absorption-driven transporters | GO:0015454 | 139 | 7954 | 701 | 20650 | 411 | 14278 | 96.1 | 99.9 | 97.3 | - | - | - | - | - | - |
| TC4.A Phosphotransfer-driven group translocators | GO:0008982 | 197 | 7887 | 220 | 18434 | 185 | 13141 | 82.7 | 99.7 | 87.9 | - | - | - | - | - | - |
| TC8.A Auxiliary transport proteins | GO:0006810 | 223 | 8388 | 174 | 17938 | 167 | 13135 | 74.3 | 99.8 | 89.2 | - | - | - | - | - | - |
| TC9.A Recognized transporters of unknown biochemical mechanism | GO:0005215 | 203 | 8034 | 189 | 18276 | 165 | 13128 | 78.8 | 99.3 | 75.1 | - | - | - | - | - | - |
| TC9.B Putative uncharacterized transport proteins | GO:0005215 | 869 | 9079 | 586 | 17158 | 585 | 13100 | 80.2 | 98.4 | 82.7 | - | - | - | - | - | - |
| 7 transmembrane receptor (metabotropic glutamate family) | GO:0004930 | 116 | 9001 | 40 | 19613 | 69 | 14283 | 89.9 | 95.8 | 16.8 | - | - | - | - | - | - |
| 7 transmembrane receptor (odorant receptor) | GO:0004930 | 130 | 8999 | 11 | 19631 | 39 | 14291 | 97.4 | 97.6 | 17.6 | - | - | - | - | - | - |
| 7 transmembrane receptor (rhodopsin family and chemoreceptor) | GO:0004930 | 729 | 8061 | 4611 | 20538 | 2294 | 14275 | 96.9 | 99.2 | 97.3 | - | - | - | - | - | - |
| 7 transmembrane receptor (secretin family) | GO:0004930 | 218 | 9007 | 71 | 19581 | 129 | 14270 | 90.7 | 94.9 | 24.0 | - | - | - | - | - | - |
| Actin binding | GO:0003779 | 584 | 10312 | 282 | 20329 | 178 | 13214 | 94.9 | 99.5 | 83.3 | - | - | - | - | - | - |
| Actin capping | GO:0051693 | 652 | 41797 | 128 | 39584 | 102 | 36797 | 95.1 | 99.99 | 93.3 | 73.3 | 99.9 | 55.0 | 91.2 | 99.9 | 71.0 |
| All DNA-binding | GO:0003677 | 3569 | 11426 | 5785 | 15685 | 3332 | 12962 | 90.4 | 85.6 | 77.8 | 81.9 | 90.2 | 61.4 | 69.0 | 98.1 | 85.2 |
| All lipid-binding proteins | GO:0008289 | 3232 | 10701 | 4017 | 14799 | 3566 | 12246 | 89.9 | 97.0 | 95.3 | - | - | - | - | - | - |
| Allergen | Not Available | 415 | 41518 | 112 | 39273 | 81 | 36530 | 85.2 | 99.9 | 60.5 | 74.1 | 100 | 79.8 | 86.4 | 99.9 | 69.3 |
| Amphibian defense peptide | GO:0006952 | 280 | 41852 | 14 | 39639 | 3 | 36854 | 100 | 100 | 75.0 | 100 | 100 | 66.7 | 66.7 | 100 | 25.0 |
| Antioxidant | GO:0016209 | 235 | 41798 | 126 | 39580 | 100 | 36799 | 89.0 | 100 | 89.9 | 69.1 | 99.9 | 73.7 | 86.0 | 99.9 | 78.9 |
| Biotin binding | Not Available | 76 | 41818 | 30 | 39607 | 18 | 36817 | 77.8 | 100 | 73.7 | 83.3 | 99.7 | 17.9 | - | - | - |
| Cadmium binding | Not Available | 83 | 41776 | 16 | 39564 | 10 | 36780 | 80.0 | 100 | 80.0 | 75.0 | 100 | 66.7 | - | - | - |
| Calcium-binding | GO:0005509 | 1816 | 11389 | 2300 | 16945 | 1310 | 14224 | 86.3 | 88.2 | 57.0 | - | - | - | - | - | - |
| Calmodulin-binding | GO:0005516 | 465 | 41405 | 223 | 39198 | 164 | 36421 | 87.2 | 100 | 90.5 | 70.0 | 99.4 | 41.6 | 82.9 | 99.9 | 84.0 |
| cAMP binding | Not Available | 1052 | 41777 | 722 | 39562 | 700 | 36778 | 97.3 | 100 | 98.6 | 85.6 | 99.8 | 86.2 | - | - | - |
| Cell adhesion | GO:0007155 | 513 | 8678 | 323 | 15223 | 270 | 11941 | 85.9 | 99.1 | 84.1 | - | - | - | - | - | - |
| cGMP binding | Not Available | 210 | 41820 | 73 | 39607 | 65 | 36821 | 98.5 | 100 | 98.5 | 82.2 | 99.8 | 42.0 | - | - | - |
| Chaperone | Not Available | 3938 | 41284 | 3292 | 39067 | 3105 | 36305 | 84.6 | 99.9 | 98.4 | 85.4 | 98.0 | 78.0 | 80.3 | 98.9 | 85.9 |
| Chitin-binding | GO:0008061 | 2462 | 41623 | 251 | 39389 | 211 | 36631 | 92.4 | 99.9 | 84.8 | 93.6 | 98.6 | 29.9 | 75.4 | 99.9 | 82.8 |
| Chloride binding | Not Available | 243 | 41750 | 134 | 39536 | 100 | 36754 | 92.0 | 100 | 92.0 | 71.6 | 99.9 | 62.3 | - | - | - |
| Chlorophyll | GO:0016168 | 189 | 7603 | 948 | 21640 | 529 | 13976 | 97.4 | 99.8 | 97.9 | - | - | - | - | - | - |
| Chlorophyll biosynthesis | GO:0015995 | 309 | 8742 | 109 | 20424 | 177 | 13935 | 86.4 | 88.8 | 16.5 | - | - | - | - | - | - |
| Chromatin regulator | GO:0016568 | 1131 | 41070 | 596 | 38675 | 403 | 36101 | 76.4 | 99.5 | 62.9 | 59.9 | 99.0 | 47.7 | 68.2 | 99.7 | 68.4 |
| Chromophore | GO:0018298 | 753 | 41724 | 480 | 39495 | 405 | 36732 | 93.8 | 99.9 | 93.8 | 89.4 | 99.5 | 68.2 | 86.9 | 99.9 | 88.4 |
| Coat proteins | GO:0030117 | 346 | 8474 | 305 | 15370 | 197 | 11914 | 84.8 | 99.4 | 85.2 | - | - | - | - | - | - |
| Cobalt-binding | GO:0050897 | 568 | 9151 | 458 | 20417 | 441 | 14816 | 81.6 | 99.9 | 98.1 | - | - | - | - | - | - |
| Collagen | GO:0005581 | 190 | 41801 | 103 | 39590 | 63 | 36803 | 96.8 | 100 | 96.8 | 84.5 | 100 | 92.6 | 96.8 | 99.9 | 74.4 |
| Copper-binding | GO:0005507 | 652 | 8999 | 526 | 20385 | 467 | 14733 | 83.5 | 98.1 | 72.8 | - | - | - | - | - | - |
| Covalent protein-RNA linkage | GO:0018144 | 58 | 41806 | 55 | 39597 | 54 | 36820 | 96.3 | 100 | 80.0 | 74.6 | 99.8 | 32.5 | 90.7 | 100 | 92.5 |
| Cyclin | GO:0061575 | 218 | 41847 | 95 | 39630 | 49 | 36848 | 98.0 | 100 | 88.9 | 74.7 | 99.8 | 52.2 | 89.8 | 99.9 | 66.7 |
| Cyclosporin | GO:0042277 | 66 | 41854 | 19 | 39644 | 16 | 36853 | 87.5 | 100 | 100 | 84.2 | 99.9 | 35.6 | 75.0 | 100 | 80.0 |
| Cytokine | GO:0005125 | 460 | 41720 | 334 | 39497 | 241 | 36734 | 91.3 | 99.9 | 89.8 | 70.7 | 99.9 | 80.0 | - | - | - |
| DNA condensation | GO:0030261 | 226 | 8999 | 191 | 20233 | 178 | 13970 | 87.6 | 99.5 | 80.4 | 83.2 | 99.9 | 72.7 | 69.8 | 99.9 | 66.4 |
| DNA integration | GO:0015074 | 410 | 8306 | 1761 | 20868 | 1040 | 13938 | 86.6 | 98.1 | 87.1 | 68.9 | 99.9 | 63.8 | 72.8 | 100 | 87.6 |
| DNA recombination | GO:0006310 | 1678 | 10614 | 3382 | 18224 | 2391 | 13763 | 85.7 | 97.4 | 92.1 | 67.5 | 99.3 | 80.3 | 77.6 | 98.9 | 77.0 |
| DNA repair | GO:0006281 | 2142 | 10643 | 1179 | 17646 | 1438 | 13544 | 88.7 | 96.8 | 85.9 | 67.6 | 96.8 | 68.0 | 64.3 | 99.3 | 90.4 |
| DNA replication | GO:0006260 | 1131 | 9603 | 933 | 18852 | 861 | 13636 | 85.6 | 96.6 | 76.8 | 59.7 | 98.5 | 66.6 | 76.8 | 98.9 | 77.6 |
| DNA replication inhibitor | GO:0008156 | 275 | 41835 | 247 | 39623 | 242 | 36835 | 97.9 | 100 | 97.9 | 93.9 | 99.9 | 83.8 | 97.1 | 99.9 | 91.1 |
| DNA-directed DNA polymerase | GO:0003887 | 825 | 9588 | 963 | 19524 | 869 | 13900 | 81.9 | 98.7 | 88.5 | 51.1 | 99.4 | 41.2 | 80.2 | 99.7 | 66.4 |
| DNA-directed RNA polymerase | GO:0003899 | 1618 | 10117 | 3041 | 18958 | 2271 | 13885 | 89.0 | 98.0 | 93.5 | - | - | - | - | - | - |
| Elongation factor | GO:0003746 | 1069 | 41788 | 938 | 39570 | 914 | 36788 | 97.5 | 100 | 98.8 | 95.8 | 99.6 | 83.7 | 84.1 | 99.9 | 94.0 |
| Envelope proteins | GO:0019031 | 177 | 8999 | 123 | 14932 | 150 | 11952 | 90.0 | 99.5 | 84.4 | - | - | - | - | - | - |
| Excision nuclease | GO:0004518 | 309 | 41842 | 306 | 39631 | 306 | 36841 | 97.4 | 100 | 99.0 | 95.8 | 99.5 | 58.8 | 93.5 | 99.9 | 91.4 |
| Eye lens protein | GO:0005212 | 109 | 41806 | 62 | 39597 | 50 | 36807 | 98.0 | 100 | 98.0 | 96.8 | 100 | 82.2 | 100 | 100 | 71.4 |
| Flavoprotein | GO:0045251 | 3542 | 41201 | 2150 | 38785 | 1930 | 36211 | 87.9 | 99.4 | 88.0 | 71.3 | 97.8 | 64.3 | 73.7 | 99.5 | 89.6 |
| Folate-binding | GO:0005542 | 361 | 41836 | 102 | 39625 | 99 | 36835 | 97.0 | 100 | 99.0 | 94.1 | 99.9 | 62.8 | 95.0 | 100 | 99.0 |
| G protein coupled receptors | GO:0004930 | 927 | 8320 | 4998 | 20216 | 2532 | 14244 | 95.6 | 98.1 | 94.5 | 96.6 | 98.9 | 64.1 | 94.1 | 99.9 | 93.4 |
| Growth factor | GO:0008083 | 423 | 41680 | 301 | 39458 | 243 | 36696 | 88.9 | 99.9 | 88.5 | 76.7 | 99.9 | 81.9 | 86.0 | 99.9 | 86.7 |
| GTPase activation | GO:0005096 | 429 | 41584 | 207 | 39359 | 113 | 36597 | 92.9 | 99.9 | 83.3 | 61.8 | 99.6 | 42.2 | 86.7 | 99.9 | 78.4 |
| Guanine-nucleotide releasing factor | GO:0005085 | 295 | 41599 | 161 | 39374 | 78 | 36607 | 83.3 | 100 | 77.4 | 93.8 | 99.2 | 31.7 | 89.7 | 99.9 | 55.6 |
| Heparin-binding | GO:0008201 | 182 | 41591 | 123 | 39344 | 92 | 36600 | 89.1 | 99.9 | 73.9 | 70.7 | 99.9 | 75.0 | 90.2 | 99.9 | 61.0 |
| Herbicide resistance | GO:0009635 | 227 | 8999 | 212 | 20198 | 209 | 13958 | 95.2 | 99.9 | 95.2 | 95.2 | 100 | 85.5 | 96.7 | 100 | 89.4 |
| Hormone | GO:0005179 | 686 | 41693 | 407 | 39463 | 333 | 36714 | 87.7 | 99.9 | 90.7 | 69.8 | 99.8 | 80.5 | 76.9 | 99.9 | 88.0 |
| Hypotensive agent | GO:0008217 | 75 | 41845 | 8 | 39635 | 3 | 36844 | 66.7 | 100 | 100 | 87.5 | 100 | 58.3 | 66.7 | 100 | 33.3 |
| Immune response | GO:0006955 | 447 | 9998 | 269 | 19563 | 152 | 14819 | 86.8 | 100 | 97.1 | - | - | - | - | - | - |
| Inflammatory response | GO:0006954 | 134 | 10320 | 98 | 19432 | 64 | 14902 | 87.5 | 99.8 | 73.7 | 55.2 | 99.8 | 59.2 | 75.4 | 99.7 | 47.0 |
| Initiation factor | GO:0003743 | 1122 | 41714 | 778 | 39457 | 729 | 36714 | 91.4 | 99.9 | 93.0 | 77.6 | 99.7 | 81.2 | 74.2 | 99.9 | 95.4 |
| Innate immunity | GO:0045087 | 193 | 10382 | 85 | 19285 | 58 | 14868 | 89.7 | 99.9 | 86.7 | 84.6 | 99.2 | 49.7 | 65.5 | 99.9 | 85.9 |
| Integrin | GO:0007229 | 54 | 41830 | 31 | 39621 | 22 | 36831 | 90.9 | 100 | 95.2 | 90.3 | 99.9 | 47.5 | 86.4 | 99.9 | 41.3 |
| Ion channel | GO:0005216 | 1142 | 41401 | 681 | 39136 | 531 | 36443 | 90.6 | 99.8 | 87.8 | 75.8 | 99.0 | 57.5 | - | - | - |
| Iron-binding | GO:0005506 | 3128 | 10428 | 5159 | 11667 | 1175 | 13926 | 94.0 | 91.4 | 64.9 | - | - | - | - | - | - |
| Lectin | GO:0030246 | 437 | 8513 | 35 | 15501 | 49 | 11993 | 81.6 | 85.7 | 5.3 | - | - | - | - | - | - |
| Lipid degradation | GO:0016042 | 403 | 8775 | 233 | 20635 | 237 | 14701 | 78.9 | 99.9 | 97.4 | 64.8 | 99.8 | 72.0 | 75.1 | 99.9 | 89.6 |
| Lipid metabolism | GO:0006629 | 293 | 8969 | 205 | 20400 | 161 | 14696 | 79.5 | 99.2 | 66.7 | 71.8 | 97.8 | 73.9 | 72.1 | 98.5 | 80.3 |
| Lipid synthesis | GO:0008610 | 891 | 9607 | 789 | 19670 | 703 | 14635 | 82.2 | 99.6 | 95.1 | - | - | - | - | - | - |
| Lipid transport | GO:0006869 | 153 | 9109 | 110 | 20462 | 78 | 14727 | 79.5 | 99.8 | 80.5 | 59.4 | 99.8 | 60.9 | 71.5 | 99.9 | 82.6 |
| Lipid-binding | GO:0008289 | 274 | 8530 | 166 | 20926 | 167 | 14724 | 84.4 | 99.9 | 93.4 | 72.8 | 99.6 | 71.2 | 66.9 | 99.7 | 72.1 |
| Lipopolysaccharide biosynthesis | GO:0009103 | 285 | 17837 | 143 | 11590 | 137 | 14723 | 76.6 | 99.8 | 89.0 | - | - | - | - | - | - |
| Lipoprotein | GO:0055097; GO:0055096 | 1648 | 12065 | 1792 | 15833 | 1657 | 12540 | 90.6 | 98.5 | 94.9 | - | - | - | - | - | - |
| Magnesium-binding | GO:0000287 | 2583 | 11023 | 2901 | 14382 | 4204 | 13653 | 81.2 | 87.9 | 80.9 | - | - | - | - | - | - |
| Manganese-binding | GO:0030145 | 1608 | 10099 | 1363 | 18927 | 1243 | 14563 | 85.4 | 94.5 | 71.9 | - | - | - | - | - | - |
| Metal-binding | GO:0046872 | 5013 | 10101 | 12821 | 11739 | 15461 | 12146 | 78.1 | 88.0 | 95.1 | - | - | - | - | - | - |
| Metalloenzyme inhibitor | GO:0004857 | 77 | 41812 | 41 | 39603 | 36 | 36813 | 83.3 | 100 | 93.8 | 70.7 | 100 | 63.0 | 88.9 | 100 | 80.0 |
| Milk protein | Not Available | 78 | 41851 | 54 | 39640 | 51 | 36850 | 92.2 | 100 | 97.9 | 74.1 | 100 | 87.0 | - | - | - |
| Mitogen | GO:0051781 | 888 | 41739 | 412 | 39504 | 372 | 36746 | 92.7 | 99.9 | 93.2 | 85.2 | 99.8 | 79.6 | 83.6 | 99.9 | 91.2 |
| Molybdenum binding | Not Available | 245 | 41752 | 144 | 39529 | 121 | 36752 | 84.3 | 100 | 92.7 | 70.8 | 99.7 | 43.2 | - | - | - |
| Motor protein | GO:0098840 | 479 | 41670 | 192 | 39443 | 114 | 36672 | 89.5 | 100 | 91.1 | 64.3 | 99.5 | 39.0 | 80.7 | 100 | 82.9 |
| mRNA capping | GO:0006370 | 60 | 8085 | 101 | 21817 | 52 | 14323 | 80.8 | 99.9 | 79.3 | 61.5 | 99.7 | 46.8 | 82.2 | 100 | 91.6 |
| mRNA slicing | Not Available | 621 | 9570 | 289 | 20046 | 220 | 14161 | 77.3 | 99.3 | 77.3 | - | - | - | - | - | - |
| mRNA-binding proteins | GO:0003729 | 277 | 9106 | 129 | 17164 | 164 | 13046 | 79.3 | 96.5 | 37.9 | - | - | - | - | - | - |
| Muscle protein | Not Available | 289 | 41760 | 173 | 39543 | 130 | 36763 | 95.4 | 100 | 90.5 | 79.8 | 99.9 | 76.7 | 89.2 | 99.9 | 77.3 |
| Mutator protein | GO:1990633 | 80 | 41854 | 70 | 39643 | 68 | 36854 | 83.8 | 100 | 98.3 | 70.0 | 100 | 73.1 | 77.9 | 99.9 | 62.4 |
| Neuropeptide | GO:0007218 | 232 | 41793 | 79 | 39566 | 58 | 36808 | 86.2 | 99.9 | 66.7 | 79.8 | 99.9 | 60.0 | 79.3 | 100 | 76.7 |
| Nickel-binding | GO:0016151 | 407 | 9001 | 97 | 20714 | 201 | 14881 | 77.6 | 99.2 | 70.6 | - | - | - | - | - | - |
| Nuclear receptors | GO:0003700 | 334 | 7538 | 608 | 8761 | 247 | 7986 | 89.5 | 97.6 | 90.2 | - | - | - | - | - | - |
| Outer membrane | GO:0009279 | 602 | 8539 | 547 | 15385 | 343 | 11948 | 92.7 | 86.4 | 32.1 | - | - | - | - | - | - |
| Pathogenesis-related protein | GO:0009607 | 1213 | 41816 | 446 | 39604 | 409 | 36818 | 96.3 | 100 | 98.0 | 91.3 | 99.9 | 91.7 | 94.9 | 99.9 | 94.2 |
| Photoreceptor | GO:0009881 | 354 | 8537 | 896 | 20622 | 590 | 13922 | 92.9 | 99.6 | 95.5 | 61.2 | 99.8 | 41.7 | 80.8 | 100 | 91.3 |
| Photorespiration | GO:0009853 | 368 | 8672 | 8201 | 20580 | 4270 | 13979 | 99.7 | 99.7 | 99.4 | 96.4 | 99.9 | 86.8 | 95.2 | 100 | 92.5 |
| Photosynthesis | GO:0015979 | 1054 | 8914 | 544 | 19997 | 657 | 13796 | 93.3 | 98.1 | 82.3 | 86.2 | 99.4 | 81.3 | 81.6 | 99.6 | 87.9 |
| Photosystem I | GO:0009522 | 264 | 8491 | 462 | 20727 | 334 | 13961 | 97.6 | 84.8 | 23.5 | 86.8 | 99.9 | 81.8 | 88.4 | 99.9 | 87.4 |
| Photosystem II | GO:0009523 | 506 | 7986 | 2022 | 21166 | 1223 | 13926 | 97.5 | 99.5 | 97.1 | - | - | - | - | - | - |
| Plant defense | GO:0006952 | 559 | 8830 | 461 | 20316 | 326 | 13917 | 88.7 | 99.1 | 82.8 | - | - | - | - | - | - |
| Plastoquinone binding | Not Available | 479 | 41815 | 459 | 39600 | 454 | 36814 | 96.7 | 100 | 98.7 | 93.5 | 99.8 | 82.0 | - | - | - |
| Porin | GO:0015288 | 182 | 41800 | 104 | 39588 | 82 | 36810 | 93.9 | 100 | 93.9 | 79.8 | 100 | 82.2 | 87.8 | 100 | 88.9 |
| Potassium-binding | GO:0030955 | 408 | 8845 | 499 | 20797 | 333 | 14851 | 90.4 | 100 | 98.7 | - | - | - | - | - | - |
| Protease inhibitor | GO:0030414 | 991 | 41633 | 280 | 39405 | 188 | 36648 | 79.8 | 99.9 | 85.2 | 66.8 | 99.7 | 63.4 | 75.5 | 99.6 | 47.2 |
| Protein phosphatase inhibitor | GO:0004864 | 49 | 41817 | 37 | 39605 | 25 | 36818 | 96.0 | 100 | 88.9 | 64.9 | 100 | 88.9 | - | - | - |
| Protein synthesis inhibitor | GO:0017148 | 531 | 41782 | 175 | 39563 | 165 | 36781 | 86.1 | 100 | 92.8 | 79.4 | 99.8 | 63.2 | 81.8 | 99.9 | 84.9 |
| RNA-binding proteins | GO:0003723 | 2161 | 9965 | 1850 | 13816 | 447 | 11881 | 97.8 | 96.0 | 69.0 | - | - | - | - | - | - |
| rRNA-binding proteins | GO:0019843 | 708 | 7972 | 1245 | 16044 | 101 | 11997 | 94.1 | 98.7 | 59.0 | 96.5 | 98.3 | 91.4 | 95.8 | 98.7 | 93.6 |
| Schiff base binding | Not Available | 908 | 41801 | 809 | 39566 | 779 | 36801 | 93.2 | 99.9 | 93.6 | 73.3 | 99.7 | 83.4 | - | - | - |
| Selenium binding | Not Available | 124 | 41811 | 100 | 39602 | 98 | 36813 | 91.8 | 100 | 96.8 | 74.0 | 100 | 78.7 | - | - | - |
| SH3-binding | GO:0017124 | 131 | 41617 | 98 | 39409 | 68 | 36630 | 94.1 | 100 | 95.5 | 58.2 | 99.9 | 65.5 | - | - | - |
| Sialic acid binding | Not Available | 55 | 41787 | 33 | 39578 | 21 | 36791 | 76.2 | 100 | 94.1 | 81.8 | 100 | 64.3 | 90.5 | 99.9 | 35.2 |
| Sigma factor | GO:0016987 | 101 | 41835 | 60 | 39616 | 54 | 36835 | 87.0 | 100 | 85.5 | 68.3 | 99.9 | 50.6 | 83.3 | 100 | 81.8 |
| Signal transduction inhibitor | GO:0009968 | 89 | 41800 | 54 | 39569 | 39 | 36801 | 84.6 | 99.9 | 49.3 | 87.0 | 99.9 | 65.3 | 84.6 | 100 | 80.5 |
| Sodium-binding | GO:0031402 | 777 | 9010 | 339 | 20621 | 451 | 14842 | 90.9 | 99.9 | 97.4 | - | - | - | - | - | - |
| Storage protein | GO:0045735 | 292 | 41790 | 22 | 39581 | 8 | 36796 | 50.0 | 100 | 40.0 | 63.6 | 99.9 | 23.0 | - | - | - |
| Structural proteins (Matrix protein,Core protein,Viral occlusion body,Keratin) | GO:0005198 | 858 | 8353 | 4981 | 15524 | 2656 | 11924 | 98.5 | 99.2 | 98.5 | - | - | - | - | - | - |
| Suppressor of RNA silencing | Not Available | 470 | 41782 | 446 | 39582 | 435 | 36802 | 95.4 | 100 | 98.6 | 93.1 | 99.0 | 51.9 | 91.0 | 100 | 99.8 |
| Thiamine pyrophosphate binding | Not Available | 494 | 41795 | 347 | 39572 | 317 | 36794 | 96.5 | 99.9 | 93.6 | 74.6 | 99.4 | 53.9 | 87.7 | 99.9 | 83.7 |
| Transmembrane | GO:0016021 | 2105 | 9563 | 12857 | 16605 | 3389 | 13063 | 90.1 | 86.7 | 79.1 | - | - | - | - | - | - |
| tRNA-binding proteins | GO:0000049 | 94 | 7792 | 114 | 16297 | 51 | 12033 | 94.1 | 99.9 | 90.6 | - | - | - | - | - | - |
| Ubiquinone binding | Not Available | 1206 | 41744 | 1117 | 39514 | 1079 | 36743 | 94.1 | 99.9 | 97.4 | 91.5 | 99.3 | 79.2 | 82.6 | 99.7 | 88.1 |
| Vasoactive | GO:0050880 | 340 | 41828 | 138 | 39615 | 119 | 36832 | 95.0 | 100 | 97.4 | 86.2 | 100 | 88.2 | 93.3 | 100 | 86.1 |
| Viral nucleoprotein | GO:0019013 | 241 | 41749 | 234 | 39547 | 228 | 36786 | 89.9 | 100 | 91.9 | 79.1 | 99.9 | 77.7 | 87.7 | 100 | 96.2 |
| Virulent protein | GO:0009405 | 544 | 9983 | 270 | 19295 | 173 | 14671 | 95.4 | 99.9 | 96.5 | 64.0 | 99.1 | 49.2 | 70.3 | 99.9 | 87.7 |
| Vitamin C binding | GO:0031418 | 247 | 41821 | 49 | 39612 | 37 | 36821 | 91.9 | 100 | 100 | 81.6 | 99.9 | 61.5 | 97.3 | 99.9 | 47.4 |
| Zinc-binding | GO:0008270 | 2731 | 13416 | 7179 | 13291 | 6162 | 13416 | 74.9 | 98.0 | 97.3 | - | - | - | - | - | - |
| Zinc-finger | GO:0070742; GO:0071535 | 6345 | 39878 | 3387 | 37681 | 2509 | 34979 | 56.3 | 99.9 | 97.8 | 71.6 | 96.8 | 66.8 | 78.4 | 97.3 | 67.9 |
