# Supplementary material for: SVM-Prot 2016: A Web-Server for Machine Learning Prediction of Protein Functional Families from Sequence Irrespective of Similarity
Source: PLoS One. 2016 Aug 15;11(8):e0155290. doi: 10.1371/journal.pone.0155290 (PMC4985167; doi:10.1371/journal.pone.0155290)
Supplement: S3 Table — (DOCX) [file pone.0155290.s003.docx]

**Table S3.** List of the novel proteins published in 2015 and 2014 that are not in the SVMProt training sets and have available sequence in the literature or public databases. Their sources of publications and sequences, and the prediction results by SVMProt, FFPred, and NCBI BLAST are also provided.

| **Protein name** | **Reported Protein Function** | **Ref** | **Year of Publish** | **Sequence Source** | **Sequence ID** | **Computer Prediction Results** | | | | | | |
| --- | --- | --- | --- | --- | --- | --- | --- | --- | --- | --- | --- | --- |
|  |  |  |  |  |  | **SVMProt** | | **FFPred** | | | **Blast** | |
|  |  |  |  |  |  | **Rank of Matched Family** | **Total No of Predicted Families** | **Rank of Matched Family** | **No of Predicted Families (H)** | **Total No of Predicted Families** | **Rank of Matched Family** | **Total No of Predicted Families** |
| PfFNT | Formate-nitrite transporter; TC1.A.16 | [[1](#_ENREF_1)] | 2015 | Uniprot | O77389 | 0 | 11 | 0 | 24 | 79 | 2/10 | 10 |
| Gp001 | DNA Binding Proteins | [[2](#_ENREF_2)] | 2015 | NCBI Protein | 332672313 | 0 | 3 | 0 | 41 | 77 | 0 | 0 |
| SSX2 | DNA Binding Protein | [[3](#_ENREF_3)] | 2014 | Uniprot | Q16385 | 0 | 5 | 1/69 | 25 | 69 | 1/27 | 27 |
| HLP1 | RNA Binding Proteins | [[4](#_ENREF_4)] | 2015 | Uniprot | Q9FM47 | 5/8 | 8 | 1 | 48 | 90 | 4/93 | 93 |
| LIX1L | RNA Binding Proteins | [[5](#_ENREF_5)] | 2015 | Uniprot | Q8IVB5 | 0 | 6 | 0 | 31 | 75 | 1/5 | 5 |
| P311 | RNA Binding Proteins | [[6](#_ENREF_6)] | 2014 | Uniprot | Q16612 | 0 | 7 | 0 | 39 | 83 | 1/5 | 5 |
| MdSHT–KP698511 | Enzyme; EC2.3 | [[7](#_ENREF_7)] | 2015 | NCBI Protein | 928510949 | 0 | 9 | 1 | 51 | 88 | 1/46 | 46 |
| BatroxPLA2 | Enzyme; EC3.1 | [[8](#_ENREF_8)] | 2015 | NCBI Protein | 129420 | 1/14 | 14 | 63/94 | 49 | 94 | 1/99 | 99 |
| maheshvara | Enzyme; EC3.6 | [[9](#_ENREF_9)] | 2015 | NCBI Protein | 16648356 | 0 | 10 | 0 | 41 | 79 | 1/100 | 100 |
| Orf1 | Enzyme; EC3.2 | [[10](#_ENREF_10)] | 2015 | NCBI Protein | 166159382 | 0 | 14 | 0 | 49 | 86 | 0 | 0 |
| UdgX | Enzyme; EC3.2 | [[11](#_ENREF_11)] | 2015 | Uniprot | A0QP43 | 0 | 8 | 0 | 47 | 90 | 0 | 0 |
| NCBI-XP_414028 | Enzyme; EC1.1 | [[12](#_ENREF_12)] | 2015 | NCBI Protein | 50753535 | 5/13 | 13 | 54/90 | 55 | 90 | 10/75 | 75 |
| S-Inosyl-L-Homocysteine Hydrolase | Enzyme; EC3.3 | [[13](#_ENREF_13)] | 2015 | Uniprot | Q58783 | 2/10 | 10 | 30/74 | 36 | 74 | 1/99 | 99 |
| E3 UFM1-protein ligase 1 | Enzyme; EC6.3 | [[14](#_ENREF_14)] | 2015 | Uniprot | B2GV24 | 0 | 6 | 0 | 34 | 72 | 1/28 | 28 |
| Endoglucanase | Enzyme; EC3.2 | [[15](#_ENREF_15)] | 2015 | Uniprot | O74705 | 1/4 | 4 | 51/52 | 25 | 52 | 1/15 | 15 |
| XYN VI | Enzyme; EC3.2 | [[16](#_ENREF_16)] | 2014 | Uniprot | G0RV92 | 1/8 | 8 | 37/66 | 32 | 66 | 1/2 | 2 |
| L-lysine decarboxylase/oxidase | Enzyme; EC4.1 | [[17](#_ENREF_17)] | 2014 | DDBJ | AB893919 | 1/5 | 5 | 0 | 37 | 74 | 2/18 | 18 |
| AcL-RbI | Enzyme; EC5.3 | [[18](#_ENREF_18)] | 2014 | Uniprot | Q93UQ5 | 0 | 8 | 0 | 32 | 69 | 0 | 0 |
| AtGALT29A | enzyme; EC2.4 | [[19](#_ENREF_19)] | 2014 | Uniprot | Q84W00 | 0 | 5 | 7/100 | 54 | 100 | 0 | 0 |
| Lhyal | Enzyme; EC3.2 | [[20](#_ENREF_20)] | 2014 | Uniprot | X4Y2L4 | 1/6 | 6 | 27/57 | 22 | 57 | 0 | 11 |
| Ver3Phr | Enzyme; EC4.1 | [[21](#_ENREF_21)] | 2014 | DDBJ | HQ443199 | 4/6 | 6 | 0 | 35 | 74 | 1/56 | 56 |
| Esterase | Enzyme; EC3.1 | [[22](#_ENREF_22)] | 2015 | Uniprot | P71778 | 0 | 10 | 68/81 | 49 | 81 | 0 | 1 |
| Fatty acid synthase | Enzyme; EC2.3 | [[23](#_ENREF_23)] | 2015 | Uniprot | Q3J1G9 | 0 | 4 | 0 | 39 | 71 | 0 | 0 |
| Agarase | Enzyme; EC3.2 | [[24](#_ENREF_24)] | 2015 | NCBI Protein | LC034562 | 1/4 | 4 | 0 | 33 | 63 | 1/18 | 18 |
| MnSOD1 | Enzyme; EC1.11 | [[25](#_ENREF_25)] | 2015 | Reference | NA | 0 | 5 | 0 | 16 | 45 | 1/100 | 100 |
| MnSOD2 | Enzyme; EC1.11 | [[25](#_ENREF_25)] | 2015 | Reference | NA | 0 | 12 | 0 | 34 | 74 | 1/100 | 100 |
| Metallo-beta-lactamase superfamily protein | Enzyme; EC1.13 | [[26](#_ENREF_26)] | 2015 | Uniprot | A0A0H3K9V0 | 0 | 18 | 0 | 33 | 69 | 8/100 | 100 |
| flavin prenyltransferase | Enzyme; EC2.5 | [[27](#_ENREF_27)] | 2015 | NCBI Protein | NP_252708.1 | 0 | 10 | 45/90 | 50 | 90 | 0 | 33 |
| alpha-galactosidase | Enzyme; EC3.2 | [[28](#_ENREF_28)] | 2015 | NCBI Protein | LC019121 | 1/8 | 8 | 0 | 27 | 52 | 0 | 50 |
| esterase | Enzyme; EC3.1 | [[29](#_ENREF_29)] | 2015 | NCBI Protein | KF994924 | 0 | 10 | 0 | 30 | 67 | 4/96 | 96 |
| dusA-associated integrases | Enzyme; EC2.7 | [[30](#_ENREF_30)] | 2015 | NCBI Protein | ABQ35548 | 0 | 8 | 0 | 48 | 91 | 3/6 | 6 |
| dusA-associated integrases | Enzyme; EC2.7 | [[30](#_ENREF_30)] | 2015 | NCBI Protein | AEA60511 | 0 | 9 | 0 | 46 | 92 | 3/7 | 7 |
| dusA-associated integrases | Enzyme; EC2.7 | [[30](#_ENREF_30)] | 2015 | NCBI Protein | AAY91263 | 0 | 8 | 0 | 52 | 97 | 1/5 | 5 |
| phage integrase family protein | Enzyme; EC2.7 | [[30](#_ENREF_30)] | 2015 | NCBI Protein | ADY64412 | 0 | 13 | 0 | 50 | 94 | 3/3 | 3 |
| dusA-associated integrases | Enzyme; EC2.7 | [[30](#_ENREF_30)] | 2015 | NCBI Protein | AAW90241 | 2/10 | 10 | 2/101 | 55 | 101 | 3/4 | 4 |
| dusA-associated integrases | Enzyme; EC2.7 | [[30](#_ENREF_30)] | 2015 | NCBI Protein | AGH34419 | 3/7 | 7 | 5/101 | 54 | 101 | 1/5 | 5 |
| SimC7 | Enzyme; EC1.1 | [[31](#_ENREF_31)] | 2015 | NCBI Protein | 358681267 | 0 | 8 | 0 | 53 | 87 | 0 | 14 |
| RecD | Enzyme; EC3.6 | [[32](#_ENREF_32)] | 2015 | NCBI Protein | 15607769 | 0 | 6 | 0 | 46 | 77 | 1/15 | 15 |
| CLA-ER | Enzyme; EC1.3 | [[33](#_ENREF_33)] | 2015 | NCBI Protein | BAO04454.1 | 0 | 10 | 0 | 47 | 91 | 0 | 21 |
| FadE34 | Enzyme; EC1.3 | [[34](#_ENREF_34)] | 2015 | NCBI Protein | 15610709 | 0 | 9 | 0 | 31 | 69 | 1/70 | 70 |
| possible acyl-CoA dehydrogenase | Enzyme; EC1.3 | [[34](#_ENREF_34)] | 2015 | NCBI Protein | 110822309 | 0 | 8 | 0 | 29 | 65 | 1/112 | 112 |
| RAS3 | Enzyme; EC3.6 | [[35](#_ENREF_35)] | 2015 | NCBI Protein | EJP70406.1 | 0 | 11 | 0 | 23 | 60 | 1/100 | 100 |

**References**

1. Wu B, Rambow J, Bock S, Holm-Bertelsen J, Wiechert M, et al. (2015) Identity of a Plasmodium lactate/H(+) symporter structurally unrelated to human transporters. Nat Commun 6: 6284.

2. Arutyunov D, Szymanski CM (2015) A novel DNA binding protein from Campylobacter jejuni bacteriophage NCTC12673. FEMS Microbiol Lett.

3. Gjerstorff MF, Relster MM, Greve KB, Moeller JB, Elias D, et al. (2014) SSX2 is a novel DNA-binding protein that antagonizes polycomb group body formation and gene repression. Nucleic Acids Res 42: 11433-11446.

4. Zhang Y, Gu L, Hou Y, Wang L, Deng X, et al. (2015) Integrative genome-wide analysis reveals HLP1, a novel RNA-binding protein, regulates plant flowering by targeting alternative polyadenylation. Cell Res 25: 864-876.

5. Nakamura S, Kahyo T, Tao H, Shibata K, Kurabe N, et al. (2015) Novel roles for LIX1L in promoting cancer cell proliferation through ROS1-mediated LIX1L phosphorylation. Sci Rep 5: 13474.

6. Yue MM, Lv K, Meredith SC, Martindale JL, Gorospe M, et al. (2014) Novel RNA-binding protein P311 binds eukaryotic translation initiation factor 3 subunit b (eIF3b) to promote translation of transforming growth factor beta1-3 (TGF-beta1-3). J Biol Chem 289: 33971-33983.

7. Elejalde-Palmett C, de Bernonville TD, Glevarec G, Pichon O, Papon N, et al. (2015) Characterization of a spermidine hydroxycinnamoyltransferase in Malus domestica highlights the evolutionary conservation of trihydroxycinnamoyl spermidines in pollen coat of core Eudicotyledons. J Exp Bot.

8. Menaldo DL, Jacob-Ferreira AL, Bernardes CP, Cintra AC, Sampaio SV (2015) Purification procedure for the isolation of a P-I metalloprotease and an acidic phospholipase A2 from Bothrops atrox snake venom. J Venom Anim Toxins Incl Trop Dis 21: 28.

9. Surabhi S, Tripathi BK, Maurya B, Bhaskar PK, Mukherjee A, et al. (2015) Regulation of Notch Signalling by an Evolutionary Conserved DEAD Box RNA Helicase, Maheshvara in Drosophila melanogaster. Genetics.

10. Wang S, Liu K, Xiao L, Yang L, Li H, et al. (2015) Characterization of a novel DNA glycosylase from S. sahachiroi involved in the reduction and repair of azinomycin B induced DNA damage. Nucleic Acids Res.

11. Sang PB, Srinath T, Patil AG, Woo EJ, Varshney U (2015) A unique uracil-DNA binding protein of the uracil DNA glycosylase superfamily. Nucleic Acids Res 43: 8452-8463.

12. Fukuda Y, Sone T, Sakuraba H, Araki T, Ohshima T, et al. (2015) A novel NAD(P)H-dependent carbonyl reductase specifically expressed in the thyroidectomized chicken fatty liver: catalytic properties and crystal structure. FEBS J.

13. Miller D, Xu H, White RH (2015) S-Inosyl-L-Homocysteine Hydrolase, a Novel Enzyme Involved in S-Adenosyl-L-Methionine Recycling. J Bacteriol 197: 2284-2291.

14. Zhang M, Zhu X, Zhang Y, Cai Y, Chen J, et al. (2015) RCAD/Ufl1, a Ufm1 E3 ligase, is essential for hematopoietic stem cell function and murine hematopoiesis. Cell Death Differ.

15. Rawat R, Kumar S, Chadha BS, Kumar D, Oberoi HS (2015) An acidothermophilic functionally active novel GH12 family endoglucanase from Aspergillus niger HO: purification, characterization and molecular interaction studies. Antonie Van Leeuwenhoek 107: 103-117.

16. Biely P, Puchart V, Stringer MA, Morkeberg Krogh KB (2014) Trichoderma reesei XYN VI--a novel appendage-dependent eukaryotic glucuronoxylan hydrolase. FEBS J 281: 3894-3903.

17. Sugawara A, Matsui D, Takahashi N, Yamada M, Asano Y, et al. (2014) Characterization of a pyridoxal-5'-phosphate-dependent l-lysine decarboxylase/oxidase from Burkholderia sp. AIU 395. J Biosci Bioeng 118: 496-501.

18. Yoshida H, Yoshihara A, Teraoka M, Terami Y, Takata G, et al. (2014) X-ray structure of a novel L-ribose isomerase acting on a non-natural sugar L-ribose as its ideal substrate. FEBS J 281: 3150-3164.

19. Dilokpimol A, Poulsen CP, Vereb G, Kaneko S, Schulz A, et al. (2014) Galactosyltransferases from Arabidopsis thaliana in the biosynthesis of type II arabinogalactan: molecular interaction enhances enzyme activity. BMC Plant Biol 14: 90.

20. Jin P, Kang Z, Zhang N, Du G, Chen J (2014) High-yield novel leech hyaluronidase to expedite the preparation of specific hyaluronan oligomers. Sci Rep 4: 4471.

21. Albarracin VH, Simon J, Pathak GP, Valle L, Douki T, et al. (2014) First characterisation of a CPD-class I photolyase from a UV-resistant extremophile isolated from High-Altitude Andean Lakes. Photochem Photobiol Sci 13: 739-750.

22. Cao J, Dang G, Li H, Li T, Yue Z, et al. (2015) Identification and Characterization of Lipase Activity and Immunogenicity of LipL from Mycobacterium tuberculosis. PLoS One 10: e0138151.

23. Mao YH, Ma JC, Li F, Hu Z, Wang HH (2015) Ralstonia solanacearum RSp0194 Encodes a Novel 3-Keto-Acyl Carrier Protein Synthase III. PLoS One 10: e0136261.

24. Tawara M, Sakatoku A, Tiodjio RE, Tanaka D, Nakamura S (2015) Cloning and Characterization of a Novel Agarase from a Newly Isolated Bacterium Simiduia sp. Strain TM-2 Able to Degrade Various Seaweeds. Appl Biochem Biotechnol 177: 610-623.

25. Rashid GM, Taylor CR, Liu Y, Zhang X, Rea D, et al. (2015) Identification of Manganese Superoxide Dismutase from Sphingobacterium sp. T2 as a Novel Bacterial Enzyme for Lignin Oxidation. ACS Chem Biol.

26. Shen J, Keithly ME, Armstrong RN, Higgins KA, Edmonds KA, et al. (2015) Staphylococcus aureus CstB Is a Novel Multidomain Persulfide Dioxygenase-Sulfurtransferase Involved in Hydrogen Sulfide Detoxification. Biochemistry 54: 4542-4554.

27. White MD, Payne KA, Fisher K, Marshall SA, Parker D, et al. (2015) UbiX is a flavin prenyltransferase required for bacterial ubiquinone biosynthesis. Nature 522: 502-506.

28. Miyazaki T, Ishizaki Y, Ichikawa M, Nishikawa A, Tonozuka T (2015) Structural and biochemical characterization of novel bacterial alpha-galactosidases belonging to glycoside hydrolase family 31. Biochem J 469: 145-158.

29. De Santi C, Ambrosino L, Tedesco P, de Pascale D, Zhai L, et al. (2015) Identification and characterization of a novel salt-tolerant esterase from a Tibetan glacier metagenomic library. Biotechnol Prog 31: 890-899.

30. Farrugia DN, Elbourne LD, Mabbutt BC, Paulsen IT (2015) A novel family of integrases associated with prophages and genomic islands integrated within the tRNA-dihydrouridine synthase A (dusA) gene. Nucleic Acids Res 43: 4547-4557.

31. Schafer M, Le TB, Hearnshaw SJ, Maxwell A, Challis GL, et al. (2015) SimC7 Is a Novel NAD(P)H-Dependent Ketoreductase Essential for the Antibiotic Activity of the DNA Gyrase Inhibitor Simocyclinone. J Mol Biol 427: 2192-2204.

32. Dewhare SS, Umesh TG, Muniyappa K (2015) Molecular and Functional Characterization of RecD, a Novel Member of the SF1 Family of Helicases, from Mycobacterium tuberculosis. J Biol Chem 290: 11948-11968.

33. Hou F, Miyakawa T, Kitamura N, Takeuchi M, Park SB, et al. (2015) Structure and reaction mechanism of a novel enone reductase. FEBS J 282: 1526-1537.

34. Ruprecht A, Maddox J, Stirling AJ, Visaggio N, Seah SY (2015) Characterization of novel acyl coenzyme A dehydrogenases involved in bacterial steroid degradation. J Bacteriol 197: 1360-1367.

35. Guan Y, Wang DY, Ying SH, Feng MG (2015) A novel Ras GTPase (Ras3) regulates conidiation, multi-stress tolerance and virulence by acting upstream of Hog1 signaling pathway in Beauveria bassiana. Fungal Genet Biol 82: 85-94.
