# Supplementary material for: SVM-Prot 2016: A Web-Server for Machine Learning Prediction of Protein Functional Families from Sequence Irrespective of Similarity
Source: PLoS One. 2016 Aug 15;11(8):e0155290. doi: 10.1371/journal.pone.0155290 (PMC4985167; doi:10.1371/journal.pone.0155290)
Supplement: S4 Table — (DOCX) [file pone.0155290.s004.docx]

**Table S4.** The detailed results of the prediction of the functional families of the 42 novel proteins by SVMProt, FFPred and NCBI BLAST. The correctly predicted functional families are marked by the green background color.

**Protein name: PfFNT**

**Predicted Results:**

**SVMProt**

| **Protein Family Name** | **GO Category** | **SVM** | **PNN** | **KNN** |
| --- | --- | --- | --- | --- |
| **Molecular Function** | | | | |
| TC2.A Electrochemical Potential-driven transporters - Porters (uniporters, symporters, antiporters) | - | 80.4 | Y | Y |
| Iron-binding | GO:0005506 iron binding | 94.2 | - | - |
| All lipid-binding proteins | GO:0008289 lipid binding | 76.2 | - | - |
| G protein coupled receptors | GO:0004930 G-protein coupled receptor activity; GO:0007186 G-protein coupled receptor signaling pathway | 65.4 | ? | ? |
| Metal-binding | GO:0046872 metal ion binding | 65.4 | - | - |
| TC3.A.5 Type II (general) secretory pathway (IISP) family | - | 58.6 | ? | ? |
| TC2.A.1 Major facilitator family (MFS) | - | 58.6 | ? | ? |
| 7 transmembrane receptor (odorant receptor) | - | 58.6 | ? | ? |
| Calcium-binding | - | 58.6 | - | - |
| Copper-binding | GO:0005507 copper binding | 58.6 | - | - |
| **Broadly Defined Function** | | | | |
| Transmembrane | GO:0016021 integral component of membrane | 99.2 | - | Y |

**FFPred**

| **Score** | **GO term** | **RL** | **Domain** | **Description** |
| --- | --- | --- | --- | --- |
| 1 | GO:0016021 | H | CC | integral_component_of_membrane |
| 1 | GO:0031224 | H | CC | intrinsic_component_of_membrane |
| 0.973 | GO:0016020 | H | CC | membrane |
| 0.969 | GO:0005887 | H | CC | integral_component_of_plasma_membrane |
| 0.937 | GO:0005886 | H | CC | plasma_membrane |
| 0.926 | GO:0022857 | H | MF | transmembrane_transporter_activity |
| 0.923 | GO:0031226 | H | CC | intrinsic_component_of_plasma_membrane |
| 0.918 | GO:0005215 | H | MF | transporter_activity |
| 0.91 | GO:0071944 | H | CC | cell_periphery |
| 0.898 | GO:0006810 | H | BP | transport |
| 0.88 | GO:0022891 | H | MF | substrate-specific_transmembrane_transporter_activity |
| 0.868 | GO:0004872 | H | MF | receptor_activity |
| 0.864 | GO:0055085 | H | BP | transmembrane_transport |
| 0.812 | GO:0031301 | H | CC | integral_component_of_organelle_membrane |
| 0.785 | GO:0004871 | H | MF | signal_transducer_activity |
| 0.78 | GO:0006820 | H | BP | anion_transport |
| 0.778 | GO:0098655 | H | BP | cation_transmembrane_transport |
| 0.774 | GO:0098588 | H | CC | bounding_membrane_of_organelle |
| 0.768 | GO:0022890 | H | MF | inorganic_cation_transmembrane_transporter_activity |
| 0.763 | GO:0015075 | H | MF | ion_transmembrane_transporter_activity |
| 0.761 | GO:0012505 | H | CC | endomembrane_system |
| 0.744 | GO:0038023 | H | MF | signaling_receptor_activity |
| 0.743 | GO:0050877 | H | BP | neurological_system_process |
| 0.724 | GO:0007166 | H | BP | cell_surface_receptor_signaling_pathway |
| 0.706 | GO:0031090 | H | CC | organelle_membrane |
| 0.701 | GO:0004888 | H | MF | transmembrane_signaling_receptor_activity |
| 0.699 | GO:0019222 | H | BP | regulation_of_metabolic_process |
| 0.682 | GO:0015711 | H | BP | organic_anion_transport |
| 0.681 | GO:0016192 | H | BP | vesicle-mediated_transport |
| 0.674 | GO:0007186 | H | BP | G-protein_coupled_receptor_signaling_pathway |
| 0.662 | GO:0070062 | H | CC | extracellular_vesicular_exosome |
| 0.66 | GO:0015031 | H | BP | protein_transport |
| 0.649 | GO:0034220 | H | BP | ion_transmembrane_transport |
| 0.64 | GO:0005789 | H | CC | endoplasmic_reticulum_membrane |
| 0.639 | GO:0004930 | H | MF | G-protein_coupled_receptor_activity |
| 0.633 | GO:0006811 | H | BP | ion_transport |
| 0.625 | GO:0006812 | H | BP | cation_transport |
| 0.617 | GO:0003824 | H | MF | catalytic_activity |
| 0.616 | GO:0031982 | H | CC | vesicle |
| 0.612 | GO:0045184 | H | BP | establishment_of_protein_localization |
| 0.61 | GO:0015267 | H | MF | channel_activity |
| 0.609 | GO:0005783 | H | CC | endoplasmic_reticulum |
| 0.594 | GO:0051649 | H | BP | establishment_of_localization_in_cell |
| 0.592 | GO:0005125 | H | MF | cytokine_activity |
| 0.577 | GO:0051641 | H | BP | cellular_localization |
| 0.575 | GO:0002376 | H | BP | immune_system_process |
| 0.57 | GO:0044281 | H | BP | small_molecule_metabolic_process |
| 0.565 | GO:0001664 | H | MF | G-protein_coupled_receptor_binding |
| 0.559 | GO:0000139 | H | CC | Golgi_membrane |
| 0.559 | GO:0044255 | H | BP | cellular_lipid_metabolic_process |
| 0.52 | GO:0030001 | H | BP | metal_ion_transport |
| 0.51 | GO:0042175 | H | CC | nuclear_outer_membrane-endoplasmic_reticulum_membrane_network |
| 0.506 | GO:0031988 | H | CC | membrane-bounded_vesicle |
| 0.502 | GO:0031253 | H | CC | cell_projection_membrane |
| 0.501 | GO:0016323 | H | CC | basolateral_plasma_membrane |
| 0.93 | GO:0050896 | L | BP | response_to_stimulus |
| 0.9 | GO:0023052 | L | BP | signaling |
| 0.863 | GO:0043231 | L | CC | intracellular_membrane-bounded_organelle |
| 0.859 | GO:0007165 | L | BP | signal_transduction |
| 0.837 | GO:0051716 | L | BP | cellular_response_to_stimulus |
| 0.816 | GO:0007154 | L | BP | cell_communication |
| 0.781 | GO:0005737 | L | CC | cytoplasm |
| 0.756 | GO:0043229 | L | CC | intracellular_organelle |
| 0.752 | GO:0032502 | L | BP | developmental_process |
| 0.703 | GO:0009966 | L | BP | regulation_of_signal_transduction |
| 0.693 | GO:0048856 | L | BP | anatomical_structure_development |
| 0.641 | GO:0042592 | L | BP | homeostatic_process |
| 0.603 | GO:0005102 | L | MF | receptor_binding |
| 0.594 | GO:0007275 | L | BP | multicellular_organismal_development |
| 0.584 | GO:0043234 | L | CC | protein_complex |
| 0.565 | GO:0009893 | L | BP | positive_regulation_of_metabolic_process |
| 0.56 | GO:0006807 | L | BP | nitrogen_compound_metabolic_process |
| 0.541 | GO:0032991 | L | CC | macromolecular_complex |
| 0.531 | GO:0008152 | L | BP | metabolic_process |
| 0.52 | GO:0010033 | L | BP | response_to_organic_substance |
| 0.515 | GO:0030154 | L | BP | cell_differentiation |
| 0.513 | GO:0005794 | L | CC | Golgi_apparatus |
| 0.51 | GO:0019538 | L | BP | protein_metabolic_process |
| 0.507 | GO:0009058 | L | BP | biosynthetic_process |

**Blast**

| **BLAST Matched UniProt ID & name** | | | **Identity** | **E Value** | **Score** |
| --- | --- | --- | --- | --- | --- |
| O05399 | YRHG_BACSU | Uncharacterized transporter YrhG | 27.562 | 1.67E-30 | 120 |
| Q50568 | FDHC_METTF | Probable formate transporter | 30.374 | 2.16E-27 | 112 |
| P35839 | FDHC_METFO | Probable formate transporter | 28.947 | 1.30E-26 | 110 |
| P39608 | YWCJ_BACSU | Uncharacterized transporter YwcJ | 30.859 | 4.05E-25 | 105 |
| P0AC25 | FOCA_ECO57 | Probable formate transporter 1 | 28.182 | 1.96E-18 | 86.3 |
| P43756 | FOCA_HAEIN | Probable formate transporter | 24.731 | 8.30E-18 | 84.7 |
| P0AC26 | NIRC_ECOLI | Nitrite transporter NirC | 26.545 | 2.53E-17 | 83.2 |
| P77733 | FOCB_ECOLI | Probable formate transporter 2 | 26.484 | 3.11E-16 | 80.1 |
| P25926 | NIRC_SALTY | Nitrite transporter NirC | 25 | 1.32E-15 | 78.2 |
| P38750 | YHA8_YEAST | Uncharacterized transporter YHL008C | 26.506 | 1.97E-15 | 80.1 |

**Protein Name: Gp001**

**Predicted Results:**

**SVMProt**

| **Protein Family Name** | **GO Category** | **SVM** | **PNN** | **KNN** |
| --- | --- | --- | --- | --- |
| **Molecular Function** | | | | |
| EC2.7 Transferases - Transferring Phosphorus-Containing Groups | - | 71.3 | - | Y |
| EC1.6 Oxidoreductases - Acting on NADH or NADPH | - | 76.2 | - | - |
| TC3.A Primary Active Transporters - P-P-bond-hydrolysis-driven transporters | - | 58.6 | - | - |

**FFPred**

| **Score** | **GO term** | **RL** | **Domain** | **Description** |
| --- | --- | --- | --- | --- |
| 0.907 | GO:0003824 | H | MF | catalytic_activity |
| 0.901 | GO:0005576 | H | CC | extracellular_region |
| 0.835 | GO:0016740 | H | MF | transferase_activity |
| 0.819 | GO:0031982 | H | CC | vesicle |
| 0.798 | GO:0070062 | H | CC | extracellular_vesicular_exosome |
| 0.791 | GO:0000166 | H | MF | nucleotide_binding |
| 0.782 | GO:0016817 | H | MF | hydrolase_activity,_acting_on_acid_anhydrides |
| 0.76 | GO:0017076 | H | MF | purine_nucleotide_binding |
| 0.758 | GO:0044281 | H | BP | small_molecule_metabolic_process |
| 0.755 | GO:0006082 | H | BP | organic_acid_metabolic_process |
| 0.754 | GO:0005739 | H | CC | mitochondrion |
| 0.741 | GO:0034645 | H | BP | cellular_macromolecule_biosynthetic_process |
| 0.728 | GO:0035639 | H | MF | purine_ribonucleoside_triphosphate_binding |
| 0.728 | GO:0030554 | H | MF | adenyl_nucleotide_binding |
| 0.718 | GO:0001882 | H | MF | nucleoside_binding |
| 0.708 | GO:0031988 | H | CC | membrane-bounded_vesicle |
| 0.706 | GO:0001883 | H | MF | purine_nucleoside_binding |
| 0.698 | GO:0032549 | H | MF | ribonucleoside_binding |
| 0.694 | GO:0016788 | H | MF | hydrolase_activity,_acting_on_ester_bonds |
| 0.683 | GO:0016818 | H | MF | hydrolase_activity,_acting_on_acid_anhydrides,_in_phosphorus-containing_anhydrides |
| 0.683 | GO:0009056 | H | BP | catabolic_process |
| 0.663 | GO:0003676 | H | MF | nucleic_acid_binding |
| 0.644 | GO:0006796 | H | BP | phosphate-containing_compound_metabolic_process |
| 0.637 | GO:0019222 | H | BP | regulation_of_metabolic_process |
| 0.634 | GO:0044255 | H | BP | cellular_lipid_metabolic_process |
| 0.63 | GO:0005524 | H | MF | ATP_binding |
| 0.626 | GO:0006520 | H | BP | cellular_amino_acid_metabolic_process |
| 0.617 | GO:0009059 | H | BP | macromolecule_biosynthetic_process |
| 0.612 | GO:0019637 | H | BP | organophosphate_metabolic_process |
| 0.596 | GO:0019752 | H | BP | carboxylic_acid_metabolic_process |
| 0.594 | GO:0017111 | H | MF | nucleoside-triphosphatase_activity |
| 0.583 | GO:0055086 | H | BP | nucleobase-containing_small_molecule_metabolic_process |
| 0.583 | GO:0016462 | H | MF | pyrophosphatase_activity |
| 0.55 | GO:1901605 | H | BP | alpha-amino_acid_metabolic_process |
| 0.543 | GO:0051171 | H | BP | regulation_of_nitrogen_compound_metabolic_process |
| 0.541 | GO:0008092 | H | MF | cytoskeletal_protein_binding |
| 0.537 | GO:0010468 | H | BP | regulation_of_gene_expression |
| 0.529 | GO:0044822 | H | MF | poly(A)_RNA_binding |
| 0.523 | GO:0009117 | H | BP | nucleotide_metabolic_process |
| 0.523 | GO:0009116 | H | BP | nucleoside_metabolic_process |
| 0.503 | GO:0055114 | H | BP | oxidation-reduction_process |
| 0.963 | GO:0008152 | L | BP | metabolic_process |
| 0.949 | GO:0097159 | L | MF | organic_cyclic_compound_binding |
| 0.924 | GO:0044237 | L | BP | cellular_metabolic_process |
| 0.913 | GO:0006807 | L | BP | nitrogen_compound_metabolic_process |
| 0.897 | GO:0009058 | L | BP | biosynthetic_process |
| 0.896 | GO:0005737 | L | CC | cytoplasm |
| 0.891 | GO:0016787 | L | MF | hydrolase_activity |
| 0.889 | GO:0006139 | L | BP | nucleobase-containing_compound_metabolic_process |
| 0.885 | GO:0043229 | L | CC | intracellular_organelle |
| 0.859 | GO:0050896 | L | BP | response_to_stimulus |
| 0.853 | GO:0043231 | L | CC | intracellular_membrane-bounded_organelle |
| 0.828 | GO:0034641 | L | BP | cellular_nitrogen_compound_metabolic_process |
| 0.825 | GO:0051716 | L | BP | cellular_response_to_stimulus |
| 0.816 | GO:0005634 | L | CC | nucleus |
| 0.813 | GO:0046483 | L | BP | heterocycle_metabolic_process |
| 0.794 | GO:0006725 | L | BP | cellular_aromatic_compound_metabolic_process |
| 0.79 | GO:0046872 | L | MF | metal_ion_binding |
| 0.789 | GO:0019538 | L | BP | protein_metabolic_process |
| 0.785 | GO:0032502 | L | BP | developmental_process |
| 0.783 | GO:0032991 | L | CC | macromolecular_complex |
| 0.781 | GO:0031981 | L | CC | nuclear_lumen |
| 0.774 | GO:0005829 | L | CC | cytosol |
| 0.773 | GO:0043169 | L | MF | cation_binding |
| 0.771 | GO:0036094 | L | MF | small_molecule_binding |
| 0.745 | GO:0044267 | L | BP | cellular_protein_metabolic_process |
| 0.739 | GO:0006464 | L | BP | cellular_protein_modification_process |
| 0.716 | GO:0010467 | L | BP | gene_expression |
| 0.7 | GO:0005654 | L | CC | nucleoplasm |
| 0.669 | GO:0043234 | L | CC | protein_complex |
| 0.663 | GO:0007154 | L | BP | cell_communication |
| 0.657 | GO:0006996 | L | BP | organelle_organization |
| 0.611 | GO:0016070 | L | BP | RNA_metabolic_process |
| 0.602 | GO:0048856 | L | BP | anatomical_structure_development |
| 0.594 | GO:0007275 | L | BP | multicellular_organismal_development |
| 0.547 | GO:0023052 | L | BP | signaling |
| 0.518 | GO:0009893 | L | BP | positive_regulation_of_metabolic_process |

**Protein Name: SSX2**

**Predicted Results:**

**SVMProt**

| **Protein Family Name** | **GO Category** | **SVM** | **PNN** | **KNN** |
| --- | --- | --- | --- | --- |
| **Molecular Function** | | | | |
| Zinc-binding | GO:0008270 zinc binding | 71.3 | - | - |
| Calcium-binding | - | 58.6 | - | - |
| Actin binding | GO:0003779 actin binding | 58.6 | ? | ? |
| **Biological Process** | | | | |
| DNA repair | GO:0006281 DNA repair | 58.6 | ? | ? |
| **Others** | | | | |
| Proto-oncogene | - | 99 | Y | Y |

**FFPred**

| **Score** | **GO term** | **RL** | **Domain** | **Description** |
| --- | --- | --- | --- | --- |
| 0.931 | GO:0019222 | H | BP | regulation of metabolic process |
| 0.908 | GO:0003676 | H | MF | nucleic acid binding |
| 0.893 | GO:0051171 | H | BP | regulation of nitrogen compound metabolic process |
| 0.884 | GO:0010468 | H | BP | regulation of gene expression |
| 0.873 | GO:0006351 | H | BP | transcription, DNA-templated |
| 0.857 | GO:0006355 | H | BP | regulation of transcription, DNA-templated |
| 0.84 | GO:2001141 | H | BP | regulation of RNA biosynthetic process |
| 0.834 | GO:1903506 | H | BP | regulation of nucleic acid-templated transcription |
| 0.832 | GO:0044822 | H | MF | poly(A) RNA binding |
| 0.83 | GO:0051252 | H | BP | regulation of RNA metabolic process |
| 0.824 | GO:0009059 | H | BP | macromolecule biosynthetic process |
| 0.798 | GO:0034645 | H | BP | cellular macromolecule biosynthetic process |
| 0.75 | GO:0008092 | H | MF | cytoskeletal protein binding |
| 0.695 | GO:0006397 | H | BP | mRNA processing |
| 0.682 | GO:0003723 | H | MF | RNA binding |
| 0.68 | GO:0030234 | H | MF | enzyme regulator activity |
| 0.675 | GO:0006810 | H | BP | transport |
| 0.582 | GO:0051641 | H | BP | cellular localization |
| 0.575 | GO:0003779 | H | MF | actin binding |
| 0.571 | GO:0019900 | H | MF | kinase binding |
| 0.568 | GO:0016020 | H | CC | membrane |
| 0.565 | GO:0006396 | H | BP | RNA processing |
| 0.564 | GO:0006357 | H | BP | regulation of transcription from RNA polymerase II promoter |
| 0.512 | GO:0003677 | H | MF | DNA binding |
| 0.501 | GO:0051649 | H | BP | establishment of localization in cell |
| 0.949 | GO:0043229 | L | CC | intracellular organelle |
| 0.932 | GO:0008152 | L | BP | metabolic process |
| 0.919 | GO:0043231 | L | CC | intracellular membrane-bounded organelle |
| 0.895 | GO:0006807 | L | BP | nitrogen compound metabolic process |
| 0.883 | GO:0006139 | L | BP | nucleobase-containing compound metabolic process |
| 0.88 | GO:0032403 | L | MF | protein complex binding |
| 0.874 | GO:0005737 | L | CC | cytoplasm |
| 0.824 | GO:0005634 | L | CC | nucleus |
| 0.806 | GO:0034641 | L | BP | cellular nitrogen compound metabolic process |
| 0.804 | GO:0097159 | L | MF | organic cyclic compound binding |
| 0.797 | GO:0044237 | L | BP | cellular metabolic process |
| 0.797 | GO:0051716 | L | BP | cellular response to stimulus |
| 0.797 | GO:0032991 | L | CC | macromolecular complex |
| 0.794 | GO:0050896 | L | BP | response to stimulus |
| 0.789 | GO:0031981 | L | CC | nuclear lumen |
| 0.78 | GO:0046483 | L | BP | heterocycle metabolic process |
| 0.778 | GO:0007165 | L | BP | signal transduction |
| 0.772 | GO:0005102 | L | MF | receptor binding |
| 0.771 | GO:0023052 | L | BP | signaling |
| 0.765 | GO:0009058 | L | BP | biosynthetic process |
| 0.763 | GO:0006725 | L | BP | cellular aromatic compound metabolic process |
| 0.762 | GO:0048856 | L | BP | anatomical structure development |
| 0.751 | GO:0032502 | L | BP | developmental process |
| 0.736 | GO:0009893 | L | BP | positive regulation of metabolic process |
| 0.728 | GO:0005654 | L | CC | nucleoplasm |
| 0.712 | GO:0007275 | L | BP | multicellular organismal development |
| 0.711 | GO:0005829 | L | CC | cytosol |
| 0.703 | GO:0046872 | L | MF | metal ion binding |
| 0.701 | GO:0007154 | L | BP | cell communication |
| 0.67 | GO:0043234 | L | CC | protein complex |
| 0.639 | GO:0031325 | L | BP | positive regulation of cellular metabolic process |
| 0.639 | GO:0006996 | L | BP | organelle organization |
| 0.634 | GO:0010467 | L | BP | gene expression |
| 0.632 | GO:0016070 | L | BP | RNA metabolic process |
| 0.625 | GO:0030154 | L | BP | cell differentiation |
| 0.611 | GO:0019538 | L | BP | protein metabolic process |
| 0.606 | GO:0043169 | L | MF | cation binding |
| 0.562 | GO:0019904 | L | MF | protein domain specific binding |
| 0.553 | GO:0009966 | L | BP | regulation of signal transduction |
| 0.551 | GO:0008134 | L | MF | transcription factor binding |
| 0.546 | GO:0048513 | L | BP | organ development |
| 0.507 | GO:0051128 | L | BP | regulation of cellular component organization |
| 0.506 | GO:0050790 | L | BP | regulation of catalytic activity |
| 0.503 | GO:0010033 | L | BP | response to organic substance |

**Blast**

| **BLAST Matched UniProt ID & name** | | | **Identity** | **E Value** | **Score** |
| --- | --- | --- | --- | --- | --- |
| Q99909 | SSX3_HUMAN | Protein SSX3 | 90.957 | 1.42E-123 | 353 |
| Q7RTT3 | SSX9_HUMAN | Protein SSX9 | 87.234 | 1.98E-118 | 340 |
| O60225 | SSX5_HUMAN | Protein SSX5 | 84.043 | 1.16E-114 | 330 |
| Q7RTT5 | SSX7_HUMAN | Protein SSX7 | 85.638 | 1.79E-114 | 330 |
| O60224 | SSX4_HUMAN | Protein SSX4 | 79.787 | 1.11E-105 | 308 |
| Q7RTT6 | SSX6_HUMAN | Putative protein SSX6 | 81.283 | 1.42E-105 | 307 |
| Q16384 | SSX1_HUMAN | Protein SSX1 | 78.191 | 1.12E-102 | 300 |
| Q7RTT4 | SSX8_HUMAN | Protein SSX8 | 78.723 | 4.98E-101 | 296 |
| A6NEJ1 | SSX10_HUMAN | Deleted. | 77.193 | 7.08E-56 | 179 |
| P0C6Y7 | PRDM9_RAT | Histone-lysine N-methyltransferase PRDM9 | 32.778 | 1.42E-15 | 78.6 |
| Q9NQW5 | PRDM7_HUMAN | Probable histone-lysine N-methyltransferase PRDM7 | 34.225 | 1.67E-15 | 77.8 |
| Q9NQV7 | PRDM9_HUMAN | Histone-lysine N-methyltransferase PRDM9 | 33.516 | 8.22E-15 | 76.3 |
| Q96EQ9 | PRDM9_MOUSE | Histone-lysine N-methyltransferase PRDM9 | 33.88 | 1.14E-12 | 69.7 |
| Q8TD17 | ZN398_HUMAN | Zinc finger protein 398 | 35.484 | 7.94E-04 | 42.7 |
| Q6IV72 | ZN425_HUMAN | Zinc finger protein 425 | 26.708 | 0.001 | 42.7 |
| Q6ZMS7 | ZN783_HUMAN | Protein ZNF783 | 34.524 | 0.001 | 42 |
| A8K8V0 | ZN785_HUMAN | Zinc finger protein 785 | 34.694 | 0.005 | 40.4 |
| A6NM28 | ZFP92_HUMAN | Zinc finger protein 92 homolog | 30.189 | 0.008 | 39.7 |
| Q9BV97 | ZN747_HUMAN | KRAB domain-containing protein ZNF747 | 34 | 0.009 | 38.9 |
| Q5FWF6 | ZN789_HUMAN | Zinc finger protein 789 | 30.986 | 0.01 | 39.3 |
| Q8N393 | ZN786_HUMAN | Zinc finger protein 786 | 35.556 | 0.011 | 39.7 |
| P51815 | ZN75D_HUMAN | Zinc finger protein 75D | 40 | 0.011 | 39.3 |
| Q9EPU7 | Z354C_RAT | Zinc finger protein 354C | 32.653 | 0.014 | 38.9 |
| Q6AW86 | Z324B_HUMAN | Zinc finger protein 324B | 33.333 | 0.021 | 38.5 |
| Q9ULD5 | ZN777_HUMAN | Zinc finger protein 777 | 29.032 | 0.023 | 38.5 |
| Q571J5 | Z354C_MOUSE | Zinc finger protein 354C | 32.653 | 0.029 | 38.1 |
| Q80TC5 | POGK_MOUSE | Pogo transposable element with KRAB domain | 39.216 | 0.03 | 38.1 |

**Protein Name: HLP1**

**Predicted Results:**

**SVMProt**

| **Protein Family Name** | **GO Category** | **SVM** | **PNN** | **KNN** |
| --- | --- | --- | --- | --- |
| **Molecular Function** | | | | |
| mRNA slicing | - | 73.8 | ? | ? |
| RNA-binding proteins | GO:0003723 RNA binding | 65.4 | ? | ? |
| Zinc-binding | GO:0008270 zinc binding | 58.6 | - | - |
| All DNA-binding | GO:0003677 DNA binding | 58.6 | ? | ? |
| mRNA-binding proteins | GO:0003729 mRNA binding | 58.6 | - | - |
| TC9.A Incompletely Characterized Transport Systems - Recognized transporters of unknown biochemical mechanism | - | 58.6 | ? | ? |
| **Biological Process** | | | | |
| DNA repair | GO:0006281 DNA repair | 58.6 | ? | ? |
| **Broadly Defined Function** | | | | |
| Transmembrane | GO:0016021 integral component of membrane | 58.6 | - | - |

**FFPred**

| **Score** | **GO term** | **RL** | **Domain** | **Description** |
| --- | --- | --- | --- | --- |
| 0.992 | GO:0003676 | H | MF | nucleic acid binding |
| 0.992 | GO:0001228 | H | MF | RNA polymerase II transcription regulatory region sequence-specific DNA binding transcription factor activity involved in positive regulation of transcription |
| 0.988 | GO:0000981 | H | MF | sequence-specific DNA binding RNA polymerase II transcription factor activity |
| 0.986 | GO:0001071 | H | MF | nucleic acid binding transcription factor activity |
| 0.985 | GO:0006351 | H | BP | transcription, DNA-templated |
| 0.979 | GO:0000976 | H | MF | transcription regulatory region sequence-specific DNA binding |
| 0.975 | GO:0000982 | H | MF | RNA polymerase II core promoter proximal region sequence-specific DNA binding transcription factor activity |
| 0.973 | GO:0000977 | H | MF | RNA polymerase II regulatory region sequence-specific DNA binding |
| 0.97 | GO:1903506 | H | BP | regulation of nucleic acid-templated transcription |
| 0.967 | GO:0043565 | H | MF | sequence-specific DNA binding |
| 0.966 | GO:0006355 | H | BP | regulation of transcription, DNA-templated |
| 0.958 | GO:0003677 | H | MF | DNA binding |
| 0.958 | GO:0051252 | H | BP | regulation of RNA metabolic process |
| 0.956 | GO:0010468 | H | BP | regulation of gene expression |
| 0.955 | GO:0000975 | H | MF | regulatory region DNA binding |
| 0.95 | GO:0051171 | H | BP | regulation of nitrogen compound metabolic process |
| 0.936 | GO:2001141 | H | BP | regulation of RNA biosynthetic process |
| 0.928 | GO:0006366 | H | BP | transcription from RNA polymerase II promoter |
| 0.926 | GO:0034645 | H | BP | cellular macromolecule biosynthetic process |
| 0.92 | GO:0009059 | H | BP | macromolecule biosynthetic process |
| 0.903 | GO:0044822 | H | MF | poly(A) RNA binding |
| 0.901 | GO:0001077 | H | MF | RNA polymerase II core promoter proximal region sequence-specific DNA binding transcription factor activity involved in positive regulation of transcription |
| 0.894 | GO:0010557 | H | BP | positive regulation of macromolecule biosynthetic process |
| 0.89 | GO:0003700 | H | MF | sequence-specific DNA binding transcription factor activity |
| 0.871 | GO:0000166 | H | MF | nucleotide binding |
| 0.868 | GO:0003723 | H | MF | RNA binding |
| 0.851 | GO:0000122 | H | BP | negative regulation of transcription from RNA polymerase II promoter |
| 0.845 | GO:0031328 | H | BP | positive regulation of cellular biosynthetic process |
| 0.844 | GO:0045944 | H | BP | positive regulation of transcription from RNA polymerase II promoter |
| 0.82 | GO:0000978 | H | MF | RNA polymerase II core promoter proximal region sequence-specific DNA binding |
| 0.797 | GO:0006357 | H | BP | regulation of transcription from RNA polymerase II promoter |
| 0.791 | GO:0045893 | H | BP | positive regulation of transcription, DNA-templated |
| 0.763 | GO:0019222 | H | BP | regulation of metabolic process |
| 0.763 | GO:0010629 | H | BP | negative regulation of gene expression |
| 0.743 | GO:1903507 | H | BP | negative regulation of nucleic acid-templated transcription |
| 0.734 | GO:0016604 | H | CC | nuclear body |
| 0.734 | GO:0030529 | H | CC | ribonucleoprotein complex |
| 0.732 | GO:0001012 | H | MF | RNA polymerase II regulatory region DNA binding |
| 0.731 | GO:0010628 | H | BP | positive regulation of gene expression |
| 0.716 | GO:0001159 | H | MF | core promoter proximal region DNA binding |
| 0.699 | GO:0000987 | H | MF | core promoter proximal region sequence-specific DNA binding |
| 0.673 | GO:0045892 | H | BP | negative regulation of transcription, DNA-templated |
| 0.654 | GO:0009890 | H | BP | negative regulation of biosynthetic process |
| 0.615 | GO:0008380 | H | BP | RNA splicing |
| 0.612 | GO:0044212 | H | MF | transcription regulatory region DNA binding |
| 0.584 | GO:0016071 | H | BP | mRNA metabolic process |
| 0.583 | GO:0006396 | H | BP | RNA processing |
| 0.579 | GO:0005730 | H | CC | nucleolus |
| 0.982 | GO:0008152 | L | BP | metabolic process |
| 0.973 | GO:0006807 | L | BP | nitrogen compound metabolic process |
| 0.97 | GO:0097159 | L | MF | organic cyclic compound binding |
| 0.969 | GO:0006139 | L | BP | nucleobase-containing compound metabolic process |
| 0.968 | GO:0043229 | L | CC | intracellular organelle |
| 0.956 | GO:0044237 | L | BP | cellular metabolic process |
| 0.952 | GO:0009058 | L | BP | biosynthetic process |
| 0.942 | GO:0043231 | L | CC | intracellular membrane-bounded organelle |
| 0.881 | GO:0005634 | L | CC | nucleus |
| 0.878 | GO:0036094 | L | MF | small molecule binding |
| 0.866 | GO:0034641 | L | BP | cellular nitrogen compound metabolic process |
| 0.865 | GO:0046483 | L | BP | heterocycle metabolic process |
| 0.859 | GO:0031981 | L | CC | nuclear lumen |
| 0.855 | GO:0006725 | L | BP | cellular aromatic compound metabolic process |
| 0.844 | GO:0016070 | L | BP | RNA metabolic process |
| 0.836 | GO:0010467 | L | BP | gene expression |
| 0.815 | GO:0005654 | L | CC | nucleoplasm |
| 0.811 | GO:0032502 | L | BP | developmental process |
| 0.794 | GO:0032991 | L | CC | macromolecular complex |
| 0.793 | GO:0048856 | L | BP | anatomical structure development |
| 0.792 | GO:0008134 | L | MF | transcription factor binding |
| 0.785 | GO:0051716 | L | BP | cellular response to stimulus |
| 0.777 | GO:0050896 | L | BP | response to stimulus |
| 0.757 | GO:0009893 | L | BP | positive regulation of metabolic process |
| 0.74 | GO:0007275 | L | BP | multicellular organismal development |
| 0.73 | GO:0005737 | L | CC | cytoplasm |
| 0.724 | GO:0007154 | L | BP | cell communication |
| 0.716 | GO:0023052 | L | BP | signaling |
| 0.71 | GO:0031324 | L | BP | negative regulation of cellular metabolic process |
| 0.689 | GO:0030154 | L | BP | cell differentiation |
| 0.688 | GO:0005829 | L | CC | cytosol |
| 0.679 | GO:0031325 | L | BP | positive regulation of cellular metabolic process |
| 0.65 | GO:0006996 | L | BP | organelle organization |
| 0.643 | GO:0048513 | L | BP | organ development |
| 0.638 | GO:0007165 | L | BP | signal transduction |
| 0.597 | GO:0019904 | L | MF | protein domain specific binding |
| 0.592 | GO:0043234 | L | CC | protein complex |
| 0.551 | GO:0009653 | L | BP | anatomical structure morphogenesis |
| 0.538 | GO:0000790 | L | CC | nuclear chromatin |
| 0.524 | GO:0043566 | L | MF | structure-specific DNA binding |
| 0.518 | GO:0010033 | L | BP | response to organic substance |
| 0.511 | GO:0051246 | L | BP | regulation of protein metabolic process |

**Blast**

| **BLAST Matched UniProt ID & name** | | | **Identity** | **E Value** | **Score** |
| --- | --- | --- | --- | --- | --- |
| Q8W034 | RNP1_ARATH | Heterogeneous nuclear ribonucleoprotein 1 | 43.316 | 3.52E-47 | 172 |
| Q8W034 | RNP1_ARATH | Heterogeneous nuclear ribonucleoprotein 1 | 45.349 | 7.59E-16 | 82.4 |
| P48809 | RB27C_DROME | Heterogeneous nuclear ribonucleoprotein 27C | 41.86 | 2.47E-40 | 153 |
| Q99383 | HRP1_YEAST | Nuclear polyadenylated RNA-binding protein 4 | 47.337 | 2.63E-40 | 155 |
| Q99383 | HRP1_YEAST | Nuclear polyadenylated RNA-binding protein 4 | 46.341 | 2.50E-14 | 78.2 |
| Q920Q6 | MSI2H_MOUSE | RNA-binding protein Musashi homolog 2 | 40.884 | 3.90E-39 | 148 |
| Q96DH6 | MSI2H_HUMAN | RNA-binding protein Musashi homolog 2 | 41.081 | 4.55E-39 | 148 |
| O94432 | YHKF_SCHPO | Uncharacterized RNA-binding protein C660.15 | 40.212 | 5.13E-39 | 150 |
| Q9VVE5 | MSIR6_DROME | RNA-binding protein Musashi homolog Rbp6 | 41.989 | 2.10E-37 | 144 |
| Q9C652 | RBP1_ARATH | RNA-binding protein 1 | 38.144 | 2.33E-37 | 144 |
| Q9C652 | RBP1_ARATH | RNA-binding protein 1 | 46.809 | 5.74E-17 | 85.1 |
| Q9C652 | RBP1_ARATH | RNA-binding protein 1 | 30.864 | 3.40E-04 | 45.8 |
| O43347 | MSI1H_HUMAN | RNA-binding protein Musashi homolog 1 | 38.86 | 1.09E-36 | 142 |
| Q61474 | MSI1H_MOUSE | RNA-binding protein Musashi homolog 1 | 38.86 | 2.09E-36 | 141 |
| Q8K3P4 | MSI1H_RAT | RNA-binding protein Musashi homolog 1 | 38.86 | 2.15E-36 | 141 |
| Q96EP5 | DAZP1_HUMAN | DAZ-associated protein 1 | 38.22 | 5.02E-35 | 138 |
| Q9JII5 | DAZP1_MOUSE | DAZ-associated protein 1 | 38.22 | 7.08E-35 | 137 |
| Q02926 | RB97D_DROME | Ribonucleoprotein RB97D | 36.318 | 1.25E-33 | 135 |
| Q98SJ2 | DAZP1_XENLA | DAZ-associated protein 1 | 33.984 | 9.61E-33 | 130 |
| Q32P51 | RA1L2_HUMAN | Heterogeneous nuclear ribonucleoprotein A1-like 2 | 35.87 | 2.16E-31 | 126 |
| Q32P51 | RA1L2_HUMAN | Heterogeneous nuclear ribonucleoprotein A1-like 2 | 34.694 | 2.61E-08 | 58.5 |
| Q99020 | ROAA_MOUSE | Heterogeneous nuclear ribonucleoprotein A/B | 37.017 | 2.67E-30 | 122 |
| Q99020 | ROAA_MOUSE | Heterogeneous nuclear ribonucleoprotein A/B | 37.647 | 3.03E-06 | 52 |
| P04256 | ROA1_RAT | Heterogeneous nuclear ribonucleoprotein A1 | 35.87 | 3.64E-30 | 122 |
| P04256 | ROA1_RAT | Heterogeneous nuclear ribonucleoprotein A1 | 35.354 | 8.96E-09 | 60.1 |
| P49312 | ROA1_MOUSE | Heterogeneous nuclear ribonucleoprotein A1 | 35.87 | 3.71E-30 | 122 |
| P49312 | ROA1_MOUSE | Heterogeneous nuclear ribonucleoprotein A1 | 35.354 | 8.80E-09 | 60.1 |
| Q99729 | ROAA_HUMAN | Heterogeneous nuclear ribonucleoprotein A/B | 35.714 | 1.27E-29 | 121 |
| Q28521 | ROA1_MACMU | Heterogeneous nuclear ribonucleoprotein A1 | 35.326 | 1.70E-29 | 120 |
| Q28521 | ROA1_MACMU | Heterogeneous nuclear ribonucleoprotein A1 | 35.354 | 7.42E-09 | 60.5 |
| Q640A2 | HNRDL_XENTR | Heterogeneous nuclear ribonucleoprotein D-like | 34.973 | 1.31E-26 | 112 |
| Q5ZI72 | HNRDL_CHICK | Heterogeneous nuclear ribonucleoprotein D-like | 33.88 | 4.90E-26 | 110 |
| Q3SWU3 | HNRDL_RAT | Heterogeneous nuclear ribonucleoprotein D-like | 35.673 | 4.99E-26 | 110 |
| Q3SWU3 | HNRDL_RAT | Heterogeneous nuclear ribonucleoprotein D-like | 34.409 | 1.67E-09 | 62.4 |
| Q7ZX83 | HNDLA_XENLA | Heterogeneous nuclear ribonucleoprotein D-like-A | 35.519 | 5.70E-26 | 110 |
| Q9Z130 | HNRDL_MOUSE | Heterogeneous nuclear ribonucleoprotein D-like | 33.166 | 8.63E-26 | 109 |
| O14979 | HNRDL_HUMAN | Heterogeneous nuclear ribonucleoprotein D-like | 34.031 | 1.47E-25 | 110 |
| Q60668 | HNRPD_MOUSE | Heterogeneous nuclear ribonucleoprotein D0 | 32.948 | 3.24E-24 | 105 |
| Q14103 | HNRPD_HUMAN | Heterogeneous nuclear ribonucleoprotein D0 | 34.969 | 3.60E-24 | 105 |
| Q9JJ54 | HNRPD_RAT | Heterogeneous nuclear ribonucleoprotein D0 | 34.356 | 4.02E-24 | 105 |
| Q6NU14 | HNDLB_XENLA | Heterogeneous nuclear ribonucleoprotein D-like-B | 36.257 | 7.55E-24 | 103 |
| Q6NU14 | HNDLB_XENLA | Heterogeneous nuclear ribonucleoprotein D-like-B | 37.634 | 3.12E-09 | 61.2 |
| Q08473 | SQD_DROME | RNA-binding protein squid | 32.627 | 1.32E-23 | 103 |
| Q13148 | TADBP_HUMAN | TAR DNA-binding protein 43 | 29.255 | 2.48E-20 | 95.5 |
| Q5R5W2 | TADBP_PONAB | TAR DNA-binding protein 43 | 29.255 | 2.53E-20 | 95.5 |
| Q5ZLN5 | TADBP_CHICK | TAR DNA-binding protein 43 | 30.851 | 5.12E-20 | 94.7 |
| Q5ZLN5 | TADBP_CHICK | TAR DNA-binding protein 43 | 29.167 | 1.87E-05 | 50.1 |
| Q28F51 | TADBP_XENTR | TAR DNA-binding protein 43 | 29.843 | 7.09E-20 | 94.4 |
| Q921F2 | TADBP_MOUSE | TAR DNA-binding protein 43 | 29.787 | 4.22E-18 | 89 |
| P51990 | RO22_XENLA | Heterogeneous nuclear ribonucleoprotein A2 homolog 2 | 29.502 | 4.84E-18 | 88.2 |
| P51990 | RO22_XENLA | Heterogeneous nuclear ribonucleoprotein A2 homolog 2 | 31.034 | 6.33E-08 | 57.8 |
| P28644 | ROC1_SPIOL | 28 kDa ribonucleoprotein, chloroplastic | 31.034 | 6.82E-18 | 85.9 |
| O80678 | UBA2B_ARATH | UBP1-associated protein 2B | 27.035 | 1.43E-13 | 75.5 |
| O80678 | UBA2B_ARATH | UBP1-associated protein 2B | 23.967 | 3.50E-04 | 46.2 |
| Q9LKA4 | UBA2C_ARATH | UBP1-associated protein 2C | 24.201 | 1.78E-13 | 75.1 |
| Q9LKA4 | UBA2C_ARATH | UBP1-associated protein 2C | 21.875 | 1.33E-04 | 47.4 |
| P19683 | ROC4_NICSY | 31 kDa ribonucleoprotein, chloroplastic | 29.651 | 2.25E-13 | 73.9 |
| Q08935 | ROC1_NICSY | 29 kDa ribonucleoprotein A, chloroplastic | 27.941 | 4.48E-13 | 72.4 |
| P49313 | ROC1_NICPL | 30 kDa ribonucleoprotein, chloroplastic | 27.128 | 2.01E-12 | 70.5 |
| Q8RWN5 | RZ1C_ARATH | Glycine-rich RNA-binding protein RZ1C | 45.161 | 8.55E-12 | 68.9 |
| Q8RWN5 | RZ1C_ARATH | Glycine-rich RNA-binding protein RZ1C | 35.443 | 3.38E-10 | 64.3 |
| Q22037 | ROA1_CAEEL | Heterogeneous nuclear ribonucleoprotein A1 | 45 | 1.28E-11 | 68.9 |
| Q22037 | ROA1_CAEEL | Heterogeneous nuclear ribonucleoprotein A1 | 34.694 | 7.35E-11 | 66.6 |
| P17130 | ROA1_XENLA | Heterogeneous nuclear ribonucleoproteins A1 homolog | 39.08 | 1.24E-09 | 63.2 |
| P10979 | GRPA_MAIZE | Glycine-rich RNA-binding, abscisic acid-inducible protein | 43.077 | 3.51E-09 | 59.3 |
| Q05966 | GRP10_BRANA | Glycine-rich RNA-binding protein 10 | 54 | 6.03E-09 | 58.5 |
| Q03878 | GRP1_DAUCA | Glycine-rich RNA-binding protein | 45.763 | 6.63E-09 | 58.5 |
| Q9FNR1 | RBG3_ARATH | Glycine-rich RNA-binding protein 3, mitochondrial | 41.935 | 6.87E-09 | 60.5 |
| Q99070 | GRP2_SORBI | Glycine-rich RNA-binding protein 2 | 38.462 | 2.30E-08 | 57 |
| P49310 | GRP1_SINAL | Glycine-rich RNA-binding protein GRP1A | 45.763 | 3.05E-08 | 56.6 |
| P09651 | ROA1_HUMAN | Heterogeneous nuclear ribonucleoprotein A1 | 32.5 | 3.18E-08 | 58.5 |
| Q03250 | RBG7_ARATH | Glycine-rich RNA-binding protein 7 | 44.068 | 3.81E-08 | 56.2 |
| Q43472 | GRP_HORVU | Glycine-rich RNA-binding protein blt801 | 44.068 | 5.77E-08 | 55.5 |
| P49311 | GRP2_SINAL | Glycine-rich RNA-binding protein GRP2A | 44.068 | 6.87E-08 | 55.5 |
| Q03251 | RBG8_ARATH | Glycine-rich RNA-binding protein 8 | 40 | 8.25E-08 | 55.1 |
| P22626 | ROA2_HUMAN | Heterogeneous nuclear ribonucleoproteins A2/B1 | 31.818 | 9.38E-08 | 57 |
| O88569 | ROA2_MOUSE | Heterogeneous nuclear ribonucleoproteins A2/B1 | 31.818 | 9.38E-08 | 57 |
| P48810 | RB87F_DROME | Heterogeneous nuclear ribonucleoprotein 87F | 36.364 | 9.46E-08 | 57.4 |
| A7VJC2 | ROA2_RAT | Heterogeneous nuclear ribonucleoproteins A2/B1 | 31.818 | 9.72E-08 | 57 |
| Q5RBU8 | ROA2_PONAB | Heterogeneous nuclear ribonucleoproteins A2/B1 | 31.818 | 1.12E-07 | 57 |
| Q9TTV2 | ROA2_SAGOE | Heterogeneous nuclear ribonucleoproteins A2/B1 | 32.692 | 1.58E-07 | 56.2 |
| Q9C909 | RBG5_ARATH | Glycine-rich RNA-binding protein 5, mitochondrial | 37.333 | 2.00E-07 | 55.8 |
| Q2HJ60 | ROA2_BOVIN | Heterogeneous nuclear ribonucleoproteins A2/B1 | 32.692 | 2.25E-07 | 55.8 |
| P51989 | RO21_XENLA | Heterogeneous nuclear ribonucleoprotein A2 homolog 1 | 34.375 | 3.51E-07 | 55.5 |
| Q9SIX3 | RBG1_ARATH | Probable glycine-rich RNA-binding protein 1 | 38.462 | 5.15E-07 | 52.4 |
| P07909 | ROA1_DROME | Heterogeneous nuclear ribonucleoprotein A1 | 33.333 | 8.08E-07 | 54.3 |
| Q43349 | CP29A_ARATH | 29 kDa ribonucleoprotein, chloroplastic | 33.333 | 2.74E-06 | 52.4 |
| Q43349 | CP29A_ARATH | 29 kDa ribonucleoprotein, chloroplastic | 33.333 | 0.001 | 44.3 |
| Q9SVM8 | RBG2_ARATH | Glycine-rich RNA-binding protein 2, mitochondrial | 42 | 1.08E-05 | 48.5 |
| Q9SVM8 | RBG2_ARATH | Glycine-rich RNA-binding protein 2, mitochondrial | 30.075 | 0.032 | 38.5 |
| Q99069 | GRP1_SORBI | Glycine-rich RNA-binding protein 1 | 42.5 | 0.001 | 42.7 |
| P51968 | RO31_XENLA | Heterogeneous nuclear ribonucleoprotein A3 homolog 1 | 25.221 | 0.008 | 41.6 |
| P21522 | ROA1_SCHAM | Heterogeneous nuclear ribonucleoprotein A1, A2/B1 homolog | 27.778 | 0.049 | 39.3 |

**Protein Name: LIX1L**

**Predicted results**

**SVMProt**

| **Protein Family Name** | **GO Category** | **SVM** | **PNN** | **KNN** |
| --- | --- | --- | --- | --- |
| **Molecular Function** | | | | |
| Zinc-binding | GO:0008270 zinc binding | 97.3 | - | - |
| All lipid-binding proteins | GO:0008289 lipid binding | 90.3 | - | - |
| All DNA-binding | GO:0003677 DNA binding | 58.6 | ? | ? |
| Metal-binding | GO:0046872 metal ion binding | 58.6 | - | - |
| Copper-binding | GO:0005507 copper binding | 58.6 | - | - |
| **Biological Process** | | | | |
| Chlorophyll biosynthesis | GO:0015995 chlorophyll biosynthetic process | 58.6 | ? | ? |

**FFPred**

| **Score** | **GO term** | **RL** | **Domain** | **Description** |
| --- | --- | --- | --- | --- |
| 0.933 | GO:0003824 | H | MF | catalytic activity |
| 0.859 | GO:0019222 | H | BP | regulation of metabolic process |
| 0.847 | GO:0010468 | H | BP | regulation of gene expression |
| 0.796 | GO:0030234 | H | MF | enzyme regulator activity |
| 0.778 | GO:0006351 | H | BP | transcription, DNA-templated |
| 0.776 | GO:0009059 | H | BP | macromolecule biosynthetic process |
| 0.764 | GO:0016740 | H | MF | transferase activity |
| 0.756 | GO:0005740 | H | CC | mitochondrial envelope |
| 0.744 | GO:0051171 | H | BP | regulation of nitrogen compound metabolic process |
| 0.736 | GO:0003676 | H | MF | nucleic acid binding |
| 0.718 | GO:0019901 | H | MF | protein kinase binding |
| 0.709 | GO:0003677 | H | MF | DNA binding |
| 0.689 | GO:0019900 | H | MF | kinase binding |
| 0.673 | GO:0051252 | H | BP | regulation of RNA metabolic process |
| 0.67 | GO:0005739 | H | CC | mitochondrion |
| 0.65 | GO:0006355 | H | BP | regulation of transcription, DNA-templated |
| 0.649 | GO:0034645 | H | BP | cellular macromolecule biosynthetic process |
| 0.644 | GO:2001141 | H | BP | regulation of RNA biosynthetic process |
| 0.639 | GO:0009056 | H | BP | catabolic process |
| 0.636 | GO:0000166 | H | MF | nucleotide binding |
| 0.631 | GO:1903506 | H | BP | regulation of nucleic acid-templated transcription |
| 0.628 | GO:0015631 | H | MF | tubulin binding |
| 0.627 | GO:0046982 | H | MF | protein heterodimerization activity |
| 0.588 | GO:0006796 | H | BP | phosphate-containing compound metabolic process |
| 0.586 | GO:0035556 | H | BP | intracellular signal transduction |
| 0.558 | GO:0031966 | H | CC | mitochondrial membrane |
| 0.546 | GO:0005516 | H | MF | calmodulin binding |
| 0.545 | GO:0016788 | H | MF | hydrolase activity, acting on ester bonds |
| 0.533 | GO:0005524 | H | MF | ATP binding |
| 0.518 | GO:0046914 | H | MF | transition metal ion binding |
| 0.508 | GO:0031090 | H | CC | organelle membrane |
| 0.979 | GO:0043229 | L | CC | intracellular organelle |
| 0.956 | GO:0043231 | L | CC | intracellular membrane-bounded organelle |
| 0.926 | GO:0044237 | L | BP | cellular metabolic process |
| 0.911 | GO:0008152 | L | BP | metabolic process |
| 0.905 | GO:0006139 | L | BP | nucleobase-containing compound metabolic process |
| 0.889 | GO:0005737 | L | CC | cytoplasm |
| 0.859 | GO:0016787 | L | MF | hydrolase activity |
| 0.847 | GO:0009058 | L | BP | biosynthetic process |
| 0.846 | GO:0006807 | L | BP | nitrogen compound metabolic process |
| 0.844 | GO:0005634 | L | CC | nucleus |
| 0.821 | GO:0050896 | L | BP | response to stimulus |
| 0.815 | GO:0046483 | L | BP | heterocycle metabolic process |
| 0.81 | GO:0034641 | L | BP | cellular nitrogen compound metabolic process |
| 0.801 | GO:0051716 | L | BP | cellular response to stimulus |
| 0.793 | GO:0019538 | L | BP | protein metabolic process |
| 0.791 | GO:0006725 | L | BP | cellular aromatic compound metabolic process |
| 0.785 | GO:0006996 | L | BP | organelle organization |
| 0.768 | GO:0031981 | L | CC | nuclear lumen |
| 0.755 | GO:0007165 | L | BP | signal transduction |
| 0.746 | GO:0097159 | L | MF | organic cyclic compound binding |
| 0.739 | GO:0023052 | L | BP | signaling |
| 0.73 | GO:0007154 | L | BP | cell communication |
| 0.721 | GO:0016070 | L | BP | RNA metabolic process |
| 0.718 | GO:0005654 | L | CC | nucleoplasm |
| 0.717 | GO:0050790 | L | BP | regulation of catalytic activity |
| 0.71 | GO:0009893 | L | BP | positive regulation of metabolic process |
| 0.708 | GO:0032502 | L | BP | developmental process |
| 0.706 | GO:0009966 | L | BP | regulation of signal transduction |
| 0.699 | GO:0043169 | L | MF | cation binding |
| 0.692 | GO:0005829 | L | CC | cytosol |
| 0.686 | GO:0007275 | L | BP | multicellular organismal development |
| 0.684 | GO:0010467 | L | BP | gene expression |
| 0.678 | GO:0044267 | L | BP | cellular protein metabolic process |
| 0.666 | GO:0032991 | L | CC | macromolecular complex |
| 0.664 | GO:0006464 | L | BP | cellular protein modification process |
| 0.648 | GO:0008134 | L | MF | transcription factor binding |
| 0.64 | GO:0048856 | L | BP | anatomical structure development |
| 0.62 | GO:0046872 | L | MF | metal ion binding |
| 0.611 | GO:0019904 | L | MF | protein domain specific binding |
| 0.597 | GO:0043234 | L | CC | protein complex |
| 0.589 | GO:0042325 | L | BP | regulation of phosphorylation |
| 0.569 | GO:0031325 | L | BP | positive regulation of cellular metabolic process |
| 0.564 | GO:0036094 | L | MF | small molecule binding |
| 0.541 | GO:0032403 | L | MF | protein complex binding |

**Blast**

| **BLAST Matched UniProt ID & name** | | | **Identity** | **E Value** | **Score** |
| --- | --- | --- | --- | --- | --- |
| Q8BQ89 | LIX1L_MOUSE | LIX1-like protein | 96.736 | 0 | 672 |
| Q5PQQ7 | LIX1L_RAT | LIX1-like protein | 96.746 | 0 | 671 |
| Q6P566 | LIX1_MOUSE | Protein limb expression 1 homolog | 61.277 | 8.26E-104 | 313 |
| Q8N485 | LIX1_HUMAN | Protein limb expression 1 homolog | 60.426 | 4.14E-102 | 308 |
| Q8UVV7 | LIX1_CHICK | Protein limb expression 1 | 60.851 | 4.87E-102 | 308 |

**Protein Name:** **P311**

**Predicted results:**

**SVMProt**

| **Protein Family Name** | **GO Category** | **SVM** | **PNN** | **KNN** |
| --- | --- | --- | --- | --- |
| **Molecular Function** | | | | |
| Metal-binding | GO:0046872 metal ion binding | 58.6 | - | - |
| EC2.1 Transferases - Transferring One-Carbon Groups | - | 58.6 | - | - |
| EC3.1 Hydrolases - Acting on Ester Bonds | - | 58.6 | - | - |
| All lipid-binding proteins | GO:0008289 lipid binding | 58.6 | - | - |
| Calcium-binding | - | 58.6 | - | - |
| **Biological Process** | | | | |
| DNA repair | GO:0006281 DNA repair | 58.6 | ? | ? |
| **Broadly Defined Function** | | | | |
| Structural proteins (Matrix protein,Core protein,Viral occlusion body,Keratin) | - | 62.2 | ? | ? |

**FFPred**

| **Score** | **GO term** | **RL** | **Domain** | **Description** |
| --- | --- | --- | --- | --- |
| 0.976 | GO:0003676 | H | MF | nucleic acid binding |
| 0.976 | GO:0019222 | H | BP | regulation of metabolic process |
| 0.953 | GO:0003677 | H | MF | DNA binding |
| 0.94 | GO:0010468 | H | BP | regulation of gene expression |
| 0.927 | GO:2001141 | H | BP | regulation of RNA biosynthetic process |
| 0.917 | GO:0031224 | H | CC | intrinsic component of membrane |
| 0.915 | GO:0016020 | H | CC | membrane |
| 0.912 | GO:0051252 | H | BP | regulation of RNA metabolic process |
| 0.905 | GO:0034645 | H | BP | cellular macromolecule biosynthetic process |
| 0.898 | GO:0051171 | H | BP | regulation of nitrogen compound metabolic process |
| 0.881 | GO:1903506 | H | BP | regulation of nucleic acid-templated transcription |
| 0.865 | GO:0006355 | H | BP | regulation of transcription, DNA-templated |
| 0.847 | GO:0003700 | H | MF | sequence-specific DNA binding transcription factor activity |
| 0.802 | GO:0003824 | H | MF | catalytic activity |
| 0.751 | GO:0008092 | H | MF | cytoskeletal protein binding |
| 0.739 | GO:0006810 | H | BP | transport |
| 0.735 | GO:0009059 | H | BP | macromolecule biosynthetic process |
| 0.715 | GO:0003723 | H | MF | RNA binding |
| 0.705 | GO:0006357 | H | BP | regulation of transcription from RNA polymerase II promoter |
| 0.694 | GO:0001071 | H | MF | nucleic acid binding transcription factor activity |
| 0.694 | GO:0006468 | H | BP | protein phosphorylation |
| 0.682 | GO:0005739 | H | CC | mitochondrion |
| 0.679 | GO:0007166 | H | BP | cell surface receptor signaling pathway |
| 0.671 | GO:0071944 | H | CC | cell periphery |
| 0.649 | GO:0005886 | H | CC | plasma membrane |
| 0.639 | GO:0016021 | H | CC | integral component of membrane |
| 0.586 | GO:0016773 | H | MF | phosphotransferase activity, alcohol group as acceptor |
| 0.585 | GO:0006351 | H | BP | transcription, DNA-templated |
| 0.585 | GO:0010629 | H | BP | negative regulation of gene expression |
| 0.582 | GO:0051641 | H | BP | cellular localization |
| 0.57 | GO:1903507 | H | BP | negative regulation of nucleic acid-templated transcription |
| 0.562 | GO:0010628 | H | BP | positive regulation of gene expression |
| 0.551 | GO:0005730 | H | CC | nucleolus |
| 0.539 | GO:0003779 | H | MF | actin binding |
| 0.539 | GO:0051649 | H | BP | establishment of localization in cell |
| 0.528 | GO:0016071 | H | BP | mRNA metabolic process |
| 0.522 | GO:0098588 | H | CC | bounding membrane of organelle |
| 0.51 | GO:0016740 | H | MF | transferase activity |
| 0.508 | GO:0005198 | H | MF | structural molecule activity |
| 0.949 | GO:0043229 | L | CC | intracellular organelle |
| 0.946 | GO:0005737 | L | CC | cytoplasm |
| 0.941 | GO:0044237 | L | BP | cellular metabolic process |
| 0.937 | GO:0008152 | L | BP | metabolic process |
| 0.918 | GO:0097159 | L | MF | organic cyclic compound binding |
| 0.904 | GO:0007154 | L | BP | cell communication |
| 0.891 | GO:0043231 | L | CC | intracellular membrane-bounded organelle |
| 0.867 | GO:0006807 | L | BP | nitrogen compound metabolic process |
| 0.867 | GO:0009058 | L | BP | biosynthetic process |
| 0.857 | GO:0032991 | L | CC | macromolecular complex |
| 0.855 | GO:0006139 | L | BP | nucleobase-containing compound metabolic process |
| 0.833 | GO:0023052 | L | BP | signaling |
| 0.833 | GO:0005102 | L | MF | receptor binding |
| 0.816 | GO:0005634 | L | CC | nucleus |
| 0.814 | GO:0034641 | L | BP | cellular nitrogen compound metabolic process |
| 0.813 | GO:0007165 | L | BP | signal transduction |
| 0.807 | GO:0016772 | L | MF | transferase activity, transferring phosphorus-containing groups |
| 0.799 | GO:0050896 | L | BP | response to stimulus |
| 0.798 | GO:0046483 | L | BP | heterocycle metabolic process |
| 0.793 | GO:0051716 | L | BP | cellular response to stimulus |
| 0.781 | GO:0006725 | L | BP | cellular aromatic compound metabolic process |
| 0.781 | GO:0007275 | L | BP | multicellular organismal development |
| 0.78 | GO:0031981 | L | CC | nuclear lumen |
| 0.779 | GO:0032502 | L | BP | developmental process |
| 0.774 | GO:0048856 | L | BP | anatomical structure development |
| 0.756 | GO:0009893 | L | BP | positive regulation of metabolic process |
| 0.754 | GO:0032403 | L | MF | protein complex binding |
| 0.729 | GO:0005654 | L | CC | nucleoplasm |
| 0.707 | GO:0031325 | L | BP | positive regulation of cellular metabolic process |
| 0.677 | GO:0010467 | L | BP | gene expression |
| 0.665 | GO:0043234 | L | CC | protein complex |
| 0.659 | GO:0036094 | L | MF | small molecule binding |
| 0.657 | GO:0005875 | L | CC | microtubule associated complex |
| 0.653 | GO:0006996 | L | BP | organelle organization |
| 0.652 | GO:0005829 | L | CC | cytosol |
| 0.648 | GO:0030154 | L | BP | cell differentiation |
| 0.624 | GO:0016070 | L | BP | RNA metabolic process |
| 0.599 | GO:0048513 | L | BP | organ development |
| 0.597 | GO:0008134 | L | MF | transcription factor binding |
| 0.596 | GO:0009966 | L | BP | regulation of signal transduction |
| 0.594 | GO:0019538 | L | BP | protein metabolic process |
| 0.569 | GO:0019904 | L | MF | protein domain specific binding |
| 0.512 | GO:0043169 | L | MF | cation binding |
| 0.502 | GO:0010033 | L | BP | response to organic substance |

**Blast**

| **BLAST Matched UniProt ID & name** | | | **Identity** | **E Value** | **Score** |
| --- | --- | --- | --- | --- | --- |
| Q4R541 | NREP_MACFA | Neuronal regeneration-related protein | 97.059 | 4.55E-42 | 137 |
| Q5NVD3 | NREP_PONAB | Neuronal regeneration-related protein | 97.059 | 1.47E-41 | 135 |
| Q07475 | NREP_MOUSE | Neuronal regeneration-related protein | 76.471 | 1.29E-28 | 102 |
| Q80Z34 | NREP_RAT | Neuronal regeneration-related protein | 75 | 2.84E-27 | 99 |
| Q90667 | NREP_CHICK | Neuronal regeneration-related protein | 41.176 | 1.75E-12 | 60.8 |

**Protein Name: MdSHT–KP698511**

**Predicted results:**

**SVMProt**

| **Protein Family Name** | **GO Category** | **SVM** | **PNN** | **KNN** |
| --- | --- | --- | --- | --- |
| **Molecular Function** | | | | |
| EC6.3 Ligases - Forming Carbon-Nitrogen Bonds | - | 89.3 | - | Y |
| Zinc-binding | GO:0008270 zinc binding | 95.2 | - | - |
| EC4.1 Lyases - Carbon-Carbon Lyases | - | 82.2 | - | - |
| Metal-binding | GO:0046872 metal ion binding | 80.4 | - | - |
| EC3.2 Hydrolases - Glycosylases | - | 76.2 | - | - |
| EC4.6 Lyases - Phosphorus-Oxygen Lyases | - | 58.6 | ? | ? |
| Lectin | GO:0030246 carbohydrate binding | 58.6 | ? | ? |
| **Broadly Defined Function** | | | | |
| Outer membrane | GO:0009279 cell outer membrane | 58.6 | - | - |
| Photosystem I | GO:0009522 photosystem I | 58.6 | ? | ? |

**FFPred**

| **Score** | **GO term** | **RL** | **Domain** | **Description** |
| --- | --- | --- | --- | --- |
| 0.936 | GO:0016740 | H | MF | transferase activity |
| 0.888 | GO:0003824 | H | MF | catalytic activity |
| 0.849 | GO:0017076 | H | MF | purine nucleotide binding |
| 0.828 | GO:0004672 | H | MF | protein kinase activity |
| 0.824 | GO:0004872 | H | MF | receptor activity |
| 0.801 | GO:0032549 | H | MF | ribonucleoside binding |
| 0.793 | GO:0019222 | H | BP | regulation of metabolic process |
| 0.793 | GO:0005739 | H | CC | mitochondrion |
| 0.789 | GO:0016020 | H | CC | membrane |
| 0.788 | GO:0035639 | H | MF | purine ribonucleoside triphosphate binding |
| 0.779 | GO:0006796 | H | BP | phosphate-containing compound metabolic process |
| 0.778 | GO:0016301 | H | MF | kinase activity |
| 0.742 | GO:0016773 | H | MF | phosphotransferase activity, alcohol group as acceptor |
| 0.738 | GO:0016021 | H | CC | integral component of membrane |
| 0.716 | GO:0000166 | H | MF | nucleotide binding |
| 0.705 | GO:0001882 | H | MF | nucleoside binding |
| 0.703 | GO:0005524 | H | MF | ATP binding |
| 0.692 | GO:0044281 | H | BP | small molecule metabolic process |
| 0.689 | GO:0006810 | H | BP | transport |
| 0.686 | GO:0071944 | H | CC | cell periphery |
| 0.683 | GO:0001883 | H | MF | purine nucleoside binding |
| 0.666 | GO:0031966 | H | CC | mitochondrial membrane |
| 0.642 | GO:0012505 | H | CC | endomembrane system |
| 0.636 | GO:0007166 | H | BP | cell surface receptor signaling pathway |
| 0.615 | GO:0009056 | H | BP | catabolic process |
| 0.613 | GO:0005886 | H | CC | plasma membrane |
| 0.609 | GO:0016874 | H | MF | ligase activity |
| 0.597 | GO:0005789 | H | CC | endoplasmic reticulum membrane |
| 0.581 | GO:0004871 | H | MF | signal transducer activity |
| 0.57 | GO:0005783 | H | CC | endoplasmic reticulum |
| 0.563 | GO:0051171 | H | BP | regulation of nitrogen compound metabolic process |
| 0.557 | GO:0005576 | H | CC | extracellular region |
| 0.55 | GO:0038023 | H | MF | signaling receptor activity |
| 0.542 | GO:0006955 | H | BP | immune response |
| 0.538 | GO:0030554 | H | MF | adenyl nucleotide binding |
| 0.532 | GO:0016491 | H | MF | oxidoreductase activity |
| 0.528 | GO:0055114 | H | BP | oxidation-reduction process |
| 0.527 | GO:0009059 | H | BP | macromolecule biosynthetic process |
| 0.527 | GO:0010468 | H | BP | regulation of gene expression |
| 0.525 | GO:0005740 | H | CC | mitochondrial envelope |
| 0.522 | GO:0098588 | H | CC | bounding membrane of organelle |
| 0.52 | GO:0016071 | H | BP | mRNA metabolic process |
| 0.519 | GO:0016310 | H | BP | phosphorylation |
| 0.514 | GO:0031224 | H | CC | intrinsic component of membrane |
| 0.514 | GO:0019637 | H | BP | organophosphate metabolic process |
| 0.509 | GO:0051649 | H | BP | establishment of localization in cell |
| 0.508 | GO:0019752 | H | BP | carboxylic acid metabolic process |
| 0.505 | GO:0070062 | H | CC | extracellular vesicular exosome |
| 0.502 | GO:0003676 | H | MF | nucleic acid binding |
| 0.5 | GO:0031090 | H | CC | organelle membrane |
| 0.5 | GO:0006508 | H | BP | proteolysis |
| 0.946 | GO:0044237 | L | BP | cellular metabolic process |
| 0.922 | GO:0043229 | L | CC | intracellular organelle |
| 0.916 | GO:0005737 | L | CC | cytoplasm |
| 0.901 | GO:0008152 | L | BP | metabolic process |
| 0.888 | GO:0050896 | L | BP | response to stimulus |
| 0.886 | GO:0044267 | L | BP | cellular protein metabolic process |
| 0.857 | GO:0043231 | L | CC | intracellular membrane-bounded organelle |
| 0.828 | GO:0019538 | L | BP | protein metabolic process |
| 0.824 | GO:0006338 | L | BP | chromatin remodeling |
| 0.818 | GO:0051716 | L | BP | cellular response to stimulus |
| 0.816 | GO:0036094 | L | MF | small molecule binding |
| 0.812 | GO:0016772 | L | MF | transferase activity, transferring phosphorus-containing groups |
| 0.793 | GO:0023052 | L | BP | signaling |
| 0.791 | GO:0007154 | L | BP | cell communication |
| 0.774 | GO:0032502 | L | BP | developmental process |
| 0.767 | GO:0007165 | L | BP | signal transduction |
| 0.763 | GO:0009058 | L | BP | biosynthetic process |
| 0.761 | GO:0006807 | L | BP | nitrogen compound metabolic process |
| 0.755 | GO:0006139 | L | BP | nucleobase-containing compound metabolic process |
| 0.708 | GO:0097159 | L | MF | organic cyclic compound binding |
| 0.707 | GO:0043169 | L | MF | cation binding |
| 0.701 | GO:0006464 | L | BP | cellular protein modification process |
|  |  |  |  |  |
| 0.696 | GO:0046872 | L | MF | metal ion binding |
| 0.679 | GO:0005102 | L | MF | receptor binding |
| 0.673 | GO:0043234 | L | CC | protein complex |
| 0.655 | GO:0034641 | L | BP | cellular nitrogen compound metabolic process |
| 0.647 | GO:0016787 | L | MF | hydrolase activity |
| 0.644 | GO:0007275 | L | BP | multicellular organismal development |
| 0.617 | GO:0009893 | L | BP | positive regulation of metabolic process |
| 0.573 | GO:0048856 | L | BP | anatomical structure development |
| 0.568 | GO:0046483 | L | BP | heterocycle metabolic process |
| 0.564 | GO:0009966 | L | BP | regulation of signal transduction |
| 0.563 | GO:0005634 | L | CC | nucleus |
| 0.554 | GO:0006725 | L | BP | cellular aromatic compound metabolic process |
| 0.548 | GO:0032991 | L | CC | macromolecular complex |
| 0.523 | GO:0005794 | L | CC | Golgi apparatus |

**Blast**

| **BLAST Matched UniProt ID & name** | | | **Identity** | **E Value** | **Score** |
| --- | --- | --- | --- | --- | --- |
| O64470 | SHT_ARATH | Spermidine hydroxycinnamoyl transferase | 61.013 | 0.00E+00 | 597 |
| Q8GSM7 | HST_TOBAC | Shikimate O-hydroxycinnamoyltransferase | 41.886 | 3.17E-116 | 354 |
| Q9FI78 | HST_ARATH | Shikimate O-hydroxycinnamoyltransferase | 40.088 | 1.02E-112 | 345 |
| A0PDV5 | RAS_PLESU | Rosmarinate synthase | 39.738 | 6.17E-96 | 302 |
| G0LD36 | RAS_MELOI | Rosmarinate synthase | 38.377 | 1.02E-91 | 291 |
| O23917 | HCBT2_DIACA | Anthranilate N-benzoyltransferase protein 2 | 36.797 | 2.20E-91 | 290 |
| O23918 | HCBT3_DIACA | Anthranilate N-benzoyltransferase protein 3 | 36.58 | 1.33E-90 | 288 |
| O24645 | HCBT1_DIACA | Anthranilate N-benzoyltransferase protein 1 | 37.013 | 7.83E-90 | 286 |
| Q94CD1 | HHT1_ARATH | Omega-hydroxypalmitate O-feruloyl transferase | 32.892 | 1.69E-65 | 223 |
| Q8GT21 | BEBT_CLABR | Benzyl alcohol O-benzoyltransferase | 28.667 | 8.37E-46 | 170 |
| Q8GT20 | BEBT_TOBAC | Benzyl alcohol O-benzoyltransferase | 27.594 | 7.67E-44 | 164 |
| A9ZPJ6 | AGCT1_HORVU | Agmatine coumaroyltransferase-1 | 29.067 | 2.84E-43 | 162 |
| A9ZPJ7 | AGCT2_HORVU | Agmatine coumaroyltransferase-2 | 27.716 | 2.52E-42 | 160 |
| Q9SRQ2 | CHAT_ARATH | (Z)-3-hexen-1-ol acetyltransferase | 27.912 | 8.10E-38 | 147 |
| Q5H873 | HLTT_LUPAL | 13-hydroxylupanine O-tigloyltransferase | 28.16 | 1.24E-37 | 147 |
| Q8LL69 | DBNBT_TAXCA | 3'-N-debenzoyl-2'-deoxytaxol N-benzoyltransferase | 26.696 | 1.03E-33 | 135 |
| Q9FPW3 | DBBT_TAXCU | 2-alpha-hydroxytaxane 2-O-benzoyltransferase | 28.065 | 4.87E-33 | 133 |
| Q3ZPN4 | ACMAT_VITLA | Methanol O-anthraniloyltransferase | 24.879 | 8.08E-32 | 129 |
| Q8S9G6 | T5AT_TAXWC | Taxadien-5-alpha-ol O-acetyltransferase | 25.178 | 1.20E-31 | 129 |
| Q9M6F0 | T5AT_TAXCU | Taxadien-5-alpha-ol O-acetyltransferase | 25.061 | 3.46E-30 | 124 |
| Q9FF86 | DCR_ARATH | BAHD acyltransferase DCR | 28.193 | 4.75E-30 | 124 |
| Q70PR7 | VINSY_RAUSE | Vinorine synthase | 26.464 | 7.86E-27 | 114 |
| Q9M6E2 | DBAT_TAXCU | 10-deacetylbaccatin III 10-O-acetyltransferase | 26.018 | 3.99E-25 | 110 |
| Q8GYW8 | SCT_ARATH | Spermidine coumaroyl CoA acyltransferase | 26.512 | 1.92E-24 | 108 |
| Q9ZWR8 | ANTA_GENTR | Anthocyanin 5-aromatic acyltransferase | 25.463 | 7.22E-23 | 103 |
| Q9ZTK5 | DAT_CATRO | Deacetylvindoline O-acetyltransferase | 24.625 | 8.45E-22 | 100 |
| K4D9Y4 | ASAT3_SOLLC | Acylsugar acyltransferase 3 | 24.374 | 1.18E-21 | 100 |
| Q9FI40 | BAHD1_ARATH | BAHD acyltransferase At5g47980 | 27.563 | 4.07E-21 | 98.6 |
| Q9LJB4 | 5MAT_ARATH | Malonyl-CoA:anthocyanidin 5-O-glucoside-6''-O-malonyltransferase | 26.961 | 4.30E-21 | 98.6 |
| Q9SND9 | Y3028_ARATH | Uncharacterized acetyltransferase At3g50280 | 25.41 | 7.87E-21 | 97.8 |
| Q94FT4 | SALAT_PAPSO | Salutaridinol 7-O-acetyltransferase | 25.169 | 9.90E-21 | 97.8 |
| O80467 | SDT_ARATH | Spermidine sinapoyl CoA acyltransferase | 23.684 | 3.80E-20 | 95.9 |
| Q6TXD2 | 5MAT2_SALSN | Pelargonidin 3-O- | 24.242 | 6.43E-20 | 94.7 |
| Q9MBC1 | 3AT_PERFR | Anthocyanidin 3-O-glucoside 6''-O-acyltransferase | 26.712 | 4.86E-18 | 89.7 |
| O64988 | BEATH_CLABR | Acetyl-CoA-benzylalcohol acetyltransferase | 24.454 | 8.91E-18 | 88.6 |
| Q9LRQ8 | PMAT2_ARATH | Phenolic glucoside malonyltransferase 2 | 23.587 | 1.45E-17 | 88.2 |
| Q9SPU3 | BEATL_CLABR | Acetyl-CoA-benzylalcohol acetyltransferase | 24.672 | 2.52E-17 | 87.4 |
| Q8GSN8 | 3MAT_DAHPI | Malonyl-coenzyme A:anthocyanin 3-O-glucoside-6''-O-malonyltransferase | 23.146 | 7.26E-16 | 83.2 |
| Q940Z5 | PMAT1_ARATH | Phenolic glucoside malonyltransferase 1 | 25.526 | 2.43E-15 | 81.6 |
| Q9FNP9 | AGCT_ARATH | Agmatine coumaroyltransferase | 22.87 | 6.42E-15 | 80.1 |
| Q8W1W9 | 5MAT1_SALSN | Malonyl-coenzyme:anthocyanin 5-O-glucoside-6'''-O-malonyltransferase | 26.683 | 1.58E-13 | 75.9 |
| Q9LRQ7 | BAHD2_ARATH | BAHD acyltransferase At3g29680 | 22.791 | 2.02E-13 | 75.5 |
| Q9ZWB4 | 3AT1_ARATH | Coumaroyl-CoA:anthocyanidin 3-O-glucoside-6''-O-coumaroyltransferase 1 | 23.799 | 1.41E-12 | 72.8 |
| Q9LR73 | 3AT2_ARATH | Coumaroyl-CoA:anthocyanidin 3-O-glucoside-6''-O-coumaroyltransferase 2 | 23.788 | 9.81E-12 | 70.1 |
| Q9LIS1 | CR26L_ARATH | Protein ECERIFERUM 26-like | 22.156 | 3.73E-08 | 58.9 |
| Q9SVM9 | CER26_ARATH | Protein ECERIFERUM 26 | 24.194 | 6.86E-08 | 57.8 |

**Protein Name: BatroxPLA2**

**Predicted results:**

**SVMProt**

| **Protein Family Name** | **GO Category** | **SVM** | **PNN** | **KNN** |
| --- | --- | --- | --- | --- |
| **Molecular Function** | | | | |
| EC3.1 Hydrolases - Acting on Ester Bonds | - | 99 | Y | Y |
| All lipid-binding proteins | GO:0008289 lipid binding | 94.2 | - | - |
| Zinc-binding | GO:0008270 zinc binding | 76.2 | - | - |
| Metal-binding | GO:0046872 metal ion binding | 62.2 | - | - |
| EC5.2 Isomerases - cis-trans-Isomerases | - | 62.2 | ? | ? |
| Magnesium-binding | GO:0000287 magnesium binding | 58.6 | ? | ? |
| Calcium-binding | - | 58.6 | - | - |
| **Biological Process** | | | | |
| Hypotensive agent | GO:0008217 regulation of blood pressure | 99.9 | Y | Y |
| Protease inhibitor | GO:0010466 negative regulation of peptidase activity; GO:0030414 peptidase inhibitor activity | 83.2 | - | Y |
| Lipid degradation | GO:0016042 lipid catabolic process | 97 | ? | ? |
| DNA repair | GO:0006281 DNA repair | 58.6 | ? | ? |
| Pathogenesis-related protein | GO:0009607 response to biotic stimulus | 52.8 | - | - |
| **Broadly Defined Function** | | | | |
| Toxin | - | 99.9 | Y | Y |
| Antimicrobial | - | 82.3 | Y | - |

**FFPred**

| **Score** | **GO term** | **RL** | **Domain** | **Description** |
| --- | --- | --- | --- | --- |
| 0.993 | GO:0008270 | H | MF | zinc ion binding |
| 0.941 | GO:0019222 | H | BP | regulation of metabolic process |
| 0.939 | GO:0046914 | H | MF | transition metal ion binding |
| 0.935 | GO:0003824 | H | MF | catalytic activity |
| 0.858 | GO:0051171 | H | BP | regulation of nitrogen compound metabolic process |
| 0.839 | GO:0016740 | H | MF | transferase activity |
| 0.834 | GO:0010468 | H | BP | regulation of gene expression |
| 0.808 | GO:0006955 | H | BP | immune response |
| 0.759 | GO:0034645 | H | BP | cellular macromolecule biosynthetic process |
| 0.759 | GO:0006796 | H | BP | phosphate-containing compound metabolic process |
| 0.752 | GO:0008092 | H | MF | cytoskeletal protein binding |
| 0.742 | GO:0055085 | H | BP | transmembrane transport |
| 0.74 | GO:0006355 | H | BP | regulation of transcription, DNA-templated |
| 0.736 | GO:0051252 | H | BP | regulation of RNA metabolic process |
| 0.727 | GO:0044281 | H | BP | small molecule metabolic process |
| 0.712 | GO:0006810 | H | BP | transport |
| 0.689 | GO:0005576 | H | CC | extracellular region |
| 0.672 | GO:0005739 | H | CC | mitochondrion |
| 0.67 | GO:0009056 | H | BP | catabolic process |
| 0.669 | GO:2001141 | H | BP | regulationoof RNA biosynthetic process |
| 0.659 | GO:1903506 | H | BP | regulation of nucleic acid-templated transcription |
| 0.658 | GO:0016791 | H | MF | phosphatase activity |
| 0.654 | GO:0006508 | H | BP | proteolysis |
| 0.644 | GO:0019787 | H | MF | ubiquitin-like protein transferase activity |
| 0.622 | GO:0005509 | H | MF | calcium ion binding |
| 0.62 | GO:0004175 | H | MF | endopeptidase activity |
| 0.613 | GO:0016020 | H | CC | membrane |
| 0.612 | GO:0070062 | H | CC | extracellular vesicular exosome |
| 0.602 | GO:0016567 | H | BP | protein ubiquitination |
| 0.597 | GO:0007166 | H | BP | cell surface receptor signaling pathway |
| 0.592 | GO:0003779 | H | MF | actin binding |
| 0.591 | GO:0035639 | H | MF | purine ribonucleoside triphosphate binding |
| 0.591 | GO:0008083 | H | MF | growth factor activity |
| 0.571 | GO:0045087 | H | BP | innate immune response |
| 0.57 | GO:0005125 | H | MF | cytokine activity |
| 0.559 | GO:0016491 | H | MF | oxidoreductase activity |
| 0.559 | GO:0010466 | H | BP | negative regulation of peptidase activity |
| 0.558 | GO:0000166 | H | MF | nucleotide binding |
| 0.558 | GO:0005975 | H | BP | carbohydrate metabolic process |
| 0.557 | GO:0009059 | H | BP | macromolecule biosynthetic process |
| 0.557 | GO:0004857 | H | MF | enzyme inhibitor activity |
| 0.555 | GO:0030234 | H | MF | enzyme regulator activity |
| 0.555 | GO:0001883 | H | MF | purine nucleoside binding |
| 0.518 | GO:0001882 | H | MF | nucleoside binding |
| 0.516 | GO:0055114 | H | BP | oxidation-reduction process |
| 0.514 | GO:0006357 | H | BP | regulation of transcription from RNA polymerase II promoter |
| 0.512 | GO:0005740 | H | CC | mitochondrial envelope |
| 0.511 | GO:0017076 | H | MF | purine nucleotide binding |
| 0.506 | GO:0031982 | H | CC | vesicle |
| 0.981 | GO:0046872 | L | MF | metal ion binding |
| 0.939 | GO:0008152 | L | BP | metabolic process |
| 0.938 | GO:0005737 | L | CC | cytoplasm |
| 0.928 | GO:0043169 | L | MF | cation binding |
| 0.923 | GO:0043229 | L | CC | intracellular organelle |
| 0.921 | GO:0044237 | L | BP | cellular metabolic process |
| 0.912 | GO:0019538 | L | BP | protein metabolic process |
| 0.897 | GO:0043231 | L | CC | intracellular membrane-bounded organelle |
| 0.887 | GO:0009058 | L | BP | biosynthetic process |
| 0.879 | GO:0050896 | L | BP | response to stimulus |
| 0.868 | GO:0023052 | L | BP | signaling |
| 0.854 | GO:0005102 | L | MF | receptor binding |
| 0.846 | GO:0044267 | L | BP | cellular protein metabolic process |
| 0.842 | GO:0016787 | L | MF | hydrolase activity |
| 0.836 | GO:0051716 | L | BP | cellular response to stimulus |
| 0.817 | GO:0007165 | L | BP | signal transduction |
| 0.811 | GO:0032991 | L | CC | macromolecular complex |
| 0.811 | GO:0007154 | L | BP | cell communication |
| 0.777 | GO:0009893 | L | BP | positive regulation of metabolic process |
| 0.758 | GO:0005634 | L | CC | nucleus |
| 0.75 | GO:0034641 | L | BP | cellular nitrogen compound metabolic process |
| 0.736 | GO:0050790 | L | BP | regulation of catalytic activity |
| 0.724 | GO:0046483 | L | BP | heterocycle metabolic process |
| 0.724 | GO:0032502 | L | BP | developmental process |
| 0.716 | GO:0048856 | L | BP | anatomical structure development |
| 0.71 | GO:0006725 | L | BP | cellular aromatic compound metabolic process |
| 0.685 | GO:0006464 | L | BP | cellular protein modification process |
| 0.678 | GO:0006807 | L | BP | nitrogen compound metabolic process |
| 0.663 | GO:0007275 | L | BP | multicellular organismal development |
| 0.662 | GO:0032269 | L | BP | negative regulation of cellular protein metabolic process |
| 0.645 | GO:0043234 | L | CC | protein complex |
| 0.64 | GO:0097159 | L | MF | organic cyclic compound binding |
| 0.638 | GO:0005829 | L | CC | cytosol |
| 0.624 | GO:0031981 | L | CC | nuclear lumen |
| 0.602 | GO:0048513 | L | BP | organ development |
| 0.584 | GO:0009966 | L | BP | regulation of signal transduction |
| 0.572 | GO:0006139 | L | BP | nucleobase-containing compound metabolic process |
| 0.566 | GO:0005654 | L | CC | nucleoplasm |
| 0.559 | GO:0032403 | L | MF | protein complex binding |
| 0.558 | GO:0036094 | L | MF | small molecule binding |
| 0.554 | GO:0006996 | L | BP | organelle organization |
| 0.553 | GO:0010467 | L | BP | gene expression |
| 0.542 | GO:0030154 | L | BP | cell differentiation |
| 0.517 | GO:0031325 | L | BP | positive regulation of cellular metabolic process |
| 0.512 | GO:0016070 | L | BP | RNA metabolic process |

**Blast**

| **BLAST Matched UniProt ID & name** | | | **Identity** | **E Value** | **Score** |
| --- | --- | --- | --- | --- | --- |
| O42192 | PA2A8_GLOHA | Acidic phospholipase A2 A' | 96.721 | 1.54E-83 | 246 |
| P20476 | PA2A1_TRIGA | Acidic phospholipase A2 1 | 92.562 | 4.62E-80 | 238 |
| Q6H3D0 | PA2AA_TRIST | Acidic phospholipase A2 Ts-A1 | 92.562 | 4.77E-80 | 238 |
| P81480 | PA2A3_TRIGA | Acidic phospholipase A2 3 | 91.803 | 6.05E-80 | 237 |
| P81479 | PA2A4_TRIGA | Acidic phospholipase A2 4 | 91.736 | 7.86E-79 | 234 |
| Q2HZ28 | PA2A_BOTER | Acidic phospholipase A2 BE-I-PLA2 | 90.164 | 1.19E-78 | 234 |
| Q9I8F8 | PA2A_BOTPC | Acidic phospholipase A2 | 86.066 | 2.34E-76 | 228 |
| P84651 | PA2A_LACST | Acidic phospholipase A2 | 82.787 | 2.95E-71 | 215 |
| P86907 | PA2A_BOTAM | Acidic phospholipase A2 | 80.992 | 2.05E-70 | 213 |
| P00625 | PA2A1_OVOOK | Acidic phospholipase A2 DE-I | 81.148 | 1.45E-69 | 211 |
| A8E2V8 | PA2A_TRIGS | Acidic phospholipase A2 Tgc-E6 | 80.488 | 1.56E-69 | 211 |
| Q2YHJ5 | PA2AB_TRIPE | Acidic phospholipase A2 Tpu-E6b | 79.508 | 2.54E-69 | 210 |
| G3DT18 | PA2A_BOTMO | Acidic phospholipase A2 BmooPLA2 | 81.148 | 5.07E-69 | 210 |
| Q92147 | PA2AP_PROFL | Acidic phospholipase A2 pgPLA 1b/pgPLA 2b | 79.339 | 3.27E-68 | 208 |
| Q6H3C8 | PA2AF_TRIST | Acidic phospholipase A2 Ts-A6 | 77.686 | 1.02E-66 | 204 |
| P70088 | PA2A6_TRIGA | Acidic phospholipase A2 6 | 77.686 | 1.12E-66 | 204 |
| P81478 | PA2A2_TRIGA | Acidic phospholipase A2 2 | 76.23 | 1.59E-66 | 203 |
| Q7LZQ4 | PA2A_GLOUS | Acidic phospholipase A2 | 79.032 | 2.35E-66 | 202 |
| O42191 | PA2A7_GLOHA | Acidic phospholipase A2 A | 79.032 | 3.40E-66 | 202 |
| Q7ZTA7 | PA2AD_CROOA | Acidic phospholipase A2 CoaPLA2 | 77.049 | 5.05E-66 | 202 |
| P00623 | PA2A_CROAD | Acidic phospholipase A2 beta | 77.049 | 5.34E-66 | 202 |
| P00624 | PA2A_CROAT | Acidic phospholipase A2 | 77.049 | 5.95E-66 | 202 |
| C9DPL5 | PA2A1_BOTPI | Acidic phospholipase A2 BpirPLA2-I | 78.689 | 1.93E-65 | 200 |
| Q6EAN6 | PA2A_SISCT | Acidic phospholipase A2 homolog sistruxin A | 76.23 | 2.32E-65 | 201 |
| P14418 | PA2A_GLOHA | Acidic phospholipase A2 | 78.226 | 2.50E-65 | 200 |
| Q7SID6 | PA2A_DEIAC | Acidic phospholipase A2 | 73.984 | 7.19E-65 | 199 |
| D6MKR0 | PA2A6_CROHD | Acidic phospholipase A2 CH-E6' | 75.41 | 7.94E-65 | 199 |
| Q7ZTA8 | PA2AE_CROVV | Acidic phospholipase A2 Cvv-E6e | 76.23 | 1.16E-64 | 199 |
| Q6H3C9 | PA2AC_TRIST | Acidic phospholipase A2 Ts-A3 | 78.049 | 1.30E-64 | 199 |
| P86456 | PA2A4_BOTAL | Acidic phospholipase A2 SpII RP4 | 76.23 | 6.10E-64 | 196 |
| Q6H3C6 | PA2AI_TRIST | Acidic phospholipase A2 CTs-A3 | 77.236 | 1.71E-63 | 195 |
| C0HJC1 | PA2_BOTLA | Acidic phospholipase A2 BlatPLA2 | 72.131 | 3.29E-63 | 194 |
| P18998 | PA2A_CROSS | Phospholipase A2 homolog mojave toxin acidic chain | 77.049 | 6.81E-63 | 194 |
| P82896 | PA2A5_TRIST | Acidic phospholipase A2 5 | 73.77 | 7.13E-63 | 194 |
| P51972 | PA2B1_AGKPI | Basic phospholipase A2 APP-D49 | 72.358 | 1.10E-62 | 193 |
| Q800C4 | PA2AA_CROVV | Acidic phospholipase A2 Cvv-E6a | 73.77 | 4.93E-62 | 192 |
| P08878 | PA1A_CRODU | Phospholipase A2 homolog crotoxin acid subunit CA | 75.41 | 1.78E-61 | 191 |
| Q8AXY1 | PA2A_BOTJR | Acidic phospholipase A2 BthA-1 | 72.951 | 4.89E-61 | 189 |
| P0DJP4 | PA2AC_TRIPE | Acidic phospholipase A2 Tpu-E6c | 68.852 | 1.19E-60 | 188 |
| D0UGJ0 | PA2A_BOTPA | Acidic phospholipase A2 BpPLA2-TXI | 73.171 | 3.21E-60 | 187 |
| O42190 | PA2A6_GLOHA | Acidic phospholipase A2 BA2 | 71.774 | 3.26E-60 | 187 |
| Q2YHJ6 | PA2AA_TRIPE | Acidic phospholipase A2 Tpu-E6a | 68.033 | 5.13E-60 | 187 |
| Q9PVF2 | PA2AE_CALRH | Acidic phospholipase A2 H1E6 | 71.311 | 1.16E-59 | 186 |
| Q6H3C7 | PA2AD_TRIST | Acidic phospholipase A2 Ts-A4 | 71.311 | 1.25E-59 | 186 |
| Q800C3 | PA2AF_CROVV | Acidic phospholipase A2 Cvv-E6f | 68.852 | 1.39E-59 | 186 |
| Q800C1 | PA2AH_CROVV | Acidic phospholipase A2 Cvv-E6h | 69.672 | 1.50E-59 | 186 |
| Q2YHJ3 | PA2A_TRIBO | Acidic phospholipase A2 Tbo-E6 | 68.033 | 2.15E-59 | 185 |
| Q9PVF0 | PA2AB_CALRH | Acidic phospholipase A2 S1E6-b | 71.545 | 2.22E-59 | 185 |
| Q9PVE9 | PA2AC_CALRH | Acidic phospholipase A2 S1E6-c | 70.732 | 4.26E-59 | 184 |
| Q9PVF1 | PA2AA_CALRH | Acidic phospholipase A2 S1E6-a | 70.732 | 4.66E-59 | 184 |
| P86389 | PA2A2_BOTAS | Acidic phospholipase A2 2 | 70.161 | 1.42E-58 | 183 |
| Q91506 | PA2A1_PROMU | Acidic phospholipase A2 1 | 70.732 | 2.77E-58 | 182 |
| Q8QG87 | PA2A_BOTIN | Acidic phospholipase A2 BITP01A | 70.492 | 3.06E-58 | 182 |
| Q2PG83 | PA2A_PROEL | Acidic phospholipase A2 PePLA2 | 71.311 | 4.72E-58 | 182 |
| Q800C2 | PA2AG_CROVV | Acidic phospholipase A2 Cvv-E6g | 67.213 | 7.62E-58 | 181 |
| O42189 | PA2A5_GLOHA | Acidic phospholipase A2 BA1 | 70.968 | 9.65E-57 | 178 |
| P06859 | PA2A1_PROFL | Acidic phospholipase A2 1 | 69.672 | 1.80E-56 | 178 |
| Q7ZTA6 | PA2AB_CROVV | Acidic phospholipase A2 Cvv-E6b | 76.23 | 2.12E-56 | 177 |
| P81243 | PA2A_BOTJA | Acidic phospholipase A2 | 68.293 | 1.60E-53 | 170 |
| O42187 | PA2BB_GLOHA | Basic phospholipase A2 B | 62.295 | 1.66E-52 | 168 |
| O42188 | PA2H_GLOHA | Basic phospholipase A2 homolog | 61.475 | 1.07E-51 | 165 |
| Q8JIG0 | PA2BQ_PROFL | Basic phospholipase A2 PLA-B' | 62.81 | 1.70E-51 | 165 |
| Q02517 | PA2BW_PROFL | Basic phospholipase A2 PL-X' | 62.81 | 1.89E-51 | 165 |
| Q90Y77 | PA2BY_PROFL | Basic phospholipase A2 PL-Y | 62.81 | 9.84E-51 | 163 |
| P04417 | PA2B_GLOBL | Basic phospholipase A2 | 61.983 | 1.14E-50 | 162 |
| P34180 | PA2N2_VIPAA | Neutral phospholipase A2 ammodytin I2 | 59.167 | 6.80E-50 | 161 |
| P0DKU1 | PA2H_GLOUS | Basic phospholipase A2 homolog Gln49-PLA2 | 60.656 | 1.48E-49 | 160 |
| Q910A0 | PA23_ECHCO | Phospholipase A2 EC3 | 59.504 | 1.88E-49 | 160 |
| A8CG87 | PA2A2_DABRR | Acidic phospholipase A2 Drk-a2 | 59.836 | 1.04E-48 | 158 |
| P31854 | PA2B_VIPBB | Basic phospholipase A2 Pla2Vb | 57.377 | 1.38E-48 | 158 |
| B5U6Z2 | PA2A_MACLN | Acidic phospholipase A2 MVL-PLA2 | 60.331 | 2.79E-48 | 157 |
| F8QN53 | PA2A2_VIPRE | Acidic phospholipase A2 Vur-PL2B | 59.167 | 3.80E-48 | 157 |
| Q6EER3 | PA2B2_SISCT | Basic phospholipase A2 Sct-N6 | 57.377 | 4.75E-48 | 156 |
| Q98996 | PA2A_DABPA | Acidic phospholipase A2 VpaPLA2 | 60.331 | 4.91E-48 | 156 |
| F8QN52 | PA2A1_VIPRE | Acidic phospholipase A2 PL1 | 59.836 | 5.59E-48 | 156 |
| Q9PWR6 | PA2A7_DABPA | Acidic phospholipase A2 VP7 | 60.331 | 6.23E-48 | 156 |
| Q9YGJ7 | PA2A8_DABPA | Acidic phospholipase A2 VP8 | 59.836 | 7.27E-48 | 156 |
| P06860 | PA2BX_PROFL | Basic phospholipase A2 PL-X | 61.983 | 7.28E-48 | 155 |
| C3W4R6 | PA2A1_MACLB | Acidic phospholipase A2 1 | 60.331 | 7.74E-48 | 156 |
| Q910A1 | PA2A1_VIPAA | Acidic phospholipase A2 ammodytin I1 | 60.331 | 1.29E-47 | 155 |
| P45881 | PA2B2_BOTJR | Basic phospholipase A2 bothropstoxin-2 | 57.851 | 3.57E-47 | 154 |
| P59265 | PA2BB_PROFL | Basic phospholipase A2 PLA-B | 61.157 | 7.74E-47 | 153 |
| P59171 | PA2A5_ECHOC | Acidic phospholipase A2 5 | 56.557 | 1.02E-46 | 153 |
| B6CQR5 | PA2A2_MACLB | Acidic phospholipase A2 2 | 59.504 | 1.21E-46 | 153 |
| F8QN51 | PA2A3_VIPRE | Acidic phospholipase A2 Vur-PL3 | 56.667 | 1.46E-46 | 152 |
| P86974 | PA2BD_BOTLC | Basic phospholipase A2 | 57.851 | 2.31E-46 | 152 |
| Q92152 | PA2H_OVOOK | Phospholipase A2 homolog PLA2-03 | 60.656 | 2.74E-46 | 152 |
| Q9I968 | PA2A2_PROMU | Acidic phospholipase A2 2 | 58.197 | 3.39E-46 | 152 |
| P59264 | PA2BA_PROFL | Basic phospholipase A2 PLA-A | 60.331 | 4.29E-46 | 151 |
| Q90ZZ9 | PA21_ECHCO | Phospholipase A2 EC1 | 54.918 | 5.41E-46 | 151 |
| Q3HLQ4 | PA2HB_PROMU | Basic phospholipase A2 homolog TM-N49 | 55.738 | 5.96E-46 | 151 |
| P20474 | PA2B3_BOTAS | Basic phospholipase A2 myotoxin III | 54.098 | 1.22E-45 | 150 |
| Q6EER4 | PA2B_BOTSC | Basic phospholipase A2 Bs-N6 | 56.557 | 1.31E-45 | 150 |
| Q7T3T5 | PA2AB_DABSI | Acidic phospholipase A2 daboiatoxin B chain | 57.377 | 2.01E-45 | 149 |
| A8CG86 | PA2A1_DABRR | Acidic phospholipase A2 Drk-a1 | 54.918 | 2.11E-45 | 150 |
| P24293 | PA2A1_ERIMA | Acidic phospholipase A2 PLA-1 | 55 | 2.21E-45 | 149 |
| Q71QE8 | PA2BN_CROVV | Basic phospholipase A2 Cvv-N6 | 53.279 | 3.63E-45 | 149 |
| P59170 | PA2A4_ECHCS | Acidic phospholipase A2 4 | 54.472 | 3.95E-45 | 149 |
| P59172 | PA2A5_ECHPL | Acidic phospholipase A2 5 | 54.472 | 4.12E-45 | 149 |

**Protein Name: Maheshvara**

**Predicted results:**

**SVMProt**

| **Protein Family Name** | **GO Category** | **SVM** | **PNN** | **KNN** |
| --- | --- | --- | --- | --- |
| **Molecular Function** | | | | |
| EC1.1 Oxidoreductases - Acting on the CH-OH group of donors | - | 85.4 | - | - |
| Zinc-binding | GO:0008270 zinc binding | 83.9 | - | - |
| All DNA-binding | GO:0003677 DNA binding | 73.8 | ? | ? |
| Metal-binding | GO:0046872 metal ion binding | 62.2 | - | - |
| mRNA slicing | - | 58.6 | ? | ? |
| mRNA-binding proteins | GO:0003729 mRNA binding | 58.6 | - | - |
| Calcium-binding | - | 58.6 | - | - |
| Magnesium-binding | GO:0000287 magnesium binding | 58.6 | ? | ? |
| RNA-binding proteins | GO:0003723 RNA binding | 58.6 | ? | ? |
| **Broadly Defined Function** | | | | |
| Photosystem I | GO:0009522 photosystem I | 58.6 | ? | ? |

**FFPred**

| **Score** | **GO term** | **RL** | **Domain** | **Description** |
| --- | --- | --- | --- | --- |
| 0.988 | GO:0003676 | H | MF | nucleic acid binding |
| 0.971 | GO:0003677 | H | MF | DNA binding |
| 0.926 | GO:0019222 | H | BP | regulation of metabolic process |
| 0.916 | GO:0051171 | H | BP | regulation of nitrogen compound metabolic process |
| 0.91 | GO:0003824 | H | MF | catalytic activity |
| 0.899 | GO:0010468 | H | BP | regulation of gene expression |
| 0.893 | GO:2001141 | H | BP | regulation of RNA biosynthetic process |
| 0.892 | GO:0006351 | H | BP | transcription, DNA-templated |
| 0.891 | GO:0034645 | H | BP | cellular macromolecule biosynthetic process |
| 0.886 | GO:0000981 | H | MF | sequence-specific DNA binding RNA polymerase II transcription factor activity |
| 0.875 | GO:0000166 | H | MF | nucleotide binding |
| 0.874 | GO:0051252 | H | BP | regulation of RNA metabolic process |
| 0.871 | GO:0009059 | H | BP | macromolecule biosynthetic process |
| 0.857 | GO:1903506 | H | BP | regulation of nucleic acid-templated transcription |
| 0.829 | GO:0006355 | H | BP | regulation of transcription, DNA-templated |
| 0.812 | GO:0010557 | H | BP | positive regulation of macromolecule biosynthetic process |
| 0.763 | GO:0000122 | H | BP | negative regulation of transcription from RNA polymerase II promoter |
| 0.75 | GO:0005730 | H | CC | nucleolus |
| 0.742 | GO:0017076 | H | MF | purine nucleotide binding |
| 0.728 | GO:0003723 | H | MF | RNA binding |
| 0.725 | GO:0044212 | H | MF | transcription regulatory region DNA binding |
| 0.69 | GO:0031328 | H | BP | positive regulation of cellular biosynthetic process |
| 0.684 | GO:0016604 | H | CC | nuclear body |
| 0.668 | GO:0045893 | H | BP | positive regulation of transcription, DNA-templated |
| 0.663 | GO:0030554 | H | MF | adenyl nucleotide binding |
| 0.662 | GO:0035639 | H | MF | purine ribonucleoside triphosphate binding |
| 0.636 | GO:0010628 | H | BP | positive regulation of gene expression |
| 0.631 | GO:0005524 | H | MF | ATP binding |
| 0.627 | GO:0010629 | H | BP | negative regulation of gene expression |
| 0.626 | GO:0044822 | H | MF | poly(A) RNA binding |
| 0.62 | GO:0043565 | H | MF | sequence-specific DNA binding |
| 0.611 | GO:0032549 | H | MF | ribonucleoside binding |
| 0.61 | GO:1903507 | H | BP | negative regulation of nucleic acid-templated transcription |
| 0.607 | GO:0006357 | H | BP | regulation of transcription from RNA polymerase II promoter |
| 0.597 | GO:0000975 | H | MF | regulatory region DNA binding |
| 0.567 | GO:0009890 | H | BP | negative regulation of biosynthetic process |
| 0.564 | GO:0001071 | H | MF | nucleic acid binding transcription factor activity |
| 0.562 | GO:0001883 | H | MF | purine nucleoside binding |
| 0.559 | GO:0030529 | H | CC | ribonucleoprotein complex |
| 0.551 | GO:0006366 | H | BP | transcription from RNA polymerase II promoter |
| 0.506 | GO:0006396 | H | BP | RNA processing |
| 0.987 | GO:0008152 | L | BP | metabolic process |
| 0.978 | GO:0097159 | L | MF | organic cyclic compound binding |
| 0.97 | GO:0044237 | L | BP | cellular metabolic process |
| 0.968 | GO:0043229 | L | CC | intracellular organelle |
| 0.964 | GO:0006807 | L | BP | nitrogen compound metabolic process |
| 0.941 | GO:0006139 | L | BP | nucleobase-containing compound metabolic process |
| 0.932 | GO:0043231 | L | CC | intracellular membrane-bounded organelle |
| 0.904 | GO:0009058 | L | BP | biosynthetic process |
| 0.874 | GO:0005634 | L | CC | nucleus |
| 0.85 | GO:0034641 | L | BP | cellular nitrogen compound metabolic process |
| 0.849 | GO:0046483 | L | BP | heterocycle metabolic process |
| 0.844 | GO:0006725 | L | BP | cellular aromatic compound metabolic process |
| 0.841 | GO:0031981 | L | CC | nuclear lumen |
| 0.831 | GO:0036094 | L | MF | small molecule binding |
| 0.825 | GO:0016070 | L | BP | RNA metabolic process |
| 0.816 | GO:0010467 | L | BP | gene expression |
| 0.809 | GO:0005654 | L | CC | nucleoplasm |
| 0.78 | GO:0032502 | L | BP | developmental process |
| 0.777 | GO:0051716 | L | BP | cellular response to stimulus |
| 0.775 | GO:0050896 | L | BP | response to stimulus |
| 0.758 | GO:0048856 | L | BP | anatomical structure development |
| 0.733 | GO:0005737 | L | CC | cytoplasm |
| 0.722 | GO:0007275 | L | BP | multicellular organismal development |
| 0.715 | GO:0009893 | L | BP | positive regulation of metabolic process |
| 0.706 | GO:0032991 | L | CC | macromolecular complex |
| 0.704 | GO:0008134 | L | MF | transcription factor binding |
| 0.703 | GO:0005829 | L | CC | cytosol |
| 0.68 | GO:0006996 | L | BP | organelle organization |
| 0.644 | GO:0043234 | L | CC | protein complex |
| 0.641 | GO:0031324 | L | BP | negative regulation of cellular metabolic process |
| 0.636 | GO:0030154 | L | BP | cell differentiation |
| 0.63 | GO:0031325 | L | BP | positive regulation of cellular metabolic process |
| 0.613 | GO:0005102 | L | MF | receptor binding |
| 0.591 | GO:0043169 | L | MF | cation binding |
| 0.582 | GO:0048513 | L | BP | organ development |
| 0.581 | GO:0007154 | L | BP | cell communication |
| 0.567 | GO:0009966 | L | BP | regulation of signal transduction |
| 0.545 | GO:0016787 | L | MF | hydrolase activity |
| 0.543 | GO:0046872 | L | MF | metal ion binding |

**Blast**

| **BLAST Matched UniProt ID & name** | | | **Identity** | **E Value** | **Score** |
| --- | --- | --- | --- | --- | --- |
| Q5R4I9 | DDX5_PONAB | Probable ATP-dependent RNA helicase DDX5 | 61.863 | 0 | 585 |
| Q61656 | DDX5_MOUSE | Probable ATP-dependent RNA helicase DDX5 | 61.641 | 0 | 583 |
| P17844 | DDX5_HUMAN | Probable ATP-dependent RNA helicase DDX5 | 61.641 | 0 | 582 |
| A5A6J2 | DDX5_PANTR | Probable ATP-dependent RNA helicase DDX5 | 61.641 | 0 | 580 |
| Q4R6M5 | DDX5_MACFA | Probable ATP-dependent RNA helicase DDX5 | 61.419 | 0 | 578 |
| Q501J6 | DDX17_MOUSE | Probable ATP-dependent RNA helicase DDX17 | 62.168 | 0 | 577 |
| Q92841 | DDX17_HUMAN | Probable ATP-dependent RNA helicase DDX17 | 58.943 | 0 | 577 |
| P19109 | DDX17_DROME | ATP-dependent RNA helicase p62 | 61.468 | 0 | 557 |
| Q5B0J9 | DBP2_EMENI | ATP-dependent RNA helicase dbp2 | 60.177 | 0.00E+00 | 554 |
| A1DGZ7 | DBP2_NEOFI | ATP-dependent RNA helicase dbp2 | 58.85 | 0.00E+00 | 548 |
| Q4X195 | DBP2_ASPFU | ATP-dependent RNA helicase dbp2 | 58.628 | 0.00E+00 | 546 |
| A6RGE3 | DBP2_AJECN | ATP-dependent RNA helicase DBP2 | 58.85 | 0.00E+00 | 543 |
| Q2H720 | DBP2_CHAGB | ATP-dependent RNA helicase DBP2 | 59.821 | 2.39E-180 | 541 |
| A2QC74 | DBP2_ASPNC | ATP-dependent RNA helicase dbp2 | 58.186 | 2.54E-179 | 538 |
| Q1DP69 | DBP2_COCIM | ATP-dependent RNA helicase DBP2 | 59.513 | 1.73E-178 | 535 |
| Q7SBC6 | DBP2_NEUCR | ATP-dependent RNA helicase dbp2 | 59.417 | 1.13E-177 | 534 |
| A4QSS5 | DBP2_MAGO7 | ATP-dependent RNA helicase DBP2 | 57.65 | 1.37E-177 | 533 |
| Q4IF76 | DBP2_GIBZE | ATP-dependent RNA helicase DBP2 | 58.427 | 1.84E-177 | 533 |
| P24782 | DBP2_SCHPO | ATP-dependent RNA helicase dbp2 | 57.718 | 3.24E-177 | 532 |
| A7E449 | DBP2_SCLS1 | ATP-dependent RNA helicase dbp2 | 58.407 | 1.55E-174 | 526 |
| A1C6C4 | DBP2_ASPCL | ATP-dependent RNA helicase dbp2 | 55.11 | 1.05E-173 | 523 |
| Q6C4D4 | DBP2_YARLI | ATP-dependent RNA helicase DBP2 | 58.661 | 2.34E-173 | 522 |
| Q2U070 | DBP2_ASPOR | ATP-dependent RNA helicase dbp2 | 54.086 | 5.31E-173 | 521 |
| Q9C718 | RH20_ARATH | DEAD-box ATP-dependent RNA helicase 20 | 55.748 | 2.31E-170 | 513 |
| Q6FLF3 | DBP2_CANGA | ATP-dependent RNA helicase DBP2 | 55.705 | 2.48E-168 | 509 |
| P0CQ77 | DBP2_CRYNB | ATP-dependent RNA helicase DBP2-A | 55.125 | 2.66E-168 | 509 |
| Q4PHU9 | DBP2_USTMA | ATP-dependent RNA helicase DBP2 | 55.782 | 5.07E-168 | 508 |
| Q5QMN3 | RH20_ORYSJ | DEAD-box ATP-dependent RNA helicase 20 | 55.921 | 5.91E-168 | 506 |
| Q6CIV2 | DBP2_KLULA | ATP-dependent RNA helicase DBP2 | 56.463 | 1.47E-167 | 507 |
| Q59LU0 | DBP2_CANAL | ATP-dependent RNA helicase DBP2 | 56.522 | 1.47E-167 | 508 |
| P24783 | DBP2_YEAST | ATP-dependent RNA helicase DBP2 | 55.111 | 5.27E-167 | 506 |
| A6ZRX0 | DBP2_YEAS7 | ATP-dependent RNA helicase DBP2 | 55.111 | 5.80E-167 | 506 |
| Q5N7W4 | RH30_ORYSJ | DEAD-box ATP-dependent RNA helicase 30 | 53.39 | 3.86E-166 | 508 |
| A5DL80 | DBP2_PICGU | ATP-dependent RNA helicase DBP2 | 55.982 | 4.66E-166 | 503 |
| Q6BY27 | DBP2_DEBHA | ATP-dependent RNA helicase DBP2 | 55.305 | 7.27E-165 | 500 |
| Q8W4R3 | RH30_ARATH | DEAD-box ATP-dependent RNA helicase 30 | 54.977 | 8.07E-165 | 502 |
| A6SFW7 | DBP2_BOTFB | ATP-dependent RNA helicase dbp2 | 52.673 | 2.11E-163 | 495 |
| Q755N4 | DBP2_ASHGO | ATP-dependent RNA helicase DBP2 | 55.227 | 3.62E-162 | 493 |
| Q54CE0 | DDX17_DICDI | Probable ATP-dependent RNA helicase ddx17 | 52.36 | 8.16E-159 | 493 |
| A5DS77 | DBP2_LODEL | ATP-dependent RNA helicase DBP2 | 55.889 | 1.60E-157 | 481 |
| A3LQW7 | DBP2_PICST | ATP-dependent RNA helicase DBP2 | 55.658 | 2.14E-156 | 478 |
| Q8SRB2 | DBP2_ENCCU | ATP-dependent RNA helicase DBP2 | 49.21 | 2.75E-143 | 442 |
| A7TTT5 | DBP2_VANPO | ATP-dependent RNA helicase DBP2 | 55.673 | 1.66E-141 | 436 |
| Q5VQL1 | RH14_ORYSJ | DEAD-box ATP-dependent RNA helicase 14 | 49.631 | 1.26E-130 | 416 |
| P46942 | DB10_NICSY | ATP-dependent RNA helicase-like protein DB10 | 48.936 | 6.55E-127 | 403 |
| Q5JKF2 | RH40_ORYSJ | DEAD-box ATP-dependent RNA helicase 40 | 50.976 | 1.82E-126 | 408 |
| Q8H136 | RH14_ARATH | DEAD-box ATP-dependent RNA helicase 14 | 47.738 | 3.68E-124 | 396 |
| Q9LYJ9 | RH46_ARATH | DEAD-box ATP-dependent RNA helicase 46 | 47.608 | 9.03E-124 | 396 |
| Q9SQV1 | RH40_ARATH | DEAD-box ATP-dependent RNA helicase 40 | 49.268 | 8.52E-120 | 398 |
| Q26696 | DDX17_TRYBB | Putative DEAD-box RNA helicase HEL64 | 46.341 | 3.80E-118 | 379 |
| Q10MH8 | RH24_ORYSJ | DEAD-box ATP-dependent RNA helicase 24 | 43.722 | 1.56E-115 | 378 |
| Q8H0U8 | RH42_ARATH | DEAD-box ATP-dependent RNA helicase 42 | 43.584 | 5.81E-113 | 380 |
| Q84UQ1 | RH42_ORYSJ | DEAD-box ATP-dependent RNA helicase 42 | 42.661 | 8.74E-112 | 375 |
| Q5F485 | DDX42_CHICK | ATP-dependent RNA helicase DDX42 | 38.318 | 1.02E-110 | 370 |
| Q810A7 | DDX42_MOUSE | ATP-dependent RNA helicase DDX42 | 38.538 | 6.38E-110 | 367 |
| Q86XP3 | DDX42_HUMAN | ATP-dependent RNA helicase DDX42 | 40.123 | 1.43E-109 | 367 |
| O22907 | RH24_ARATH | DEAD-box ATP-dependent RNA helicase 24 | 42.922 | 2.50E-109 | 361 |
| Q9C551 | RH5_ARATH | DEAD-box ATP-dependent RNA helicase 5 | 46.667 | 3.67E-109 | 354 |
| Q5R7D1 | DDX42_PONAB | ATP-dependent RNA helicase DDX42 | 40.123 | 6.64E-109 | 365 |
| Q2U2J6 | PRP5_ASPOR | Pre-mRNA-processing ATP-dependent RNA helicase prp5 | 43.764 | 1.19E-108 | 369 |
| Q9NXZ2 | DDX43_HUMAN | Probable ATP-dependent RNA helicase DDX43 | 40.083 | 1.46E-108 | 356 |
| Q9P7C7 | PRP11_SCHPO | Pre-mRNA-processing ATP-dependent RNA helicase prp11 | 44.773 | 4.88E-108 | 364 |
| A4RN46 | PRP5_MAGO7 | Pre-mRNA-processing ATP-dependent RNA helicase PRP5 | 43.274 | 1.65E-107 | 362 |
| Q7ZY47 | DDX42_XENLA | ATP-dependent RNA helicase DDX42 | 40.43 | 6.74E-107 | 359 |
| Q54IV3 | DDX42_DICDI | Probable ATP-dependent RNA helicase ddx42 | 40.58 | 1.44E-106 | 359 |
| Q6YS30 | RH5_ORYSJ | DEAD-box ATP-dependent RNA helicase 5 | 45.939 | 1.63E-106 | 346 |
| Q5BDW4 | PRP5_EMENI | Pre-mRNA-processing ATP-dependent RNA helicase prp5 | 42.733 | 3.85E-106 | 362 |
| A7ENE0 | PRP5_SCLS1 | Pre-mRNA-processing ATP-dependent RNA helicase prp5 | 42.568 | 4.18E-106 | 361 |
| Q0D1K3 | PRP5_ASPTN | Pre-mRNA-processing ATP-dependent RNA helicase prp5 | 44.118 | 7.26E-106 | 361 |
| Q4PFD9 | PRP5_USTMA | Pre-mRNA-processing ATP-dependent RNA helicase PRP5 | 43.632 | 1.03E-105 | 360 |
| Q2HAD8 | PRP5_CHAGB | Pre-mRNA-processing ATP-dependent RNA helicase PRP5 | 41.474 | 1.47E-105 | 358 |
| Q4IP34 | PRP5_GIBZE | Pre-mRNA-processing ATP-dependent RNA helicase PRP5 | 43.192 | 2.02E-105 | 360 |
| A1CQA9 | PRP5_ASPCL | Pre-mRNA-processing ATP-dependent RNA helicase prp5 | 43.665 | 3.24E-105 | 360 |
| A6RW79 | PRP5_BOTFB | Pre-mRNA-processing ATP-dependent RNA helicase prp5 | 41.81 | 9.49E-105 | 357 |
| A1D373 | PRP5_NEOFI | Pre-mRNA-processing ATP-dependent RNA helicase prp5 | 43.084 | 1.38E-104 | 358 |
| A2QQA8 | PRP5_ASPNC | Pre-mRNA-processing ATP-dependent RNA helicase prp5 | 43.439 | 5.09E-104 | 356 |
| Q1DHB2 | PRP5_COCIM | Pre-mRNA-processing ATP-dependent RNA helicase PRP5 | 42.825 | 8.99E-104 | 355 |
| Q4WT99 | PRP5_ASPFU | Pre-mRNA-processing ATP-dependent RNA helicase prp5 | 42.63 | 1.19E-103 | 355 |
| Q7SH33 | PRP5_NEUCR | Pre-mRNA-processing ATP-dependent RNA helicase prp-5 | 41.277 | 1.29E-102 | 352 |
| Q62780 | DDX46_RAT | Probable ATP-dependent RNA helicase DDX46 | 40.598 | 2.41E-102 | 349 |
| Q7L014 | DDX46_HUMAN | Probable ATP-dependent RNA helicase DDX46 | 40.598 | 2.59E-102 | 348 |
| Q86TM3 | DDX53_HUMAN | Probable ATP-dependent RNA helicase DDX53 | 41.425 | 2.79E-102 | 338 |
| Q569Z5 | DDX46_MOUSE | Probable ATP-dependent RNA helicase DDX46 | 40.598 | 2.88E-102 | 348 |
| Q5R6D8 | DDX46_PONAB | Probable ATP-dependent RNA helicase DDX46 | 40.385 | 1.00E-101 | 347 |
| Q75HJ0 | RH37_ORYSJ | DEAD-box ATP-dependent RNA helicase 37 | 44.526 | 2.13E-100 | 334 |
| Q4TVV3 | DDX46_DANRE | Probable ATP-dependent RNA helicase DDX46 | 41.08 | 2.86E-100 | 343 |
| Q0DB53 | RH52A_ORYSJ | DEAD-box ATP-dependent RNA helicase 52A | 43.659 | 1.31E-98 | 328 |
| Q6Z4K6 | RH52B_ORYSJ | DEAD-box ATP-dependent RNA helicase 52B | 43.373 | 4.05E-98 | 328 |
| Q553B1 | DDX46_DICDI | ATP-dependent RNA helicase ddx46 | 41.805 | 1.19E-97 | 338 |
| P0CQ99 | PRP5_CRYNB | Pre-mRNA-processing ATP-dependent RNA helicase PRP5 | 41.608 | 1.64E-97 | 336 |
| P16381 | DDX3L_MOUSE | Putative ATP-dependent RNA helicase Pl10 | 41.629 | 4.16E-97 | 325 |
| Q6FP38 | DBP1_CANGA | ATP-dependent RNA helicase DBP1 | 40.044 | 8.09E-97 | 323 |
| Q2R1M8 | RH52C_ORYSJ | DEAD-box ATP-dependent RNA helicase 52C | 42.506 | 1.31E-96 | 323 |
| Q6BML1 | PRP5_DEBHA | Pre-mRNA-processing ATP-dependent RNA helicase PRP5 | 41.299 | 4.33E-96 | 329 |
| A7TKR8 | DED1_VANPO | ATP-dependent RNA helicase DED1 | 39.732 | 5.06E-96 | 322 |
| O15523 | DDX3Y_HUMAN | ATP-dependent RNA helicase DDX3Y | 40.498 | 7.99E-96 | 322 |
| Q62167 | DDX3X_MOUSE | ATP-dependent RNA helicase DDX3X | 42.043 | 9.58E-96 | 322 |
| Q6CLR3 | DED1_KLULA | ATP-dependent RNA helicase DED1 | 40.426 | 2.04E-95 | 320 |
| Q6GVM6 | DDX3Y_PANTR | ATP-dependent RNA helicase DDX3Y | 40.498 | 2.20E-95 | 321 |
| Q62095 | DDX3Y_MOUSE | ATP-dependent RNA helicase DDX3Y | 40.46 | 2.99E-95 | 320 |

**Protein Name: Orf1**

**Predicted results:**

**SVMProt**

| **Protein Family Name** | **GO Category** | **SVM** | **PNN** | **KNN** |
| --- | --- | --- | --- | --- |
| **Molecular Function** | | | | |
| EC4.1 Lyases - Carbon-Carbon Lyases | - | 92.9 | - | Y |
| All lipid-binding proteins | GO:0008289 lipid binding | 99 | - | - |
| Iron-binding | GO:0005506 iron binding | 98.9 | - | - |
| EC3.4 Hydrolases - Acting on peptide bonds (Peptidases) | - | 89.3 | - | - |
| EC6.1 Ligases - Forming Carbon-Oxygen Bonds | - | 68.5 | - | Y |
| EC2.3 Transferases - Acyltransferases | - | 83.9 | - | - |
| EC4.2 Lyases - Carbon-Oxygen Lyases | - | 62.2 | - | Y |
| Metal-binding | GO:0046872 metal ion binding | 78.4 | - | - |
| All DNA-binding | GO:0003677 DNA binding | 73.8 | ? | ? |
| EC2.7 Transferases - Transferring Phosphorus-Containing Groups | - | 73.8 | - | - |
| Magnesium-binding | GO:0000287 magnesium binding | 58.6 | ? | ? |
| Biological Process: |  |  |  |  |
| Lipid metabolism | GO:0006629 lipid metabolic process | 83.9 | ? | ? |
| DNA repair | GO:0006281 DNA repair | 58.6 | ? | ? |
| **Broadly Defined Function** | | | | |
| Photosystem I | GO:0009522 photosystem I | 58.6 | ? | ? |

**FFPred**

| **Score** | **GO term** | **RL** | **Domain** | **Description** |
| --- | --- | --- | --- | --- |
| 0.938 | GO:0003824 | H | MF | catalytic activity |
| 0.922 | GO:0005740 | H | CC | mitochondrial envelope |
| 0.917 | GO:0005739 | H | CC | mitochondrion |
| 0.883 | GO:0031966 | H | CC | mitochondrial membrane |
| 0.846 | GO:0032787 | H | BP | monocarboxylic acid metabolic process |
| 0.803 | GO:0005759 | H | CC | mitochondrial matrix |
| 0.785 | GO:0016740 | H | MF | transferase activity |
| 0.782 | GO:0032549 | H | MF | ribonucleoside binding |
| 0.771 | GO:0035639 | H | MF | purine ribonucleoside triphosphate binding |
| 0.762 | GO:0017076 | H | MF | purine nucleotide binding |
| 0.761 | GO:0000166 | H | MF | nucleotide binding |
| 0.743 | GO:0055114 | H | BP | oxidation-reduction process |
| 0.736 | GO:0019222 | H | BP | regulation of metabolic process |
| 0.717 | GO:0006082 | H | BP | organic acid metabolic process |
| 0.696 | GO:0005743 | H | CC | mitochondrial inner membrane |
| 0.689 | GO:0016773 | H | MF | phosphotransferase activity, alcohol group as acceptor |
| 0.689 | GO:0001883 | H | MF | purine nucleoside binding |
| 0.68 | GO:0001882 | H | MF | nucleoside binding |
| 0.671 | GO:0016746 | H | MF | transferase activity, transferring acyl groups |
| 0.664 | GO:0009056 | H | BP | catabolic process |
| 0.663 | GO:0019752 | H | BP | carboxylic acid metabolic process |
| 0.656 | GO:0009059 | H | BP | macromolecule biosynthetic process |
| 0.653 | GO:0030554 | H | MF | adenyl nucleotide binding |
| 0.653 | GO:0051171 | H | BP | regulation of nitrogen compound metabolic process |
| 0.645 | GO:0006796 | H | BP | phosphate-containing compound metabolic process |
| 0.644 | GO:0003676 | H | MF | nucleic acid binding |
| 0.637 | GO:0003723 | H | MF | RNA binding |
| 0.629 | GO:0006790 | H | BP | sulfur compound metabolic process |
| 0.621 | GO:0016491 | H | MF | oxidoreductase activity |
| 0.613 | GO:1901605 | H | BP | alpha-amino acid metabolic process |
| 0.607 | GO:0010468 | H | BP | regulation of gene expression |
| 0.604 | GO:0051252 | H | BP | regulation of RNA metabolic process |
| 0.598 | GO:0006631 | H | BP | fatty acid metabolic process |
| 0.587 | GO:0006629 | H | BP | lipid metabolic process |
| 0.582 | GO:0006355 | H | BP | regulation of transcription, DNA-templated |
| 0.578 | GO:0019637 | H | BP | organophosphate metabolic process |
| 0.572 | GO:0005524 | H | MF | ATP binding |
| 0.556 | GO:0070062 | H | CC | extracellular vesicular exosome |
| 0.542 | GO:0005975 | H | BP | carbohydrate metabolic process |
| 0.537 | GO:2001141 | H | BP | regulation of RNA biosynthetic process |
| 0.529 | GO:0051186 | H | BP | cofactor metabolic process |
| 0.524 | GO:0034645 | H | BP | cellular macromolecule biosynthetic process |
| 0.522 | GO:0031090 | H | CC | organelle membrane |
| 0.519 | GO:0031982 | H | CC | vesicle |
| 0.516 | GO:0015980 | H | BP | energy derivation by oxidation of organic compounds |
| 0.512 | GO:0016310 | H | BP | phosphorylation |
| 0.507 | GO:0016020 | H | CC | membrane |
| 0.503 | GO:0016301 | H | MF | kinase activity |
| 0.501 | GO:0032561 | H | MF | guanyl ribonucleotide binding |
| 0.962 | GO:0008152 | L | BP | metabolic process |
| 0.955 | GO:0044237 | L | BP | cellular metabolic process |
| 0.949 | GO:0043229 | L | CC | intracellular organelle |
| 0.927 | GO:0005737 | L | CC | cytoplasm |
| 0.907 | GO:0043231 | L | CC | intracellular membrane-bounded organelle |
| 0.864 | GO:0050896 | L | BP | response to stimulus |
| 0.848 | GO:0016787 | L | MF | hydrolase activity |
| 0.842 | GO:0019538 | L | BP | protein metabolic process |
| 0.827 | GO:0051716 | L | BP | cellular response to stimulus |
| 0.821 | GO:0044267 | L | BP | cellular protein metabolic process |
| 0.819 | GO:0034641 | L | BP | cellular nitrogen compound metabolic process |
| 0.817 | GO:0005634 | L | CC | nucleus |
| 0.81 | GO:0046483 | L | BP | heterocycle metabolic process |
| 0.792 | GO:0009058 | L | BP | biosynthetic process |
| 0.787 | GO:0006725 | L | BP | cellular aromatic compound metabolic process |
| 0.778 | GO:0097159 | L | MF | organic cyclic compound binding |
| 0.767 | GO:0032502 | L | BP | developmental process |
| 0.763 | GO:0006464 | L | BP | cellular protein modification process |
| 0.724 | GO:0031981 | L | CC | nuclear lumen |
| 0.715 | GO:0005829 | L | CC | cytosol |
| 0.697 | GO:0036094 | L | MF | small molecule binding |
| 0.69 | GO:0006807 | L | BP | nitrogen compound metabolic process |
| 0.683 | GO:0010467 | L | BP | gene expression |
| 0.67 | GO:0005654 | L | CC | nucleoplasm |
| 0.664 | GO:0016070 | L | BP | RNA metabolic process |
| 0.657 | GO:0032991 | L | CC | macromolecular complex |
| 0.63 | GO:0006996 | L | BP | organelle organization |
| 0.617 | GO:0043234 | L | CC | protein complex |
| 0.604 | GO:0007275 | L | BP | multicellular organismal development |
| 0.562 | GO:0007154 | L | BP | cell communication |
| 0.561 | GO:0016772 | L | MF | transferase activity, transferring phosphorus-containing groups |
| 0.553 | GO:0043169 | L | MF | cation binding |
| 0.551 | GO:0048856 | L | BP | anatomical structure development |
| 0.542 | GO:0010033 | L | BP | response to organic substance |
| 0.529 | GO:0006139 | L | BP | nucleobase-containing compound metabolic process |
| 0.518 | GO:0023052 | L | BP | signaling |
| 0.513 | GO:0005102 | L | MF | receptor binding |

**Protein Name: UdgX**

**Predicted results:**

**SVMProt**

| **Protein Family Name** | **GO Category** | **SVM** | **PNN** | **KNN** |
| --- | --- | --- | --- | --- |
| **Molecular Function** | | | | |
| EC2.7 Transferases - Transferring Phosphorus-Containing Groups | - | 78.4 | - | Y |
| Zinc-binding | GO:0008270 zinc binding | 96.4 | - | - |
| Manganese-binding | GO:0030145 manganese binding | 89.3 | ? | ? |
| EC5.3 Isomerases - Intramolecular Oxidoreductases | - | 58.6 | - | Y |
| EC2.1 Transferases - Transferring One-Carbon Groups | - | 58.6 | - | Y |
| Iron-binding | GO:0005506 iron binding | 73.8 | - | - |
| Magnesium-binding | GO:0000287 magnesium binding | 58.6 | ? | ? |
| **Biological Process** | | | | |
| DNA repair | GO:0006281 DNA repair | 58.6 | ? | ? |

**FFPred**

| **Score** | **GO term** | **RL** | **Domain** | **Description** |
| --- | --- | --- | --- | --- |
| 0.974 | GO:0016740 | H | MF | transferase activity |
| 0.942 | GO:0003824 | H | MF | catalytic activity |
| 0.886 | GO:0046914 | H | MF | transition metal ion binding |
| 0.864 | GO:0009116 | H | BP | nucleoside metabolic process |
| 0.821 | GO:0030554 | H | MF | adenyl nucleotide binding |
| 0.815 | GO:0017076 | H | MF | purine nucleotide binding |
| 0.798 | GO:0003676 | H | MF | nucleic acid binding |
| 0.797 | GO:0044281 | H | BP | small molecule metabolic process |
| 0.788 | GO:0035639 | H | MF | purine ribonucleoside triphosphate binding |
| 0.783 | GO:0000166 | H | MF | nucleotide binding |
| 0.782 | GO:0001882 | H | MF | nucleoside binding |
| 0.778 | GO:0032549 | H | MF | ribonucleoside binding |
| 0.778 | GO:0009056 | H | BP | catabolic process |
| 0.76 | GO:0006796 | H | BP | phosphate-containing compound metabolic process |
| 0.737 | GO:0019222 | H | BP | regulation of metabolic process |
| 0.722 | GO:0001883 | H | MF | purine nucleoside binding |
| 0.71 | GO:0005739 | H | CC | mitochondrion |
| 0.702 | GO:0055086 | H | BP | nucleobase-containing small molecule metabolic process |
| 0.7 | GO:0019637 | H | BP | organophosphate metabolic process |
| 0.697 | GO:0009117 | H | BP | nucleotide metabolic process |
| 0.693 | GO:0070062 | H | CC | extracellular vesicular exosome |
| 0.666 | GO:0005524 | H | MF | ATP binding |
| 0.666 | GO:0010468 | H | BP | regulation of gene expression |
| 0.65 | GO:0051171 | H | BP | regulation of nitrogen compound metabolic process |
| 0.648 | GO:0009059 | H | BP | macromolecule biosynthetic process |
| 0.632 | GO:2001141 | H | BP | regulation of RNA biosynthetic process |
| 0.628 | GO:1903506 | H | BP | regulation of nucleic acid-templated transcription |
| 0.627 | GO:0016491 | H | MF | oxidoreductase activity |
| 0.62 | GO:0031982 | H | CC | vesicle |
| 0.596 | GO:0016773 | H | MF | phosphotransferase activity, alcohol group as acceptor |
| 0.586 | GO:0031988 | H | CC | membrane-bounded vesicle |
| 0.581 | GO:0051252 | H | BP | regulation of RNA metabolic process |
| 0.568 | GO:0006810 | H | BP | transport |
| 0.567 | GO:0006355 | H | BP | regulation of transcription, DNA-templated |
| 0.567 | GO:0016747 | H | MF | transferase activity, transferring acyl groups other than amino-acyl groups |
| 0.563 | GO:0006790 | H | BP | sulfur compound metabolic process |
| 0.56 | GO:0006811 | H | BP | ion transport |
| 0.543 | GO:0006082 | H | BP | organic acid metabolic process |
| 0.536 | GO:0055114 | H | BP | oxidation-reduction process |
| 0.532 | GO:0006163 | H | BP | purine nucleotide metabolic process |
| 0.529 | GO:0005576 | H | CC | extracellular region |
| 0.528 | GO:0016874 | H | MF | ligase activity |
| 0.527 | GO:0016310 | H | BP | phosphorylation |
| 0.522 | GO:0009259 | H | BP | ribonucleotide metabolic process |
| 0.52 | GO:0034645 | H | BP | cellular macromolecule biosynthetic process |
| 0.511 | GO:0016817 | H | MF | hydrolase activity, acting on acid anhydrides |
| 0.506 | GO:0008092 | H | MF | cytoskeletal protein binding |
| 0.973 | GO:0006139 | L | BP | nucleobase-containing compound metabolic process |
| 0.971 | GO:0006807 | L | BP | nitrogen compound metabolic process |
| 0.942 | GO:0008152 | L | BP | metabolic process |
| 0.933 | GO:0005737 | L | CC | cytoplasm |
| 0.932 | GO:0043229 | L | CC | intracellular organelle |
| 0.926 | GO:0044237 | L | BP | cellular metabolic process |
| 0.914 | GO:0019538 | L | BP | protein metabolic process |
| 0.883 | GO:0043231 | L | CC | intracellular membrane-bounded organelle |
| 0.863 | GO:0097159 | L | MF | organic cyclic compound binding |
| 0.861 | GO:0006464 | L | BP | cellular protein modification process |
| 0.857 | GO:0050896 | L | BP | response to stimulus |
| 0.854 | GO:0009058 | L | BP | biosynthetic process |
| 0.852 | GO:0036094 | L | MF | small molecule binding |
| 0.851 | GO:0044267 | L | BP | cellular protein metabolic process |
| 0.838 | GO:0005634 | L | CC | nucleus |
| 0.824 | GO:0051716 | L | BP | cellular response to stimulus |
| 0.823 | GO:0034641 | L | BP | cellular nitrogen compound metabolic process |
| 0.813 | GO:0046483 | L | BP | heterocycle metabolic process |
| 0.806 | GO:0023052 | L | BP | signaling |
| 0.796 | GO:0031981 | L | CC | nuclear lumen |
| 0.792 | GO:0006725 | L | BP | cellular aromatic compound metabolic process |
| 0.788 | GO:0005829 | L | CC | cytosol |
| 0.783 | GO:0043169 | L | MF | cation binding |
| 0.766 | GO:0007165 | L | BP | signal transduction |
| 0.723 | GO:0005654 | L | CC | nucleoplasm |
| 0.699 | GO:0032502 | L | BP | developmental process |
| 0.693 | GO:0010467 | L | BP | gene expression |
| 0.687 | GO:0046872 | L | MF | metal ion binding |
| 0.674 | GO:0032991 | L | CC | macromolecular complex |
| 0.66 | GO:0006996 | L | BP | organelle organization |
| 0.658 | GO:0016070 | L | BP | RNA metabolic process |
| 0.654 | GO:0005102 | L | MF | receptor binding |
| 0.64 | GO:0009893 | L | BP | positive regulation of metabolic process |
| 0.634 | GO:0016787 | L | MF | hydrolase activity |
| 0.627 | GO:0007275 | L | BP | multicellular organismal development |
| 0.59 | GO:0043234 | L | CC | protein complex |
| 0.589 | GO:0048856 | L | BP | anatomical structure development |
| 0.577 | GO:0050790 | L | BP | regulation of catalytic activity |
| 0.56 | GO:0007154 | L | BP | cell communication |
| 0.556 | GO:0016772 | L | MF | transferase activity, transferring phosphorus-containing groups |
| 0.528 | GO:0009966 | L | BP | regulation of signal transduction |
| 0.505 | GO:0031325 | L | BP | positive regulation of cellular metabolic process |
| 0.504 | GO:0010033 | L | BP | response to organic substance |

**Protein Name: NCBI-XP_414028**

**Predicted results:**

**SVMProt**

| **Protein Family Name** | **GO Category** | **SVM** | **PNN** | **KNN** |
| --- | --- | --- | --- | --- |
| **Molecular Function** | | | | |
| EC4.2 Lyases - Carbon-Oxygen Lyases | - | 76.2 | - | Y |
| All lipid-binding proteins | GO:0008289 lipid binding | 96.1 | - | - |
| EC2.6 Transferases - Transferring Nitrogenous Groups | - | 96.1 | - | - |
| EC1.3 Oxidoreductases - Acting on the CH-CH group of donors | - | 86.8 | ? | ? |
| EC1.1 Oxidoreductases - Acting on the CH-OH group of donors | - | 86.8 | - | - |
| EC1.5 Oxidoreductases - Acting on the CH-NH group of donors | - | 85.4 | ? | ? |
| Metal-binding | GO:0046872 metal ion binding | 76.2 | - | - |
| Zinc-binding | GO:0008270 zinc binding | 73.8 | - | - |
| EC1.6 Oxidoreductases - Acting on NADH or NADPH | - | 73.8 | - | - |
| EC1.2 Oxidoreductases - Acting on the aldehyde or oxo group of donors | - | 65.4 | - | - |
| TC3.A.1 ATP-binding cassette (ABC) family | - | 58.6 | ? | ? |
| **Biological Process** | | | | |
| Chlorophyll biosynthesis | GO:0015995 chlorophyll biosynthetic process | 58.6 | ? | ? |
| DNA repair | GO:0006281 DNA repair | 58.6 | ? | ? |

**FFPred**

| **Score** | **GO term** | **RL** | **Domain** | **Description** |
| --- | --- | --- | --- | --- |
| 0.984 | GO:0003824 | H | MF | catalytic activity |
| 0.974 | GO:0044281 | H | BP | small molecule metabolic process |
| 0.941 | GO:0016740 | H | MF | transferase activity |
| 0.899 | GO:0006082 | H | BP | organic acid metabolic process |
| 0.895 | GO:0019752 | H | BP | carboxylic acid metabolic process |
| 0.877 | GO:0009056 | H | BP | catabolic process |
| 0.865 | GO:0006796 | H | BP | phosphate-containing compound metabolic process |
| 0.862 | GO:0005739 | H | CC | mitochondrion |
| 0.852 | GO:1901605 | H | BP | alpha-amino acid metabolic process |
| 0.841 | GO:0030554 | H | MF | adenyl nucleotide binding |
| 0.839 | GO:0000166 | H | MF | nucleotide binding |
| 0.834 | GO:0005524 | H | MF | ATP binding |
| 0.834 | GO:0035639 | H | MF | purine ribonucleoside triphosphate binding |
| 0.832 | GO:0032549 | H | MF | ribonucleoside binding |
| 0.816 | GO:0017076 | H | MF | purine nucleotide binding |
| 0.808 | GO:0019222 | H | BP | regulation of metabolic process |
| 0.798 | GO:0001883 | H | MF | purine nucleoside binding |
| 0.76 | GO:0019637 | H | BP | organophosphate metabolic process |
| 0.754 | GO:0001882 | H | MF | nucleoside binding |
| 0.732 | GO:0010468 | H | BP | regulation of gene expression |
| 0.723 | GO:0016301 | H | MF | kinase activity |
| 0.713 | GO:0005576 | H | CC | extracellular region |
| 0.712 | GO:0046395 | H | BP | carboxylic acid catabolic process |
| 0.709 | GO:0055114 | H | BP | oxidation-reduction process |
| 0.704 | GO:0005975 | H | BP | carbohydrate metabolic process |
| 0.699 | GO:0016788 | H | MF | hydrolase activity, acting on ester bonds |
| 0.681 | GO:0008270 | H | MF | zinc ion binding |
| 0.678 | GO:0009117 | H | BP | nucleotide metabolic process |
| 0.674 | GO:0006629 | H | BP | lipid metabolic process |
| 0.655 | GO:2001141 | H | BP | regulation of RNA biosynthetic process |
| 0.653 | GO:0016311 | H | BP | dephosphorylation |
| 0.645 | GO:0003676 | H | MF | nucleic acid binding |
| 0.641 | GO:0008233 | H | MF | peptidase activity |
| 0.626 | GO:1903506 | H | BP | regulation of nucleic acid-templated transcription |
| 0.624 | GO:0051171 | H | BP | regulation of nitrogen compound metabolic process |
| 0.615 | GO:0016310 | H | BP | phosphorylation |
| 0.613 | GO:0031988 | H | CC | membrane-bounded vesicle |
| 0.609 | GO:0016817 | H | MF | hydrolase activity, acting on acid anhydrides |
| 0.605 | GO:0016773 | H | MF | phosphotransferase activity, alcohol group as acceptor |
| 0.595 | GO:0006355 | H | BP | regulation of transcription, DNA-templated |
| 0.595 | GO:0070062 | H | CC | extracellular vesicular exosome |
| 0.593 | GO:0006520 | H | BP | cellular amino acid metabolic process |
| 0.592 | GO:0055086 | H | BP | nucleobase-containing small molecule metabolic process |
| 0.588 | GO:0006811 | H | BP | ion transport |
| 0.586 | GO:0045184 | H | BP | establishment of protein localization |
| 0.575 | GO:0006508 | H | BP | proteolysis |
| 0.574 | GO:0051252 | H | BP | regulation of RNA metabolic process |
| 0.572 | GO:0031982 | H | CC | vesicle |
| 0.567 | GO:0016818 | H | MF | hydrolase activity, acting on acid anhydrides, in phosphorus-containing anhydrides |
| 0.566 | GO:0017111 | H | MF | nucleoside-triphosphatase activity |
| 0.523 | GO:0044255 | H | BP | cellular lipid metabolic process |
| 0.517 | GO:0032787 | H | BP | monocarboxylic acid metabolic process |
| 0.515 | GO:0034645 | H | BP | cellular macromolecule biosynthetic process |
| 0.509 | GO:0016491 | H | MF | oxidoreductase activity |
| 0.505 | GO:0008092 | H | MF | cytoskeletal protein binding |
| 0.968 | GO:0008152 | L | BP | metabolic process |
| 0.952 | GO:0019538 | L | BP | protein metabolic process |
| 0.951 | GO:0044237 | L | BP | cellular metabolic process |
| 0.927 | GO:0009058 | L | BP | biosynthetic process |
| 0.921 | GO:0043229 | L | CC | intracellular organelle |
| 0.896 | GO:0005737 | L | CC | cytoplasm |
| 0.883 | GO:0043231 | L | CC | intracellular membrane-bounded organelle |
| 0.879 | GO:0006464 | L | BP | cellular protein modification process |
| 0.866 | GO:0050896 | L | BP | response to stimulus |
| 0.851 | GO:0044267 | L | BP | cellular protein metabolic process |
| 0.839 | GO:0036094 | L | MF | small molecule binding |
| 0.828 | GO:0051716 | L | BP | cellular response to stimulus |
| 0.803 | GO:0043169 | L | MF | cation binding |
| 0.793 | GO:0005634 | L | CC | nucleus |
| 0.783 | GO:0016787 | L | MF | hydrolase activity |
| 0.782 | GO:0034641 | L | BP | cellular nitrogen compound metabolic process |
| 0.776 | GO:0046483 | L | BP | heterocycle metabolic process |
| 0.756 | GO:0046872 | L | MF | metal ion binding |
| 0.751 | GO:0006725 | L | BP | cellular aromatic compound metabolic process |
| 0.75 | GO:0016772 | L | MF | transferase activity, transferring phosphorus-containing groups |
| 0.746 | GO:0097159 | L | MF | organic cyclic compound binding |
| 0.71 | GO:0007154 | L | BP | cell communication |
| 0.707 | GO:0032502 | L | BP | developmental process |
| 0.702 | GO:0031981 | L | CC | nuclear lumen |
| 0.697 | GO:0006807 | L | BP | nitrogen compound metabolic process |
| 0.695 | GO:0050790 | L | BP | regulation of catalytic activity |
| 0.674 | GO:0005829 | L | CC | cytosol |
| 0.669 | GO:0006996 | L | BP | organelle organization |
| 0.655 | GO:0009893 | L | BP | positive regulation of metabolic process |
| 0.645 | GO:0005654 | L | CC | nucleoplasm |
| 0.621 | GO:0010467 | L | BP | gene expression |
| 0.619 | GO:0007275 | L | BP | multicellular organismal development |
| 0.581 | GO:0016070 | L | BP | RNA metabolic process |
| 0.506 | GO:0006139 | L | BP | nucleobase-containing compound metabolic process |
| 0.5 | GO:0032991 | L | CC | macromolecular complex |

**Blast**

| **BLAST Matched UniProt ID & name** | | | **Identity** | **E Value** | **Score** |
| --- | --- | --- | --- | --- | --- |
| Q7Z9I2 | YCP9_SCHPO | Uncharacterized oxidoreductase C663.09c | 30.566 | 2.00E-26 | 108 |
| Q7Z9I4 | YCP6_SCHPO | Uncharacterized oxidoreductase C663.06c | 29.74 | 2.61E-24 | 102 |
| P21158 | CSGA_MYXXA | C-factor | 35.088 | 6.99E-24 | 99 |
| Q7Z9I3 | YCP8_SCHPO | Uncharacterized oxidoreductase C663.08c | 28.679 | 6.39E-22 | 95.5 |
| Q9P7I6 | YJNK_SCHPO | Uncharacterized oxidoreductase C24B10.20 | 31.461 | 1.03E-20 | 92 |
| P36086 | YKH1_YEAST | Uncharacterized oxidoreductase YKL071W | 28.517 | 8.86E-18 | 83.6 |
| P05406 | FIXR_BRADU | Protein FixR | 30.317 | 1.36E-12 | 68.9 |
| Q00278 | AFLD_ASPPA | Norsolorinic acid ketoreductase | 25.67 | 4.28E-10 | 62 |
| P71534 | FABG_MYCS2 | 3-oxoacyl-[acyl-carrier-protein] reductase FabG | 26.939 | 1.15E-09 | 60.5 |
| Q8K354 | CBR3_MOUSE | Carbonyl reductase [NADPH] 3 | 27.953 | 1.17E-09 | 60.8 |
| O07399 | FABG_MYCAV | 3-oxoacyl-[acyl-carrier-protein] reductase FabG | 28.251 | 2.66E-09 | 59.7 |
| Q5R6U1 | DRS7B_PONAB | Dehydrogenase/reductase SDR family member 7B | 26.941 | 4.44E-09 | 59.3 |
| P37694 | HETN_NOSS1 | Ketoacyl reductase HetN | 27.556 | 1.21E-08 | 58.2 |
| A4UHT7 | SALR_PAPBR | Salutaridine reductase | 24.127 | 1.25E-08 | 58.2 |
| Q6IAN0 | DRS7B_HUMAN | Dehydrogenase/reductase SDR family member 7B | 26.484 | 1.38E-08 | 58.2 |
| Q02337 | BDH_BOVIN | D-beta-hydroxybutyrate dehydrogenase, mitochondrial | 25.481 | 2.62E-08 | 57.4 |
| Q3SZD7 | CBR1_BOVIN | Carbonyl reductase [NADPH] 1 | 27.143 | 2.92E-08 | 56.6 |
| P29147 | BDH_RAT | D-beta-hydroxybutyrate dehydrogenase, mitochondrial | 28.049 | 4.45E-08 | 56.6 |
| Q9ZNN8 | BUDC_CORGT | L-2,3-butanediol dehydrogenase | 29.032 | 6.50E-08 | 55.5 |
| Q27979 | RDH1_BOVIN | 11-cis retinol dehydrogenase | 28.899 | 8.96E-08 | 55.5 |
| Q5AV81 | Y7799_EMENI | Uncharacterized oxidoreductase AN7799 | 27.381 | 9.30E-08 | 55.1 |
| O93868 | MTDH_AGABI | NADP-dependent mannitol dehydrogenase | 25 | 1.27E-07 | 54.7 |
| Q99J47 | DRS7B_MOUSE | Dehydrogenase/reductase SDR family member 7B | 27.922 | 1.35E-07 | 55.1 |
| P47844 | CBR1_RABIT | Carbonyl reductase [NADPH] 1 | 27.419 | 1.36E-07 | 54.7 |
| Q80XN0 | BDH_MOUSE | D-beta-hydroxybutyrate dehydrogenase, mitochondrial | 24.519 | 1.47E-07 | 55.1 |
| Q7Z4W1 | DCXR_HUMAN | L-xylulose reductase | 27.82 | 1.54E-07 | 54.3 |
| O14351 | YB45_SCHPO | Uncharacterized oxidoreductase C30D10.05c | 27.426 | 1.84E-07 | 54.3 |
| P50199 | GNO_GLUOX | Gluconate 5-dehydrogenase | 27.232 | 3.12E-07 | 53.5 |
| P0A5Y5 | FABG_MYCBO | 3-oxoacyl-[acyl-carrier-protein] reductase FabG | 27.315 | 3.23E-07 | 53.5 |
| Q28960 | CBR1_PIG | Carbonyl reductase [NADPH] 1 | 26.102 | 4.41E-07 | 53.5 |
| Q8NEX9 | DR9C7_HUMAN | Short-chain dehydrogenase/reductase family 9C member 7 | 30.952 | 6.12E-07 | 53.1 |
| Q3T0R4 | DRS7B_BOVIN | Dehydrogenase/reductase SDR family member 7B | 24.891 | 9.58E-07 | 52.4 |
| Q92781 | RDH1_HUMAN | 11-cis retinol dehydrogenase | 35 | 1.20E-06 | 52 |
| P47727 | CBR1_RAT | Carbonyl reductase [NADPH] 1 | 26.786 | 1.31E-06 | 52 |
| P16152 | CBR1_HUMAN | Carbonyl reductase [NADPH] 1 | 27.018 | 1.41E-06 | 52 |
| Q5RCU5 | CBR1_PONAB | Carbonyl reductase [NADPH] 1 | 27.402 | 1.82E-06 | 51.6 |
| Q8MI29 | CBR1_MACFA | Carbonyl reductase [NADPH] 1 | 26.829 | 3.00E-06 | 50.8 |
| P51657 | DHB1_RAT | Estradiol 17-beta-dehydrogenase 1 | 32.061 | 3.20E-06 | 50.8 |
| A7DY56 | TRN1_COCOF | Tropinone reductase | 26.442 | 3.75E-06 | 50.4 |
| P51656 | DHB1_MOUSE | Estradiol 17-beta-dehydrogenase 1 | 26.606 | 4.69E-06 | 50.4 |
| Q02338 | BDH_HUMAN | D-beta-hydroxybutyrate dehydrogenase, mitochondrial | 27.439 | 5.47E-06 | 50.1 |
| Q0IH28 | DRS7B_XENLA | Dehydrogenase/reductase SDR family member 7B | 25 | 7.12E-06 | 49.7 |
| Q89AG9 | FABG_BUCBP | 3-oxoacyl-[acyl-carrier-protein] reductase FabG | 26.047 | 8.53E-06 | 49.3 |
| Q9BPW9 | DHRS9_HUMAN | Dehydrogenase/reductase SDR family member 9 | 25.943 | 9.03E-06 | 49.7 |
| Q5RJY4 | DRS7B_RAT | Dehydrogenase/reductase SDR family member 7B | 25.974 | 9.39E-06 | 49.3 |
| O55240 | RDH1_MOUSE | 11-cis retinol dehydrogenase | 32.5 | 1.03E-05 | 49.3 |
| O75828 | CBR3_HUMAN | Carbonyl reductase [NADPH] 3 | 29.461 | 1.04E-05 | 49.3 |
| Q9ZKW1 | VDLC_HELPJ | Probable short-chain type dehydrogenase/reductase VdlC | 25 | 1.07E-05 | 49.3 |
| P40580 | BZRD_YEAST | Benzil reductase | 26.389 | 1.64E-05 | 48.5 |
| Q8NBN7 | RDH13_HUMAN | Retinol dehydrogenase 13 | 29.778 | 2.05E-05 | 48.5 |
| Q6MGB5 | DHB8_RAT | Estradiol 17-beta-dehydrogenase 8 | 29.091 | 3.06E-05 | 47.8 |
| P14061 | DHB1_HUMAN | Estradiol 17-beta-dehydrogenase 1 | 29.771 | 3.36E-05 | 47.8 |
| P48758 | CBR1_MOUSE | Carbonyl reductase [NADPH] 1 | 25.41 | 3.97E-05 | 47.4 |
| Q96LJ7 | DHRS1_HUMAN | Dehydrogenase/reductase SDR family member 1 | 29.858 | 4.52E-05 | 47.4 |
| P50197 | LINC_SPHPI | 2,5-dichloro-2,5-cyclohexadiene-1,4-diol dehydrogenase | 25.909 | 4.91E-05 | 47 |
| Q8HYR6 | DHRS9_BOVIN | Dehydrogenase/reductase SDR family member 9 | 26.415 | 5.23E-05 | 47.4 |
| O05730 | VDLC_HELPY | Probable short-chain type dehydrogenase/reductase VdlC | 24.038 | 5.55E-05 | 47 |
| Q1WNP0 | DHB1_PANTR | Estradiol 17-beta-dehydrogenase 1 | 29.771 | 5.57E-05 | 47 |
| Q9HBH5 | RDH14_HUMAN | Retinol dehydrogenase 14 | 27.706 | 5.87E-05 | 47 |
| P39577 | DLTE_BACSU | Uncharacterized oxidoreductase DltE | 27.111 | 7.47E-05 | 46.6 |
| Q9ERI6 | RDH14_MOUSE | Retinol dehydrogenase 14 | 29.614 | 9.01E-05 | 46.6 |
| Q8CEE7 | RDH13_MOUSE | Retinol dehydrogenase 13 | 29.68 | 1.58E-04 | 45.8 |
| Q58NB6 | DHRS9_MOUSE | Dehydrogenase/reductase SDR family member 9 | 25.11 | 3.04E-04 | 44.7 |
| Q6WAU1 | IPIPR_MENPI | (-)-isopiperitenone reductase | 35.922 | 3.66E-04 | 44.7 |
| Q5SS80 | DHR13_MOUSE | Dehydrogenase/reductase SDR family member 13 | 27.556 | 5.29E-04 | 44.3 |
| Q5TJF5 | DHB8_CANFA | Estradiol 17-beta-dehydrogenase 8 | 29.6 | 0.002 | 42.4 |
| P9WGP8 | SADH_MYCTO | Putative oxidoreductase SadH | 26.984 | 0.002 | 42.4 |
| Q9EQ06 | DHB11_MOUSE | Estradiol 17-beta-dehydrogenase 11 | 24.215 | 0.002 | 42 |
| Q5ZJZ5 | BDH_CHICK | D-beta-hydroxybutyrate dehydrogenase, mitochondrial | 24.832 | 0.003 | 41.6 |
| Q5P5I4 | PED_AROAE | (S)-1-Phenylethanol dehydrogenase | 23.113 | 0.004 | 41.2 |
| Q51576 | Y3106_PSEAE | Uncharacterized oxidoreductase PA3106 | 29.299 | 0.005 | 40.8 |
| Q8VD48 | DHRS9_RAT | Dehydrogenase/reductase SDR family member 9 | 24.229 | 0.005 | 41.2 |
| Q92506 | DHB8_HUMAN | Estradiol 17-beta-dehydrogenase 8 | 28.8 | 0.005 | 40.8 |
| Q00674 | STCE_EMENI | Putative sterigmatocystin biosynthesis ketoreductase stcE | 25.651 | 0.011 | 40 |
| Q6AYS8 | DHB11_RAT | Estradiol 17-beta-dehydrogenase 11 | 22.87 | 0.032 | 38.5 |

**Protein Name: S-Inosyl-L-Homocysteine Hydrolase**

**Predicted results:**

**SVMProt**

| **Protein Family Name** | **GO Category** | **SVM** | **PNN** | **KNN** |
| --- | --- | --- | --- | --- |
| **Molecular Function** | | | | |
| EC2.7 Transferases - Transferring Phosphorus-Containing Groups | - | 96.1 | Y | Y |
| EC3.3 Hydrolases - Acting on Ether Bonds | - | 99 | ? | ? |
| Zinc-binding | GO:0008270 zinc binding | 99 | - | - |
| All DNA-binding | GO:0003677 DNA binding | 90.3 | ? | ? |
| All lipid-binding proteins | GO:0008289 lipid binding | 71.3 | - | - |
| EC4.1 Lyases - Carbon-Carbon Lyases | - | 71.3 | - | - |
| DNA-directed DNA polymerase | GO:0003887 DNA-directed DNA polymerase activity | 65.4 | ? | ? |
| TC3.A.5 Type II (general) secretory pathway (IISP) family | - | 58.6 | ? | ? |
| Magnesium-binding | GO:0000287 magnesium binding | 58.6 | ? | ? |
| **Biological Process** | | | | |
| DNA repair | GO:0006281 DNA repair | 58.6 | ? | ? |

**FFPred**

| **Score** | **GO term** | **RL** | **Domain** | **Description** |
| --- | --- | --- | --- | --- |
| 0.956 | GO:0005576 | H | CC | extracellular region |
| 0.943 | GO:0003824 | H | MF | catalytic activity |
| 0.922 | GO:0006082 | H | BP | organic acid metabolic process |
| 0.899 | GO:0044281 | H | BP | small molecule metabolic process |
| 0.877 | GO:0031982 | H | CC | vesicle |
| 0.86 | GO:0070062 | H | CC | extracellular vesicular exosome |
| 0.841 | GO:0017076 | H | MF | purine nucleotide binding |
| 0.818 | GO:0001882 | H | MF | nucleoside binding |
| 0.812 | GO:0005739 | H | CC | mitochondrion |
| 0.806 | GO:0000166 | H | MF | nucleotide binding |
| 0.8 | GO:0031988 | H | CC | membrane-bounded vesicle |
| 0.799 | GO:0009165 | H | BP | nucleotide biosynthetic process |
| 0.798 | GO:0001883 | H | MF | purine nucleoside binding |
| 0.782 | GO:0055086 | H | BP | nucleobase-containing small molecule metabolic process |
| 0.778 | GO:0006796 | H | BP | phosphate-containing compound metabolic process |
| 0.768 | GO:0019752 | H | BP | carboxylic acid metabolic process |
| 0.765 | GO:0032549 | H | MF | ribonucleoside binding |
| 0.758 | GO:0035639 | H | MF | purine ribonucleoside triphosphate binding |
| 0.744 | GO:0009056 | H | BP | catabolic process |
| 0.735 | GO:0006412 | H | BP | translation |
| 0.727 | GO:0006163 | H | BP | purine nucleotide metabolic process |
| 0.656 | GO:0009259 | H | BP | ribonucleotide metabolic process |
| 0.653 | GO:0030554 | H | MF | adenyl nucleotide binding |
| 0.645 | GO:0019222 | H | BP | regulation of metabolic process |
| 0.607 | GO:0042278 | H | BP | purine nucleoside metabolic process |
| 0.606 | GO:0009117 | H | BP | nucleotide metabolic process |
| 0.586 | GO:0055114 | H | BP | oxidation-reduction process |
| 0.585 | GO:0005524 | H | MF | ATP binding |
| 0.561 | GO:0009116 | H | BP | nucleoside metabolic process |
| 0.545 | GO:0016817 | H | MF | hydrolase activity, acting on acid anhydrides |
| 0.544 | GO:0044822 | H | MF | poly(A) RNA binding |
| 0.539 | GO:0016462 | H | MF | pyrophosphatase activity |
| 0.511 | GO:0016491 | H | MF | oxidoreductase activity |
| 0.508 | GO:0005975 | H | BP | carbohydrate metabolic process |
| 0.505 | GO:0016020 | H | CC | membrane |
| 0.965 | GO:0005737 | L | CC | cytoplasm |
| 0.943 | GO:0044237 | L | BP | cellular metabolic process |
| 0.942 | GO:0006139 | L | BP | nucleobase-containing compound metabolic process |
| 0.934 | GO:0006807 | L | BP | nitrogen compound metabolic process |
| 0.921 | GO:0097159 | L | MF | organic cyclic compound binding |
| 0.902 | GO:0036094 | L | MF | small molecule binding |
| 0.9 | GO:0008152 | L | BP | metabolic process |
| 0.875 | GO:0009058 | L | BP | biosynthetic process |
| 0.862 | GO:0050896 | L | BP | response to stimulus |
| 0.831 | GO:0043169 | L | MF | cation binding |
| 0.83 | GO:0051716 | L | BP | cellular response to stimulus |
| 0.811 | GO:0032502 | L | BP | developmental process |
| 0.79 | GO:0034641 | L | BP | cellular nitrogen compound metabolic process |
| 0.787 | GO:0005829 | L | CC | cytosol |
| 0.784 | GO:0005634 | L | CC | nucleus |
| 0.776 | GO:0044267 | L | BP | cellular protein metabolic process |
| 0.761 | GO:0046872 | L | MF | metal ion binding |
| 0.747 | GO:0046483 | L | BP | heterocycle metabolic process |
| 0.743 | GO:0006725 | L | BP | cellular aromatic compound metabolic process |
| 0.734 | GO:0031981 | L | CC | nuclear lumen |
| 0.701 | GO:0007154 | L | BP | cell communication |
| 0.687 | GO:0006996 | L | BP | organelle organization |
| 0.65 | GO:0005654 | L | CC | nucleoplasm |
| 0.646 | GO:0043231 | L | CC | intracellular membrane-bounded organelle |
| 0.643 | GO:0019538 | L | BP | protein metabolic process |
| 0.602 | GO:0032991 | L | CC | macromolecular complex |
| 0.602 | GO:0048856 | L | BP | anatomical structure development |
| 0.599 | GO:0010467 | L | BP | gene expression |
| 0.598 | GO:0007165 | L | BP | signal transduction |
| 0.587 | GO:0007275 | L | BP | multicellular organismal development |
| 0.57 | GO:0006464 | L | BP | cellular protein modification process |
| 0.549 | GO:0010033 | L | BP | response to organic substance |
| 0.543 | GO:0031410 | L | CC | cytoplasmic vesicle |
| 0.535 | GO:0032403 | L | MF | protein complex binding |
| 0.534 | GO:0043229 | L | CC | intracellular organelle |
| 0.51 | GO:0043234 | L | CC | protein complex |
| 0.508 | GO:0009893 | L | BP | positive regulation of metabolic process |
| 0.508 | GO:0016070 | L | BP | RNA metabolic process |
| 0.504 | GO:0071310 | L | BP | cellular response to organic substance |

**Blast**

| **BLAST Matched UniProt ID & name** | | | **Identity** | **E Value** | **Score** |
| --- | --- | --- | --- | --- | --- |
| Q6LYR8 | SAHH_METMP | Adenosylhomocysteinase | 75.663 | 0 | 665 |
| O28279 | SAHH_ARCFU | Adenosylhomocysteinase | 67.327 | 0 | 571 |
| P58855 | SAHH_METKA | Adenosylhomocysteinase | 62.26 | 0 | 546 |
| Q9UYK5 | SAHH_PYRAB | Adenosylhomocysteinase | 63.855 | 0.00E+00 | 545 |
| O27673 | SAHH_METTH | Adenosylhomocysteinase | 62.83 | 0.00E+00 | 545 |
| O58275 | SAHH_PYRHO | Adenosylhomocysteinase | 64.096 | 0.00E+00 | 538 |
| P50251 | SAHH_PYRFU | Adenosylhomocysteinase | 63.614 | 0.00E+00 | 538 |
| P50252 | SAHH_SULSO | Adenosylhomocysteinase | 60.577 | 0.00E+00 | 531 |
| Q8PUQ4 | SAHH_METMA | Adenosylhomocysteinase | 62.255 | 0.00E+00 | 523 |
| Q8TRA5 | SAHH_METAC | Adenosylhomocysteinase | 62.5 | 0.00E+00 | 521 |
| Q5JED2 | SAHH_THEKO | Adenosylhomocysteinase | 62.892 | 0.00E+00 | 521 |
| O67240 | SAHH_AQUAE | Adenosylhomocysteinase | 60.337 | 2.14E-180 | 516 |
| Q975T0 | SAHH_SULTO | Adenosylhomocysteinase | 58.795 | 2.45E-180 | 516 |
| Q4JAZ7 | SAHH_SULAC | Adenosylhomocysteinase | 59.277 | 2.91E-180 | 516 |
| Q9YEF2 | SAHH_AERPE | Adenosylhomocysteinase | 56.796 | 1.17E-165 | 478 |
| O51933 | SAHH_THEMA | Adenosylhomocysteinase | 57.357 | 1.27E-164 | 475 |
| P74008 | SAHH_SYNY3 | Adenosylhomocysteinase | 58.413 | 1.76E-164 | 476 |
| Q979Z4 | SAHH_THEVO | Adenosylhomocysteinase | 56.65 | 7.90E-164 | 473 |
| Q9HKX4 | SAHH_THEAC | Adenosylhomocysteinase | 55.774 | 5.42E-163 | 471 |
| B7K8X6 | SAHH_CYAP7 | Adenosylhomocysteinase | 57.692 | 5.27E-161 | 467 |
| Q8ZTQ7 | SAHH_PYRAE | Adenosylhomocysteinase | 54.378 | 2.34E-160 | 466 |
| Q7NGI6 | SAHH_GLOVI | Adenosylhomocysteinase | 56.25 | 2.70E-159 | 462 |
| Q8DGC8 | SAHH_THEEB | Adenosylhomocysteinase | 56.01 | 3.08E-159 | 462 |
| Q8YX05 | SAHH_NOSS1 | Adenosylhomocysteinase | 55.048 | 2.17E-153 | 447 |
| Q18EV6 | SAHH_HALWD | Adenosylhomocysteinase | 53.676 | 4.70E-148 | 434 |
| Q9HN50 | SAHH_HALSA | Adenosylhomocysteinase | 53.317 | 1.18E-146 | 430 |
| G0LFB0 | SAHH_HALWC | Adenosylhomocysteinase | 53.186 | 1.26E-146 | 431 |
| O93477 | SAHHB_XENLA | Adenosylhomocysteinase B | 45.215 | 1.09E-113 | 346 |
| P51893 | SAHHA_XENLA | Adenosylhomocysteinase A | 45.215 | 4.74E-113 | 345 |
| B6J6H1 | SAHH_COXB1 | Adenosylhomocysteinase | 45.146 | 4.70E-111 | 340 |
| A9KD88 | SAHH_COXBN | Adenosylhomocysteinase | 45.146 | 5.83E-111 | 339 |
| Q83A77 | SAHH_COXBU | Adenosylhomocysteinase | 45.146 | 1.19E-110 | 338 |
| Q3MHL4 | SAHH_BOVIN | Adenosylhomocysteinase | 45.564 | 2.02E-109 | 335 |
| O13639 | SAHH_SCHPO | Adenosylhomocysteinase | 45.036 | 2.51E-109 | 335 |
| A1WXM7 | SAHH_HALHL | Adenosylhomocysteinase | 45.146 | 3.88E-109 | 334 |
| Q710C4 | SAHH_PIG | Adenosylhomocysteinase | 45.803 | 6.84E-109 | 334 |
| A0M5W6 | SAHH_GRAFK | Adenosylhomocysteinase | 45.564 | 7.78E-109 | 334 |
| Q4R596 | SAHH_MACFA | Adenosylhomocysteinase | 45.084 | 2.08E-108 | 333 |
| Q7TTZ5 | SAHH_RHOBA | Adenosylhomocysteinase | 43.972 | 2.99E-108 | 333 |
| Q27580 | SAHH_DROME | Adenosylhomocysteinase | 46.117 | 3.48E-108 | 332 |
| P23526 | SAHH_HUMAN | Adenosylhomocysteinase | 45.499 | 4.81E-108 | 332 |
| O76757 | SAHH_ANOGA | Adenosylhomocysteinase | 45.933 | 2.94E-107 | 330 |
| P39954 | SAHH_YEAST | Adenosylhomocysteinase | 42.956 | 1.29E-106 | 328 |
| P50247 | SAHH_MOUSE | Adenosylhomocysteinase | 45.012 | 2.62E-106 | 327 |
| P10760 | SAHH_RAT | Adenosylhomocysteinase | 45.012 | 8.56E-106 | 326 |
| P10819 | SAHH_DICDI | Adenosylhomocysteinase | 45.036 | 1.45E-105 | 325 |
| P27604 | SAHH_CAEEL | Adenosylhomocysteinase | 43.373 | 2.00E-104 | 322 |
| Q6MNC0 | SAHH_BDEBA | Adenosylhomocysteinase | 44.068 | 2.32E-104 | 323 |
| Q04NN6 | SAHH_LEPBJ | Adenosylhomocysteinase | 44.175 | 4.50E-104 | 322 |
| Q8EXV1 | SAHH_LEPIN | Adenosylhomocysteinase | 43.932 | 1.15E-103 | 320 |
| A8G7D1 | SAHH_PROM2 | Adenosylhomocysteinase | 41.372 | 2.70E-103 | 321 |
| Q318B6 | SAHH_PROM9 | Adenosylhomocysteinase | 41.814 | 6.60E-103 | 320 |
| A3PFB5 | SAHH_PROM0 | Adenosylhomocysteinase | 41.372 | 8.53E-103 | 320 |
| B3EDY3 | SAHH_CHLL2 | Adenosylhomocysteinase | 41.871 | 1.34E-102 | 319 |
| A4SF77 | SAHH_PROVI | Adenosylhomocysteinase | 41.203 | 1.42E-102 | 319 |
| A9C184 | SAHH_DELAS | Adenosylhomocysteinase | 41.228 | 1.76E-101 | 316 |
| Q3A392 | SAHH_PELCD | Adenosylhomocysteinase | 41.685 | 2.05E-101 | 316 |
| A5FJK3 | SAHH_FLAJ1 | Adenosylhomocysteinase | 42.373 | 2.58E-101 | 315 |
| Q3ANF4 | SAHH_SYNSC | Adenosylhomocysteinase | 42.129 | 4.19E-101 | 315 |
| P61617 | SAHH_GEOSL | Adenosylhomocysteinase | 42.058 | 8.18E-101 | 315 |
| B1Y647 | SAHH_LEPCP | Adenosylhomocysteinase | 41.372 | 1.01E-100 | 314 |
| Q30WL8 | SAHH_DESAG | Adenosylhomocysteinase | 41.425 | 1.03E-100 | 314 |
| P83783 | SAHH_CANAL | Adenosylhomocysteinase | 41.935 | 1.08E-100 | 313 |
| Q3B532 | SAHH_PELLD | Adenosylhomocysteinase | 41.871 | 1.46E-100 | 313 |
| Q7UZN3 | SAHH_PROMP | Adenosylhomocysteinase | 40.044 | 1.73E-100 | 313 |
| Q8FRJ4 | SAHH_COREF | Adenosylhomocysteinase | 39.825 | 2.84E-100 | 313 |
| A6GW32 | SAHH_FLAPJ | Adenosylhomocysteinase | 42.373 | 4.55E-100 | 311 |
| A2C620 | SAHH_PROM3 | Adenosylhomocysteinase | 40.839 | 8.36E-100 | 312 |
| Q8KEG8 | SAHH_CHLTE | Adenosylhomocysteinase | 41.425 | 1.31E-99 | 311 |
| B2JIP4 | SAHH_BURP8 | Adenosylhomocysteinase | 41.163 | 1.83E-99 | 311 |
| Q1CY84 | SAHH_MYXXD | Adenosylhomocysteinase | 40.044 | 1.89E-99 | 311 |
| Q7V926 | SAHH_PROMM | Adenosylhomocysteinase | 40.397 | 2.47E-99 | 311 |
| A5GI30 | SAHH_SYNPW | Adenosylhomocysteinase | 41.501 | 3.48E-99 | 310 |
| B3QMF5 | SAHH_CHLP8 | Adenosylhomocysteinase | 40.757 | 4.34E-99 | 310 |
| B2U774 | SAHH_RALPJ | Adenosylhomocysteinase | 40.919 | 9.40E-99 | 309 |
| Q9PEJ1 | SAHH_XYLFA | Adenosylhomocysteinase | 39.514 | 1.37E-98 | 309 |
| A9BD69 | SAHH_PROM4 | Adenosylhomocysteinase | 40.045 | 2.02E-98 | 308 |
| Q7NZF7 | SAHH_CHRVO | Adenosylhomocysteinase | 40.177 | 3.82E-98 | 307 |
| A6T2Y9 | SAHH_JANMA | Adenosylhomocysteinase | 40.708 | 4.60E-98 | 307 |
| A4QC87 | SAHH_CORGB | Adenosylhomocysteinase | 38.562 | 5.42E-98 | 307 |
| Q8NSC4 | SAHH_CORGL | Adenosylhomocysteinase | 38.78 | 5.57E-98 | 307 |
| Q0AEV8 | SAHH_NITEC | Adenosylhomocysteinase | 40.402 | 6.94E-98 | 307 |
| Q87EI8 | SAHH_XYLFT | Adenosylhomocysteinase | 39.294 | 7.27E-98 | 307 |
| Q3B0K7 | SAHH_SYNS9 | Adenosylhomocysteinase | 41.242 | 1.15E-97 | 306 |
| Q0IDX7 | SAHH_SYNS3 | Adenosylhomocysteinase | 40.839 | 1.22E-97 | 306 |
| Q8Y387 | SAHH_RALSO | Adenosylhomocysteinase | 40.919 | 1.36E-97 | 306 |
| Q82WL1 | SAHH_NITEU | Adenosylhomocysteinase | 40.402 | 1.60E-97 | 306 |
| B2AGG2 | SAHH_CUPTR | Adenosylhomocysteinase | 40.659 | 1.92E-97 | 306 |
| B0U232 | SAHH_XYLFM | Adenosylhomocysteinase | 39.294 | 2.01E-97 | 306 |
| Q92TC1 | SAHH_RHIME | Adenosylhomocysteinase | 40.929 | 2.68E-97 | 305 |
| Q01VU1 | SAHH_SOLUE | Adenosylhomocysteinase | 39.062 | 3.01E-97 | 305 |
| Q1LS20 | SAHH_RALME | Adenosylhomocysteinase | 40.659 | 3.12E-97 | 305 |
| Q3AQC2 | SAHH_CHLCH | Adenosylhomocysteinase | 41.203 | 3.20E-97 | 305 |
| Q82DC9 | SAHH_STRAW | Adenosylhomocysteinase | 38.612 | 3.26E-97 | 305 |
| B1VUW6 | SAHH_STRGG | Adenosylhomocysteinase | 39.046 | 3.33E-97 | 305 |
| P61456 | SAHH_CORDI | Adenosylhomocysteinase | 39.13 | 3.42E-97 | 305 |
| Q9KZM1 | SAHH_STRCO | Adenosylhomocysteinase | 38.612 | 3.44E-97 | 305 |
| Q0KF25 | SAHH_CUPNH | Adenosylhomocysteinase | 40 | 4.03E-97 | 305 |
| Q7V9P3 | SAHH_PROMA | Adenosylhomocysteinase | 40.177 | 4.10E-97 | 305 |

**Protein Name: E3 UFM1-protein ligase 1**

**Predicted results:**

**SVMProt**

| **Protein Family Name** | **GO Category** | **SVM** | **PNN** | **KNN** |
| --- | --- | --- | --- | --- |
| **Molecular Function** | | | | |
| All lipid-binding proteins | GO:0008289 lipid binding | 95.7 | - | - |
| All DNA-binding | GO:0003677 DNA binding | 94.7 | ? | ? |
| Zinc-binding | GO:0008270 zinc binding | 88.1 | - | - |
| Actin binding | GO:0003779 actin binding | 58.6 | ? | ? |
| **Broadly Defined Function** | | | | |
| Photosystem I | GO:0009522 photosystem I | 58.6 | ? | ? |
| **Others** | | | | |
| Tumor suppressor | - | 98.8 | Y | Y |

**FFPred**

| **Score** | **GO term** | **RL** | **Domain** | **Description** |
| --- | --- | --- | --- | --- |
| 0.935 | GO:0003676 | H | MF | nucleic acid binding |
| 0.888 | GO:0003779 | H | MF | actin binding |
| 0.845 | GO:0008092 | H | MF | cytoskeletal protein binding |
| 0.808 | GO:0051020 | H | MF | GTPase binding |
| 0.788 | GO:0017016 | H | MF | Ras GTPase binding |
| 0.755 | GO:0001883 | H | MF | purine nucleoside binding |
| 0.754 | GO:0019222 | H | BP | regulation of metabolic process |
| 0.752 | GO:0003677 | H | MF | DNA binding |
| 0.741 | GO:0031267 | H | MF | small GTPase binding |
| 0.734 | GO:0000166 | H | MF | nucleotide binding |
| 0.728 | GO:0016740 | H | MF | transferase activity |
| 0.692 | GO:0035639 | H | MF | purine ribonucleoside triphosphate binding |
| 0.688 | GO:0032549 | H | MF | ribonucleoside binding |
| 0.679 | GO:0017076 | H | MF | purine nucleotide binding |
| 0.664 | GO:0044822 | H | MF | poly(A) RNA binding |
| 0.662 | GO:0001882 | H | MF | nucleoside binding |
| 0.639 | GO:0003824 | H | MF | catalytic activity |
| 0.624 | GO:0016874 | H | MF | ligase activity |
| 0.62 | GO:0019901 | H | MF | protein kinase binding |
| 0.607 | GO:0030554 | H | MF | adenyl nucleotide binding |
| 0.604 | GO:0019900 | H | MF | kinase binding |
| 0.584 | GO:0051641 | H | BP | cellular localization |
| 0.579 | GO:0006810 | H | BP | transport |
| 0.568 | GO:0016604 | H | CC | nuclear body |
| 0.566 | GO:0005524 | H | MF | ATP binding |
| 0.564 | GO:0016817 | H | MF | hydrolase activity, acting on acid anhydrides |
| 0.543 | GO:0005516 | H | MF | calmodulin binding |
| 0.53 | GO:0016887 | H | MF | ATPase activity |
| 0.521 | GO:0015631 | H | MF | tubulin binding |
| 0.518 | GO:0016818 | H | MF | hydrolase activity, acting on acid anhydrides, in phosphorus-containing anhydrides |
| 0.514 | GO:0015031 | H | BP | protein transport |
| 0.512 | GO:0034645 | H | BP | cellular macromolecule biosynthetic process |
| 0.502 | GO:0017111 | H | MF | nucleoside-triphosphatase activity |
| 0.5 | GO:0046907 | H | BP | intracellular transport |
| 0.965 | GO:0097159 | L | MF | organic cyclic compound binding |
| 0.962 | GO:0043229 | L | CC | intracellular organelle |
| 0.913 | GO:0005737 | L | CC | cytoplasm |
| 0.88 | GO:0043231 | L | CC | intracellular membrane-bounded organelle |
| 0.871 | GO:0044237 | L | BP | cellular metabolic process |
| 0.869 | GO:0005634 | L | CC | nucleus |
| 0.828 | GO:0034641 | L | BP | cellular nitrogen compound metabolic process |
| 0.826 | GO:0046483 | L | BP | heterocycle metabolic process |
| 0.824 | GO:0032403 | L | MF | protein complex binding |
| 0.822 | GO:0006725 | L | BP | cellular aromatic compound metabolic process |
| 0.808 | GO:0050896 | L | BP | response to stimulus |
| 0.803 | GO:0036094 | L | MF | small molecule binding |
| 0.802 | GO:0051716 | L | BP | cellular response to stimulus |
| 0.779 | GO:0006996 | L | BP | organelle organization |
| 0.776 | GO:0005829 | L | CC | cytosol |
| 0.771 | GO:0031981 | L | CC | nuclear lumen |
| 0.764 | GO:0016070 | L | BP | RNA metabolic process |
| 0.761 | GO:0009966 | L | BP | regulation of signal transduction |
| 0.757 | GO:0032991 | L | CC | macromolecular complex |
| 0.753 | GO:0008152 | L | BP | metabolic process |
| 0.743 | GO:0010467 | L | BP | gene expression |
| 0.728 | GO:0032502 | L | BP | developmental process |
| 0.724 | GO:0005102 | L | MF | receptor binding |
| 0.722 | GO:0005654 | L | CC | nucleoplasm |
| 0.698 | GO:0019904 | L | MF | protein domain specific binding |
| 0.691 | GO:0044267 | L | BP | cellular protein metabolic process |
| 0.686 | GO:0006464 | L | BP | cellular protein modification process |
| 0.683 | GO:0016787 | L | MF | hydrolase activity |
| 0.677 | GO:0048856 | L | BP | anatomical structure development |
| 0.673 | GO:0019538 | L | BP | protein metabolic process |
| 0.585 | GO:0007275 | L | BP | multicellular organismal development |
| 0.558 | GO:0051246 | L | BP | regulation of protein metabolic process |
| 0.553 | GO:0006807 | L | BP | nitrogen compound metabolic process |
| 0.55 | GO:0006139 | L | BP | nucleobase-containing compound metabolic process |
| 0.522 | GO:0032268 | L | BP | regulation of cellular protein metabolic process |
| 0.522 | GO:0046872 | L | MF | metal ion binding |
| 0.514 | GO:0007154 | L | BP | cell communication |
| 0.502 | GO:0016772 | L | MF | transferase activity, transferring phosphorus-containing groups |

**Blast**

| **BLAST Matched UniProt ID & name** | | | **Identity** | **E Value** | **Score** |
| --- | --- | --- | --- | --- | --- |
| Q8CCJ3 | UFL1_MOUSE | E3 UFM1-protein ligase 1 | 95.839 | 0.00E+00 | 1490 |
| Q4R367 | UFL1_MACFA | E3 UFM1-protein ligase 1 | 90.554 | 0.00E+00 | 1462 |
| O94874 | UFL1_HUMAN | E3 UFM1-protein ligase 1 | 90.68 | 0.00E+00 | 1420 |
| A1A4I9 | UFL1_BOVIN | E3 UFM1-protein ligase 1 | 89.043 | 0.00E+00 | 1392 |
| Q5ZMG1 | UFL1_CHICK | E3 UFM1-protein ligase 1 | 74.433 | 0.00E+00 | 1135 |
| Q6PGY6 | UFL1_DANRE | E3 UFM1-protein ligase 1 | 62.138 | 0.00E+00 | 983 |
| A7S2N8 | UFL1_NEMVE | E3 UFM1-protein ligase 1 homolog | 45.025 | 0.00E+00 | 666 |
| B3RYG4 | UFL1_TRIAD | E3 UFM1-protein ligase 1 homolog | 42.013 | 0.00E+00 | 637 |
| Q0IG18 | UFL1_AEDAE | E3 UFM1-protein ligase 1 homolog | 37.179 | 2.01E-161 | 494 |
| Q7Q373 | UFL1_ANOGA | E3 UFM1-protein ligase 1 homolog | 34.658 | 2.36E-152 | 471 |
| B4JV25 | UFL1_DROGR | E3 UFM1-protein ligase 1 homolog | 36.232 | 1.66E-150 | 466 |
| B0WU24 | UFL1_CULQU | E3 UFM1-protein ligase 1 homolog | 35.025 | 8.57E-150 | 464 |
| Q9VI55 | UFL1_DROME | E3 UFM1-protein ligase 1 homolog | 36.724 | 6.53E-146 | 454 |
| B4I4N0 | UFL1_DROSE | E3 UFM1-protein ligase 1 homolog | 36.196 | 3.95E-144 | 449 |
| B4QYZ3 | UFL1_DROSI | E3 UFM1-protein ligase 1 homolog | 36.196 | 5.49E-144 | 449 |
| B3P2S2 | UFL1_DROER | E3 UFM1-protein ligase 1 homolog | 36.196 | 1.01E-143 | 449 |
| B4PS24 | UFL1_DROYA | E3 UFM1-protein ligase 1 homolog | 35.894 | 3.92E-141 | 442 |
| Q296V2 | UFL1_DROPS | E3 UFM1-protein ligase 1 homolog | 34.91 | 1.65E-138 | 435 |
| B4GEL3 | UFL1_DROPE | E3 UFM1-protein ligase 1 homolog | 35.038 | 7.73E-138 | 433 |
| B4KDK5 | UFL1_DROMO | E3 UFM1-protein ligase 1 homolog | 34.66 | 1.58E-134 | 424 |
| B4LZW7 | UFL1_DROVI | E3 UFM1-protein ligase 1 homolog | 35.667 | 1.65E-132 | 419 |
| B4NAB3 | UFL1_DROWI | E3 UFM1-protein ligase 1 homolog | 35.348 | 3.02E-131 | 416 |
| B3LYC0 | UFL1_DROAN | E3 UFM1-protein ligase 1 homolog | 34.428 | 4.39E-127 | 405 |
| B8AXB6 | UFL1_ORYSI | E3 UFM1-protein ligase 1 homolog | 28.9 | 2.35E-89 | 305 |
| Q9LX73 | UFL1_ARATH | E3 UFM1-protein ligase 1 homolog | 26.918 | 8.55E-71 | 253 |
| Q54QS0 | UFL1_DICDI | E3 UFM1-protein ligase 1 homolog | 30.769 | 3.82E-60 | 222 |
| Q17750 | UFL1_CAEEL | E3 UFM1-protein ligase 1 homolog | 28.614 | 7.02E-38 | 155 |
| A8WN14 | UFL1_CAEBR | E3 UFM1-protein ligase 1 homolog | 25.428 | 2.32E-31 | 134 |

**Protein Name: Endoglucanase**

**Predicted results:**

**SVMProt**

| **Protein Family Name** | **GO Category** | **SVM** | **PNN** | **KNN** |
| --- | --- | --- | --- | --- |
| **Molecular Function** | | | | |
| EC3.2 Hydrolases - Glycosylases | - | 85.4 | Y | Y |
| All lipid-binding proteins | GO:0008289 lipid binding | 93.6 | - | - |
| EC3.1 Hydrolases - Acting on Ester Bonds | - | 83.9 | - | - |
| Zinc-binding | GO:0008270 zinc binding | 62.2 | - | - |

**FFPred**

| **Score** | **GO term** | **RL** | **Domain** | **Description** |
| --- | --- | --- | --- | --- |
| 0.898 | GO:0070062 | H | CC | extracellular vesicular exosome |
| 0.887 | GO:0031224 | H | CC | intrinsic component of membrane |
| 0.882 | GO:0005576 | H | CC | extracellular region |
| 0.88 | GO:0016020 | H | CC | membrane |
| 0.847 | GO:0005615 | H | CC | extracellular space |
| 0.827 | GO:0038023 | H | MF | signaling receptor activity |
| 0.795 | GO:0006810 | H | BP | transport |
| 0.789 | GO:0005886 | H | CC | plasma membrane |
| 0.782 | GO:0004871 | H | MF | signal transducer activity |
| 0.778 | GO:0071944 | H | CC | cell periphery |
| 0.77 | GO:0031982 | H | CC | vesicle |
| 0.71 | GO:0002684 | H | BP | positive regulation of immune system process |
| 0.709 | GO:0031988 | H | CC | membrane-bounded vesicle |
| 0.665 | GO:0005125 | H | MF | cytokine activity |
| 0.655 | GO:0003823 | H | MF | antigen binding |
| 0.649 | GO:0012505 | H | CC | endo membrane system |
| 0.613 | GO:0006952 | H | BP | defense response |
| 0.607 | GO:0006955 | H | BP | immune response |
| 0.602 | GO:0007166 | H | BP | cell surface receptor signaling pathway |
| 0.6 | GO:0045087 | H | BP | innate immune response |
| 0.583 | GO:0019222 | H | BP | regulation of metabolic process |
| 0.571 | GO:0002376 | H | BP | immune system process |
| 0.57 | GO:0044281 | H | BP | small molecule metabolic process |
| 0.531 | GO:0030246 | H | MF | carbohydrate binding |
| 0.528 | GO:0002682 | H | BP | regulation of immune system process |
| 0.94 | GO:0008152 | L | BP | metabolic process |
| 0.907 | GO:0050896 | L | BP | response to stimulus |
| 0.888 | GO:0046872 | L | MF | metalion binding |
| 0.835 | GO:0051716 | L | BP | cellular response to stimulus |
| 0.805 | GO:0005102 | L | MF | receptor binding |
| 0.801 | GO:0043169 | L | MF | cation binding |
| 0.778 | GO:0007154 | L | BP | cell communication |
| 0.777 | GO:0048856 | L | BP | anatomical structure development |
| 0.774 | GO:0019538 | L | BP | protein metabolic process |
| 0.774 | GO:0005737 | L | CC | cytoplasm |
| 0.721 | GO:0007275 | L | BP | multicellular organismal development |
| 0.72 | GO:0043231 | L | CC | intracellular membrane-bounded organelle |
| 0.695 | GO:0007165 | L | BP | signal transduction |
| 0.685 | GO:0032502 | L | BP | developmental process |
| 0.673 | GO:0043229 | L | CC | intracellular organelle |
| 0.641 | GO:0023052 | L | BP | signaling |
| 0.62 | GO:0043234 | L | CC | protein complex |
| 0.606 | GO:0032403 | L | MF | protein complex binding |
| 0.603 | GO:0030154 | L | BP | cell differentiation |
| 0.588 | GO:0048513 | L | BP | organ development |
| 0.585 | GO:0009605 | L | BP | response to external stimulus |
| 0.584 | GO:0032991 | L | CC | macromolecular complex |
| 0.527 | GO:0050790 | L | BP | regulation of catalytic activity |
| 0.524 | GO:0036094 | L | MF | small molecule binding |
| 0.52 | GO:0009893 | L | BP | positive regulation of metabolic process |
| 0.516 | GO:0016787 | L | MF | hydrolase activity |
| 0.509 | GO:0051246 | L | BP | regulation of protein metabolic process |

**Blast**

| **BLAST Matched UniProt ID & name** | | | **Identity** | **E Value** | **Score** |
| --- | --- | --- | --- | --- | --- |
| Q12679 | GUNA_ASPKW | Endoglucanase A | 87.866 | 3.24E-155 | 437 |
| P22669 | GUN_ASPAC | Endoglucanase-1 | 65.297 | 1.41E-101 | 301 |
| G4NAZ1 | CE12A_MAGO7 | Endoglucanase cel12A | 47.083 | 8.99E-71 | 223 |
| G4N5V2 | CE12B_MAGO7 | Endoglucanase cel12B | 44.348 | 8.07E-60 | 195 |
| Q2UK93 | XGEA_ASPOR | Probable xyloglucan-specific endo-beta-1,4-glucanase A | 41.964 | 6.39E-51 | 172 |
| G4NBX2 | CE12C_MAGO7 | Endoglucanase cel12C | 44.545 | 1.51E-49 | 170 |
| Q4WJU8 | XGEA_ASPFU | Probable xyloglucan-specific endo-beta-1,4-glucanase A | 40.891 | 5.17E-49 | 167 |
| A1XP58 | XGEA_ASPNG | Xyloglucan-specific endo-beta-1,4-glucanase A | 40 | 2.55E-48 | 165 |
| B0XNQ1 | XGEA_ASPFC | Probable xyloglucan-specific endo-beta-1,4-glucanase A | 41.593 | 6.56E-48 | 164 |
| Q0CRC9 | XGEA_ASPTN | Probable xyloglucan-specific endo-beta-1,4-glucanase A | 42.731 | 8.57E-48 | 164 |
| A2Q877 | XGEA_ASPNC | Probable xyloglucan-specific endo-beta-1,4-glucanase A | 39.732 | 9.59E-48 | 163 |
| A1D4F1 | XGEA_NEOFI | Probable xyloglucan-specific endo-beta-1,4-glucanase A | 41.15 | 1.43E-46 | 160 |
| O94218 | XGEA_ASPAC | Xyloglucan-specific endo-beta-1,4-glucanase A | 42.152 | 5.29E-45 | 156 |
| A1CRJ0 | XGEA_ASPCL | Probable xyloglucan-specific endo-beta-1,4-glucanase A | 38.618 | 5.06E-44 | 154 |
| Q5BG78 | XGEA_EMENI | Xyloglucan-specific endo-beta-1,4-glucanase A | 43.519 | 5.24E-42 | 148 |

**Protein Name: XYN VI**

**Predicted results:**

**SVMProt**

| **Protein Family Name** | **GO Category** | **SVM** | **PNN** | **KNN** |
| --- | --- | --- | --- | --- |
| **Molecular Function** | | | | |
| EC3.2 Hydrolases - Glycosylases | - | 97.7 | - | Y |
| Zinc-binding | GO:0008270 zinc binding | 92.9 | - | - |
| EC3.4 Hydrolases - Acting on peptide bonds (Peptidases) | - | 80.4 | - | - |
| Manganese-binding | GO:0030145 manganese binding | 71.3 | ? | ? |
| Metal-binding | GO:0046872 metal ion binding | 65.4 | - | - |
| All lipid-binding proteins | GO:0008289 lipid binding | 58.6 | - | - |
| Calcium-binding | - | 58.6 | - | - |
| Copper-binding | GO:0005507 copper binding | 58.6 | - | - |

**FFPred**

| **Score** | **GO term** | **RL** | **Domain** | **Description** |
| --- | --- | --- | --- | --- |
| 0.943 | GO:0005576 | H | CC | extracellular region |
| 0.923 | GO:0003824 | H | MF | catalytic activity |
| 0.915 | GO:0070062 | H | CC | extracellular vesicular exosome |
| 0.888 | GO:0008236 | H | MF | serine-type peptidase activity |
| 0.847 | GO:0005615 | H | CC | extracellular space |
| 0.833 | GO:0030246 | H | MF | carbohydrate binding |
| 0.832 | GO:0031988 | H | CC | membrane-bounded vesicle |
| 0.832 | GO:0016020 | H | CC | membrane |
| 0.828 | GO:0071944 | H | CC | cell periphery |
| 0.821 | GO:0005886 | H | CC | plasma membrane |
| 0.817 | GO:0009897 | H | CC | external side of plasma membrane |
| 0.81 | GO:0031982 | H | CC | vesicle |
| 0.758 | GO:0012505 | H | CC | endomembrane system |
| 0.718 | GO:0016021 | H | CC | integral component of membrane |
| 0.709 | GO:0050778 | H | BP | positive regulation of immune response |
| 0.696 | GO:0008233 | H | MF | peptidase activity |
| 0.691 | GO:0002376 | H | BP | immune system process |
| 0.674 | GO:0005975 | H | BP | carbohydrate metabolic process |
| 0.659 | GO:0006629 | H | BP | lipid metabolic process |
| 0.653 | GO:0019222 | H | BP | regulation of metabolic process |
| 0.614 | GO:0007166 | H | BP | cell surface receptor signaling pathway |
| 0.607 | GO:0006952 | H | BP | defense response |
| 0.586 | GO:0009056 | H | BP | catabolic process |
| 0.581 | GO:0098588 | H | CC | bounding membrane of organelle |
| 0.58 | GO:0002682 | H | BP | regulation of immune system process |
| 0.577 | GO:0004866 | H | MF | endopeptidase inhibitor activity |
| 0.566 | GO:0051707 | H | BP | response to other organism |
| 0.561 | GO:0009059 | H | BP | macromolecule biosynthetic process |
| 0.547 | GO:0005783 | H | CC | endoplasmic reticulum |
| 0.545 | GO:0006955 | H | BP | immune response |
| 0.525 | GO:0031224 | H | CC | intrinsic component of membrane |
| 0.518 | GO:0006810 | H | BP | transport |
| 0.934 | GO:0008152 | L | BP | metabolic process |
| 0.932 | GO:0050896 | L | BP | response to stimulus |
| 0.847 | GO:0005102 | L | MF | receptor binding |
| 0.833 | GO:0051716 | L | BP | cellular response to stimulus |
| 0.819 | GO:0016787 | L | MF | hydrolase activity |
| 0.816 | GO:0006807 | L | BP | nitrogen compound metabolic process |
| 0.804 | GO:0005737 | L | CC | cytoplasm |
| 0.799 | GO:0046872 | L | MF | metal ion binding |
| 0.791 | GO:0048856 | L | BP | anatomical structure development |
| 0.769 | GO:0007275 | L | BP | multicellular organismal development |
| 0.745 | GO:0044237 | L | BP | cellular metabolic process |
| 0.713 | GO:0007154 | L | BP | cell communication |
| 0.707 | GO:0043169 | L | MF | cation binding |
| 0.707 | GO:0032502 | L | BP | developmental process |
| 0.705 | GO:0009607 | L | BP | response to biotic stimulus |
| 0.691 | GO:0005764 | L | CC | lysosome |
| 0.688 | GO:0019538 | L | BP | protein metabolic process |
| 0.679 | GO:0007165 | L | BP | signal transduction |
| 0.678 | GO:0043231 | L | CC | intracellular membrane-bounded organelle |
| 0.673 | GO:0009966 | L | BP | regulation of signal transduction |
| 0.64 | GO:0030154 | L | BP | cell differentiation |
| 0.628 | GO:0097159 | L | MF | organic cyclic compound binding |
| 0.622 | GO:0048513 | L | BP | organ development |
| 0.615 | GO:0005773 | L | CC | vacuole |
| 0.61 | GO:0043229 | L | CC | intracellular organelle |
| 0.594 | GO:0009605 | L | BP | response to external stimulus |
| 0.576 | GO:0010033 | L | BP | response to organic substance |
| 0.562 | GO:0044267 | L | BP | cellular protein metabolic process |
| 0.561 | GO:0009893 | L | BP | positive regulation of metabolic process |
| 0.534 | GO:0071310 | L | BP | cellular response to organic substance |
| 0.533 | GO:0050790 | L | BP | regulation of catalytic activity |
| 0.52 | GO:0023052 | L | BP | signaling |
| 0.518 | GO:0051246 | L | BP | regulation of protein metabolic process |
| 0.506 | GO:0009653 | L | BP | anatomical structure morphogenesis |

**Blast**

| **BLAST Matched UniProt ID & name** | | | **Identity** | **E Value** | **Score** |
| --- | --- | --- | --- | --- | --- |
| O16580 | GLCM1_CAEEL | Putative glucosylceramidase 1 | 23.636 | 8.02E-06 | 51.6 |
| Q9UB00 | GLCM4_CAEEL | Putative glucosylceramidase 4 | 22.986 | 9.66E-05 | 48.1 |

**Protein Name: L-lysine decarboxylase/oxidase**

**Predicted results:**

**SVMProt**

| **Protein Family Name** | **GO Category** | **SVM** | **PNN** | **KNN** |
| --- | --- | --- | --- | --- |
| **Molecular Function** | | | | |
| EC4.1 Lyases - Carbon-Carbon Lyases | - | 68.5 | Y | Y |
| EC1.5 Oxidoreductases - Acting on the CH-NH group of donors | - | 80.4 | ? | ? |
| TC9.B Incompletely Characterized Transport Systems - Putative uncharacterized transport proteins | - | 80.4 | ? | ? |
| EC1.14 Oxidoreductases - Acting on paired donors with incorporation or reduction of molecular oxygen | - | 58.6 | ? | ? |
| **Broadly Defined Function** | | | | |
| Photosystem I | GO:0009522 photosystem I | 58.6 | ? | ? |

**FFPred**

| **Score** | **GO term** | **RL** | **Domain** | **Description** |
| --- | --- | --- | --- | --- |
| 0.98 | GO:0003824 | H | MF | catalytic activity |
| 0.935 | GO:0006082 | H | BP | organic acid metabolic process |
| 0.924 | GO:0044281 | H | BP | small molecule metabolic process |
| 0.895 | GO:0019752 | H | BP | carboxylic acid metabolic process |
| 0.894 | GO:0016740 | H | MF | transferase activity |
| 0.857 | GO:0000166 | H | MF | nucleotide binding |
| 0.842 | GO:0005739 | H | CC | mitochondrion |
| 0.832 | GO:0005759 | H | CC | mitochondrial matrix |
| 0.823 | GO:0032787 | H | BP | monocarboxylic acid metabolic process |
| 0.804 | GO:0005975 | H | BP | carbohydrate metabolic process |
| 0.798 | GO:0055114 | H | BP | oxidation-reduction process |
| 0.761 | GO:0017076 | H | MF | purine nucleotide binding |
| 0.757 | GO:0035639 | H | MF | purine ribonucleoside triphosphate binding |
| 0.756 | GO:0019222 | H | BP | regulation of metabolic process |
| 0.75 | GO:0005743 | H | CC | mitochondrial inner membrane |
| 0.741 | GO:0001883 | H | MF | purine nucleoside binding |
| 0.736 | GO:0006796 | H | BP | phosphate-containing compound metabolic process |
| 0.731 | GO:0032549 | H | MF | ribonucleoside binding |
| 0.725 | GO:0001882 | H | MF | nucleoside binding |
| 0.71 | GO:0030554 | H | MF | adenyl nucleotide binding |
| 0.699 | GO:0006629 | H | BP | lipid metabolic process |
| 0.697 | GO:0009056 | H | BP | catabolic process |
| 0.692 | GO:0005576 | H | CC | extracellular region |
| 0.654 | GO:0031982 | H | CC | vesicle |
| 0.653 | GO:0031966 | H | CC | mitochondrial membrane |
| 0.652 | GO:0070062 | H | CC | extracellular vesicular exosome |
| 0.623 | GO:0051186 | H | BP | cofactor metabolic process |
| 0.614 | GO:0005740 | H | CC | mitochondrial envelope |
| 0.6 | GO:0016757 | H | MF | transferase activity, transferring glycosyl groups |
| 0.598 | GO:0005524 | H | MF | ATP binding |
| 0.557 | GO:0016020 | H | CC | membrane |
| 0.54 | GO:0051171 | H | BP | regulation of nitrogen compound metabolic process |
| 0.529 | GO:0055086 | H | BP | nucleobase-containing small molecule metabolic process |
| 0.529 | GO:0031988 | H | CC | membrane-bounded vesicle |
| 0.526 | GO:0006163 | H | BP | purine nucleotide metabolic process |
| 0.512 | GO:0019637 | H | BP | organophosphate metabolic process |
| 0.509 | GO:1901605 | H | BP | alpha-amino acid metabolic process |
| 0.973 | GO:0008152 | L | BP | metabolic process |
| 0.962 | GO:0044237 | L | BP | cellular metabolic process |
| 0.945 | GO:0005737 | L | CC | cytoplasm |
| 0.92 | GO:0043229 | L | CC | intracellular organelle |
| 0.907 | GO:0097159 | L | MF | organic cyclic compound binding |
| 0.878 | GO:0036094 | L | MF | small molecule binding |
| 0.857 | GO:0009058 | L | BP | biosynthetic process |
| 0.852 | GO:0050896 | L | BP | response to stimulus |
| 0.817 | GO:0051716 | L | BP | cellular response to stimulus |
| 0.805 | GO:0043231 | L | CC | intracellular membrane-bounded organelle |
| 0.803 | GO:0019538 | L | BP | protein metabolic process |
| 0.8 | GO:0032502 | L | BP | developmental process |
| 0.795 | GO:0046872 | L | MF | metal ion binding |
| 0.793 | GO:0043169 | L | MF | cation binding |
| 0.767 | GO:0034641 | L | BP | cellular nitrogen compound metabolic process |
| 0.754 | GO:0005634 | L | CC | nucleus |
| 0.736 | GO:0044267 | L | BP | cellular protein metabolic process |
| 0.734 | GO:0046483 | L | BP | heterocycle metabolic process |
| 0.726 | GO:0006725 | L | BP | cellular aromatic compound metabolic process |
| 0.725 | GO:0007154 | L | BP | cell communication |
| 0.711 | GO:0005829 | L | CC | cytosol |
| 0.694 | GO:0007165 | L | BP | signal transduction |
| 0.692 | GO:0023052 | L | BP | signaling |
| 0.689 | GO:0006807 | L | BP | nitrogen compound metabolic process |
| 0.687 | GO:0043234 | L | CC | protein complex |
| 0.67 | GO:0031981 | L | CC | nuclear lumen |
| 0.661 | GO:0006996 | L | BP | organelle organization |
| 0.658 | GO:0071310 | L | BP | cellular response to organic substance |
| 0.629 | GO:0005102 | L | MF | receptor binding |
| 0.625 | GO:0005654 | L | CC | nucleoplasm |
| 0.618 | GO:0007275 | L | BP | multicellular organismal development |
| 0.571 | GO:0010033 | L | BP | response to organic substance |
| 0.565 | GO:0032991 | L | CC | macromolecular complex |
| 0.564 | GO:0010467 | L | BP | gene expression |
| 0.546 | GO:0019318 | L | BP | hexose metabolic process |
| 0.538 | GO:0032403 | L | MF | protein complex binding |
| 0.522 | GO:0048856 | L | BP | anatomical structure development |

**Blast**

| **BLAST Matched UniProt ID & name** | | | **Identity** | **E Value** | **Score** |
| --- | --- | --- | --- | --- | --- |
| P28629 | ADIA_ECOLI | Biodegradative arginine decarboxylase | 49.736 | 0 | 801 |
| P05033 | DCLY_HAFAL | Lysine decarboxylase | 38.806 | 4.01E-177 | 533 |
| P0A9H4 | LDCI_ECO57 | Lysine decarboxylase, inducible | 38.736 | 4.79E-174 | 525 |
| P0A1Z0 | LDCI_SALTY | Lysine decarboxylase, inducible | 38.049 | 2.33E-171 | 518 |
| P52095 | DCLZ_ECOLI | Lysine decarboxylase, constitutive | 36.757 | 2.29E-161 | 491 |
| P24169 | DCOS_ECOLI | Ornithine decarboxylase, inducible | 32.336 | 3.99E-107 | 350 |
| P21169 | DCOR_ECOLI | Ornithine decarboxylase, constitutive | 33.094 | 1.66E-105 | 345 |
| P43099 | DCOR_LACS3 | Ornithine decarboxylase, inducible | 32.452 | 4.61E-101 | 334 |
| P44317 | DCOR_HAEIN | Ornithine decarboxylase | 32.07 | 5.65E-100 | 331 |
| P37536 | YAAO_BACSU | Uncharacterized protein YaaO | 26.667 | 9.55E-23 | 105 |
| Q9K9K5 | SPEA_BACHD | Arginine decarboxylase | 27.036 | 8.56E-19 | 94 |
| Q9K9K5 | SPEA_BACHD | Arginine decarboxylase | 32.099 | 0.009 | 42.7 |
| Q81MS2 | SPEA_BACAN | Arginine decarboxylase | 25.203 | 4.70E-16 | 85.1 |
| Q81MS2 | SPEA_BACAN | Arginine decarboxylase | 24.161 | 1.20E-02 | 42.4 |
| Q819L4 | SPEA_BACCR | Arginine decarboxylase | 25.745 | 7.72E-16 | 84.3 |
| Q819L4 | SPEA_BACCR | Arginine decarboxylase | 24 | 1.70E-02 | 42 |
| P21885 | SPEA_BACSU | Arginine decarboxylase | 25 | 1.74E-14 | 80.1 |
| P21885 | SPEA_BACSU | Arginine decarboxylase | 39.216 | 2.80E-02 | 41.2 |

**Protein Name: AcL-RbI**

**Predicted results:**

**SVMProt**

| **Protein Family Name** | **GO Category** | **SVM** | **PNN** | **KNN** |
| --- | --- | --- | --- | --- |
| **Molecular Function** | | | | |
| Zinc-binding | GO:0008270 zinc binding | 98.9 | - | - |
| EC2.7 Transferases - Transferring Phosphorus-Containing Groups | - | 68.5 | - | Y |
| EC4.1 Lyases - Carbon-Carbon Lyases | - | 68.5 | - | Y |
| Metal-binding | GO:0046872 metal ion binding | 76.2 | - | - |
| Iron-binding | GO:0005506 iron binding | 71.3 | - | - |
| Copper-binding | GO:0005507 copper binding | 58.6 | - | - |
| Actin binding | GO:0003779 actin binding | 58.6 | ? | ? |
| Calcium-binding | - | 58.6 | - | - |

**FFPred**

| **Score** | **GO term** | **RL** | **Domain** | **Description** |
| --- | --- | --- | --- | --- |
| 0.918 | GO:0003824 | H | MF | catalytic activity |
| 0.787 | GO:0005739 | H | CC | mitochondrion |
| 0.708 | GO:0019222 | H | BP | regulation of metabolic process |
| 0.673 | GO:0016567 | H | BP | protein ubiquitination |
| 0.661 | GO:0016740 | H | MF | transferase activity |
| 0.653 | GO:0005759 | H | CC | mitochondrial matrix |
| 0.621 | GO:0017076 | H | MF | purine nucleotide binding |
| 0.62 | GO:0048037 | H | MF | cofactor binding |
| 0.618 | GO:0044281 | H | BP | small molecule metabolic process |
| 0.617 | GO:0006811 | H | BP | ion transport |
| 0.605 | GO:0070062 | H | CC | extracellular vesicular exosome |
| 0.6 | GO:0016874 | H | MF | ligase activity |
| 0.589 | GO:0005740 | H | CC | mitochondrial envelope |
| 0.583 | GO:0006082 | H | BP | organic acid metabolic process |
| 0.577 | GO:0046914 | H | MF | transition metal ion binding |
| 0.572 | GO:0031982 | H | CC | vesicle |
| 0.571 | GO:0019787 | H | MF | ubiquitin-like protein transferase activity |
| 0.57 | GO:0009056 | H | BP | catabolic process |
| 0.562 | GO:0004842 | H | MF | ubiquitin-protein transferase activity |
| 0.561 | GO:0016491 | H | MF | oxidoreductase activity |
| 0.557 | GO:0009059 | H | BP | macromolecule biosynthetic process |
| 0.555 | GO:0035639 | H | MF | purine ribonucleoside triphosphate binding |
| 0.552 | GO:0055114 | H | BP | oxidation-reduction process |
| 0.551 | GO:0005576 | H | CC | extracellular region |
| 0.55 | GO:0032549 | H | MF | ribonucleoside binding |
| 0.549 | GO:0006810 | H | BP | transport |
| 0.542 | GO:0031966 | H | CC | mitochondrial membrane |
| 0.521 | GO:0000166 | H | MF | nucleotide binding |
| 0.508 | GO:0016311 | H | BP | dephosphorylation |
| 0.504 | GO:0031988 | H | CC | membrane-bounded vesicle |
| 0.501 | GO:0034645 | H | BP | cellular macromolecule biosynthetic process |
| 0.501 | GO:0001882 | H | MF | nucleoside binding |
| 0.966 | GO:0043229 | L | CC | intracellular organelle |
| 0.941 | GO:0005737 | L | CC | cytoplasm |
| 0.936 | GO:0043231 | L | CC | intracellular membrane-bounded organelle |
| 0.915 | GO:0019538 | L | BP | protein metabolic process |
| 0.909 | GO:0044237 | L | BP | cellular metabolic process |
| 0.904 | GO:0008152 | L | BP | metabolic process |
| 0.897 | GO:0043169 | L | MF | cation binding |
| 0.856 | GO:0046872 | L | MF | metal ion binding |
| 0.855 | GO:0050896 | L | BP | response to stimulus |
| 0.845 | GO:0006139 | L | BP | nucleobase-containing compound metabolic process |
| 0.844 | GO:0044267 | L | BP | cellular protein metabolic process |
| 0.835 | GO:0006807 | L | BP | nitrogen compound metabolic process |
| 0.824 | GO:0034641 | L | BP | cellular nitrogen compound metabolic process |
| 0.824 | GO:0051716 | L | BP | cellular response to stimulus |
| 0.823 | GO:0032991 | L | CC | macromolecular complex |
| 0.803 | GO:0007165 | L | BP | signal transduction |
| 0.8 | GO:0006464 | L | BP | cellular protein modification process |
| 0.797 | GO:0046483 | L | BP | heterocycle metabolic process |
| 0.791 | GO:0005634 | L | CC | nucleus |
| 0.781 | GO:0006725 | L | BP | cellular aromatic compound metabolic process |
| 0.75 | GO:0005829 | L | CC | cytosol |
| 0.748 | GO:0009058 | L | BP | biosynthetic process |
| 0.741 | GO:0016787 | L | MF | hydrolase activity |
| 0.738 | GO:0031981 | L | CC | nuclear lumen |
| 0.732 | GO:0023052 | L | BP | signaling |
| 0.731 | GO:0005102 | L | MF | receptor binding |
| 0.695 | GO:0007154 | L | BP | cell communication |
| 0.668 | GO:0043234 | L | CC | protein complex |
| 0.665 | GO:0010467 | L | BP | gene expression |
| 0.655 | GO:0032502 | L | BP | developmental process |
| 0.651 | GO:0005654 | L | CC | nucleoplasm |
| 0.619 | GO:0007275 | L | BP | multicellular organismal development |
| 0.614 | GO:0048856 | L | BP | anatomical structure development |
| 0.58 | GO:0016070 | L | BP | RNA metabolic process |
| 0.578 | GO:0006996 | L | BP | organelle organization |
| 0.573 | GO:0097159 | L | MF | organic cyclic compound binding |
| 0.507 | GO:0010033 | L | BP | response to organic substance |

**Protein Name: AtGALT29A**

**Predicted results:**

**SVMProt**

| **Protein Family Name** | **GO Category** | **SVM** | **PNN** | **KNN** |
| --- | --- | --- | --- | --- |
| **Molecular Function** | | | | |
| All lipid-binding proteins | GO:0008289 lipid binding | 73.8 | - | - |
| TC3.A.3 P-type ATPase (P-ATPase) family | - | 58.6 | ? | ? |
| Metal-binding | GO:0046872 metal ion binding | 58.6 | - | - |
| **Biological Process** | | | | |
| DNA recombination | GO:0006310 DNA recombination | 58.6 | ? | ? |
| DNA repair | GO:0006281 DNA repair | 58.6 | ? | ? |

**FFPred**

| **Score** | **GO term** | **RL** | **Domain** | **Description** |
| --- | --- | --- | --- | --- |
| 0.924 | GO:0034645 | H | BP | cellular macromolecule biosynthetic process |
| 0.922 | GO:0003676 | H | MF | nucleic acid binding |
| 0.906 | GO:0019222 | H | BP | regulation of metabolic process |
| 0.873 | GO:0005739 | H | CC | mitochondrion |
| 0.811 | GO:0006810 | H | BP | transport |
| 0.809 | GO:0003735 | H | MF | structural constituent of ribosome |
| 0.809 | GO:0016740 | H | MF | transferase activity |
| 0.803 | GO:0016020 | H | CC | membrane |
| 0.795 | GO:0008092 | H | MF | cytoskeletal protein binding |
| 0.793 | GO:0003824 | H | MF | catalytic activity |
| 0.789 | GO:0009059 | H | BP | macromolecule biosynthetic process |
| 0.786 | GO:0031966 | H | CC | mitochondrial membrane |
| 0.785 | GO:0055085 | H | BP | transmembrane transport |
| 0.762 | GO:0000166 | H | MF | nucleotide binding |
| 0.756 | GO:0070062 | H | CC | extracellular vesicular exosome |
| 0.752 | GO:0006412 | H | BP | translation |
| 0.743 | GO:0005840 | H | CC | ribosome |
| 0.725 | GO:0043087 | H | BP | regulation of GTPase activity |
| 0.711 | GO:0031982 | H | CC | vesicle |
| 0.701 | GO:0045333 | H | BP | cellular respiration |
| 0.699 | GO:0010468 | H | BP | regulation of gene expression |
| 0.698 | GO:0051171 | H | BP | regulation of nitrogen compound metabolic process |
| 0.694 | GO:0005743 | H | CC | mitochondrial inner membrane |
| 0.69 | GO:0035639 | H | MF | purine ribonucleoside triphosphate binding |
| 0.673 | GO:0015631 | H | MF | tubulin binding |
| 0.667 | GO:0006355 | H | BP | regulation of transcription, DNA-templated |
| 0.651 | GO:0051252 | H | BP | regulation of RNA metabolic process |
| 0.65 | GO:0005576 | H | CC | extracellular region |
| 0.644 | GO:0044281 | H | BP | small molecule metabolic process |
| 0.643 | GO:0001883 | H | MF | purine nucleoside binding |
| 0.635 | GO:0015075 | H | MF | ion transmembrane transporter activity |
| 0.633 | GO:0044822 | H | MF | poly(A) RNA binding |
| 0.631 | GO:0003723 | H | MF | RNA binding |
| 0.629 | GO:0006812 | H | BP | cation transport |
| 0.627 | GO:0006796 | H | BP | phosphate-containing compound metabolic process |
| 0.583 | GO:0022857 | H | MF | transmembrane transporter activity |
| 0.583 | GO:0016310 | H | BP | phosphorylation |
| 0.577 | GO:0003779 | H | MF | actin binding |
| 0.576 | GO:0016491 | H | MF | oxidoreductase activity |
| 0.565 | GO:0006091 | H | BP | generation of precursor metabolites and energy |
| 0.562 | GO:0032318 | H | BP | regulation of Ras GTPase activity |
| 0.56 | GO:0005215 | H | MF | transporter activity |
| 0.556 | GO:0008017 | H | MF | microtubule binding |
| 0.554 | GO:0030554 | H | MF | adenyl nucleotide binding |
| 0.552 | GO:0031090 | H | CC | organelle membrane |
| 0.55 | GO:0005524 | H | MF | ATP binding |
| 0.55 | GO:0006811 | H | BP | ion transport |
| 0.549 | GO:1903506 | H | BP | regulation of nucleic acid-templated transcription |
| 0.541 | GO:0051641 | H | BP | cellular localization |
| 0.534 | GO:0046914 | H | MF | transition metal ion binding |
| 0.51 | GO:0030234 | H | MF | enzyme regulator activity |
| 0.501 | GO:0031988 | H | CC | membrane-bounded vesicle |
| 0.501 | GO:0031224 | H | CC | intrinsic component of membrane |
| 0.5 | GO:0005198 | H | MF | structural molecule activity |
| 0.975 | GO:0008152 | L | BP | metabolic process |
| 0.95 | GO:0005737 | L | CC | cytoplasm |
| 0.946 | GO:0043229 | L | CC | intracellular organelle |
| 0.943 | GO:0044237 | L | BP | cellular metabolic process |
| 0.937 | GO:0097159 | L | MF | organic cyclic compound binding |
| 0.912 | GO:0046872 | L | MF | metal ion binding |
| 0.875 | GO:0043231 | L | CC | intracellular membrane-bounded organelle |
| 0.867 | GO:0050896 | L | BP | response to stimulus |
| 0.838 | GO:0009058 | L | BP | biosynthetic process |
| 0.836 | GO:0051716 | L | BP | cellular response to stimulus |
| 0.811 | GO:0034641 | L | BP | cellular nitrogen compound metabolic process |
| 0.797 | GO:0006807 | L | BP | nitrogen compound metabolic process |
| 0.788 | GO:0005634 | L | CC | nucleus |
| 0.787 | GO:0043169 | L | MF | cation binding |
| 0.784 | GO:0019538 | L | BP | protein metabolic process |
| 0.783 | GO:0007154 | L | BP | cell communication |
| 0.781 | GO:0005102 | L | MF | receptor binding |
| 0.775 | GO:0046483 | L | BP | heterocycle metabolic process |
| 0.762 | GO:0007165 | L | BP | signal transduction |
| 0.761 | GO:0032502 | L | BP | developmental process |
| 0.757 | GO:0006725 | L | BP | cellular aromatic compound metabolic process |
| 0.757 | GO:0048856 | L | BP | anatomical structure development |
| 0.754 | GO:0006139 | L | BP | nucleobase-containing compound metabolic process |
| 0.746 | GO:0032991 | L | CC | macromolecular complex |
| 0.737 | GO:0032403 | L | MF | protein complex binding |
| 0.732 | GO:0009893 | L | BP | positive regulation of metabolic process |
| 0.72 | GO:0005829 | L | CC | cytosol |
| 0.71 | GO:0023052 | L | BP | signaling |
| 0.697 | GO:0031981 | L | CC | nuclear lumen |
| 0.68 | GO:0036094 | L | MF | small molecule binding |
| 0.675 | GO:0050790 | L | BP | regulation of catalytic activity |
| 0.674 | GO:0044267 | L | BP | cellular protein metabolic process |
| 0.672 | GO:0043234 | L | CC | protein complex |
| 0.655 | GO:0007275 | L | BP | multicellular organismal development |
| 0.622 | GO:0010467 | L | BP | gene expression |
| 0.621 | GO:0005654 | L | CC | nucleoplasm |
| 0.603 | GO:0030154 | L | BP | cell differentiation |
| 0.597 | GO:0006996 | L | BP | organelle organization |
| 0.592 | GO:0019904 | L | MF | protein domain specific binding |
| 0.581 | GO:0016070 | L | BP | RNA metabolic process |
| 0.566 | GO:0009966 | L | BP | regulation of signal transduction |
| 0.565 | GO:0016787 | L | MF | hydrolase activity |
| 0.525 | GO:0005856 | L | CC | cytoskeleton |
| 0.524 | GO:0048513 | L | BP | organ development |
| 0.52 | GO:0006464 | L | BP | cellular protein modification process |
| 0.501 | GO:0010033 | L | BP | response to organic substance |

**Protein Name: Lhyal**

**Predicted results:**

**SVMProt**

| **Protein Family Name** | **GO Category** | **SVM** | **PNN** | **KNN** |
| --- | --- | --- | --- | --- |
| **Molecular Function** | | | | |
| EC3.2 Hydrolases - Glycosylases | - | 98.6 | - | Y |
| All lipid-binding proteins | GO:0008289 lipid binding | 96.1 | - | - |
| Metal-binding | GO:0046872 metal ion binding | 78.4 | - | - |
| Zinc-binding | GO:0008270 zinc binding | 76.2 | - | - |
| Calcium-binding | - | 58.6 | - | - |
| **Broadly Defined Function** | | | | |
| Photosystem I | GO:0009522 photosystem I | 58.6 | ? | ? |

**FFPred**

| **Score** | **GO term** | **RL** | **Domain** | **Description** |
| --- | --- | --- | --- | --- |
| 0.952 | GO:0003824 | H | MF | catalytic activity |
| 0.759 | GO:0016740 | H | MF | transferase activity |
| 0.718 | GO:0030554 | H | MF | adenyl nucleotide binding |
| 0.691 | GO:0000166 | H | MF | nucleotide binding |
| 0.686 | GO:0019222 | H | BP | regulation of metabolic process |
| 0.655 | GO:0005576 | H | CC | extracellular region |
| 0.653 | GO:0031982 | H | CC | vesicle |
| 0.631 | GO:0016874 | H | MF | ligase activity |
| 0.624 | GO:0019637 | H | BP | organophosphate metabolic process |
| 0.619 | GO:0035639 | H | MF | purine ribonucleoside triphosphate binding |
| 0.597 | GO:0034645 | H | BP | cellular macromolecule biosynthetic process |
| 0.596 | GO:0070062 | H | CC | extracellular vesicular exosome |
| 0.595 | GO:0031988 | H | CC | membrane-bounded vesicle |
| 0.594 | GO:0044281 | H | BP | small molecule metabolic process |
| 0.585 | GO:0017076 | H | MF | purine nucleotide binding |
| 0.564 | GO:0005739 | H | CC | mitochondrion |
| 0.559 | GO:0009056 | H | BP | catabolic process |
| 0.555 | GO:0003779 | H | MF | actin binding |
| 0.534 | GO:0008092 | H | MF | cytoskeletal protein binding |
| 0.517 | GO:0003723 | H | MF | RNA binding |
| 0.514 | GO:0003676 | H | MF | nucleic acid binding |
| 0.508 | GO:0051171 | H | BP | regulation of nitrogen compound metabolic process |
| 0.941 | GO:0044237 | L | BP | cellular metabolic process |
| 0.937 | GO:0008152 | L | BP | metabolic process |
| 0.912 | GO:0005737 | L | CC | cytoplasm |
| 0.889 | GO:0006139 | L | BP | nucleobase-containing compound metabolic process |
| 0.879 | GO:0016787 | L | MF | hydrolase activity |
| 0.877 | GO:0006807 | L | BP | nitrogen compound metabolic process |
| 0.851 | GO:0050896 | L | BP | response to stimulus |
| 0.846 | GO:0009058 | L | BP | biosynthetic process |
| 0.843 | GO:0097159 | L | MF | organic cyclic compound binding |
| 0.822 | GO:0005634 | L | CC | nucleus |
| 0.821 | GO:0051716 | L | BP | cellular response to stimulus |
| 0.808 | GO:0034641 | L | BP | cellular nitrogen compound metabolic process |
| 0.803 | GO:0046483 | L | BP | heterocycle metabolic process |
| 0.797 | GO:0019538 | L | BP | protein metabolic process |
| 0.796 | GO:0006464 | L | BP | cellular protein modification process |
| 0.791 | GO:0006725 | L | BP | cellular aromatic compound metabolic process |
| 0.79 | GO:0031981 | L | CC | nuclear lumen |
| 0.784 | GO:0005829 | L | CC | cytosol |
| 0.77 | GO:0043169 | L | MF | cation binding |
| 0.754 | GO:0036094 | L | MF | small molecule binding |
| 0.747 | GO:0043229 | L | CC | intracellular organelle |
| 0.735 | GO:0005654 | L | CC | nucleoplasm |
| 0.728 | GO:0032502 | L | BP | developmental process |
| 0.723 | GO:0044267 | L | BP | cellular protein metabolic process |
| 0.69 | GO:0010467 | L | BP | gene expression |
| 0.663 | GO:0006996 | L | BP | organelle organization |
| 0.656 | GO:0043231 | L | CC | intracellular membrane-bounded organelle |
| 0.654 | GO:0046872 | L | MF | metal ion binding |
| 0.647 | GO:0032991 | L | CC | macromolecular complex |
| 0.634 | GO:0043234 | L | CC | protein complex |
| 0.626 | GO:0016810 | L | MF | hydrolase activity, acting on carbon-nitrogen (but not peptide) bonds |
| 0.625 | GO:0016070 | L | BP | RNA metabolic process |
| 0.607 | GO:0007275 | L | BP | multicellular organismal development |
| 0.577 | GO:0007154 | L | BP | cell communication |
| 0.511 | GO:0023052 | L | BP | signaling |

**Blast**

| **BLAST Matched UniProt ID & name** | | | **Identity** | **E Value** | **Score** |
| --- | --- | --- | --- | --- | --- |
| Q6YGZ1 | HPSE_MOUSE | Heparanase | 35.26 | 2.47E-74 | 249 |
| Q71RP1 | HPSE_RAT | Heparanase | 35.057 | 2.31E-73 | 247 |
| Q9Y251 | HPSE_HUMAN | Heparanase | 35.214 | 3.18E-71 | 241 |
| Q9MYY0 | HPSE_BOVIN | Heparanase | 33.333 | 5.77E-66 | 228 |
| Q90YK5 | HPSE_CHICK | Heparanase | 31.589 | 8.36E-66 | 226 |
| B2RY83 | HPSE2_MOUSE | Inactive heparanase-2 | 29.745 | 1.23E-49 | 184 |
| Q8WWQ2 | HPSE2_HUMAN | Inactive heparanase-2 | 29.38 | 7.46E-49 | 181 |
| Q9FF10 | HPSE1_ARATH | Heparanase-like protein 1 | 27.407 | 2.37E-41 | 159 |
| Q8L608 | HPSE2_ARATH | Heparanase-like protein 2 | 25 | 1.68E-32 | 133 |
| Q9LRC8 | BAGLU_SCUBA | Baicalin-beta-D-glucuronidase | 27.778 | 4.68E-29 | 122 |
| Q9FZP1 | HPSE3_ARATH | Heparanase-like protein 3 | 23.663 | 4.44E-26 | 114 |

**Protein Name: Ver3Phr**

**Predicted results:**

**SVMProt**

| **Protein Family Name** | **GO Category** | **SVM** | **PNN** | **KNN** |
| --- | --- | --- | --- | --- |
| **Molecular Function** | | | | |
| All lipid-binding proteins | GO:0008289 lipid binding | 95.2 | - | - |
| Manganese-binding | GO:0030145 manganese binding | 88.1 | ? | ? |
| Iron-binding | GO:0005506 iron binding | 86.8 | - | - |
| EC4.1 Lyases - Carbon-Carbon Lyases | - | 65.4 | - | Y |
| Zinc-binding | GO:0008270 zinc binding | 65.4 | - | - |
| **Biological Process** | | | | |
| Chromophore | GO:0018298 protein-chromophore linkage | 87.5 | - | - |

**FFPred**

| **Score** | **GO term** | **RL** | **Domain** | **Description** |
| --- | --- | --- | --- | --- |
| 0.963 | GO:0003824 | H | MF | catalytic activity |
| 0.946 | GO:0016740 | H | MF | transferase activity |
| 0.864 | GO:0005739 | H | CC | mitochondrion |
| 0.818 | GO:0017076 | H | MF | purine nucleotide binding |
| 0.809 | GO:0035639 | H | MF | purine ribonucleoside triphosphate binding |
| 0.809 | GO:0006796 | H | BP | phosphate-containing compound metabolic process |
| 0.807 | GO:0032549 | H | MF | ribonucleoside binding |
| 0.803 | GO:0019222 | H | BP | regulation of metabolic process |
| 0.802 | GO:0000166 | H | MF | nucleotide binding |
| 0.788 | GO:0006082 | H | BP | organic acid metabolic process |
| 0.768 | GO:0030554 | H | MF | adenyl nucleotide binding |
| 0.74 | GO:0001883 | H | MF | purine nucleoside binding |
| 0.717 | GO:0001882 | H | MF | nucleoside binding |
| 0.713 | GO:0016301 | H | MF | kinase activity |
| 0.696 | GO:0005524 | H | MF | ATP binding |
| 0.68 | GO:0034645 | H | BP | cellular macromolecule biosynthetic process |
| 0.665 | GO:0004674 | H | MF | protein serine/threonine kinase activity |
| 0.657 | GO:0008092 | H | MF | cytoskeletal protein binding |
| 0.656 | GO:0004672 | H | MF | protein kinase activity |
| 0.645 | GO:0044281 | H | BP | small molecule metabolic process |
| 0.602 | GO:0006810 | H | BP | transport |
| 0.594 | GO:0005975 | H | BP | carbohydrate metabolic process |
| 0.587 | GO:0005740 | H | CC | mitochondrial envelope |
| 0.585 | GO:0005576 | H | CC | extracellular region |
| 0.582 | GO:0031982 | H | CC | vesicle |
| 0.573 | GO:0070062 | H | CC | extracellular vesicular exosome |
| 0.571 | GO:0006629 | H | BP | lipid metabolic process |
| 0.548 | GO:0006468 | H | BP | protein phosphorylation |
| 0.531 | GO:0008270 | H | MF | zinc ion binding |
| 0.53 | GO:0019752 | H | BP | carboxylic acid metabolic process |
| 0.527 | GO:0009056 | H | BP | catabolic process |
| 0.517 | GO:0016310 | H | BP | phosphorylation |
| 0.517 | GO:0003723 | H | MF | RNA binding |
| 0.512 | GO:0016773 | H | MF | phosphotransferase activity, alcohol group as acceptor |
| 0.511 | GO:0051641 | H | BP | cellular localization |
| 0.952 | GO:0044237 | L | BP | cellular metabolic process |
| 0.945 | GO:0005737 | L | CC | cytoplasm |
| 0.924 | GO:0008152 | L | BP | metabolic process |
| 0.885 | GO:0043229 | L | CC | intracellular organelle |
| 0.873 | GO:0009058 | L | BP | biosynthetic process |
| 0.865 | GO:0036094 | L | MF | small molecule binding |
| 0.86 | GO:0007165 | L | BP | signal transduction |
| 0.853 | GO:0050896 | L | BP | response to stimulus |
| 0.828 | GO:0005634 | L | CC | nucleus |
| 0.824 | GO:0051716 | L | BP | cellular response to stimulus |
| 0.823 | GO:0007154 | L | BP | cell communication |
| 0.807 | GO:0034641 | L | BP | cellular nitrogen compound metabolic process |
| 0.795 | GO:0016772 | L | MF | transferase activity, transferring phosphorus-containing groups |
| 0.788 | GO:0023052 | L | BP | signaling |
| 0.788 | GO:0046483 | L | BP | heterocycle metabolic process |
| 0.781 | GO:0005829 | L | CC | cytosol |
| 0.779 | GO:0006807 | L | BP | nitrogen compound metabolic process |
| 0.775 | GO:0044267 | L | BP | cellular protein metabolic process |
| 0.763 | GO:0006725 | L | BP | cellular aromatic compound metabolic process |
| 0.761 | GO:0031981 | L | CC | nuclear lumen |
| 0.76 | GO:0043231 | L | CC | intracellular membrane-bounded organelle |
| 0.759 | GO:0097159 | L | MF | organic cyclic compound binding |
| 0.735 | GO:0032502 | L | BP | developmental process |
| 0.734 | GO:0043169 | L | MF | cation binding |
| 0.719 | GO:0046872 | L | MF | metal ion binding |
| 0.717 | GO:0006139 | L | BP | nucleobase-containing compound metabolic process |
| 0.706 | GO:0005654 | L | CC | nucleoplasm |
| 0.701 | GO:0006996 | L | BP | organelle organization |
| 0.651 | GO:0019904 | L | MF | protein domain specific binding |
| 0.651 | GO:0010467 | L | BP | gene expression |
| 0.636 | GO:0019538 | L | BP | protein metabolic process |
| 0.627 | GO:0016070 | L | BP | RNA metabolic process |
| 0.616 | GO:0006464 | L | BP | cellular protein modification process |
| 0.609 | GO:0007275 | L | BP | multicellular organismal development |
| 0.595 | GO:0005102 | L | MF | receptor binding |
| 0.56 | GO:0048856 | L | BP | anatomical structure development |
| 0.547 | GO:0032403 | L | MF | protein complex binding |
| 0.512 | GO:0010033 | L | BP | response to organic substance |
| 0.502 | GO:0071310 | L | BP | cellular response to organic substance |

**Blast**

| **BLAST Matched UniProt ID & name** | | | **Identity** | **E Value** | **Score** |
| --- | --- | --- | --- | --- | --- |
| P00914 | PHR_ECOLI | Deoxyribodipyrimidine photo-lyase | 42.616 | 1.01E-123 | 375 |
| Q9KNA8 | PHR_VIBCH | Deoxyribodipyrimidine photo-lyase | 41.3 | 7.79E-118 | 360 |
| P57386 | PHR_BUCAI | Deoxyribodipyrimidine photo-lyase | 40.546 | 2.51E-117 | 359 |
| P25078 | PHR_SALTY | Deoxyribodipyrimidine photo-lyase | 40.803 | 6.18E-117 | 358 |
| P27526 | PHR_NEUCR | Deoxyribodipyrimidine photo-lyase | 37.4 | 4.28E-109 | 342 |
| Q89AJ9 | PHR_BUCBP | Deoxyribodipyrimidine photo-lyase | 36.688 | 3.60E-103 | 323 |
| P05066 | PHR_YEAST | Deoxyribodipyrimidine photo-lyase, mitochondrial | 35.283 | 1.40E-90 | 293 |
| A9CJC9 | PHRA_AGRFC | Deoxyribodipyrimidine photo-lyase | 35.714 | 6.96E-86 | 278 |
| Q04449 | PHR_BACPE | Deoxyribodipyrimidine photo-lyase | 35.412 | 6.73E-83 | 270 |
| P05327 | PHR_SYNP6 | Deoxyribodipyrimidine photo-lyase | 35.417 | 1.78E-81 | 266 |
| Q55081 | PHR_SYNY3 | Deoxyribodipyrimidine photo-lyase | 35.931 | 3.81E-80 | 263 |
| Q9HQ46 | PHR_HALSA | Deoxyribodipyrimidine photo-lyase | 33.947 | 1.22E-75 | 251 |
| P61496 | PHR_THET2 | Deoxyribodipyrimidine photo-lyase | 31.368 | 3.67E-60 | 208 |
| P12768 | PHR_STRGR | Deoxyribodipyrimidine photo-lyase | 30.898 | 4.03E-60 | 209 |
| Q96524 | CRY2_ARATH | Cryptochrome-2 | 29.876 | 4.88E-56 | 201 |
| P61497 | PHR_THET8 | Deoxyribodipyrimidine photo-lyase | 29.717 | 1.85E-55 | 196 |
| Q43125 | CRY1_ARATH | Cryptochrome-1 | 28.243 | 1.52E-51 | 189 |
| P40115 | CRY1_SINAL | Cryptochrome-1 | 29.228 | 6.95E-51 | 185 |
| Q7UJB1 | CRYD_RHOBA | Cryptochrome DASH | 29.228 | 7.46E-51 | 185 |
| Q7NMD1 | CRYD_GLOVI | Cryptochrome DASH | 28.081 | 3.35E-49 | 181 |
| P77967 | CRYD_SYNY3 | Cryptochrome DASH | 28.66 | 1.53E-44 | 167 |
| Q5IZC5 | CRY1_ERIRU | Cryptochrome-1 | 30.123 | 6.23E-44 | 167 |
| Q8QG61 | CRY1_CHICK | Cryptochrome-1 | 30.123 | 2.54E-43 | 165 |
| Q16526 | CRY1_HUMAN | Cryptochrome-1 | 29.774 | 3.45E-43 | 165 |
| Q8WP19 | CRY1_MACFA | Cryptochrome-1 | 29.774 | 3.94E-43 | 164 |
| Q75WS4 | CRYD_XENLA | Cryptochrome DASH | 29.691 | 5.80E-43 | 163 |
| Q70AD6 | CRY1_SPAJD | Cryptochrome-1 | 29.774 | 5.95E-43 | 164 |
| Q6ZZY0 | CRY1_SYLBO | Cryptochrome-1 | 29.918 | 1.16E-42 | 163 |
| O48652 | UVR3_ARATH | (6-4)DNA photolyase | 28.866 | 3.97E-42 | 161 |
| Q3IPX9 | CRYD_NATPD | Cryptochrome DASH | 26.489 | 7.29E-42 | 159 |
| Q32Q86 | CRY1_RAT | Cryptochrome-1 | 29.363 | 1.13E-41 | 160 |
| P97784 | CRY1_MOUSE | Cryptochrome-1 | 29.363 | 1.27E-41 | 160 |
| Q7PYI7 | CRY1_ANOGA | Cryptochrome-1 | 25.097 | 4.52E-41 | 158 |
| Q923I8 | CRY2_RAT | Cryptochrome-2 | 27.975 | 5.67E-41 | 158 |
| Q49AN0 | CRY2_HUMAN | Cryptochrome-2 | 27.766 | 9.88E-41 | 157 |
| Q9R194 | CRY2_MOUSE | Cryptochrome-2 | 27.708 | 1.50E-40 | 157 |
| Q4KML2 | CRYD_DANRE | Cryptochrome DASH | 26.514 | 8.78E-39 | 151 |
| Q17DK5 | CRY1_AEDAE | Cryptochrome-1 | 26.078 | 9.03E-39 | 151 |
| Q8QG60 | CRY2_CHICK | Cryptochrome-2 | 27.366 | 1.24E-38 | 151 |
| B0WRR9 | CRY1_CULQU | Cryptochrome-1 | 27.077 | 2.74E-37 | 147 |
| Q5IFN2 | CRYD_OSTTA | Cryptochrome DASH, chloroplastic/mitochondrial | 26.253 | 7.69E-37 | 145 |
| O77059 | CRY1_DROME | Cryptochrome-1 | 24.44 | 1.22E-35 | 142 |
| Q0E2Y1 | UVR3_ORYSJ | (6-4)DNA photolyasE | 26.253 | 1.33E-35 | 142 |
| Q38JU2 | CRYD_SOLLC | Cryptochrome DASH, chloroplastic/mitochondrial | 24.089 | 2.64E-34 | 138 |
| Q293P8 | CRY1_DROPS | Cryptochrome-1 | 24.283 | 2.17E-33 | 135 |
| Q5QXE0 | CRYD_IDILO | Cryptochrome DASH | 27.494 | 6.48E-32 | 130 |
| Q9KS67 | CRY2_VIBCH | Cryptochrome-like protein cry2 | 25.826 | 9.37E-32 | 130 |
| Q7SI68 | CRYD_NEUCR | Putative cryptochrome DASH, mitochondrial | 30.797 | 8.48E-31 | 129 |
| Q87JP5 | CRYD_VIBPA | Cryptochrome DASH | 27.64 | 1.04E-27 | 117 |
| Q9KR33 | CRYD_VIBCH | Cryptochrome DASH | 26.608 | 1.97E-26 | 114 |
| Q651U1 | CRYD_ORYSJ | Cryptochrome DASH, chloroplastic/mitochondrial | 30.078 | 1.02E-25 | 113 |
| Q4I1Q6 | CRYD_GIBZE | Putative cryptochrome DASH | 23.573 | 1.12E-25 | 114 |
| Q84KJ5 | CRYD_ARATH | Cryptochrome DASH, chloroplastic/mitochondrial | 24.544 | 2.93E-25 | 112 |
| Q05380 | YAT1_SYNP1 | Uncharacterized 31.6 kDa protein in atpI 5'region | 27.397 | 5.48E-18 | 87.4 |
| Q8LB72 | PHR2_ARATH | Blue-light photoreceptor PHR2 | 21.451 | 1.52E-04 | 47.4 |
| P34205 | PHR_CARAU | Deoxyribodipyrimidine photo-lyase | 25.658 | 2.15E-04 | 47.4 |

**Protein Name: Esterase**

**Predicted results:**

**SVMProt**

| **Protein Family Name** | **GO Category** | **SVM** | **PNN** | **KNN** |
| --- | --- | --- | --- | --- |
| **Molecular Function** | | | | |
| EC2.3 Transferases - Acyltransferases | - | 92.1 | - | Y |
| EC3.5 Hydrolases - Acting on Carbon-Nitrogen Bonds, other than Peptide Bonds | - | 88.1 | - | Y |
| Iron-binding | GO:0005506 iron binding | 96.4 | - | - |
| EC4.1 Lyases - Carbon-Carbon Lyases | - | 68.5 | - | Y |
| EC2.4 Transferases - Glycosyltransferases | - | 68.5 | - | Y |
| EC3.2 Hydrolases - Glycosylases | - | 62.2 | - | Y |
| EC2.1 Transferases - Transferring One-Carbon Groups | - | 58.6 | - | Y |
| EC4.2 Lyases - Carbon-Oxygen Lyases | - | 58.6 | - | - |
| **Biological Process** | | | | |
| Chlorophyll biosynthesis | GO:0015995 chlorophyll biosynthetic process | 58.6 | ? | ? |
| DNA repair | GO:0006281 DNA repair | 58.6 | ? | ? |

**FFPred**

| **Score** | **GO term** | **RL** | **Domain** | **Description** |
| --- | --- | --- | --- | --- |
| 0.979 | GO:0003824 | H | MF | catalytic activity |
| 0.958 | GO:0016020 | H | CC | membrane |
| 0.946 | GO:0006082 | H | BP | organic acid metabolic process |
| 0.922 | GO:0044281 | H | BP | small molecule metabolic process |
| 0.886 | GO:0016740 | H | MF | transferase activity |
| 0.875 | GO:0006790 | H | BP | sulfur compound metabolic process |
| 0.828 | GO:0044255 | H | BP | cellular lipid metabolic process |
| 0.805 | GO:0055114 | H | BP | oxidation-reduction process |
| 0.804 | GO:0008610 | H | BP | lipid biosynthetic process |
| 0.797 | GO:0019637 | H | BP | organophosphate metabolic process |
| 0.789 | GO:0006629 | H | BP | lipid metabolic process |
| 0.759 | GO:0019222 | H | BP | regulation of metabolic process |
| 0.75 | GO:0032549 | H | MF | ribonucleoside binding |
| 0.746 | GO:0030554 | H | MF | adenyl nucleotide binding |
| 0.746 | GO:0005886 | H | CC | plasma membrane |
| 0.745 | GO:0017076 | H | MF | purine nucleotide binding |
| 0.743 | GO:0035639 | H | MF | purine ribonucleoside triphosphate binding |
| 0.739 | GO:0006796 | H | BP | phosphate-containing compound metabolic process |
| 0.718 | GO:0006066 | H | BP | alcohol metabolic process |
| 0.713 | GO:0000166 | H | MF | nucleotide binding |
| 0.706 | GO:0009117 | H | BP | nucleotide metabolic process |
| 0.706 | GO:0001882 | H | MF | nucleoside binding |
| 0.705 | GO:0005887 | H | CC | integral component of plasma membrane |
| 0.698 | GO:0016021 | H | CC | integral component of membrane |
| 0.696 | GO:0001883 | H | MF | purine nucleoside binding |
| 0.695 | GO:0016310 | H | BP | phosphorylation |
| 0.679 | GO:0005524 | H | MF | ATP binding |
| 0.679 | GO:0055086 | H | BP | nucleobase-containing small molecule metabolic process |
| 0.671 | GO:0070062 | H | CC | extracellular vesicular exosome |
| 0.661 | GO:0031224 | H | CC | intrinsic component of membrane |
| 0.659 | GO:0012505 | H | CC | endomembrane system |
| 0.658 | GO:0005739 | H | CC | mitochondrion |
| 0.648 | GO:0098588 | H | CC | bounding membrane of organelle |
| 0.638 | GO:0005783 | H | CC | endoplasmic reticulum |
| 0.614 | GO:0051171 | H | BP | regulation of nitrogen compound metabolic process |
| 0.603 | GO:0032787 | H | BP | monocarboxylic acid metabolic process |
| 0.6 | GO:0010468 | H | BP | regulation of gene expression |
| 0.589 | GO:0031982 | H | CC | vesicle |
| 0.587 | GO:0046914 | H | MF | transition metal ion binding |
| 0.587 | GO:0031090 | H | CC | organelle membrane |
| 0.569 | GO:0009056 | H | BP | catabolic process |
| 0.565 | GO:0009116 | H | BP | nucleoside metabolic process |
| 0.563 | GO:0009059 | H | BP | macromolecule biosynthetic process |
| 0.549 | GO:0005789 | H | CC | endoplasmic reticulum membrane |
| 0.548 | GO:0019752 | H | BP | carboxylic acid metabolic process |
| 0.547 | GO:0006811 | H | BP | ion transport |
| 0.532 | GO:0006631 | H | BP | fatty acid metabolic process |
| 0.53 | GO:0071944 | H | CC | cell periphery |
| 0.523 | GO:0006810 | H | BP | transport |
| 0.978 | GO:0008152 | L | BP | metabolic process |
| 0.953 | GO:0044237 | L | BP | cellular metabolic process |
| 0.936 | GO:0043229 | L | CC | intracellular organelle |
| 0.922 | GO:0005737 | L | CC | cytoplasm |
| 0.918 | GO:0009058 | L | BP | biosynthetic process |
| 0.906 | GO:0043231 | L | CC | intracellular membrane-bounded organelle |
| 0.88 | GO:0050896 | L | BP | response to stimulus |
| 0.863 | GO:0044267 | L | BP | cellular protein metabolic process |
| 0.844 | GO:0019538 | L | BP | protein metabolic process |
| 0.817 | GO:0051716 | L | BP | cellular response to stimulus |
| 0.81 | GO:0006807 | L | BP | nitrogen compound metabolic process |
| 0.776 | GO:0006464 | L | BP | cellular protein modification process |
| 0.749 | GO:0097159 | L | MF | organic cyclic compound binding |
| 0.741 | GO:0036094 | L | MF | small molecule binding |
| 0.718 | GO:0032502 | L | BP | developmental process |
| 0.698 | GO:0043169 | L | MF | cation binding |
| 0.697 | GO:0023052 | L | BP | signaling |
| 0.69 | GO:0043234 | L | CC | protein complex |
| 0.661 | GO:0016787 | L | MF | hydrolase activity |
| 0.657 | GO:0009893 | L | BP | positive regulation of metabolic process |
| 0.647 | GO:0034641 | L | BP | cellular nitrogen compound metabolic process |
| 0.638 | GO:0007275 | L | BP | multicellular organismal development |
| 0.63 | GO:0007165 | L | BP | signal transduction |
| 0.627 | GO:0006139 | L | BP | nucleobase-containing compound metabolic process |
| 0.622 | GO:0007154 | L | BP | cell communication |
| 0.589 | GO:0005634 | L | CC | nucleus |
| 0.567 | GO:0046483 | L | BP | heterocycle metabolic process |
| 0.565 | GO:0006725 | L | BP | cellular aromatic compound metabolic process |
| 0.55 | GO:0005794 | L | CC | Golgi apparatus |
| 0.521 | GO:0031325 | L | BP | positive regulation of cellular metabolic process |
| 0.506 | GO:0006996 | L | BP | organelle organization |
| 0.502 | GO:0046872 | L | MF | metal ion binding |

**Blast**

| **BLAST Matched UniProt ID & name** | | | **Identity** | **E Value** | **Score** |
| --- | --- | --- | --- | --- | --- |
| Q09621 | LACT2_CAEEL | Beta-lactamase domain-containing protein 2 | 30.239 | 2.19E-32 | 131 |

**Protein Name: Rhodobacter sphaeroides 2.4.1 chromosome 1**

**Predicted results:**

**SVMProt**

| **Protein Family Name** | **GO Category** | **SVM** | **PNN** | **KNN** |
| --- | --- | --- | --- | --- |
| **Molecular Function** | | | | |
| EC2.4 Transferases - Glycosyltransferases | - | 89.3 | - | Y |
| All lipid-binding proteins | GO:0008289 lipid binding | 65.4 | - | - |
| TC3.A.1 ATP-binding cassette (ABC) family | - | 58.6 | ? | ? |
| Magnesium-binding | GO:0000287 magnesium binding | 58.6 | ? | ? |

**FFPred**

| **Score** | **GO term** | **RL** | **Domain** | **Description** |
| --- | --- | --- | --- | --- |
| 0.993 | GO:0016021 | H | CC | integral component of membrane |
| 0.992 | GO:0031224 | H | CC | intrinsic component of membrane |
| 0.963 | GO:0016020 | H | CC | membrane |
| 0.951 | GO:0070062 | H | CC | extracellular vesicular exosome |
| 0.95 | GO:0003824 | H | MF | catalytic activity |
| 0.945 | GO:0005887 | H | CC | integral component of plasma membrane |
| 0.92 | GO:0005576 | H | CC | extracellular region |
| 0.869 | GO:0005886 | H | CC | plasma membrane |
| 0.861 | GO:0031982 | H | CC | vesicle |
| 0.828 | GO:0006810 | H | BP | transport |
| 0.813 | GO:0005125 | H | MF | cytokine activity |
| 0.8 | GO:0031226 | H | CC | intrinsic component of plasma membrane |
| 0.795 | GO:0071944 | H | CC | cell periphery |
| 0.753 | GO:0042175 | H | CC | nuclear outer membrane-endoplasmic reticulum membrane network |
| 0.752 | GO:0006955 | H | BP | immune response |
| 0.746 | GO:0005789 | H | CC | endoplasmic reticulum membrane |
| 0.745 | GO:0098588 | H | CC | bounding membrane of organelle |
| 0.732 | GO:0031966 | H | CC | mitochondrial membrane |
| 0.726 | GO:0012505 | H | CC | endomembrane system |
| 0.714 | GO:0005615 | H | CC | extracellular space |
| 0.672 | GO:0055114 | H | BP | oxidation-reduction process |
| 0.649 | GO:0031988 | H | CC | membrane-bounded vesicle |
| 0.642 | GO:0031090 | H | CC | organelle membrane |
| 0.625 | GO:0005126 | H | MF | cytokine receptor binding |
| 0.614 | GO:0007166 | H | BP | cell surface receptor signaling pathway |
| 0.612 | GO:0005740 | H | CC | mitochondrial envelope |
| 0.606 | GO:0016491 | H | MF | oxidoreductase activity |
| 0.578 | GO:0005783 | H | CC | endoplasmic reticulum |
| 0.563 | GO:0019222 | H | BP | regulation of metabolic process |
| 0.557 | GO:0046914 | H | MF | transition metal ion binding |
| 0.552 | GO:0044281 | H | BP | small molecule metabolic process |
| 0.549 | GO:0031301 | H | CC | integral component of organelle membrane |
| 0.535 | GO:0005743 | H | CC | mitochondrial inner membrane |
| 0.529 | GO:0005739 | H | CC | mitochondrion |
| 0.524 | GO:0000139 | H | CC | Golgi membrane |
| 0.524 | GO:0008083 | H | MF | growth factor activity |
| 0.514 | GO:0051641 | H | BP | cellular localization |
| 0.506 | GO:0016740 | H | MF | transferase activity |
| 0.503 | GO:0006952 | H | BP | defense response |
| 0.915 | GO:0050896 | L | BP | response to stimulus |
| 0.885 | GO:0005737 | L | CC | cytoplasm |
| 0.878 | GO:0043229 | L | CC | intracellular organelle |
| 0.869 | GO:0008152 | L | BP | metabolic process |
| 0.868 | GO:0005102 | L | MF | receptor binding |
| 0.854 | GO:0043231 | L | CC | intracellular membrane-bounded organelle |
| 0.837 | GO:0051716 | L | BP | cellular response to stimulus |
| 0.816 | GO:0006807 | L | BP | nitrogen compound metabolic process |
| 0.797 | GO:0032502 | L | BP | developmental process |
| 0.795 | GO:0007154 | L | BP | cell communication |
| 0.788 | GO:0044267 | L | BP | cellular protein metabolic process |
| 0.779 | GO:0044237 | L | BP | cellular metabolic process |
| 0.777 | GO:0016787 | L | MF | hydrolase activity |
| 0.757 | GO:0023052 | L | BP | signaling |
| 0.746 | GO:0019538 | L | BP | protein metabolic process |
| 0.742 | GO:0046872 | L | MF | metal ion binding |
| 0.741 | GO:0043169 | L | MF | cation binding |
| 0.734 | GO:0007165 | L | BP | signal transduction |
| 0.694 | GO:0048856 | L | BP | anatomical structure development |
| 0.666 | GO:0043234 | L | CC | protein complex |
| 0.653 | GO:0009058 | L | BP | biosynthetic process |
| 0.646 | GO:0032991 | L | CC | macromolecular complex |
| 0.639 | GO:0009893 | L | BP | positive regulation of metabolic process |
| 0.629 | GO:0007275 | L | BP | multicellular organismal development |
| 0.625 | GO:0009966 | L | BP | regulation of signal transduction |
| 0.556 | GO:0032403 | L | MF | protein complex binding |
| 0.555 | GO:0030154 | L | BP | cell differentiation |
| 0.546 | GO:0042127 | L | BP | regulation of cell proliferation |
| 0.545 | GO:0006464 | L | BP | cellular protein modification process |
| 0.516 | GO:0034641 | L | BP | cellular nitrogen compound metabolic process |
| 0.508 | GO:0010033 | L | BP | response to organic substance |
| 0.503 | GO:0006139 | L | BP | nucleobase-containing compound metabolic process |

**Protein Name: Agarase**

**Predicted results:**

**SVMProt**

| **Protein Family Name** | **GO Category** | **SVM** | **PNN** | **KNN** |
| --- | --- | --- | --- | --- |
| **Molecular Function** | | | | |
| EC3.2 Hydrolases - Glycosylases | - | 82.2 | - | Y |
| Metal-binding | GO:0046872 metal ion binding | 68.5 | - | - |
| All lipid-binding proteins | GO:0008289 lipid binding | 62.2 | - | - |
| **Biological Process** | | | | |
| Virulence | GO:0009405 pathogenesis | 65.4 | ? | ? |

**FFPred**

| **Score** | **GO term** | **RL** | **Domain** | **Description** |
| --- | --- | --- | --- | --- |
| 0.925 | GO:0070062 | H | CC | extracellular vesicular exosome |
| 0.902 | GO:0005576 | H | CC | extracellular region |
| 0.899 | GO:0005615 | H | CC | extracellular space |
| 0.855 | GO:0003824 | H | MF | catalytic activity |
| 0.796 | GO:0006810 | H | BP | transport |
| 0.786 | GO:0031982 | H | CC | vesicle |
| 0.779 | GO:0030203 | H | BP | glycosaminoglycan metabolic process |
| 0.774 | GO:0098609 | H | BP | cell-cell adhesion |
| 0.712 | GO:0005509 | H | MF | calcium ion binding |
| 0.707 | GO:0002684 | H | BP | positive regulation of immune system process |
| 0.704 | GO:0030198 | H | BP | extracellular matrix organization |
| 0.701 | GO:0005886 | H | CC | plasma membrane |
| 0.697 | GO:0016020 | H | CC | membrane |
| 0.688 | GO:0044281 | H | BP | small molecule metabolic process |
| 0.684 | GO:0019222 | H | BP | regulation of metabolic process |
| 0.68 | GO:0012505 | H | CC | endomembrane system |
| 0.676 | GO:0050839 | H | MF | cell adhesion molecule binding |
| 0.669 | GO:0071944 | H | CC | cell periphery |
| 0.664 | GO:0031988 | H | CC | membrane-bounded vesicle |
| 0.649 | GO:0004872 | H | MF | receptor activity |
| 0.638 | GO:0004871 | H | MF | signal transducer activity |
| 0.634 | GO:0043062 | H | BP | extracellular structure organization |
| 0.619 | GO:0038023 | H | MF | signaling receptor activity |
| 0.61 | GO:0007155 | H | BP | cell adhesion |
| 0.574 | GO:0006952 | H | BP | defense response |
| 0.549 | GO:0002376 | H | BP | immune system process |
| 0.535 | GO:0030234 | H | MF | enzyme regulator activity |
| 0.532 | GO:0051171 | H | BP | regulation of nitrogen compound metabolic process |
| 0.532 | GO:0016192 | H | BP | vesicle-mediated transport |
| 0.517 | GO:0004866 | H | MF | endopeptidase inhibitor activity |
| 0.511 | GO:0007166 | H | BP | cell surface receptor signaling pathway |
| 0.501 | GO:0002682 | H | BP | regulation of immune system process |
| 0.501 | GO:0006955 | H | BP | immune response |
| 0.94 | GO:0008152 | L | BP | metabolic process |
| 0.909 | GO:0050896 | L | BP | response to stimulus |
| 0.865 | GO:0046872 | L | MF | metal ion binding |
| 0.853 | GO:0005102 | L | MF | receptor binding |
| 0.832 | GO:0051716 | L | BP | cellular response to stimulus |
| 0.831 | GO:0043169 | L | MF | cation binding |
| 0.816 | GO:0019538 | L | BP | protein metabolic process |
| 0.813 | GO:0048856 | L | BP | anatomical structure development |
| 0.791 | GO:0032403 | L | MF | protein complex binding |
| 0.774 | GO:0007275 | L | BP | multicellular organismal development |
| 0.722 | GO:0032502 | L | BP | developmental process |
| 0.703 | GO:0023052 | L | BP | signaling |
| 0.697 | GO:0007154 | L | BP | cell communication |
| 0.68 | GO:0006807 | L | BP | nitrogen compound metabolic process |
| 0.67 | GO:0048513 | L | BP | organ development |
| 0.664 | GO:0005737 | L | CC | cytoplasm |
| 0.659 | GO:0016787 | L | MF | hydrolase activity |
| 0.649 | GO:0030154 | L | BP | cell differentiation |
| 0.615 | GO:0009605 | L | BP | response to external stimulus |
| 0.603 | GO:0043231 | L | CC | intracellular membrane-bounded organelle |
| 0.579 | GO:0007165 | L | BP | signal transduction |
| 0.573 | GO:0043229 | L | CC | intracellular organelle |
| 0.565 | GO:0050790 | L | BP | regulation of catalytic activity |
| 0.561 | GO:0045595 | L | BP | regulation of cell differentiation |
| 0.553 | GO:0051246 | L | BP | regulation of protein metabolic process |
| 0.548 | GO:0010033 | L | BP | response to organic substance |
| 0.546 | GO:0009893 | L | BP | positive regulation of metabolic process |
| 0.529 | GO:0009966 | L | BP | regulation of signal transduction |
| 0.505 | GO:0009888 | L | BP | tissue development |
| 0.5 | GO:0030054 | L | CC | cell junction |

**Blast**

| **BLAST Matched UniProt ID & name** | | | **Identity** | **E Value** | **Score** |
| --- | --- | --- | --- | --- | --- |
| B5CY73 | BAGA_BACPM | Beta-agarase | 49.515 | 1.98E-94 | 298 |
| Q9RGX8 | AGAB_ZOBGA | Beta-agarase B | 47.157 | 2.14E-90 | 289 |
| G0L322 | AGAA_ZOBGA | Beta-agarase A | 47.917 | 3.51E-80 | 268 |
| A8W969 | AGB34_AGAAL | Beta-agarase AgaB34 | 41.768 | 4.33E-67 | 231 |
| D7GXG4 | AGAD_ZOBGA | Beta-agarase D | 33.915 | 1.19E-56 | 203 |
| P07883 | AGAR_STRCO | Extracellular agarase | 35.172 | 8.39E-48 | 174 |
| A1IGV8 | AAGAR_THASX | Alpha-agarase | 43.75 | 1.09E-27 | 122 |
| A1IGV8 | AAGAR_THASX | Alpha-agarase | 31.818 | 2.16E-13 | 77 |
| A1IGV8 | AAGAR_THASX | Alpha-agarase | 35.664 | 6.66E-13 | 75.5 |
| Q9LAP7 | AAGAR_ALTAG | Alpha-agarase | 43.571 | 3.17E-23 | 108 |
| Q9LAP7 | AAGAR_ALTAG | Alpha-agarase | 36.667 | 2.42E-15 | 83.2 |
| Q9LAP7 | AAGAR_ALTAG | Alpha-agarase | 30.719 | 2.29E-11 | 70.5 |
| D7GXG0 | PORA_ZOBGA | Beta-porphyranase A | 27.326 | 4.75E-14 | 78.2 |
| D7GXG3 | PORE_ZOBGA | Beta-porphyranase E | 28.821 | 5.58E-14 | 75.9 |
| B5CY92 | PORB_BACPM | Beta-porphyranase B | 27.381 | 2.76E-11 | 68.6 |
| D7GXG5 | AGAC_ZOBGA | Beta-agarase C | 25.887 | 1.32E-10 | 66.6 |
| D7GXF9 | PORB_ZOBGA | Beta-porphyranase B | 25.128 | 1.22E-04 | 47.8 |
| D7GXG2 | PORD_ZOBGA | Beta-porphyranase D | 23.125 | 2.35E-04 | 47.4 |

**Protein Name: Manganese superoxide dismutase MnSOD1**

**Predicted results:**

**SVMProt**

| **Protein Family Name** | **GO Category** | **SVM** | **PNN** | **KNN** |
| --- | --- | --- | --- | --- |
| **Molecular Function** | | | | |
| EC3.1 Hydrolases - Acting on Ester Bonds | - | 92.1 | - | Y |
| EC1.15 Oxidoreductases - Acting on superoxide as acceptor | - | 98.8 | ? | ? |
| Manganese-binding | GO:0030145 manganese binding | 91.3 | ? | ? |
| Metal-binding | GO:0046872 metal ion binding | 71.3 | - | - |
| **Broadly Defined Function** | | | | |
| Outer membrane | GO:0009279 cell outer membrane | 58.6 | - | Y |

**FFPred**

| **Score** | **GO term** | **RL** | **Domain** | **Description** |
| --- | --- | --- | --- | --- |
| 0.782 | GO:0070062 | H | CC | extracellular vesicular exosome |
| 0.752 | GO:0019222 | H | BP | regulation of metabolic process |
| 0.734 | GO:0005576 | H | CC | extracellular region |
| 0.714 | GO:0003824 | H | MF | catalytic activity |
| 0.693 | GO:0005739 | H | CC | mitochondrion |
| 0.65 | GO:0051171 | H | BP | regulation of nitrogen compound metabolic process |
| 0.635 | GO:0005615 | H | CC | extracellular space |
| 0.611 | GO:0031982 | H | CC | vesicle |
| 0.601 | GO:0006796 | H | BP | phosphate-containing compound metabolic process |
| 0.59 | GO:0012505 | H | CC | endomembrane system |
| 0.58 | GO:0048037 | H | MF | cofactor binding |
| 0.532 | GO:0031090 | H | CC | organelle membrane |
| 0.526 | GO:0002376 | H | BP | immune system process |
| 0.525 | GO:0005783 | H | CC | endoplasmic reticulum |
| 0.524 | GO:0001664 | H | MF | G-protein coupled receptor binding |
| 0.513 | GO:0007166 | H | BP | cell surface receptor signaling pathway |
| 0.905 | GO:0005737 | L | CC | cytoplasm |
| 0.896 | GO:0043169 | L | MF | cation binding |
| 0.896 | GO:0050896 | L | BP | response to stimulus |
| 0.849 | GO:0043231 | L | CC | intracellular membrane-bounded organelle |
| 0.833 | GO:0044237 | L | BP | cellular metabolic process |
| 0.831 | GO:0046872 | L | MF | metal ion binding |
| 0.827 | GO:0051716 | L | BP | cellular response to stimulus |
| 0.8 | GO:0043229 | L | CC | intracellular organelle |
| 0.777 | GO:0005102 | L | MF | receptor binding |
| 0.754 | GO:0048856 | L | BP | anatomical structure development |
| 0.747 | GO:0032502 | L | BP | developmental process |
| 0.733 | GO:0007165 | L | BP | signal transduction |
| 0.733 | GO:0007275 | L | BP | multicellular organismal development |
| 0.708 | GO:0008152 | L | BP | metabolic process |
| 0.708 | GO:0009966 | L | BP | regulation of signal transduction |
| 0.695 | GO:0009058 | L | BP | biosynthetic process |
| 0.674 | GO:0023052 | L | BP | signaling |
| 0.645 | GO:0019538 | L | BP | protein metabolic process |
| 0.639 | GO:0007154 | L | BP | cell communication |
| 0.637 | GO:0009893 | L | BP | positive regulation of metabolic process |
| 0.591 | GO:0048513 | L | BP | organ development |
| 0.587 | GO:0030154 | L | BP | cell differentiation |
| 0.579 | GO:0043234 | L | CC | protein complex |
| 0.544 | GO:0034641 | L | BP | cellular nitrogen compound metabolic process |
| 0.542 | GO:1902531 | L | BP | regulation of intracellular signal transduction |
| 0.538 | GO:0010033 | L | BP | response to organic substance |
| 0.533 | GO:0009605 | L | BP | response to external stimulus |
| 0.517 | GO:0032991 | L | CC | macromolecular complex |
| 0.511 | GO:0005634 | L | CC | nucleus |

**Blast**

| **BLAST Matched UniProt ID & name** | | | **Identity** | **E Value** | **Score** |
| --- | --- | --- | --- | --- | --- |
| Q59094 | SODM_ACIAD | Superoxide dismutase [Mn] | 53.947 | 5.06E-86 | 261 |
| P50058 | SODM1_LEPBY | Superoxide dismutase [Mn] 1 | 51.628 | 5.11E-76 | 236 |
| P28760 | SODM_BACCA | Superoxide dismutase [Mn] | 51.691 | 6.33E-73 | 226 |
| P00449 | SODM_GEOSE | Superoxide dismutase [Mn] | 52.174 | 6.98E-73 | 226 |
| P54375 | SODM_BACSU | Superoxide dismutase [Mn] | 55.276 | 8.74E-72 | 223 |
| P28764 | SODM_LISMO | Superoxide dismutase [Mn] | 53.769 | 9.84E-72 | 223 |
| P61502 | SODM_THET2 | Superoxide dismutase [Mn] | 55.882 | 9.93E-72 | 223 |
| Q92BR6 | SODM_LISIN | Superoxide dismutase [Mn] | 53.769 | 3.24E-71 | 222 |
| Q838I4 | SODM_ENTFA | Superoxide dismutase [Fe] | 52.764 | 1.66E-70 | 220 |
| P50059 | SODM2_LEPBY | Superoxide dismutase [Mn] 2 | 49.756 | 1.06E-69 | 218 |
| Q49XZ6 | SODM_STAS1 | Superoxide dismutase [Mn/Fe] | 53.695 | 1.97E-69 | 218 |
| Q9RUV2 | SODM_DEIRA | Superoxide dismutase [Mn] | 53.431 | 2.10E-69 | 218 |
| Q9F326 | SODM_STACA | Superoxide dismutase [Mn/Fe] | 53.171 | 2.27E-69 | 217 |
| P28763 | SODM_LISIV | Superoxide dismutase [Mn] | 53 | 3.64E-69 | 217 |
| Q9K4V3 | SODM_STAXY | Superoxide dismutase [Mn/Fe] | 53.202 | 5.01E-69 | 216 |
| P53652 | SODM_PSEAE | Superoxide dismutase [Mn] | 51 | 5.92E-69 | 216 |
| Q9KD10 | SODM_BACHD | Superoxide dismutase [Mn] | 53.922 | 9.76E-69 | 216 |
| Q8VQ15 | SODM_STAEP | Superoxide dismutase [Mn/Fe] | 53.202 | 9.90E-69 | 216 |
| Q81LW0 | SODM1_BACAN | Superoxide dismutase [Mn] 1 | 52.261 | 1.62E-68 | 215 |
| Q5HNZ5 | SODM_STAEQ | Superoxide dismutase [Mn/Fe] | 53.5 | 2.65E-68 | 214 |
| Q4L6Q3 | SODM_STAHJ | Superoxide dismutase [Mn/Fe] | 53.5 | 4.56E-68 | 214 |
| P53653 | SODM_THEAQ | Superoxide dismutase [Mn] | 53.431 | 1.28E-67 | 213 |
| Q818I1 | SODM1_BACCR | Superoxide dismutase [Mn] 1 | 52.02 | 3.25E-67 | 212 |
| Q7SIC3 | SODM_VIRHA | Superoxide dismutase [Mn] | 50.485 | 8.34E-67 | 211 |
| P23744 | SODF_METJ | Superoxide dismutase [Mn/Fe] | 52.5 | 4.27E-66 | 209 |
| Q6G913 | SODM1_STAAS | Superoxide dismutase [Mn/Fe] 1 | 51.5 | 5.05E-66 | 209 |
| P0C0F9 | SODM_XANC8 | Superoxide dismutase [Mn] | 49.749 | 2.31E-65 | 207 |
| P43725 | SODM_HAEIN | Superoxide dismutase [Mn] | 51.244 | 5.78E-64 | 204 |
| P0DF72 | SODM_STRP3 | Superoxide dismutase [Mn] | 50 | 3.68E-63 | 201 |
| Q8P0D4 | SODM_STRP8 | Superoxide dismutase [Mn] | 49.749 | 4.93E-63 | 201 |
| P53655 | SODM_YEREN | Superoxide dismutase [Mn] | 49.254 | 9.83E-63 | 201 |
| P0C0I0 | SODM_STRPY | Superoxide dismutase [Mn] | 49.749 | 9.94E-63 | 200 |
| P0C0I1 | SODM_STRP1 | Superoxide dismutase [Mn] | 49.246 | 1.02E-62 | 200 |
| Q5XBF8 | SODM_STRP6 | Superoxide dismutase [Mn] | 49.749 | 1.04E-62 | 200 |
| Q2YUU9 | SODM2_STAAB | Superoxide dismutase [Mn/Fe] 2 | 48.276 | 1.89E-62 | 199 |
| P66831 | SODM2_STAAN | Superoxide dismutase [Mn/Fe] 2 | 48.276 | 3.69E-62 | 199 |
| Q42684 | SODM_CHLRE | Superoxide dismutase [Mn], mitochondrial | 47.805 | 3.91E-62 | 199 |
| P77929 | SODM_PSEPU | Superoxide dismutase [Mn] | 49 | 1.01E-61 | 198 |
| P09738 | SODM_STRMU | Superoxide dismutase [Mn/Fe] | 52.239 | 1.07E-61 | 197 |
| P50060 | SODM3_LEPBY | Superoxide dismutase [Mn] 3 | 47.783 | 8.54E-61 | 197 |
| P0A4J6 | SODM_STRPN | Superoxide dismutase [Mn] | 51.01 | 3.69E-60 | 194 |
| P66829 | SODM_SHIFL | Superoxide dismutase [Mn] | 49.495 | 9.44E-60 | 193 |
| Q8Z2V9 | SODM_SALTI | Superoxide dismutase [Mn] | 49.495 | 9.54E-60 | 193 |
| Q9CPN6 | SODM_PASMU | Superoxide dismutase [Mn] | 47.783 | 1.19E-59 | 193 |
| P43019 | SODM_SALTY | Superoxide dismutase [Mn] | 49.495 | 1.78E-59 | 192 |
| P00448 | SODM_ECOLI | Superoxide dismutase [Mn] | 48.99 | 1.80E-59 | 192 |
| P0A4J3 | SODM_STRA3 | Superoxide dismutase [Mn/Fe] | 50 | 6.50E-59 | 191 |
| Q814I6 | SODM2_BACCR | Superoxide dismutase [Mn] 2 | 43.062 | 4.14E-58 | 189 |
| Q59679 | SODM_MANHA | Superoxide dismutase [Mn] | 50 | 7.47E-58 | 188 |
| Q81JK8 | SODM2_BACAN | Superoxide dismutase [Mn] 2 | 43.062 | 9.28E-58 | 188 |
| P53642 | SODM_BORPE | Superoxide dismutase [Mn] | 46.535 | 9.62E-58 | 188 |
| O30826 | SODM_HAEDU | Superoxide dismutase [Mn] | 48.529 | 3.24E-56 | 184 |
| P57286 | SODM_BUCAI | Superoxide dismutase [Mn] | 44.221 | 7.96E-56 | 182 |
| P22799 | SODM_RAOPL | Superoxide dismutase [Fe] | 54.762 | 2.17E-55 | 180 |
| Q8K9V4 | SODM_BUCAP | Superoxide dismutase [Mn] | 44.724 | 3.33E-53 | 176 |
| P19665 | SODF_PORGI | Superoxide dismutase [Mn/Fe] | 45.026 | 3.68E-53 | 175 |
| O30563 | SODM_BORBU | Superoxide dismutase [Fe] | 44.175 | 8.49E-53 | 175 |
| Q9UQX0 | SODM_SCHPO | Superoxide dismutase [Mn], mitochondrial | 47.449 | 9.48E-53 | 175 |
| Q8HXP6 | SODM_PONPY | Superoxide dismutase [Mn], mitochondrial | 48.223 | 1.69E-52 | 175 |
| P04179 | SODM_HUMAN | Superoxide dismutase [Mn], mitochondrial | 48.223 | 1.80E-52 | 174 |
| P19685 | SODF_COXBU | Superoxide dismutase [Fe] | 43.147 | 2.09E-52 | 174 |
| Q8HXP7 | SODM_PANTR | Superoxide dismutase [Mn], mitochondrial | 47.716 | 2.37E-52 | 174 |
| Q8HXP3 | SODM_MACFA | Superoxide dismutase [Mn], mitochondrial | 47.716 | 4.11E-52 | 174 |
| P77968 | SODF_SYNY3 | Superoxide dismutase [Fe] | 44.67 | 4.31E-52 | 173 |
| Q8HXP2 | SODM_MACMU | Superoxide dismutase [Mn], mitochondrial | 47.208 | 4.97E-52 | 172 |
| Q8HXP0 | SODM_CALJA | Superoxide dismutase [Mn], mitochondrial | 47.423 | 5.13E-52 | 172 |
| P41976 | SODM_BOVIN | Superoxide dismutase [Mn], mitochondrial | 46.734 | 6.66E-52 | 173 |
| Q9XS41 | SODM_HORSE | Superoxide dismutase [Mn], mitochondrial | 48.223 | 7.03E-52 | 173 |
| Q8HXP5 | SODM_HYLLA | Superoxide dismutase [Mn], mitochondrial | 47.208 | 8.60E-52 | 172 |
| Q89AR7 | SODM_BUCBP | Superoxide dismutase [Mn] | 42.132 | 1.53E-51 | 172 |
| P50061 | SODF_LEPBY | Superoxide dismutase [Fe] | 46.193 | 2.11E-51 | 171 |
| P0A4J1 | SODM_LACLA | Superoxide dismutase [Mn] | 44.811 | 2.48E-51 | 171 |
| Q5FB30 | SODM_MACNE | Superoxide dismutase [Mn], mitochondrial | 47.208 | 3.72E-50 | 169 |
| P53638 | SODF_BACFR | Superoxide dismutase [Fe] | 43.878 | 7.22E-50 | 167 |
| P09671 | SODM_MOUSE | Superoxide dismutase [Mn], mitochondrial | 46.04 | 9.44E-50 | 167 |
| Q9SM64 | SODM_PRUPE | Superoxide dismutase [Mn], mitochondrial | 44.397 | 1.02E-49 | 167 |
| P07895 | SODM_RAT | Superoxide dismutase [Mn], mitochondrial | 46.701 | 1.32E-49 | 167 |
| Q43008 | SODM_ORYSJ | Superoxide dismutase [Mn], mitochondrial | 47.087 | 6.50E-49 | 166 |
| P41982 | SODM_RABIT | Superoxide dismutase [Mn], mitochondrial | 46.875 | 7.74E-49 | 164 |
| O30970 | SODF_RHOCA | Superoxide dismutase [Fe] | 44.335 | 1.24E-48 | 164 |
| P11796 | SODM_NICPL | Superoxide dismutase [Mn], mitochondrial | 43.534 | 1.25E-48 | 165 |
| P53641 | SODF_PSEAE | Superoxide dismutase [Fe] | 41.837 | 1.32E-48 | 164 |
| Q88PD5 | SODF_PSEPK | Superoxide dismutase [Fe] | 40.609 | 1.51E-48 | 164 |
| P09223 | SODF_PSEPU | Superoxide dismutase [Fe] | 40.609 | 1.66E-48 | 164 |
| P31108 | SODF_LEGPH | Superoxide dismutase [Fe] | 40.722 | 1.91E-48 | 163 |
| P37369 | SODF_BORPE | Superoxide dismutase [Fe] | 46.237 | 2.15E-48 | 163 |
| P09233 | SODM1_MAIZE | Superoxide dismutase [Mn] 3.1, mitochondrial | 46.829 | 5.13E-48 | 164 |
| P84612 | SODF_PSEHT | Superoxide dismutase [Fe] | 42.347 | 9.06E-48 | 161 |
| P41978 | SODM2_MAIZE | Superoxide dismutase [Mn] 3.2, mitochondrial | 45.588 | 1.78E-47 | 162 |
| P22302 | SODF_NICPL | Superoxide dismutase [Fe], chloroplastic | 41.667 | 1.87E-47 | 161 |
| P0A2F4 | SODF_SALTY | Superoxide dismutase [Fe] | 42.784 | 2.04E-47 | 160 |
| Q00637 | SODM_DROME | Superoxide dismutase [Mn], mitochondrial | 44.67 | 2.15E-47 | 161 |
| P0AGD5 | SODF_ECO57 | Superoxide dismutase [Fe] | 42.268 | 3.71E-47 | 160 |
| P41980 | SODM4_MAIZE | Superoxide dismutase [Mn] 3.4, mitochondrial | 46.341 | 5.55E-47 | 160 |
| P35017 | SODM_HEVBR | Superoxide dismutase [Mn], mitochondrial | 44.643 | 1.07E-46 | 160 |
| O81235 | SODM1_ARATH | Superoxide dismutase [Mn] 1, mitochondrial | 40.833 | 1.92E-46 | 159 |
| P41979 | SODM3_MAIZE | Superoxide dismutase [Mn] 3.3, mitochondrial | 44.878 | 3.10E-46 | 159 |
| P42821 | SODM_CORDI | Superoxide dismutase [Mn] | 43.147 | 3.91E-46 | 157 |
| O49066 | SODM_CAPAN | Superoxide dismutase [Mn], mitochondrial | 41.485 | 4.38E-46 | 158 |
| Q8YSZ1 | SODF_NOSS1 | Superoxide dismutase [Fe] | 42.439 | 6.94E-46 | 157 |

**Protein Name: Manganese superoxide dismutase MnSOD2**

**Predicted results:**

**SVMProt**

| **Protein Family Name** | **GO Category** | **SVM** | **PNN** | **KNN** |
| --- | --- | --- | --- | --- |
| **Molecular Function** | | | | |
| EC1.15 Oxidoreductases - Acting on superoxide as acceptor | - | 98.9 | ? | ? |
| Manganese-binding | GO:0030145 manganese binding | 98.3 | ? | ? |
| Zinc-binding | GO:0008270 zinc binding | 97.7 | - | - |
| Iron-binding | GO:0005506 iron binding | 92.1 | - | - |
| EC2.1 Transferases - Transferring One-Carbon Groups | - | 58.6 | - | Y |
| All DNA-binding | GO:0003677 DNA binding | 65.4 | ? | ? |
| EC3.4 Hydrolases - Acting on peptide bonds (Peptidases) | - | 62.2 | - | - |
| Magnesium-binding | GO:0000287 magnesium binding | 58.6 | ? | ? |
| Actin binding | GO:0003779 actin binding | 58.6 | ? | ? |
| Calcium-binding | - | 58.6 | - | - |
| Metal-binding | GO:0046872 metal ion binding | 58.6 | - | - |
| **Broadly Defined Function** | | | | |
| Outer membrane | GO:0009279 cell outer membrane | 58.6 | - | - |

**FFPred**

| **Score** | **GO term** | **RL** | **Domain** | **Description** |
| --- | --- | --- | --- | --- |
| 0.904 | GO:0070062 | H | CC | extracellular vesicular exosome |
| 0.891 | GO:0003824 | H | MF | catalytic activity |
| 0.883 | GO:0006811 | H | BP | ion transport |
| 0.841 | GO:0031982 | H | CC | vesicle |
| 0.816 | GO:0044281 | H | BP | small molecule metabolic process |
| 0.814 | GO:0005576 | H | CC | extracellular region |
| 0.804 | GO:0009056 | H | BP | catabolic process |
| 0.774 | GO:0019222 | H | BP | regulation of metabolic process |
| 0.749 | GO:0034645 | H | BP | cellular macromolecule biosynthetic process |
| 0.74 | GO:0008092 | H | MF | cytoskeletal protein binding |
| 0.717 | GO:0005739 | H | CC | mitochondrion |
| 0.709 | GO:0055114 | H | BP | oxidation-reduction process |
| 0.701 | GO:0006810 | H | BP | transport |
| 0.696 | GO:0051171 | H | BP | regulation of nitrogen compound metabolic process |
| 0.675 | GO:0017076 | H | MF | purine nucleotide binding |
| 0.661 | GO:0000166 | H | MF | nucleotide binding |
| 0.652 | GO:0006796 | H | BP | phosphate-containing compound metabolic process |
| 0.644 | GO:0016020 | H | CC | membrane |
| 0.641 | GO:0006082 | H | BP | organic acid metabolic process |
| 0.63 | GO:0035639 | H | MF | purine ribonucleoside triphosphate binding |
| 0.622 | GO:0016462 | H | MF | pyrophosphatase activity |
| 0.608 | GO:0016491 | H | MF | oxidoreductase activity |
| 0.602 | GO:0051252 | H | BP | regulation of RNA metabolic process |
| 0.602 | GO:0032549 | H | MF | ribonucleoside binding |
| 0.596 | GO:0001882 | H | MF | nucleoside binding |
| 0.593 | GO:0009059 | H | BP | macromolecule biosynthetic process |
| 0.589 | GO:0031988 | H | CC | membrane-bounded vesicle |
| 0.582 | GO:0046914 | H | MF | transition metal ion binding |
| 0.575 | GO:0006355 | H | BP | regulation of transcription, DNA-templated |
| 0.568 | GO:0016817 | H | MF | hydrolase activity, acting on acid anhydrides |
| 0.567 | GO:0008017 | H | MF | microtubule binding |
| 0.552 | GO:0005975 | H | BP | carbohydrate metabolic process |
| 0.531 | GO:0001883 | H | MF | purine nucleoside binding |
| 0.51 | GO:0010468 | H | BP | regulation of gene expression |
| 0.967 | GO:0005737 | L | CC | cytoplasm |
| 0.945 | GO:0008152 | L | BP | metabolic process |
| 0.923 | GO:0044237 | L | BP | cellular metabolic process |
| 0.863 | GO:0097159 | L | MF | organic cyclic compound binding |
| 0.863 | GO:0050896 | L | BP | response to stimulus |
| 0.832 | GO:0051716 | L | BP | cellular response to stimulus |
| 0.83 | GO:0019538 | L | BP | protein metabolic process |
| 0.828 | GO:0007154 | L | BP | cell communication |
| 0.826 | GO:0036094 | L | MF | small molecule binding |
| 0.825 | GO:0043169 | L | MF | cation binding |
| 0.819 | GO:0005634 | L | CC | nucleus |
| 0.806 | GO:0043231 | L | CC | intracellular membrane-bounded organelle |
| 0.805 | GO:0009058 | L | BP | biosynthetic process |
| 0.793 | GO:0034641 | L | BP | cellular nitrogen compound metabolic process |
| 0.784 | GO:0046872 | L | MF | metal ion binding |
| 0.781 | GO:0005829 | L | CC | cytosol |
| 0.774 | GO:0046483 | L | BP | heterocycle metabolic process |
| 0.768 | GO:0043229 | L | CC | intracellular organelle |
| 0.757 | GO:0006725 | L | BP | cellular aromatic compound metabolic process |
| 0.754 | GO:0031981 | L | CC | nuclear lumen |
| 0.746 | GO:0044267 | L | BP | cellular protein metabolic process |
| 0.739 | GO:0050790 | L | BP | regulation of catalytic activity |
| 0.728 | GO:0006807 | L | BP | nitrogen compound metabolic process |
| 0.722 | GO:0032502 | L | BP | developmental process |
| 0.697 | GO:0032991 | L | CC | macromolecular complex |
| 0.684 | GO:0007165 | L | BP | signal transduction |
| 0.683 | GO:0005654 | L | CC | nucleoplasm |
| 0.68 | GO:0048856 | L | BP | anatomical structure development |
| 0.679 | GO:0006139 | L | BP | nucleobase-containing compound metabolic process |
| 0.669 | GO:0005102 | L | MF | receptor binding |
| 0.664 | GO:0006996 | L | BP | organelle organization |
| 0.653 | GO:0032403 | L | MF | protein complex binding |
| 0.639 | GO:0043234 | L | CC | protein complex |
| 0.624 | GO:0010467 | L | BP | gene expression |
| 0.619 | GO:0007275 | L | BP | multicellular organismal development |
| 0.614 | GO:0009893 | L | BP | positive regulation of metabolic process |
| 0.608 | GO:0006464 | L | BP | cellular protein modification process |
| 0.597 | GO:0019904 | L | MF | protein domain specific binding |
| 0.59 | GO:0016070 | L | BP | RNA metabolic process |
| 0.504 | GO:0010033 | L | BP | response to organic substance |

**Blast**

| **BLAST Matched UniProt ID & name** | | | **Identity** | **E Value** | **Score** |
| --- | --- | --- | --- | --- | --- |
| P28760 | SODM_BACCA | Superoxide dismutase [Mn] | 59.783 | 1.31E-73 | 226 |
| P00449 | SODM_GEOSE | Superoxide dismutase [Mn] | 59.783 | 3.40E-73 | 225 |
| Q9RUV2 | SODM_DEIRA | Superoxide dismutase [Mn] | 56.085 | 1.80E-69 | 216 |
| P54375 | SODM_BACSU | Superoxide dismutase [Mn] | 58.696 | 6.42E-69 | 214 |
| Q9KD10 | SODM_BACHD | Superoxide dismutase [Mn] | 57.609 | 1.09E-67 | 211 |
| Q59094 | SODM_ACIAD | Superoxide dismutase [Mn] | 54.945 | 1.25E-67 | 212 |
| Q81LW0 | SODM1_BACAN | Superoxide dismutase [Mn] 1 | 55.435 | 7.65E-67 | 209 |
| Q818I1 | SODM1_BACCR | Superoxide dismutase [Mn] 1 | 55.435 | 1.31E-66 | 209 |
| Q9F326 | SODM_STACA | Superoxide dismutase [Mn/Fe] | 54.891 | 3.70E-66 | 207 |
| P0C0F9 | SODM_XANC8 | Superoxide dismutase [Mn] | 52.198 | 5.07E-65 | 204 |
| Q838I4 | SODM_ENTFA | Superoxide dismutase [Fe] | 53.804 | 8.64E-65 | 204 |
| Q6G913 | SODM1_STAAS | Superoxide dismutase [Mn/Fe] 1 | 55.191 | 1.08E-64 | 203 |
| P53652 | SODM_PSEAE | Superoxide dismutase [Mn] | 53.846 | 1.23E-64 | 203 |
| Q8VQ15 | SODM_STAEP | Superoxide dismutase [Mn/Fe] | 54.645 | 1.33E-64 | 203 |
| P23744 | SODF_METJ | Superoxide dismutase [Mn/Fe] | 53.846 | 1.33E-64 | 203 |
| Q7SIC3 | SODM_VIRHA | Superoxide dismutase [Mn] | 54.645 | 2.79E-64 | 202 |
| Q5HNZ5 | SODM_STAEQ | Superoxide dismutase [Mn/Fe] | 54.098 | 4.56E-64 | 202 |
| P50058 | SODM1_LEPBY | Superoxide dismutase [Mn] 1 | 51.351 | 7.45E-64 | 203 |
| Q4L6Q3 | SODM_STAHJ | Superoxide dismutase [Mn/Fe] | 53.005 | 2.69E-63 | 200 |
| P28764 | SODM_LISMO | Superoxide dismutase [Mn] | 53.804 | 6.37E-63 | 199 |
| Q49XZ6 | SODM_STAS1 | Superoxide dismutase [Mn/Fe] | 53.005 | 4.86E-62 | 197 |
| P50059 | SODM2_LEPBY | Superoxide dismutase [Mn] 2 | 50.273 | 7.06E-62 | 196 |
| P53653 | SODM_THEAQ | Superoxide dismutase [Mn] | 54.645 | 1.92E-61 | 195 |
| P61502 | SODM_THET2 | Superoxide dismutase [Mn] | 53.552 | 3.56E-61 | 194 |
| P77929 | SODM_PSEPU | Superoxide dismutase [Mn] | 51.63 | 6.00E-61 | 194 |
| P66831 | SODM2_STAAN | Superoxide dismutase [Mn/Fe] 2 | 52.459 | 6.59E-61 | 194 |
| Q2YUU9 | SODM2_STAAB | Superoxide dismutase [Mn/Fe] 2 | 52.459 | 9.32E-61 | 193 |
| Q92BR6 | SODM_LISIN | Superoxide dismutase [Mn] | 50.543 | 1.20E-60 | 193 |
| Q9K4V3 | SODM_STAXY | Superoxide dismutase [Mn/Fe] | 51.913 | 1.88E-60 | 192 |
| P28763 | SODM_LISIV | Superoxide dismutase [Mn] | 52.973 | 3.43E-59 | 189 |
| Q42684 | SODM_CHLRE | Superoxide dismutase [Mn], mitochondrial | 50.785 | 9.70E-59 | 189 |
| P43725 | SODM_HAEIN | Superoxide dismutase [Mn] | 51.913 | 5.45E-58 | 187 |
| P53642 | SODM_BORPE | Superoxide dismutase [Mn] | 51.087 | 1.09E-57 | 186 |
| P77968 | SODF_SYNY3 | Superoxide dismutase [Fe] | 50.276 | 1.35E-57 | 185 |
| P50060 | SODM3_LEPBY | Superoxide dismutase [Mn] 3 | 51.22 | 4.80E-57 | 185 |
| P53655 | SODM_YEREN | Superoxide dismutase [Mn] | 50.538 | 5.05E-57 | 184 |
| P0A4J6 | SODM_STRPN | Superoxide dismutase [Mn] | 51.366 | 1.00E-56 | 183 |
| P0A4J1 | SODM_LACLA | Superoxide dismutase [Mn] | 50.802 | 1.27E-56 | 183 |
| Q814I6 | SODM2_BACCR | Superoxide dismutase [Mn] 2 | 48.634 | 1.88E-56 | 182 |
| Q81JK8 | SODM2_BACAN | Superoxide dismutase [Mn] 2 | 48.634 | 2.46E-56 | 182 |
| P53641 | SODF_PSEAE | Superoxide dismutase [Fe] | 49.718 | 5.90E-56 | 181 |
| P31108 | SODF_LEGPH | Superoxide dismutase [Fe] | 47.126 | 9.61E-55 | 177 |
| P0AGD5 | SODF_ECO57 | Superoxide dismutase [Fe] | 48.588 | 1.66E-54 | 177 |
| P0A2F4 | SODF_SALTY | Superoxide dismutase [Fe] | 48.023 | 3.18E-54 | 176 |
| Q59679 | SODM_MANHA | Superoxide dismutase [Mn] | 49.451 | 1.91E-53 | 175 |
| P0DF72 | SODM_STRP3 | Superoxide dismutase [Mn] | 48.634 | 2.36E-53 | 174 |
| Q8P0D4 | SODM_STRP8 | Superoxide dismutase [Mn] | 48.634 | 4.18E-53 | 174 |
| P50061 | SODF_LEPBY | Superoxide dismutase [Fe] | 50 | 5.00E-53 | 174 |
| O30826 | SODM_HAEDU | Superoxide dismutase [Mn] | 49.451 | 5.25E-53 | 174 |
| P09738 | SODM_STRMU | Superoxide dismutase [Mn/Fe] | 51.099 | 5.55E-53 | 174 |
| Q5XBF8 | SODM_STRP6 | Superoxide dismutase [Mn] | 48.634 | 7.24E-53 | 173 |
| P0C0I1 | SODM_STRP1 | Superoxide dismutase [Mn] | 48.087 | 7.73E-53 | 173 |
| Q9CPN6 | SODM_PASMU | Superoxide dismutase [Mn] | 47.802 | 7.96E-53 | 173 |
| Q8HXP2 | SODM_MACMU | Superoxide dismutase [Mn], mitochondrial | 50.276 | 8.14E-53 | 173 |
| P0C0I0 | SODM_STRPY | Superoxide dismutase [Mn] | 48.634 | 8.33E-53 | 173 |
| Q8HXP3 | SODM_MACFA | Superoxide dismutase [Mn], mitochondrial | 50.276 | 9.90E-53 | 173 |
| P43019 | SODM_SALTY | Superoxide dismutase [Mn] | 49.733 | 1.07E-52 | 173 |
| P66829 | SODM_SHIFL | Superoxide dismutase [Mn] | 47.849 | 1.29E-52 | 172 |
| Q8Z2V9 | SODM_SALTI | Superoxide dismutase [Mn] | 47.849 | 2.57E-52 | 172 |
| P00448 | SODM_ECOLI | Superoxide dismutase [Mn] | 47.312 | 2.60E-52 | 172 |
| Q8HXP0 | SODM_CALJA | Superoxide dismutase [Mn], mitochondrial | 51.351 | 2.85E-52 | 171 |
| Q8YSZ1 | SODF_NOSS1 | Superoxide dismutase [Fe] | 49.18 | 3.47E-52 | 171 |
| P41976 | SODM_BOVIN | Superoxide dismutase [Mn], mitochondrial | 49.727 | 3.53E-52 | 172 |
| P04179 | SODM_HUMAN | Superoxide dismutase [Mn], mitochondrial | 50.276 | 3.68E-52 | 172 |
| Q8HXP6 | SODM_PONPY | Superoxide dismutase [Mn], mitochondrial | 50.276 | 3.68E-52 | 172 |
| Q8HXP7 | SODM_PANTR | Superoxide dismutase [Mn], mitochondrial | 50.276 | 3.98E-52 | 171 |
| P19665 | SODF_PORGI | Superoxide dismutase [Mn/Fe] | 47.727 | 8.11E-52 | 170 |
| Q8HXP5 | SODM_HYLLA | Superoxide dismutase [Mn], mitochondrial | 49.724 | 1.13E-51 | 170 |
| P0A4J3 | SODM_STRA3 | Superoxide dismutase [Mn/Fe] | 49.18 | 1.61E-51 | 170 |
| Q9XS41 | SODM_HORSE | Superoxide dismutase [Mn], mitochondrial | 48.619 | 1.77E-51 | 170 |
| Q88PD5 | SODF_PSEPK | Superoxide dismutase [Fe] | 46.286 | 2.80E-51 | 169 |
| P09671 | SODM_MOUSE | Superoxide dismutase [Mn], mitochondrial | 49.724 | 4.38E-51 | 169 |
| Q5FB30 | SODM_MACNE | Superoxide dismutase [Mn], mitochondrial | 49.724 | 6.51E-51 | 169 |
| P22799 | SODM_RAOPL | Superoxide dismutase [Fe] | 53.642 | 1.11E-50 | 166 |
| P22302 | SODF_NICPL | Superoxide dismutase [Fe], chloroplastic | 48.087 | 1.23E-50 | 167 |
| P19685 | SODF_COXBU | Superoxide dismutase [Fe] | 43.503 | 1.39E-50 | 167 |
| P09223 | SODF_PSEPU | Superoxide dismutase [Fe] | 45.143 | 3.60E-50 | 166 |
| P41982 | SODM_RABIT | Superoxide dismutase [Mn], mitochondrial | 50 | 7.53E-50 | 166 |
| P07895 | SODM_RAT | Superoxide dismutase [Mn], mitochondrial | 48.066 | 1.43E-49 | 165 |
| P09213 | SODF_PHOLE | Superoxide dismutase [Fe] | 42.373 | 3.30E-48 | 161 |
| P37369 | SODF_BORPE | Superoxide dismutase [Fe] | 45.455 | 6.96E-48 | 160 |
| Q9UQX0 | SODM_SCHPO | Superoxide dismutase [Mn], mitochondrial | 46.111 | 2.93E-47 | 159 |
| P18655 | SODF_SYNE7 | Superoxide dismutase [Fe] | 45.349 | 3.13E-47 | 159 |
| Q8K9V4 | SODM_BUCAP | Superoxide dismutase [Mn] | 42.697 | 4.97E-47 | 158 |
| Q9XD74 | SODF_RHIME | Superoxide dismutase [Mn] | 47.253 | 1.61E-46 | 157 |
| P84612 | SODF_PSEHT | Superoxide dismutase [Fe] | 44.318 | 3.89E-46 | 155 |
| O30970 | SODF_RHOCA | Superoxide dismutase [Fe] | 44.382 | 5.45E-45 | 153 |
| O30563 | SODM_BORBU | Superoxide dismutase [Fe] | 42.778 | 6.80E-45 | 152 |
| Q89AR7 | SODM_BUCBP | Superoxide dismutase [Mn] | 41.436 | 9.26E-45 | 152 |
| Q00637 | SODM_DROME | Superoxide dismutase [Mn], mitochondrial | 45.304 | 2.75E-44 | 152 |
| P49114 | SODM_CAVPO | Superoxide dismutase [Mn], mitochondrial | 48.503 | 9.95E-44 | 150 |
| O81235 | SODM1_ARATH | Superoxide dismutase [Mn] 1, mitochondrial | 47.802 | 1.60E-43 | 150 |
| P57286 | SODM_BUCAI | Superoxide dismutase [Mn] | 40 | 2.39E-43 | 149 |
| Q9SM64 | SODM_PRUPE | Superoxide dismutase [Mn], mitochondrial | 46.927 | 2.33E-42 | 147 |
| P35017 | SODM_HEVBR | Superoxide dismutase [Mn], mitochondrial | 47.312 | 6.57E-42 | 146 |
| Q27740 | SODF_PLAFX | Superoxide dismutase [Fe] | 39.444 | 1.02E-41 | 144 |
| P41980 | SODM4_MAIZE | Superoxide dismutase [Mn] 3.4, mitochondrial | 47.093 | 2.83E-41 | 144 |
| P41978 | SODM2_MAIZE | Superoxide dismutase [Mn] 3.2, mitochondrial | 45.614 | 3.10E-41 | 144 |
| Q8IAY6 | SODF_PLAF7 | Superoxide dismutase [Fe] | 38.889 | 3.17E-41 | 143 |
| P53638 | SODF_BACFR | Superoxide dismutase [Fe] | 44.186 | 3.51E-41 | 143 |

**Protein name: Metallo-beta-lactamase superfamily protein**

**Predicted results:**

**SVMProt**

| **Protein Family Name** | **GO Category** | **SVM** | **PNN** | **KNN** |
| --- | --- | --- | --- | --- |
| **Molecular Function** | | | | |
| EC3.4 Hydrolases - Acting on peptide bonds (Peptidases) | - | 80.4 | - | Y |
| Zinc-binding | GO:0008270 zinc binding | 99 | - | - |
| EC1.1 Oxidoreductases - Acting on the CH-OH group of donors | - | 98.3 | - | - |
| EC3.1 Hydrolases - Acting on Ester Bonds | - | 76.2 | Y | - |
| EC2.3 Transferases - Acyltransferases | - | 95.2 | - | - |
| Iron-binding | GO:0005506 iron binding | 94.2 | - | - |
| EC4.1 Lyases - Carbon-Carbon Lyases | - | 94.2 | - | - |
| EC6.3 Ligases - Forming Carbon-Nitrogen Bonds | - | 93.6 | - | - |
| EC4.2 Lyases - Carbon-Oxygen Lyases | - | 91.3 | - | - |
| EC1.18 Oxidoreductases - Acting on iron-sulfur proteins as donors | - | 88.1 | ? | ? |
| EC3.2 Hydrolases - Glycosylases | - | 73.8 | - | - |
| Cobalt-binding | GO:0050897 cobalt binding | 68.5 | ? | ? |
| EC1.11 Oxidoreductases - Acting on a peroxide as acceptor | - | 65.4 | ? | ? |
| EC3.5 Hydrolases - Acting on Carbon-Nitrogen Bonds, other than Peptide Bonds | - | 62.2 | - | - |
| EC4.6 Lyases - Phosphorus-Oxygen Lyases | - | 58.6 | ? | ? |
| EC2.1 Transferases - Transferring One-Carbon Groups | - | 58.6 | - | - |
| Calcium-binding | - | 58.6 | - | - |
| Magnesium-binding | GO:0000287 magnesium binding | 58.6 | ? | ? |

**FFPred**

| **Score** | **GO term** | **RL** | **Domain** | **Description** |
| --- | --- | --- | --- | --- |
| 0.997 | GO:0003824 | H | MF | catalytic activity |
| 0.962 | GO:0044281 | H | BP | small molecule metabolic process |
| 0.942 | GO:0005576 | H | CC | extracellular region |
| 0.886 | GO:0006082 | H | BP | organic acid metabolic process |
| 0.877 | GO:0031988 | H | CC | membrane-bounded vesicle |
| 0.875 | GO:0050662 | H | MF | coenzyme binding |
| 0.864 | GO:0070062 | H | CC | extracellular vesicular exosome |
| 0.86 | GO:0019752 | H | BP | carboxylic acid metabolic process |
| 0.852 | GO:0055114 | H | BP | oxidation-reduction process |
| 0.846 | GO:0031982 | H | CC | vesicle |
| 0.82 | GO:0000166 | H | MF | nucleotide binding |
| 0.801 | GO:0016491 | H | MF | oxidoreductase activity |
| 0.777 | GO:0017076 | H | MF | purine nucleotide binding |
| 0.769 | GO:0006091 | H | BP | generation of precursor metabolites and energy |
| 0.702 | GO:0035639 | H | MF | purine ribonucleoside triphosphate binding |
| 0.7 | GO:0006796 | H | BP | phosphate-containing compound metabolic process |
| 0.694 | GO:1901605 | H | BP | alpha-amino acid metabolic process |
| 0.682 | GO:0005975 | H | BP | carbohydrate metabolic process |
| 0.679 | GO:0032549 | H | MF | ribonucleoside binding |
| 0.675 | GO:0016740 | H | MF | transferase activity |
| 0.655 | GO:0005739 | H | CC | mitochondrion |
| 0.65 | GO:0001883 | H | MF | purine nucleoside binding |
| 0.644 | GO:0048037 | H | MF | cofactor binding |
| 0.638 | GO:0032787 | H | BP | monocarboxylic acid metabolic process |
| 0.619 | GO:0009056 | H | BP | catabolic process |
| 0.594 | GO:0007264 | H | BP | small GTPase mediated signal transduction |
| 0.589 | GO:0044255 | H | BP | cellular lipid metabolic process |
| 0.586 | GO:0001882 | H | MF | nucleoside binding |
| 0.55 | GO:0006629 | H | BP | lipid metabolic process |
| 0.54 | GO:0006810 | H | BP | transport |
| 0.511 | GO:0055086 | H | BP | nucleobase-containing small molecule metabolic process |
| 0.509 | GO:0019637 | H | BP | organophosphate metabolic process |
| 0.508 | GO:0051641 | H | BP | cellular localization |
| 0.953 | GO:0044237 | L | BP | cellular metabolic process |
| 0.951 | GO:0005737 | L | CC | cytoplasm |
| 0.939 | GO:0008152 | L | BP | metabolic process |
| 0.904 | GO:0006807 | L | BP | nitrogen compound metabolic process |
| 0.88 | GO:0009058 | L | BP | biosynthetic process |
| 0.876 | GO:0036094 | L | MF | small molecule binding |
| 0.865 | GO:0006139 | L | BP | nucleobase-containing compound metabolic process |
| 0.862 | GO:0050896 | L | BP | response to stimulus |
| 0.857 | GO:0097159 | L | MF | organic cyclic compound binding |
| 0.826 | GO:0051716 | L | BP | cellular response to stimulus |
| 0.799 | GO:0043169 | L | MF | cation binding |
| 0.793 | GO:0005829 | L | CC | cytosol |
| 0.789 | GO:0046872 | L | MF | metal ion binding |
| 0.789 | GO:0005634 | L | CC | nucleus |
| 0.785 | GO:0034641 | L | BP | cellular nitrogen compound metabolic process |
| 0.757 | GO:0046483 | L | BP | heterocycle metabolic process |
| 0.75 | GO:0031981 | L | CC | nuclear lumen |
| 0.748 | GO:0006725 | L | BP | cellular aromatic compound metabolic process |
| 0.748 | GO:0032502 | L | BP | developmental process |
| 0.696 | GO:0005654 | L | CC | nucleoplasm |
| 0.69 | GO:0006464 | L | BP | cellular protein modification process |
| 0.683 | GO:0044267 | L | BP | cellular protein metabolic process |
| 0.679 | GO:0005794 | L | CC | Golgi apparatus |
| 0.668 | GO:0009405 | L | BP | pathogenesis |
| 0.658 | GO:0043229 | L | CC | intracellular organelle |
| 0.646 | GO:0007154 | L | BP | cell communication |
| 0.629 | GO:0006996 | L | BP | organelle organization |
| 0.617 | GO:0019538 | L | BP | protein metabolic process |
| 0.613 | GO:0010467 | L | BP | gene expression |
| 0.611 | GO:0007275 | L | BP | multicellular organismal development |
| 0.561 | GO:0048856 | L | BP | anatomical structure development |
| 0.544 | GO:0007165 | L | BP | signal transduction |
| 0.528 | GO:0043231 | L | CC | intracellular membrane-bounded organelle |
| 0.527 | GO:0023052 | L | BP | signaling |
| 0.522 | GO:0016070 | L | BP | RNA metabolic process |
| 0.509 | GO:0019318 | L | BP | hexose metabolic process |

**Blast**

| **BLAST Matched UniProt ID & name** | | | **Identity** | **E Value** | **Score** |
| --- | --- | --- | --- | --- | --- |
| P54435 | YRKH_BACSU | Uncharacterized protein YrkH | 29.752 | 5.77E-20 | 94.4 |
| P75849 | YCBL_ECOLI | Uncharacterized protein YcbL | 30.088 | 6.43E-20 | 91.7 |
| Q8N490 | PNKD_HUMAN | Probable hydrolase PNKD | 28.44 | 4.79E-15 | 80.1 |
| Q69ZP3 | PNKD_MOUSE | Probable hydrolase PNKD | 27.982 | 8.46E-15 | 79.3 |
| A7YY46 | PNKD_BOVIN | Probable hydrolase PNKD | 27.315 | 1.05E-14 | 79 |
| Q31ND6 | GLO2_SYNE7 | Hydroxyacylglutathione hydrolase | 30.233 | 8.78E-14 | 74.3 |
| Q9PFB0 | BLH_XYLFA | Beta-lactamase hydrolase-like protein | 26.95 | 3.37E-13 | 74.7 |
| Q3T094 | ETHE1_BOVIN | Persulfide dioxygenase ETHE1, mitochondrial | 27.363 | 3.90E-13 | 72.4 |
| Q87AD6 | BLH_XYLFT | Beta-lactamase hydrolase-like protein | 26.596 | 5.15E-13 | 73.9 |
| Q9DCM0 | ETHE1_MOUSE | Persulfide dioxygenase ETHE1, mitochondrial | 27.363 | 5.75E-13 | 72 |
| O95571 | ETHE1_HUMAN | Persulfide dioxygenase ETHE1, mitochondrial | 27.363 | 1.66E-12 | 70.5 |
| Q4URM1 | GLO2_XANC8 | Hydroxyacylglutathione hydrolase | 30.636 | 1.81E-12 | 70.5 |
| Q12BV7 | GLO2_POLSJ | Hydroxyacylglutathione hydrolase | 31.551 | 2.49E-12 | 70.1 |
| Q3JAC4 | GLO2_NITOC | Hydroxyacylglutathione hydrolase | 28.283 | 6.49E-12 | 68.9 |
| Q6NC62 | GLO2_RHOPA | Hydroxyacylglutathione hydrolase | 26.446 | 6.49E-12 | 68.9 |
| O34769 | PKSB_BACSU | Probable polyketide biosynthesis zinc-dependent hydrolase PksB | 26.776 | 8.26E-12 | 68.2 |
| Q8DIF1 | GLO2_THEEB | Hydroxyacylglutathione hydrolase | 29.319 | 3.82E-11 | 66.2 |
| A7Z4X7 | BAEB_BACA2 | Probable polyketide biosynthesis zinc-dependent hydrolase BaeB | 23.697 | 1.24E-10 | 64.3 |
| B7KEB4 | GLO2_CYAP7 | Hydroxyacylglutathione hydrolase | 28.652 | 1.82E-10 | 64.3 |
| Q9PBI4 | GLO2_XYLFA | Hydroxyacylglutathione hydrolase | 31.214 | 2.04E-10 | 64.3 |
| P72933 | GLO2_SYNY3 | Hydroxyacylglutathione hydrolase | 26.519 | 2.09E-10 | 64.3 |
| B0BZI8 | GLO2_ACAM1 | Hydroxyacylglutathione hydrolase | 24.719 | 2.93E-10 | 63.9 |
| B0U365 | GLO2_XYLFM | Hydroxyacylglutathione hydrolase | 30.636 | 3.48E-10 | 63.5 |
| Q87C74 | GLO2_XYLFT | Hydroxyacylglutathione hydrolase | 30.636 | 3.61E-10 | 63.5 |
| P54433 | YRKF_BACSU | UPF0033 protein YrkF | 36.486 | 1.02E-09 | 61.2 |
| Q21C03 | GLO2_RHOPB | Hydroxyacylglutathione hydrolase | 26.111 | 1.07E-09 | 62 |
| B1WUT9 | GLO2_CYAA5 | Hydroxyacylglutathione hydrolase | 28.07 | 1.07E-09 | 62 |
| Q1QQZ1 | GLO2_NITHX | Hydroxyacylglutathione hydrolase | 26.606 | 1.36E-09 | 62 |
| Q3JRV4 | Y2304_BURP1 | Probable metallo-hydrolase BURPS1710b_2304 | 23.864 | 1.51E-09 | 62.4 |
| B7K3R6 | GLO2_CYAP8 | Hydroxyacylglutathione hydrolase | 27.746 | 1.62E-09 | 61.6 |
| Q2J429 | GLO2_RHOP2 | Hydroxyacylglutathione hydrolase | 25 | 1.63E-09 | 61.6 |
| Q13F06 | GLO2_RHOPS | Hydroxyacylglutathione hydrolase | 25 | 1.67E-09 | 61.6 |
| Q9UT36 | GLO21_SCHPO | Probable hydroxyacylglutathione hydrolase C824.07 | 29.189 | 1.86E-09 | 61.6 |
| O94250 | GLO22_SCHPO | Probable hydroxyacylglutathione hydrolase C13B11.03c | 27.128 | 2.81E-09 | 60.8 |
| Q10Y41 | GLO2_TRIEI | Hydroxyacylglutathione hydrolase | 27.381 | 4.89E-09 | 60.1 |
| O67893 | Y2135_AQUAE | Uncharacterized protein aq_2135 | 26.374 | 5.42E-09 | 59.3 |
| A4YKS8 | GLO2_BRASO | Hydroxyacylglutathione hydrolase | 23.048 | 2.43E-08 | 58.2 |
| Q07VA9 | GLO2_RHOP5 | Hydroxyacylglutathione hydrolase | 23.889 | 4.50E-08 | 57.4 |
| Q3SVQ1 | GLO2_NITWN | Hydroxyacylglutathione hydrolase | 24.855 | 1.38E-07 | 55.8 |
| B2IVH7 | GLO2_NOSP7 | Hydroxyacylglutathione hydrolase | 25.882 | 2.22E-07 | 55.5 |
| Q65U07 | GLO2_MANSM | Hydroxyacylglutathione hydrolase | 25.833 | 2.24E-07 | 55.1 |
| B8HMJ9 | GLO2_CYAP4 | Hydroxyacylglutathione hydrolase | 27.66 | 2.59E-07 | 55.1 |
| Q57544 | Y1663_HAEIN | Uncharacterized protein HI_1663 | 25.389 | 2.74E-07 | 54.3 |
| A5ETG1 | GLO2_BRASB | Hydroxyacylglutathione hydrolase | 23.333 | 3.72E-07 | 54.7 |
| B8ICA2 | GLO2_METNO | Hydroxyacylglutathione hydrolase | 24.157 | 3.72E-07 | 54.7 |
| P54501 | YQGX_BACSU | Probable metallo-hydrolase YqgX | 25.946 | 4.25E-07 | 53.9 |
| Q89XT5 | GLO2_BRADU | Hydroxyacylglutathione hydrolase | 22.93 | 5.54E-07 | 53.9 |
| B0JW10 | GLO2_MICAN | Hydroxyacylglutathione hydrolase | 23.596 | 9.39E-07 | 53.5 |
| Q11DS3 | GLO2_CHESB | Hydroxyacylglutathione hydrolase | 26.59 | 3.20E-06 | 52 |
| Q08889 | GLO2_BUCAP | Hydroxyacylglutathione hydrolase | 28.488 | 4.22E-06 | 51.2 |
| Q8Z983 | GLO2_SALTI | Hydroxyacylglutathione hydrolase | 26.59 | 1.00E-05 | 50.4 |
| Q1LZ83 | LACB2_BOVIN | Beta-lactamase-like protein 2 | 27.626 | 1.05E-05 | 50.4 |
| Q99KR3 | LACB2_MOUSE | Beta-lactamase-like protein 2 | 24.873 | 1.94E-05 | 49.7 |
| A6VNK4 | GLO2_ACTSZ | Hydroxyacylglutathione hydrolase | 25.287 | 2.05E-05 | 49.3 |
| Q53H82 | LACB2_HUMAN | Beta-lactamase-like protein 2 | 25.888 | 2.53E-05 | 49.3 |
| A3PIB4 | GLO2_RHOS1 | Hydroxyacylglutathione hydrolase | 25.414 | 3.22E-05 | 48.9 |
| Q3J436 | GLO2_RHOS4 | Hydroxyacylglutathione hydrolase | 25.414 | 3.53E-05 | 48.5 |
| Q6NYF0 | LACB2_DANRE | Beta-lactamase-like protein 2 | 30.137 | 3.54E-05 | 48.9 |
| B3R1Y0 | GLO2_CUPTR | Hydroxyacylglutathione hydrolase | 26.809 | 5.12E-05 | 48.1 |
| P64262 | Y2612_MYCBO | Uncharacterized protein Mb2612c | 24.775 | 6.13E-05 | 47.8 |
| Q2NU52 | Y1048_SODGM | UPF0176 protein SG1048 | 29.412 | 6.35E-05 | 48.5 |
| Q7UFS5 | Y8368_RHOBA | UPF0176 protein RB8368 | 30.588 | 3.05E-04 | 46.2 |
| Q54EJ5 | GLOB2_DICDI | Glyoxylase B2 | 24.51 | 4.81E-04 | 45.4 |
| B2VDJ9 | Y2059_ERWT9 | UPF0176 protein ETA_20590 | 24.59 | 5.82E-04 | 45.4 |
| Q0V9A9 | LACB2_XENTR | Beta-lactamase-like protein 2 | 25.381 | 8.72E-04 | 44.7 |
| Q8ZRM2 | GLO2_SALTY | Hydroxyacylglutathione hydrolase | 25.989 | 0.001 | 43.9 |
| Q5PFD6 | GLO2_SALPA | Hydroxyacylglutathione hydrolase | 25.989 | 0.001 | 43.5 |
| Q1LT19 | Y454_BAUCH | UPF0176 protein BCI_0454 | 30.263 | 0.002 | 43.9 |
| B5FJ56 | GLO2_SALDC | Hydroxyacylglutathione hydrolase | 25.989 | 0.002 | 43.5 |
| Q57SZ8 | GLO2_SALCH | Hydroxyacylglutathione hydrolase | 25.989 | 0.002 | 43.1 |
| P51335 | MOEB_PORPU | Probable molybdopterin-synthase adenylyltransferase | 34.722 | 0.002 | 43.5 |
| C4K8L9 | Y081_HAMD5 | UPF0176 protein HDEF_0081 | 28.235 | 0.002 | 43.5 |
| A9MPF3 | GLO2_SALAR | Hydroxyacylglutathione hydrolase | 27.168 | 0.002 | 43.1 |
| B5R5L1 | GLO2_SALG2 | Hydroxyacylglutathione hydrolase | 25.989 | 0.003 | 42.7 |
| Q89AG4 | Y330_BUCBP | UPF0176 protein bbp_330 | 31.325 | 0.003 | 43.1 |
| A1JN32 | Y1619_YERE8 | UPF0176 protein YE1619 | 27.059 | 0.003 | 43.1 |
| B9DJ55 | Y2303_STACT | UPF0176 protein Sca_2303 | 38.158 | 0.003 | 43.1 |
| Q8ZDV4 | Y2451_YERPE | UPF0176 protein YPO2451/y1738/YP_2271 | 27.059 | 0.003 | 43.1 |
| C0Q6N0 | GLO2_SALPC | Hydroxyacylglutathione hydrolase | 25.989 | 0.005 | 42 |
| P57336 | GLO2_BUCAI | Hydroxyacylglutathione hydrolase | 27.957 | 6.00E-03 | 42 |
| B0C1U8 | Y1075_ACAM1 | UPF0176 protein AM1_1075 | 30.588 | 6.00E-03 | 42 |
| A8F9I7 | Y205_BACP2 | UPF0176 protein BPUM_0205 | 39.726 | 6.00E-03 | 42 |
| Q5NFX8 | Y1085_FRATT | UPF0176 protein FTT_1085 | 25.397 | 7.00E-03 | 42 |
| Q5XGR8 | LACB2_XENLA | Beta-lactamase-like protein 2 | 25.949 | 7.00E-03 | 41.6 |
| Q2A397 | Y1118_FRATH | UPF0176 protein FTL_1118 | 25.397 | 7.00E-03 | 41.6 |
| Q7N5W3 | Y1816_PHOLL | UPF0176 protein plu1816 | 25.882 | 8.00E-03 | 42 |
| Q2GJC1 | Y962_ANAPZ | UPF0176 protein APH_0962 | 28.409 | 8.00E-03 | 41.6 |
| Q0HVM5 | Y1838_SHESR | UPF0176 protein Shewmr7_1838 | 26.115 | 9.00E-03 | 41.6 |
| Q65EW5 | Y3574_BACLD | UPF0176 protein BLi03574/BL00834 | 36.842 | 9.00E-03 | 41.6 |
| Q5PBC6 | Y315_ANAMM | UPF0176 protein AM315 | 29.545 | 9.00E-03 | 41.2 |
| A0Q614 | Y789_FRATN | UPF0176 protein FTN_0789 | 28.723 | 9.00E-03 | 41.6 |
| A0KXH1 | Y2261_SHESA | UPF0176 protein Shewana3_2261 | 26.115 | 1.00E-02 | 41.2 |
| Q0HJD3 | Y1760_SHESM | UPF0176 protein Shewmr4_1760 | 26.115 | 1.00E-02 | 41.2 |
| Q837T2 | Y748_ENTFA | UPF0176 protein EF_0748 | 35 | 1.00E-02 | 41.2 |
| A8GCZ9 | Y1886_SERP5 | UPF0176 protein Spro_1886 | 25.882 | 1.20E-02 | 41.2 |
| Q4A0W6 | Y127_STAS1 | UPF0176 protein SSP0127 | 36.842 | 1.20E-02 | 41.2 |
| Q5WEM1 | Y2654_BACSK | UPF0176 protein ABC2654 | 32.53 | 1.40E-02 | 40.8 |
| B9KHZ8 | Y234_ANAMF | UPF0176 protein AMF_234 | 29.545 | 1.50E-02 | 40.8 |
| Q9PJB6 | Y916_CHLMU | UPF0176 protein TC_0916 | 30.864 | 1.50E-02 | 40.8 |
| Q47UT5 | Y4798_COLP3 | UPF0176 protein CPS_4798 | 28.235 | 2.20E-02 | 40.4 |

**Protein name: flavin prenyltransferase**

**Predicted results:**

**SVMProt**

| **Protein Family Name** | **GO Category** | **SVM** | **PNN** | **KNN** |
| --- | --- | --- | --- | --- |
| **Molecular Function** | | | | |
| EC2.4 Transferases - Glycosyltransferases | - | 78.4 | - | Y |
| EC2.1 Transferases - Transferring One-Carbon Groups | - | 58.6 | - | Y |
| Iron-binding | GO:0005506 iron binding | 71.3 | - | - |
| All lipid-binding proteins | GO:0008289 lipid binding | 65.4 | - | - |
| Magnesium-binding | GO:0000287 magnesium binding | 58.6 | ? | ? |
| Calcium-binding | - | 58.6 | - | - |
| **Biological Process** | | | | |
| Flavoprotein | - | 98.7 | Y | Y |
| DNA repair | GO:0006281 DNA repair | 58.6 | ? | ? |
| **Broadly Defined Function** | | | | |
| Transmembrane | GO:0016021 integral component of membrane | 65.4 | - | - |
| Outer membrane | GO:0009279 cell outer membrane | 58.6 | - | - |

**FFPred**

| **Score** | **GO term** | **RL** | **Domain** | **Description** |
| --- | --- | --- | --- | --- |
| 0.987 | GO:0003824 | H | MF | catalytic activity |
| 0.955 | GO:0006082 | H | BP | organic acid metabolic process |
| 0.934 | GO:0044281 | H | BP | small molecule metabolic process |
| 0.928 | GO:0005576 | H | CC | extracellular region |
| 0.891 | GO:0009165 | H | BP | nucleotide biosynthetic process |
| 0.889 | GO:0019752 | H | BP | carboxylic acid metabolic process |
| 0.875 | GO:0006796 | H | BP | phosphate-containing compound metabolic process |
| 0.856 | GO:0019637 | H | BP | organophosphate metabolic process |
| 0.854 | GO:0046395 | H | BP | carboxylic acid catabolic process |
| 0.833 | GO:0005975 | H | BP | carbohydrate metabolic process |
| 0.832 | GO:0009056 | H | BP | catabolic process |
| 0.824 | GO:0031988 | H | CC | membrane-bounded vesicle |
| 0.82 | GO:0031982 | H | CC | vesicle |
| 0.811 | GO:0035639 | H | MF | purine ribonucleoside triphosphate binding |
| 0.806 | GO:0000166 | H | MF | nucleotide binding |
| 0.794 | GO:0019222 | H | BP | regulation of metabolic process |
| 0.783 | GO:0032549 | H | MF | ribonucleoside binding |
| 0.779 | GO:0017076 | H | MF | purine nucleotide binding |
| 0.778 | GO:0001883 | H | MF | purine nucleoside binding |
| 0.755 | GO:0070062 | H | CC | extracellular vesicular exosome |
| 0.752 | GO:0005739 | H | CC | mitochondrion |
| 0.741 | GO:0005524 | H | MF | ATP binding |
| 0.73 | GO:0001882 | H | MF | nucleoside binding |
| 0.7 | GO:0005525 | H | MF | GTP binding |
| 0.678 | GO:0055086 | H | BP | nucleobase-containing small molecule metabolic process |
| 0.674 | GO:0010468 | H | BP | regulation of gene expression |
| 0.671 | GO:0006520 | H | BP | cellular amino acid metabolic process |
| 0.67 | GO:0009117 | H | BP | nucleotide metabolic process |
| 0.665 | GO:1901605 | H | BP | alpha-amino acid metabolic process |
| 0.664 | GO:0006355 | H | BP | regulation of transcription, DNA-templated |
| 0.66 | GO:0051171 | H | BP | regulation of nitrogen compound metabolic process |
| 0.648 | GO:0032561 | H | MF | guanyl ribonucleotide binding |
| 0.647 | GO:0016311 | H | BP | dephosphorylation |
| 0.633 | GO:1903506 | H | BP | regulation of nucleic acid-templated transcription |
| 0.613 | GO:0006163 | H | BP | purine nucleotide metabolic process |
| 0.594 | GO:0006629 | H | BP | lipid metabolic process |
| 0.591 | GO:0032787 | H | BP | monocarboxylic acid metabolic process |
| 0.581 | GO:0035556 | H | BP | intracellular signal transduction |
| 0.577 | GO:0055114 | H | BP | oxidation-reduction process |
| 0.576 | GO:0016788 | H | MF | hydrolase activity, acting on ester bonds |
| 0.576 | GO:0016301 | H | MF | kinase activity |
| 0.57 | GO:2001141 | H | BP | regulation of RNA biosynthetic process |
| 0.566 | GO:0006810 | H | BP | transport |
| 0.564 | GO:0051641 | H | BP | cellular localization |
| 0.557 | GO:0016740 | H | MF | transferase activity |
| 0.533 | GO:0009059 | H | BP | macromolecule biosynthetic process |
| 0.527 | GO:0051252 | H | BP | regulation of RNA metabolic process |
| 0.522 | GO:0030554 | H | MF | adenyl nucleotide binding |
| 0.501 | GO:0046907 | H | BP | intracellular transport |
| 0.5 | GO:0008233 | H | MF | peptidase activity |
| 0.978 | GO:0008152 | L | BP | metabolic process |
| 0.943 | GO:0044237 | L | BP | cellular metabolic process |
| 0.941 | GO:0005737 | L | CC | cytoplasm |
| 0.936 | GO:0009058 | L | BP | biosynthetic process |
| 0.918 | GO:0019538 | L | BP | protein metabolic process |
| 0.903 | GO:0006139 | L | BP | nucleobase-containing compound metabolic process |
| 0.887 | GO:0043229 | L | CC | intracellular organelle |
| 0.884 | GO:0006807 | L | BP | nitrogen compound metabolic process |
| 0.866 | GO:0050896 | L | BP | response to stimulus |
| 0.85 | GO:0016787 | L | MF | hydrolase activity |
| 0.838 | GO:0043231 | L | CC | intracellular membrane-bounded organelle |
| 0.829 | GO:0051716 | L | BP | cellular response to stimulus |
| 0.807 | GO:0005634 | L | CC | nucleus |
| 0.793 | GO:0034641 | L | BP | cellular nitrogen compound metabolic process |
| 0.791 | GO:0036094 | L | MF | small molecule binding |
| 0.781 | GO:0046483 | L | BP | heterocycle metabolic process |
| 0.759 | GO:0006725 | L | BP | cellular aromatic compound metabolic process |
| 0.756 | GO:0043169 | L | MF | cation binding |
| 0.756 | GO:0032502 | L | BP | developmental process |
| 0.747 | GO:0007154 | L | BP | cell communication |
| 0.738 | GO:0005829 | L | CC | cytosol |
| 0.726 | GO:0044267 | L | BP | cellular protein metabolic process |
| 0.721 | GO:0046872 | L | MF | metal ion binding |
| 0.72 | GO:0031981 | L | CC | nuclear lumen |
| 0.713 | GO:0097159 | L | MF | organic cyclic compound binding |
| 0.686 | GO:0007165 | L | BP | signal transduction |
| 0.68 | GO:0006996 | L | BP | organelle organization |
| 0.657 | GO:0005654 | L | CC | nucleoplasm |
| 0.631 | GO:0032991 | L | CC | macromolecular complex |
| 0.623 | GO:0010467 | L | BP | gene expression |
| 0.623 | GO:0023052 | L | BP | signaling |
| 0.616 | GO:0006464 | L | BP | cellular protein modification process |
| 0.611 | GO:0009893 | L | BP | positive regulation of metabolic process |
| 0.601 | GO:0043234 | L | CC | protein complex |
| 0.591 | GO:0007275 | L | BP | multicellular organismal development |
| 0.59 | GO:0005102 | L | MF | receptor binding |
| 0.585 | GO:0016070 | L | BP | RNA metabolic process |
| 0.569 | GO:0009966 | L | BP | regulation of signal transduction |
| 0.549 | GO:0048856 | L | BP | anatomical structure development |
| 0.501 | GO:0010033 | L | BP | response to organic substance |

**Blast**

| **BLAST Matched UniProt ID & name** | | | **Identity** | **E Value** | **Score** |
| --- | --- | --- | --- | --- | --- |
| Q9KP38 | PAAD_VIBCH | Probable aromatic acid decarboxylase | 59.809 | 6.27E-86 | 259 |
| Q9KCC2 | PAAD_BACHD | Probable aromatic acid decarboxylase | 47.449 | 1.21E-60 | 194 |
| P94300 | PAAD_BACPE | Probable aromatic acid decarboxylase | 47.449 | 1.39E-60 | 194 |
| Q57566 | PAAD_METJA | Probable aromatic acid decarboxylase | 44.388 | 9.40E-57 | 184 |
| Q9V030 | PAAD_PYRAB | Probable aromatic acid decarboxylase | 43.719 | 6.80E-53 | 174 |
| O66811 | PAAD_AQUAE | Probable aromatic acid decarboxylase | 40.909 | 2.40E-52 | 172 |
| O26250 | PAAD_METTH | Probable aromatic acid decarboxylase | 46.392 | 1.47E-51 | 171 |
| O58742 | PAAD_PYRHO | Probable aromatic acid decarboxylase | 41.327 | 9.12E-48 | 160 |
| Q9HJ72 | PAAD_THEAC | Probable aromatic acid decarboxylase | 42.051 | 2.32E-46 | 157 |
| P72743 | PAAD_SYNY3 | Probable aromatic acid decarboxylase | 42.564 | 1.25E-44 | 153 |
| P33751 | PAD1_YEAST | Phenylacrylic acid decarboxylase 1, mitochondrial | 42.132 | 1.47E-44 | 154 |
| P94404 | BSDB_BACSU | Phenolic acid decarboxylase subunit B | 39.394 | 4.79E-43 | 149 |
| O29054 | PAAD_ARCFU | Probable aromatic acid decarboxylase | 40.306 | 2.49E-41 | 144 |
| Q9ZD09 | PAAD_RICPR | Probable aromatic acid decarboxylase | 38.624 | 4.99E-41 | 143 |
| Q9RR91 | PAAD_DEIRA | Probable aromatic acid decarboxylase | 42.079 | 1.21E-40 | 142 |
| Q9Z8S4 | BSDB_CHLPN | 4-hydroxybenzoate decarboxylase subunit B | 39.086 | 4.67E-40 | 141 |
| P0AG04 | UBIX_ECO57 | 3-octaprenyl-4-hydroxybenzoate carboxy-lyase partner protein | 40.201 | 4.05E-39 | 138 |
| P57767 | PAAD_THAAR | Probable aromatic acid decarboxylase | 40.513 | 8.67E-39 | 137 |
| P69772 | PAD1_ECO57 | Probable aromatic acid decarboxylase | 38.889 | 1.98E-38 | 137 |
| P69774 | PAD1_ECOLX | Probable aromatic acid decarboxylase | 38.889 | 4.48E-38 | 136 |
| P0A245 | UBIX_SALTY | 3-octaprenyl-4-hydroxybenzoate carboxy-lyase partner protein | 39.698 | 3.81E-37 | 133 |
| O84222 | PAAD_CHLTR | Probable aromatic acid decarboxylase | 38.265 | 8.50E-37 | 132 |
| P69773 | PAD1_ECO11 | Probable aromatic acid decarboxylase | 38.384 | 1.34E-36 | 132 |
| Q9Y8K8 | PAAD_SULSO | Probable aromatic acid decarboxylase | 36.224 | 1.38E-36 | 132 |
| Q9PKH2 | PAAD_CHLMU | Probable aromatic acid decarboxylase | 37 | 6.25E-36 | 130 |
| Q9JXP4 | PAAD_NEIMB | Probable aromatic acid decarboxylase | 36.735 | 9.23E-36 | 130 |
| Q9YBF0 | PAAD_AERPE | Probable aromatic acid decarboxylase | 35.025 | 4.30E-35 | 128 |
| Q9JW78 | PAAD_NEIMA | Probable aromatic acid decarboxylase | 36.224 | 4.34E-35 | 128 |
| O26011 | PAAD_HELPY | Probable aromatic acid decarboxylase | 37.245 | 6.11E-35 | 127 |
| Q9ZJE3 | PAAD_HELPJ | Probable aromatic acid decarboxylase | 36.224 | 1.76E-34 | 126 |
| Q9KYP1 | PAAD_STRCO | Probable aromatic acid decarboxylase | 32.512 | 4.54E-29 | 113 |
| Q9PPF1 | PAAD_CAMJE | Probable aromatic acid decarboxylase | 32.143 | 3.53E-24 | 99.4 |
| Q54Y51 | COAC_DICDI | Putative phosphopantothenoylcysteine decarboxylase | 20.874 | 2.00E-03 | 41.2 |

**Protein name: alpha-galactosidase**

**Predicted results:**

**SVMProt**

| **Protein Family Name** | **GO Category** | **SVM** | **PNN** | **KNN** |
| --- | --- | --- | --- | --- |
| **Molecular Function** | | | | |
| EC3.2 Hydrolases - Glycosylases | - | 99.1 | - | Y |
| EC6.1 Ligases - Forming Carbon-Oxygen Bonds | - | 96.4 | - | - |
| Zinc-binding | GO:0008270 zinc binding | 89.3 | - | - |
| Iron-binding | GO:0005506 iron binding | 80.4 | - | - |
| Metal-binding | GO:0046872 metal ion binding | 62.2 | - | - |
| EC4.6 Lyases - Phosphorus-Oxygen Lyases | - | 58.6 | ? | ? |
| Lectin | GO:0030246 carbohydrate binding | 58.6 | ? | ? |
| **Broadly Defined Function** | | | | |
| Photosystem I | GO:0009522 photosystem I | 58.6 | ? | ? |

**FFPred**

| **Score** | **GO term** | **RL** | **Domain** | **Description** |
| --- | --- | --- | --- | --- |
| 0.934 | GO:0005576 | H | CC | extracellular region |
| 0.899 | GO:0005615 | H | CC | extracellular space |
| 0.895 | GO:0003824 | H | MF | catalytic activity |
| 0.891 | GO:0070062 | H | CC | extracellular vesicular exosome |
| 0.831 | GO:0005578 | H | CC | proteinaceous extracellular matrix |
| 0.817 | GO:0031988 | H | CC | membrane-bounded vesicle |
| 0.804 | GO:0031982 | H | CC | vesicle |
| 0.803 | GO:0005975 | H | BP | carbohydrate metabolic process |
| 0.796 | GO:0043062 | H | BP | extracellular structure organization |
| 0.737 | GO:0030203 | H | BP | glycosaminoglycan metabolic process |
| 0.712 | GO:0044281 | H | BP | small molecule metabolic process |
| 0.7 | GO:0005788 | H | CC | endoplasmic reticulum lumen |
| 0.684 | GO:0012505 | H | CC | endomembrane system |
| 0.655 | GO:0030198 | H | BP | extracellular matrix organization |
| 0.616 | GO:0098588 | H | CC | bounding membrane of organelle |
| 0.614 | GO:0030246 | H | MF | carbohydrate binding |
| 0.598 | GO:0016758 | H | MF | transferase activity, transferring hexosyl groups |
| 0.597 | GO:0006066 | H | BP | alcohol metabolic process |
| 0.588 | GO:0006082 | H | BP | organic acid metabolic process |
| 0.565 | GO:0006810 | H | BP | transport |
| 0.554 | GO:0016020 | H | CC | membrane |
| 0.525 | GO:0050839 | H | MF | cell adhesion molecule binding |
| 0.518 | GO:0005783 | H | CC | endoplasmic reticulum |
| 0.507 | GO:0005886 | H | CC | plasma membrane |
| 0.506 | GO:0071944 | H | CC | cell periphery |
| 0.503 | GO:0008233 | H | MF | peptidase activity |
| 0.502 | GO:0055114 | H | BP | oxidation-reduction process |
| 0.903 | GO:0050896 | L | BP | response to stimulus |
| 0.902 | GO:0008152 | L | BP | metabolic process |
| 0.826 | GO:0051716 | L | BP | cellular response to stimulus |
| 0.804 | GO:0048856 | L | BP | anatomical structure development |
| 0.794 | GO:0019538 | L | BP | protein metabolic process |
| 0.782 | GO:0005737 | L | CC | cytoplasm |
| 0.758 | GO:0007275 | L | BP | multicellular organismal development |
| 0.755 | GO:0044237 | L | BP | cellular metabolic process |
| 0.738 | GO:0032502 | L | BP | developmental process |
| 0.726 | GO:0043231 | L | CC | intracellular membrane-bounded organelle |
| 0.716 | GO:0005102 | L | MF | receptor binding |
| 0.714 | GO:0006807 | L | BP | nitrogen compound metabolic process |
| 0.685 | GO:0016787 | L | MF | hydrolase activity |
| 0.677 | GO:0043229 | L | CC | intracellular organelle |
| 0.674 | GO:0046872 | L | MF | metal ion binding |
| 0.664 | GO:0030154 | L | BP | cell differentiation |
| 0.643 | GO:0044267 | L | BP | cellular protein metabolic process |
| 0.623 | GO:0048513 | L | BP | organ development |
| 0.617 | GO:0007154 | L | BP | cell communication |
| 0.606 | GO:0009605 | L | BP | response to external stimulus |
| 0.599 | GO:0043169 | L | MF | cation binding |
| 0.539 | GO:0010033 | L | BP | response to organic substance |
| 0.534 | GO:0050790 | L | BP | regulation of catalytic activity |
| 0.531 | GO:0005764 | L | CC | lysosome |
| 0.506 | GO:0032991 | L | CC | macromolecular complex |

**Blast**

| **BLAST Matched UniProt ID & name** | | | **Identity** | **E Value** | **Score** |
| --- | --- | --- | --- | --- | --- |
| P31434 | XYLS_ECOLI | Alpha-xylosidase | 24.528 | 6.05E-43 | 169 |
| Q9F234 | AGL2_BACTQ | Alpha-glucosidase 2 | 27.188 | 1.24E-41 | 166 |
| Q9P999 | XYLS_SULSO | Alpha-xylosidase | 24.655 | 2.02E-37 | 152 |
| Q5AW25 | AGDD_EMENI | Alpha-xylosidase | 24.92 | 2.97E-36 | 149 |
| Q4R4N7 | GANAB_MACFA | Neutral alpha-glucosidase AB | 26.087 | 1.15E-28 | 126 |
| Q14697 | GANAB_HUMAN | Neutral alpha-glucosidase AB | 25.543 | 4.40E-27 | 121 |
| A7LXT0 | GH31A_BACO1 | Alpha-xylosidase BoGH31A | 23.408 | 5.98E-27 | 120 |
| Q8BHN3 | GANAB_MOUSE | Neutral alpha-glucosidase AB | 25.316 | 2.59E-26 | 119 |
| P79403 | GANAB_PIG | Neutral alpha-glucosidase AB | 25.362 | 3.19E-26 | 118 |
| D0KQM8 | AGLU_SULS9 | Alpha-glucosidase | 21.842 | 1.34E-25 | 116 |
| P0CD66 | AGLU_SULSO | Alpha-glucosidase | 22.088 | 4.72E-25 | 114 |
| B3PEE6 | OL4AG_CELJU | Oligosaccharide 4-alpha-D-glucosyltransferase | 23.744 | 2.17E-24 | 112 |
| Q94502 | GANAB_DICDI | Neutral alpha-glucosidase AB | 23.363 | 1.19E-23 | 110 |
| Q9BE70 | GANC_MACFA | Neutral alpha-glucosidase C | 23.819 | 2.91E-23 | 108 |
| Q8TET4 | GANC_HUMAN | Neutral alpha-glucosidase C | 24.085 | 3.52E-23 | 108 |
| Q8BVW0 | GANC_MOUSE | Neutral alpha-glucosidase C | 23.674 | 1.80E-21 | 103 |
| Q9US55 | GLU2A_SCHPO | Glucosidase 2 subunit alpha | 22.68 | 4.41E-21 | 102 |
| B9F676 | GLU2A_ORYSJ | Probable glucan 1,3-alpha-glucosidase | 23.282 | 1.52E-17 | 90.9 |
| Q9MYM4 | LYAG_BOVIN | Lysosomal alpha-glucosidase | 24.242 | 1.68E-17 | 90.5 |
| P70699 | LYAG_MOUSE | Lysosomal alpha-glucosidase | 22.852 | 1.65E-13 | 77.8 |
| P10253 | LYAG_HUMAN | Lysosomal alpha-glucosidase | 22.575 | 1.70E-12 | 74.7 |
| P38138 | GLU2A_YEAST | Glucosidase 2 subunit alpha | 22.004 | 8.54E-12 | 72.4 |
| Q6P7A9 | LYAG_RAT | Lysosomal alpha-glucosidase | 22.783 | 7.65E-11 | 69.3 |
| O04893 | AGLU_SPIOL | Alpha-glucosidase | 23.2 | 1.89E-10 | 67.8 |
| Q69ZQ1 | K1161_MOUSE | Uncharacterized family 31 glucosidase KIAA1161 | 24.672 | 1.16E-09 | 65.1 |
| Q653V7 | AGLU_ORYSJ | Probable alpha-glucosidase Os06g0675700 | 21.636 | 1.57E-08 | 61.6 |
| O74254 | AMYG_CANAL | Glucoamylase 1 | 24.299 | 8.94E-08 | 59.3 |
| Q92442 | AGLU_MUCJA | Alpha-glucosidase | 26.009 | 1.02E-07 | 58.9 |
| Q6NSJ0 | K1161_HUMAN | Uncharacterized family 31 glucosidase KIAA1161 | 25.532 | 1.33E-07 | 58.5 |
| F4J6T7 | XYL2_ARATH | Putative alpha-xylosidase 2 | 21.926 | 2.06E-07 | 58.2 |
| Q9C0Y4 | AGLU_SCHPO | Alpha-glucosidase | 25.333 | 2.31E-07 | 57.8 |
| Q43763 | AGLU_HORVU | Alpha-glucosidase | 20.109 | 2.10E-06 | 54.7 |
| O43451 | MGA_HUMAN | Maltase-glucoamylase, intestinal | 27.149 | 2.10E-06 | 55.1 |
| O43451 | MGA_HUMAN | Maltase-glucoamylase, intestinal | 25.781 | 0.037 | 41.2 |
| P56526 | AGLU_ASPNG | Alpha-glucosidase | 27.368 | 2.21E-06 | 54.7 |
| O04931 | AGLU_BETVU | Alpha-glucosidase | 20.348 | 2.35E-06 | 54.7 |
| P14410 | SUIS_HUMAN | Sucrase-isomaltase, intestinal | 25.789 | 8.44E-06 | 53.1 |
| P23739 | SUIS_RAT | Sucrase-isomaltase, intestinal | 24.402 | 1.07E-05 | 52.8 |
| P23739 | SUIS_RAT | Sucrase-isomaltase, intestinal | 25 | 0.012 | 42.7 |
| P07768 | SUIS_RABIT | Sucrase-isomaltase, intestinal | 25.521 | 1.88E-05 | 52 |
| Q5R7A9 | LYAG_PONAB | Lysosomal alpha-glucosidase | 26.667 | 3.98E-05 | 50.8 |
| Q0CMA7 | AGDC_ASPTN | Probable alpha/beta-glucosidase agdC | 27.273 | 7.58E-05 | 49.7 |
| Q12558 | AGLU_ASPOR | Alpha-glucosidase | 23.75 | 7.67E-05 | 49.7 |
| O62653 | SUIS_SUNMU | Sucrase-isomaltase, intestinal | 26.425 | 8.08E-05 | 49.7 |
| O62653 | SUIS_SUNMU | Sucrase-isomaltase, intestinal | 25.862 | 3.20E-02 | 41.2 |
| A1D1E6 | AGDC_NEOFI | Probable alpha/beta-glucosidase agdC | 24.779 | 1.89E-04 | 48.5 |
| Q2M2H8 | MGAL_HUMAN | Probable maltase-glucoamylase 2 | 24.309 | 1.00E-03 | 46.2 |
| Q4WRH9 | AGDC_ASPFU | Probable alpha/beta-glucosidase agdC | 24.034 | 7.00E-03 | 43.5 |
| Q5AWI5 | AGDC_EMENI | Alpha/beta-glucosidase agdC | 21.591 | 1.20E-02 | 42.7 |
| P22861 | AMYG_SCHOC | Glucoamylase 1 | 28.571 | 4.30E-02 | 40.8 |

**Protein name: Esterase**

**Predicted results:**

**SVMProt**

| **Protein Family Name** | **GO Category** | **SVM** | **PNN** | **KNN** |
| --- | --- | --- | --- | --- |
| **Molecular Function** | | | | |
| EC2.7 Transferases - Transferring Phosphorus-Containing Groups | - | 98.6 | - | - |
| Manganese-binding | GO:0030145 manganese binding | 97.5 | ? | ? |
| Zinc-binding | GO:0008270 zinc binding | 88.1 | - | - |
| All DNA-binding | GO:0003677 DNA binding | 76.2 | ? | ? |
| EC4.1 Lyases - Carbon-Carbon Lyases | - | 71.3 | - | - |
| EC2.4 Transferases - Glycosyltransferases | - | 65.4 | - | - |
| EC1.2 Oxidoreductases - Acting on the aldehyde or oxo group of donors | - | 62.2 | - | - |
| Magnesium-binding | GO:0000287 magnesium binding | 58.6 | ? | ? |
| **Biological Process** | | | | |
| DNA recombination | GO:0006310 DNA recombination | 68.5 | ? | ? |
| **Broadly Defined Function** | | | | |
| Outer membrane | GO:0009279 cell outer membrane | 58.6 | - | - |

**FFPred**

| **Score** | **GO term** | **RL** | **Domain** | **Description** |
| --- | --- | --- | --- | --- |
| 0.988 | GO:0003824 | H | MF | catalytic activity |
| 0.913 | GO:0005739 | H | CC | mitochondrion |
| 0.904 | GO:0044281 | H | BP | small molecule metabolic process |
| 0.846 | GO:0030554 | H | MF | adenyl nucleotide binding |
| 0.775 | GO:0035639 | H | MF | purine ribonucleoside triphosphate binding |
| 0.753 | GO:0006796 | H | BP | phosphate-containing compound metabolic process |
| 0.753 | GO:0017076 | H | MF | purine nucleotide binding |
| 0.746 | GO:0000166 | H | MF | nucleotide binding |
| 0.714 | GO:0009056 | H | BP | catabolic process |
| 0.7 | GO:0055114 | H | BP | oxidation-reduction process |
| 0.699 | GO:0001883 | H | MF | purine nucleoside binding |
| 0.696 | GO:0032549 | H | MF | ribonucleoside binding |
| 0.684 | GO:0001882 | H | MF | nucleoside binding |
| 0.679 | GO:0019752 | H | BP | carboxylic acid metabolic process |
| 0.658 | GO:0019222 | H | BP | regulation of metabolic process |
| 0.642 | GO:0005524 | H | MF | ATP binding |
| 0.632 | GO:0009116 | H | BP | nucleoside metabolic process |
| 0.628 | GO:0048037 | H | MF | cofactor binding |
| 0.626 | GO:0055086 | H | BP | nucleobase-containing small molecule metabolic process |
| 0.591 | GO:0016874 | H | MF | ligase activity |
| 0.576 | GO:0006629 | H | BP | lipid metabolic process |
| 0.572 | GO:0009117 | H | BP | nucleotide metabolic process |
| 0.536 | GO:0046914 | H | MF | transition metal ion binding |
| 0.536 | GO:0044255 | H | BP | cellular lipid metabolic process |
| 0.535 | GO:0005576 | H | CC | extracellular region |
| 0.53 | GO:0016740 | H | MF | transferase activity |
| 0.53 | GO:0031982 | H | CC | vesicle |
| 0.526 | GO:0031988 | H | CC | membrane-bounded vesicle |
| 0.513 | GO:0006810 | H | BP | transport |
| 0.508 | GO:0016462 | H | MF | pyrophosphatase activity |
| 0.965 | GO:0008152 | L | BP | metabolic process |
| 0.954 | GO:0043169 | L | MF | cation binding |
| 0.952 | GO:0044237 | L | BP | cellular metabolic process |
| 0.935 | GO:0005737 | L | CC | cytoplasm |
| 0.914 | GO:0009058 | L | BP | biosynthetic process |
| 0.894 | GO:0046872 | L | MF | metal ion binding |
| 0.891 | GO:0019538 | L | BP | protein metabolic process |
| 0.844 | GO:0050896 | L | BP | response to stimulus |
| 0.835 | GO:0005634 | L | CC | nucleus |
| 0.824 | GO:0036094 | L | MF | small molecule binding |
| 0.819 | GO:0006807 | L | BP | nitrogen compound metabolic process |
| 0.817 | GO:0051716 | L | BP | cellular response to stimulus |
| 0.814 | GO:0034641 | L | BP | cellular nitrogen compound metabolic process |
| 0.813 | GO:0044267 | L | BP | cellular protein metabolic process |
| 0.807 | GO:0046483 | L | BP | heterocycle metabolic process |
| 0.794 | GO:0006725 | L | BP | cellular aromatic compound metabolic process |
| 0.787 | GO:0016787 | L | MF | hydrolase activity |
| 0.785 | GO:0005829 | L | CC | cytosol |
| 0.784 | GO:0006139 | L | BP | nucleobase-containing compound metabolic process |
| 0.783 | GO:0043229 | L | CC | intracellular organelle |
| 0.78 | GO:0043231 | L | CC | intracellular membrane-bounded organelle |
| 0.774 | GO:0032502 | L | BP | developmental process |
| 0.771 | GO:0031981 | L | CC | nuclear lumen |
| 0.767 | GO:0006464 | L | BP | cellular protein modification process |
| 0.74 | GO:0023052 | L | BP | signaling |
| 0.73 | GO:0007154 | L | BP | cell communication |
| 0.715 | GO:0097159 | L | MF | organic cyclic compound binding |
| 0.698 | GO:0005654 | L | CC | nucleoplasm |
| 0.696 | GO:0006996 | L | BP | organelle organization |
| 0.683 | GO:0010467 | L | BP | gene expression |
| 0.662 | GO:0016070 | L | BP | RNA metabolic process |
| 0.618 | GO:0007165 | L | BP | signal transduction |
| 0.607 | GO:0007275 | L | BP | multicellular organismal development |
| 0.569 | GO:0048856 | L | BP | anatomical structure development |
| 0.536 | GO:0032991 | L | CC | macromolecular complex |
| 0.534 | GO:0010033 | L | BP | response to organic substance |
| 0.534 | GO:0005102 | L | MF | receptor binding |

**Blast**

| **BLAST Matched UniProt ID & name** | | | **Identity** | **E Value** | **Score** |
| --- | --- | --- | --- | --- | --- |
| P24484 | LIP2_MORS1 | Lipase 2 | 33.465 | 1.59E-37 | 144 |
| P9WK86 | NLHH_MYCTO | Carboxylesterase NlhH | 32.441 | 1.53E-35 | 136 |
| Q5UQ83 | YR526_MIMIV | Putative alpha/beta hydrolase R526 | 33.755 | 5.02E-32 | 127 |
| Q0TKG5 | AES_ECOL5 | Acetyl esterase | 28.244 | 4.22E-21 | 95.5 |
| Q1RF59 | AES_ECOUT | Acetyl esterase | 28.244 | 4.58E-21 | 95.1 |
| B7UKF6 | AES_ECO27 | Acetyl esterase | 28.244 | 4.99E-21 | 95.1 |
| Q8FK82 | AES_ECOL6 | Acetyl esterase | 28.244 | 1.02E-20 | 94.4 |
| Q325C0 | AES_SHIBS | Acetyl esterase | 28.352 | 2.09E-20 | 93.2 |
| B7MQJ1 | AES_ECO81 | Acetyl esterase | 28.244 | 2.37E-20 | 93.2 |
| B7L7A1 | AES_ECO55 | Acetyl esterase | 28.352 | 2.74E-20 | 92.8 |
| B2U4S9 | AES_SHIB3 | Acetyl esterase | 28.352 | 7.48E-20 | 91.7 |
| A7ZIN6 | AES_ECO24 | Acetyl esterase | 27.969 | 9.25E-20 | 91.7 |
| Q0T7A9 | AES_SHIF8 | Acetyl esterase | 28.352 | 1.29E-19 | 90.9 |
| Q8XD38 | AES_ECO57 | Acetyl esterase | 27.969 | 1.50E-19 | 90.9 |
| B7N929 | AES_ECOLU | Acetyl esterase | 27.969 | 2.94E-19 | 90.1 |
| Q83M39 | AES_SHIFL | Acetyl esterase | 27.969 | 3.28E-19 | 89.7 |
| P23872 | AES_ECOLI | Acetyl esterase | 27.969 | 3.34E-19 | 89.7 |
| Q3Z4S3 | AES_SHISS | Acetyl esterase | 27.969 | 3.80E-19 | 89.7 |
| A7ZXD4 | AES_ECOHS | Acetyl esterase | 27.969 | 4.62E-19 | 89.4 |
| B1IZB8 | AES_ECOLC | Acetyl esterase | 27.586 | 1.28E-18 | 88.2 |
| B4TMG8 | AES_SALSV | Acetyl esterase | 27.16 | 2.62E-17 | 84.7 |
| A9MW81 | AES_SALPB | Acetyl esterase | 27.16 | 4.16E-17 | 84 |
| B5BD42 | AES_SALPK | Acetyl esterase | 27.16 | 4.29E-17 | 84 |
| Q8ZRA1 | AES_SALTY | Acetyl esterase | 27.16 | 4.83E-17 | 84 |
| Q5PFJ2 | AES_SALPA | Acetyl esterase | 27.16 | 4.97E-17 | 84 |
| B5EXN3 | AES_SALA4 | Acetyl esterase | 27.16 | 4.97E-17 | 84 |
| Q57S73 | AES_SALCH | Acetyl esterase | 27.16 | 6.24E-17 | 83.6 |
| B5FLK0 | AES_SALDC | Acetyl esterase | 27.16 | 6.24E-17 | 83.6 |
| P18773 | EST_ACILW | Esterase | 28.194 | 6.43E-17 | 83.2 |
| Q8Z8T1 | AES_SALTI | Acetyl esterase | 27.311 | 2.66E-16 | 81.6 |
| Q9US38 | YFZ3_SCHPO | AB hydrolase superfamily protein C1039.03 | 25.105 | 2.52E-15 | 79.3 |
| Q9LYC1 | GID1B_ARATH | Gibberellin receptor GID1B | 29.187 | 3.55E-14 | 76.3 |
| P22760 | AAAD_HUMAN | Arylacetamide deacetylase | 27.707 | 1.27E-13 | 74.7 |
| Q9LK21 | CXE11_ARATH | Probable carboxylesterase 11 | 25.796 | 1.50E-13 | 74.7 |
| Q8BLF1 | NCEH1_MOUSE | Neutral cholesterol ester hydrolase 1 | 24.49 | 2.95E-13 | 73.9 |
| Q1JQE6 | NCEH1_BOVIN | Neutral cholesterol ester hydrolase 1 | 23.973 | 7.46E-13 | 72.4 |
| B2GV54 | NCEH1_RAT | Neutral cholesterol ester hydrolase 1 | 24.49 | 1.22E-12 | 72 |
| Q9R101 | LIPS_SPETR | Hormone-sensitive lipase | 32.192 | 2.34E-11 | 68.6 |
| Q8BM81 | ADCL4_MOUSE | Arylacetamide deacetylase-like 4 | 30.872 | 2.81E-11 | 67.8 |
| I4DST8 | TCEA1_TULGE | Tuliposide A-converting enzyme 1, chloroplastic | 28.302 | 3.21E-11 | 67.4 |
| Q68J42 | LIPS_PIG | Hormone-sensitive lipase | 32.877 | 3.53E-11 | 68.2 |
| Q9HDX3 | YKN2_SCHPO | AB hydrolase superfamily protein B1A11.02 | 25 | 5.39E-11 | 66.6 |
| P54310 | LIPS_MOUSE | Hormone-sensitive lipase | 33.333 | 6.37E-11 | 67 |
| Q5NUF4 | HIDM_GLYEC | 2-hydroxyisoflavanone dehydratase | 26.182 | 7.34E-11 | 66.2 |
| Q6PIU2 | NCEH1_HUMAN | Neutral cholesterol ester hydrolase 1 | 23.776 | 7.52E-11 | 66.6 |
| Q05469 | LIPS_HUMAN | Hormone-sensitive lipase | 31.69 | 8.68E-11 | 67 |
| Q9MAA7 | GID1A_ARATH | Gibberellin receptor GID1A | 28.279 | 8.72E-11 | 65.9 |
| P15304 | LIPS_RAT | Hormone-sensitive lipase | 32.558 | 8.97E-11 | 67 |
| I4DST9 | TCEA2_TULGE | Tuliposide A-converting enzyme 2, chloroplastic | 28.302 | 9.32E-11 | 66.2 |
| Q6UEG5 | AFLJ_ASPPA | Versiconal hemiacetal acetate esterase | 25.214 | 1.43E-10 | 65.1 |
| Q8LED9 | CXE16_ARATH | Probable carboxylesterase 16 | 25.092 | 1.50E-10 | 65.5 |
| Q00675 | STCI_EMENI | Putative sterigmatocystin biosynthesis lipase/esterase stcI | 25.532 | 1.76E-10 | 64.3 |
| P16386 | LIPS_BOVIN | Hormone-sensitive lipase | 33.871 | 2.86E-10 | 65.1 |
| Q9EX73 | MLHB_RHOER | Monoterpene epsilon-lactone hydrolase | 28.713 | 5.26E-10 | 63.2 |
| Q940G6 | GID1C_ARATH | Gibberellin receptor GID1C | 28.241 | 5.47E-10 | 63.5 |
| Q01109 | BAH_STRHY | Acetyl-hydrolase | 27.236 | 6.87E-10 | 62.8 |
| A2A7Z8 | ADCL3_MOUSE | Arylacetamide deacetylase-like 3 | 31.875 | 8.21E-10 | 63.2 |
| R4X5P0 | TCEB3_TULGE | Tuliposide A-converting enzyme b3, amyloplastic | 26.087 | 1.07E-09 | 62.8 |
| R4X244 | TCEB1_TULGE | Tuliposide A-converting enzyme b1, amyloplastic | 26.087 | 1.18E-09 | 62.8 |
| R4X4V6 | TCEB2_TULGE | Tuliposide A-converting enzyme b2, amyloplastic | 26.087 | 1.18E-09 | 62.8 |
| R4X247 | TCEB4_TULGE | Probable tuliposide A-converting enzyme b6, amyloplastic | 26.087 | 1.36E-09 | 62.4 |
| Q9SX25 | CXE6_ARATH | Probable carboxylesterase 6 | 34.146 | 1.65E-09 | 62 |
| Q7M370 | AAAD_RABIT | Arylacetamide deacetylase | 36.842 | 6.13E-09 | 60.5 |
| Q7M370 | AAAD_RABIT | Arylacetamide deacetylase | 32.394 | 3.14E-04 | 45.8 |
| Q6L545 | GID1_ORYSJ | Gibberellin receptor GID1 | 27.523 | 1.17E-08 | 59.3 |
| Q9LFR7 | CXE17_ARATH | Probable carboxylesterase 17 | 26.809 | 1.69E-08 | 58.9 |
| Q9ZQ91 | CXE7_ARATH | Probable carboxylesterase 7 | 25.1 | 3.72E-08 | 57.8 |
| Q6P093 | ADCL2_HUMAN | Arylacetamide deacetylase-like 2 | 34.416 | 1.14E-07 | 56.6 |
| Q6P093 | ADCL2_HUMAN | Arylacetamide deacetylase-like 2 | 34.615 | 5.65E-06 | 51.2 |
| Q0P5B7 | AAAD_BOVIN | Arylacetamide deacetylase | 35.252 | 1.22E-07 | 56.6 |
| Q0P5B7 | AAAD_BOVIN | Arylacetamide deacetylase | 32.394 | 0.001 | 43.9 |
| Q9LT10 | CXE18_ARATH | Probable carboxylesterase 18 | 32.639 | 1.59E-07 | 55.8 |
| Q9FG13 | CXE15_ARATH | Probable carboxylesterase 15 | 28.358 | 3.78E-07 | 54.7 |
| Q0ZPV7 | CXE1_ACTER | Carboxylesterase 1 | 35.922 | 6.29E-07 | 53.9 |
| Q9LVB8 | CXE20_ARATH | Probable carboxylesterase 120 | 28.743 | 1.16E-06 | 53.1 |
| Q5R8Y5 | NCEH1_PONAB | Neutral cholesterol ester hydrolase 1 | 38.158 | 3.36E-06 | 52 |
| O64640 | CXE8_ARATH | Probable carboxylesterase 8 | 27.273 | 3.00E-05 | 48.9 |
| Q99PG0 | AAAD_MOUSE | Arylacetamide deacetylase | 31.034 | 1.08E-04 | 47.4 |
| Q9LMA7 | CXE1_ARATH | Probable carboxylesterase 1 | 23.571 | 1.09E-04 | 47 |
| Q9SX78 | CXE2_ARATH | Probable carboxylesterase 2 | 24.215 | 1.46E-04 | 46.6 |
| Q9QZH8 | AAAD_RAT | Arylacetamide deacetylase | 28.448 | 1.80E-04 | 46.6 |
| Q9QZH8 | AAAD_RAT | Arylacetamide deacetylase | 32.394 | 5.79E-04 | 45.1 |
| Q9FX92 | CXE3_ARATH | Probable carboxylesterase 3 | 27.907 | 2.21E-04 | 46.2 |
| Q9FX93 | CXE4_ARATH | Probable carboxylesterase 4 | 27.907 | 2.55E-04 | 45.8 |
| Q9SMN0 | CXE12_ARATH | Probable carboxylesterase 12 | 24.889 | 4.65E-04 | 45.1 |
| P37967 | PNBA_BACSU | Para-nitrobenzyl esterase | 26.531 | 7.37E-04 | 44.7 |
| Q9UKY3 | CES1P_HUMAN | Putative inactive carboxylesterase 4 | 25.714 | 0.001 | 43.9 |
| P23141 | EST1_HUMAN | Liver carboxylesterase 1 | 25 | 0.002 | 43.5 |
| P9WK84 | LIPR_MYCTO | Putative acetyl-hydrolase LipR | 24.055 | 0.002 | 43.1 |
| Q9SMM9 | CXE13_ARATH | Probable carboxylesterase 13 | 23.695 | 0.008 | 41.2 |
| Q64176 | EST1E_MOUSE | Carboxylesterase 1E | 23.899 | 0.009 | 41.2 |
| P12337 | EST1_RABIT | Liver carboxylesterase 1 | 23.611 | 0.01 | 41.2 |
| Q8VCT4 | CES1D_MOUSE | Carboxylesterase 1D | 23.611 | 0.019 | 40.4 |
| Q29550 | EST1_PIG | Liver carboxylesterase | 24.306 | 0.02 | 40.4 |
| O46421 | EST1_MACFA | Liver carboxylesterase 1 | 25 | 0.03 | 39.7 |
| P16303 | CES1D_RAT | Carboxylesterase 1D | 24.39 | 0.03 | 39.7 |

**Protein name: dusA-associated integrases**

**Predicted results:**

**SVMProt**

| **Protein Family Name** | **GO Category** | **SVM** | **PNN** | **KNN** |
| --- | --- | --- | --- | --- |
| **Molecular Function** | | | | |
| All DNA-binding | GO:0003677 DNA binding | 95.7 | ? | ? |
| Zinc-binding | GO:0008270 zinc binding | 92.9 | - | - |
| All lipid-binding proteins | GO:0008289 lipid binding | 92.1 | - | - |
| Metal-binding | GO:0046872 metal ion binding | 90.3 | - | - |
| Iron-binding | GO:0005506 iron binding | 82.2 | - | - |
| **Biological Process** | | | | |
| DNA integration | GO:0015074 DNA integration | 91.3 | ? | ? |
| DNA recombination | GO:0006310 DNA recombination | 86.8 | ? | ? |
| DNA repair | GO:0006281 DNA repair | 58.6 | ? | ? |

**FFPred**

| **Score** | **GO term** | **RL** | **Domain** | **Description** |
| --- | --- | --- | --- | --- |
| 0.969 | GO:0003824 | H | MF | catalytic activity |
| 0.862 | GO:0016740 | H | MF | transferase activity |
| 0.83 | GO:0017076 | H | MF | purine nucleotide binding |
| 0.811 | GO:0000166 | H | MF | nucleotide binding |
| 0.794 | GO:0046914 | H | MF | transition metal ion binding |
| 0.789 | GO:0006810 | H | BP | transport |
| 0.784 | GO:0032549 | H | MF | ribonucleoside binding |
| 0.764 | GO:0001883 | H | MF | purine nucleoside binding |
| 0.759 | GO:0003676 | H | MF | nucleic acid binding |
| 0.755 | GO:0030554 | H | MF | adenyl nucleotide binding |
| 0.746 | GO:0035639 | H | MF | purine ribonucleoside triphosphate binding |
| 0.744 | GO:0001882 | H | MF | nucleoside binding |
| 0.741 | GO:0019222 | H | BP | regulation of metabolic process |
| 0.736 | GO:0005739 | H | CC | mitochondrion |
| 0.729 | GO:0005524 | H | MF | ATP binding |
| 0.724 | GO:0006796 | H | BP | phosphate-containing compound metabolic process |
| 0.721 | GO:0030234 | H | MF | enzyme regulator activity |
| 0.71 | GO:0016310 | H | BP | phosphorylation |
| 0.7 | GO:0008092 | H | MF | cytoskeletal protein binding |
| 0.7 | GO:0016817 | H | MF | hydrolase activity, acting on acid anhydrides |
| 0.692 | GO:0006082 | H | BP | organic acid metabolic process |
| 0.658 | GO:0051649 | H | BP | establishment of localization in cell |
| 0.643 | GO:0051641 | H | BP | cellular localization |
| 0.634 | GO:0016301 | H | MF | kinase activity |
| 0.614 | GO:0003723 | H | MF | RNA binding |
| 0.614 | GO:0051020 | H | MF | GTPase binding |
| 0.608 | GO:0045184 | H | BP | establishment of protein localization |
| 0.602 | GO:0031267 | H | MF | small GTPase binding |
| 0.594 | GO:0005740 | H | CC | mitochondrial envelope |
| 0.593 | GO:0044281 | H | BP | small molecule metabolic process |
| 0.587 | GO:0055114 | H | BP | oxidation-reduction process |
| 0.585 | GO:0046907 | H | BP | intracellular transport |
| 0.557 | GO:0016020 | H | CC | membrane |
| 0.554 | GO:0008104 | H | BP | protein localization |
| 0.552 | GO:0010468 | H | BP | regulation of gene expression |
| 0.544 | GO:0015631 | H | MF | tubulin binding |
| 0.542 | GO:0005198 | H | MF | structural molecule activity |
| 0.541 | GO:0048037 | H | MF | cofactor binding |
| 0.538 | GO:0009056 | H | BP | catabolic process |
| 0.537 | GO:0032787 | H | BP | monocarboxylic acid metabolic process |
| 0.512 | GO:0016818 | H | MF | hydrolase activity, acting on acid anhydrides, in phosphorus-containing anhydrides |
| 0.511 | GO:0003735 | H | MF | structural constituent of ribosome |
| 0.511 | GO:0017111 | H | MF | nucleoside-triphosphatase activity |
| 0.51 | GO:0005730 | H | CC | nucleolus |
| 0.507 | GO:0043547 | H | BP | positive regulation of GTPase activity |
| 0.502 | GO:0007005 | H | BP | mitochondrion organization |
| 0.501 | GO:0017016 | H | MF | Ras GTPase binding |
| 0.5 | GO:0031988 | H | CC | membrane-bounded vesicle |
| 0.954 | GO:0005737 | L | CC | cytoplasm |
| 0.951 | GO:0043229 | L | CC | intracellular organelle |
| 0.946 | GO:0044237 | L | BP | cellular metabolic process |
| 0.937 | GO:0097159 | L | MF | organic cyclic compound binding |
| 0.893 | GO:0006139 | L | BP | nucleobase-containing compound metabolic process |
| 0.889 | GO:0008152 | L | BP | metabolic process |
| 0.861 | GO:0006807 | L | BP | nitrogen compound metabolic process |
| 0.848 | GO:0050896 | L | BP | response to stimulus |
| 0.837 | GO:0046872 | L | MF | metal ion binding |
| 0.836 | GO:0043231 | L | CC | intracellular membrane-bounded organelle |
| 0.827 | GO:0009058 | L | BP | biosynthetic process |
| 0.823 | GO:0043169 | L | MF | cation binding |
| 0.822 | GO:0005634 | L | CC | nucleus |
| 0.819 | GO:0051716 | L | BP | cellular response to stimulus |
| 0.808 | GO:0044267 | L | BP | cellular protein metabolic process |
| 0.803 | GO:0019538 | L | BP | protein metabolic process |
| 0.802 | GO:0034641 | L | BP | cellular nitrogen compound metabolic process |
| 0.802 | GO:0032502 | L | BP | developmental process |
| 0.79 | GO:0046483 | L | BP | heterocycle metabolic process |
| 0.788 | GO:0016787 | L | MF | hydrolase activity |
| 0.775 | GO:0006725 | L | BP | cellular aromatic compound metabolic process |
| 0.764 | GO:0005829 | L | CC | cytosol |
| 0.759 | GO:0006996 | L | BP | organelle organization |
| 0.754 | GO:0007165 | L | BP | signal transduction |
| 0.742 | GO:0031981 | L | CC | nuclear lumen |
| 0.729 | GO:0036094 | L | MF | small molecule binding |
| 0.726 | GO:0007154 | L | BP | cell communication |
| 0.712 | GO:0050790 | L | BP | regulation of catalytic activity |
| 0.699 | GO:0019904 | L | MF | protein domain specific binding |
| 0.696 | GO:0005654 | L | CC | nucleoplasm |
| 0.69 | GO:0032991 | L | CC | macromolecular complex |
| 0.668 | GO:0023052 | L | BP | signaling |
| 0.636 | GO:0010467 | L | BP | gene expression |
| 0.635 | GO:0016070 | L | BP | RNA metabolic process |
| 0.632 | GO:0009893 | L | BP | positive regulation of metabolic process |
| 0.632 | GO:0006464 | L | BP | cellular protein modification process |
| 0.606 | GO:0007275 | L | BP | multicellular organismal development |
| 0.59 | GO:0048856 | L | BP | anatomical structure development |
| 0.59 | GO:0009966 | L | BP | regulation of signal transduction |
| 0.541 | GO:0010033 | L | BP | response to organic substance |
| 0.526 | GO:0043234 | L | CC | protein complex |
| 0.522 | GO:0016772 | L | MF | transferase activity, transferring phosphorus-containing groups |
| 0.503 | GO:0043085 | L | BP | positive regulation of catalytic activity |

**Blast**

| **BLAST Matched UniProt ID & name** | | | **Identity** | **E Value** | **Score** |
| --- | --- | --- | --- | --- | --- |
| P16470 | RCI2_ECOLX | Shufflon-specific DNA recombinase | 31.395 | 8.66E-24 | 103 |
| P10487 | RCI1_ECOLX | Shufflon-specific DNA recombinase | 31.395 | 8.91E-24 | 103 |
| P9WMB2 | INT2_MYCTO | Putative prophage phiRv2 integrase | 22.384 | 1.86E-04 | 46.6 |
| B3DQV1 | XERC_BIFLD | Tyrosine recombinase XerC | 32.787 | 6.00E-03 | 41.6 |
| B7GQE1 | XERC_BIFLS | Tyrosine recombinase XerC | 32.787 | 7.00E-03 | 41.6 |
| P24218 | INTD_ECOLI | Prophage DLP12 integrase | 36.364 | 8.00E-03 | 41.6 |

**Protein name: dusA-associated integrases**

**Predicted results:**

**SVMProt**

| **Protein Family Name** | **GO Category** | **SVM** | **PNN** | **KNN** |
| --- | --- | --- | --- | --- |
| **Molecular Function** | | | | |
| All DNA-binding | GO:0003677 DNA binding | 97.7 | ? | ? |
| Iron-binding | GO:0005506 iron binding | 97.5 | - | - |
| EC6.1 Ligases - Forming Carbon-Oxygen Bonds | - | 89.3 | - | - |
| Metal-binding | GO:0046872 metal ion binding | 82.2 | - | - |
| **Biological Process** | | | | |
| DNA integration | GO:0015074 DNA integration | 99.1 | ? | ? |
| DNA recombination | GO:0006310 DNA recombination | 98.4 | ? | ? |
| DNA repair | GO:0006281 DNA repair | 58.6 | ? | ? |
| **Broadly Defined Function** | | | | |
| Photosystem I | GO:0009522 photosystem I | 58.6 | ? | ? |
| Outer membrane | GO:0009279 cell outer membrane | 58.6 | - | - |

**FFPred**

| **Score** | **GO term** | **RL** | **Domain** | **Description** |
| --- | --- | --- | --- | --- |
| 0.87 | GO:0000166 | H | MF | nucleotide binding |
| 0.834 | GO:0003824 | H | MF | catalytic activity |
| 0.825 | GO:0001883 | H | MF | purine nucleoside binding |
| 0.82 | GO:0008092 | H | MF | cytoskeletal protei binding |
| 0.819 | GO:0001882 | H | MF | nucleoside binding |
| 0.79 | GO:0019222 | H | BP | regulatio of metabolic process |
| 0.789 | GO:0016740 | H | MF | transferase activity |
| 0.77 | GO:0005524 | H | MF | ATP binding |
| 0.77 | GO:0017076 | H | MF | purine nucleotide binding |
| 0.759 | GO:0032549 | H | MF | ribonucleoside binding |
| 0.737 | GO:0016301 | H | MF | kinase activity |
| 0.721 | GO:0006810 | H | BP | transport |
| 0.71 | GO:0003779 | H | MF | acti binding |
| 0.694 | GO:0006796 | H | BP | phosphate-containing compound metabolic process |
| 0.691 | GO:0035639 | H | MF | purine ribonucleoside triphosphate binding |
| 0.686 | GO:0044281 | H | BP | small molecule metabolic process |
| 0.683 | GO:0006082 | H | BP | organic acid metabolic process |
| 0.679 | GO:0003676 | H | MF | nucleic acid binding |
| 0.679 | GO:0016310 | H | BP | phosphorylatio |
| 0.674 | GO:0046914 | H | MF | transitio metal io binding |
| 0.663 | GO:0030554 | H | MF | adenyl nucleotide binding |
| 0.652 | GO:0030234 | H | MF | enzyme regulator activity |
| 0.65 | GO:0005198 | H | MF | structural molecule activity |
| 0.632 | GO:0016817 | H | MF | hydrolase activity, acting o acid anhydrides |
| 0.632 | GO:0019439 | H | BP | aromatic compound catabolic process |
| 0.614 | GO:0048667 | H | BP | cell morphogenesis involved i neuro differentiatio |
| 0.606 | GO:0043547 | H | BP | positive regulatio of GTPase activity |
| 0.606 | GO:0010468 | H | BP | regulatio of gene expressio |
| 0.601 | GO:0046395 | H | BP | carboxylic acid catabolic process |
| 0.597 | GO:0051649 | H | BP | establishment of localizatio i cell |
| 0.596 | GO:0055114 | H | BP | oxidation-reductio process |
| 0.594 | GO:0002431 | H | BP | Fc receptor mediated stimulatory signaling pathway |
| 0.586 | GO:0015631 | H | MF | tubuli binding |
| 0.585 | GO:0019901 | H | MF | protei kinase binding |
| 0.582 | GO:0007409 | H | BP | axonogenesis |
| 0.581 | GO:0051641 | H | BP | cellular localizatio |
| 0.571 | GO:0016020 | H | CC | membrane |
| 0.557 | GO:0009056 | H | BP | catabolic process |
| 0.54 | GO:0009059 | H | BP | macromolecule biosynthetic process |
| 0.518 | GO:0019900 | H | MF | kinase binding |
| 0.517 | GO:0006412 | H | BP | translatio |
| 0.516 | GO:0008104 | H | BP | protei localizatio |
| 0.515 | GO:0051056 | H | BP | regulatio of small GTPase mediated signal transduction |
| 0.507 | GO:0003723 | H | MF | RNA binding |
| 0.501 | GO:0045184 | H | BP | establishment of protei localizatio |
| 0.5 | GO:0034645 | H | BP | cellular macromolecule biosynthetic process |
| 0.944 | GO:0044237 | L | BP | cellular metabolic process |
| 0.924 | GO:0043229 | L | CC | intracellular organelle |
| 0.91 | GO:0005737 | L | CC | cytoplasm |
| 0.864 | GO:0008152 | L | BP | metabolic process |
| 0.861 | GO:0019538 | L | BP | protei metabolic process |
| 0.854 | GO:0005634 | L | CC | nucleus |
| 0.849 | GO:0006807 | L | BP | nitroge compound metabolic process |
| 0.844 | GO:0050896 | L | BP | response to stimulus |
| 0.831 | GO:0044267 | L | BP | cellular protei metabolic process |
| 0.819 | GO:0034641 | L | BP | cellular nitroge compound metabolic process |
| 0.817 | GO:0051716 | L | BP | cellular response to stimulus |
| 0.817 | GO:0007154 | L | BP | cell communicatio |
| 0.815 | GO:0046483 | L | BP | heterocycle metabolic process |
| 0.807 | GO:0006139 | L | BP | nucleobase-containing compound metabolic process |
| 0.805 | GO:0032502 | L | BP | developmental process |
| 0.803 | GO:0006725 | L | BP | cellular aromatic compound metabolic process |
| 0.802 | GO:0009058 | L | BP | biosynthetic process |
| 0.8 | GO:0097159 | L | MF | organic cyclic compound binding |
| 0.791 | GO:0046872 | L | MF | metal io binding |
| 0.788 | GO:0031981 | L | CC | nuclear lume |
| 0.78 | GO:0005829 | L | CC | cytosol |
| 0.775 | GO:0007165 | L | BP | signal transductio |
| 0.772 | GO:0023052 | L | BP | signaling |
| 0.769 | GO:0036094 | L | MF | small molecule binding |
| 0.768 | GO:0050790 | L | BP | regulatio of catalytic activity |
| 0.768 | GO:0032991 | L | CC | macromolecular complex |
| 0.766 | GO:0043231 | L | CC | intracellular membrane-bounded organelle |
| 0.762 | GO:0006996 | L | BP | organelle organizatio |
| 0.75 | GO:0005654 | L | CC | nucleoplasm |
| 0.733 | GO:0043169 | L | MF | catio binding |
| 0.729 | GO:0006464 | L | BP | cellular protei modificatio process |
| 0.721 | GO:0016070 | L | BP | RNA metabolic process |
| 0.716 | GO:0048812 | L | BP | neuro projectio morphogenesis |
| 0.703 | GO:0010467 | L | BP | gene expressio |
| 0.688 | GO:0019904 | L | MF | protei domai specific binding |
| 0.675 | GO:0009893 | L | BP | positive regulatio of metabolic process |
| 0.613 | GO:0007275 | L | BP | multicellular organismal development |
| 0.611 | GO:0009966 | L | BP | regulatio of signal transductio |
| 0.599 | GO:0016772 | L | MF | transferase activity, transferring phosphorus-containing groups |
| 0.599 | GO:0016787 | L | MF | hydrolase activity |
| 0.595 | GO:0043234 | L | CC | protei complex |
| 0.586 | GO:0048856 | L | BP | anatomical structure development |
| 0.581 | GO:0042325 | L | BP | regulatio of phosphorylatio |
| 0.541 | GO:0032403 | L | MF | protei complex binding |
| 0.531 | GO:0043085 | L | BP | positive regulatio of catalytic activity |
| 0.523 | GO:0010033 | L | BP | response to organic substance |

**Blast**

| **BLAST Matched UniProt ID & name** | | | **Identity** | **E Value** | **Score** |
| --- | --- | --- | --- | --- | --- |
| P10487 | RCI1_ECOLX | Shufflon-specific DNA recombinase | 32.7 | 1.39E-20 | 94.7 |
| P16470 | RCI2_ECOLX | Shufflon-specific DNA recombinase | 32.7 | 1.50E-20 | 94.7 |
| P24218 | INTD_ECOLI | Prophage DLP12 integrase | 21.807 | 3.88E-05 | 48.5 |
| P04890 | VINT_BPP22 | Integrase | 23.404 | 4.14E-04 | 45.4 |
| Q7ZAJ4 | XERC_STAES | Tyrosine recombinase XerC | 27.059 | 6.56E-04 | 44.7 |
| Q4L5V4 | XERC_STAHJ | Tyrosine recombinase XerC | 32.203 | 0.029 | 39.3 |
| B9DPG4 | XERC_STACT | Tyrosine recombinase XerC | 25 | 0.039 | 38.9 |

**Protein name: dusA-associated integrases**

**Predicted results:**

**SVMProt**

| **Protein Family Name** | **GO Category** | **SVM** | **PNN** | **KNN** |
| --- | --- | --- | --- | --- |
| **Molecular Function** | | | | |
| Iron-binding | GO:0005506 iron binding | 97 | - | - |
| EC6.1 Ligases - Forming Carbon-Oxygen Bonds | - | 65.4 | - | Y |
| All DNA-binding | GO:0003677 DNA binding | 85.4 | ? | ? |
| Metal-binding | GO:0046872 metal ion binding | 83.9 | - | - |
| EC3.1 Hydrolases - Acting on Ester Bonds | - | 83.9 | - | - |
| **Biological Process** | | | | |
| DNA recombination | GO:0006310 DNA recombination | 92.9 | ? | ? |
| DNA integration | GO:0015074 DNA integration | 85.4 | ? | ? |
| **Broadly Defined Function** | | | | |
| Outer membrane | GO:0009279 cell outer membrane | 58.6 | - | - |

**FFPred**

| **Score** | **GO term** | **RL** | **Domain** | **Description** |
| --- | --- | --- | --- | --- |
| 0.981 | GO:0003824 | H | MF | catalytic activity |
| 0.917 | GO:0003676 | H | MF | nucleic acid binding |
| 0.876 | GO:0019222 | H | BP | regulation of metabolic process |
| 0.86 | GO:0000166 | H | MF | nucleotide binding |
| 0.841 | GO:0019439 | H | BP | aromatic compound catabolic process |
| 0.796 | GO:0010468 | H | BP | regulation of gene expression |
| 0.794 | GO:0016773 | H | MF | phosphotransferase activity, alcohol group as acceptor |
| 0.785 | GO:0046914 | H | MF | transition metal ion binding |
| 0.783 | GO:0005524 | H | MF | ATP binding |
| 0.778 | GO:0016740 | H | MF | transferase activity |
| 0.766 | GO:0009056 | H | BP | catabolic process |
| 0.762 | GO:0006796 | H | BP | phosphate-containing compound metabolic process |
| 0.761 | GO:0044281 | H | BP | small molecule metabolic process |
| 0.743 | GO:0032549 | H | MF | ribonucleoside binding |
| 0.738 | GO:0046395 | H | BP | carboxylic acid catabolic process |
| 0.732 | GO:0001883 | H | MF | purine nucleoside binding |
| 0.731 | GO:0006082 | H | BP | organic acid metabolic process |
| 0.72 | GO:0017076 | H | MF | purine nucleotide binding |
| 0.72 | GO:0001882 | H | MF | nucleoside binding |
| 0.716 | GO:0004672 | H | MF | protein kinase activity |
| 0.715 | GO:0016818 | H | MF | hydrolase activity, acting on acid anhydrides, in phosphorus-containing anhydrides |
| 0.7 | GO:0009059 | H | BP | macromolecule biosynthetic process |
| 0.684 | GO:0030234 | H | MF | enzyme regulator activity |
| 0.678 | GO:0005739 | H | CC | mitochondrion |
| 0.677 | GO:0035639 | H | MF | purine ribonucleoside triphosphate binding |
| 0.675 | GO:0034645 | H | BP | cellular macromolecule biosynthetic process |
| 0.666 | GO:0005740 | H | CC | mitochondrial envelope |
| 0.662 | GO:0016301 | H | MF | kinase activity |
| 0.659 | GO:0030554 | H | MF | adenyl nucleotide binding |
| 0.657 | GO:0006351 | H | BP | transcription, DNA-templated |
| 0.653 | GO:0008092 | H | MF | cytoskeletal protein binding |
| 0.653 | GO:0016817 | H | MF | hydrolase activity, acting on acid anhydrides |
| 0.645 | GO:1903506 | H | BP | regulation of nucleic acid-templated transcription |
| 0.642 | GO:0017111 | H | MF | nucleoside-triphosphatase activity |
| 0.638 | GO:0019900 | H | MF | kinase binding |
| 0.636 | GO:0015077 | H | MF | monovalent inorganic cation transmembrane transporter activity |
| 0.625 | GO:0016310 | H | BP | phosphorylation |
| 0.617 | GO:0003723 | H | MF | RNA binding |
| 0.602 | GO:0019901 | H | MF | protein kinase binding |
| 0.602 | GO:0015631 | H | MF | tubulin binding |
| 0.576 | GO:0004674 | H | MF | protein serine/threonine kinase activity |
| 0.575 | GO:0051171 | H | BP | regulation of nitrogen compound metabolic process |
| 0.571 | GO:0006810 | H | BP | transport |
| 0.571 | GO:2001141 | H | BP | regulation of RNA biosynthetic process |
| 0.57 | GO:0030529 | H | CC | ribonucleoprotein complex |
| 0.553 | GO:0044822 | H | MF | poly(A) RNA binding |
| 0.529 | GO:0051641 | H | BP | cellular localization |
| 0.523 | GO:0004386 | H | MF | helicase activity |
| 0.52 | GO:0007005 | H | BP | mitochondrion organization |
| 0.512 | GO:0005244 | H | MF | voltage-gated ion channel activity |
| 0.508 | GO:0031966 | H | CC | mitochondrial membrane |
| 0.505 | GO:0003677 | H | MF | DNA binding |
| 0.958 | GO:0044237 | L | BP | cellular metabolic process |
| 0.949 | GO:0097159 | L | MF | organic cyclic compound binding |
| 0.943 | GO:0043229 | L | CC | intracellular organelle |
| 0.916 | GO:0006807 | L | BP | nitrogen compound metabolic process |
| 0.913 | GO:0006139 | L | BP | nucleobase-containing compound metabolic process |
| 0.908 | GO:0016787 | L | MF | hydrolase activity |
| 0.903 | GO:0008152 | L | BP | metabolic process |
| 0.9 | GO:0005737 | L | CC | cytoplasm |
| 0.889 | GO:0044267 | L | BP | cellular protein metabolic process |
| 0.88 | GO:0019538 | L | BP | protein metabolic process |
| 0.876 | GO:0043231 | L | CC | intracellular membrane-bounded organelle |
| 0.859 | GO:0046872 | L | MF | metal ion binding |
| 0.858 | GO:0009058 | L | BP | biosynthetic process |
| 0.852 | GO:0005634 | L | CC | nucleus |
| 0.842 | GO:0043169 | L | MF | cation binding |
| 0.838 | GO:0050896 | L | BP | response to stimulus |
| 0.829 | GO:0034641 | L | BP | cellular nitrogen compound metabolic process |
| 0.827 | GO:0046483 | L | BP | heterocycle metabolic process |
| 0.815 | GO:0051716 | L | BP | cellular response to stimulus |
| 0.815 | GO:0032502 | L | BP | developmental process |
| 0.812 | GO:0006725 | L | BP | cellular aromatic compound metabolic process |
| 0.796 | GO:0032991 | L | CC | macromolecular complex |
| 0.777 | GO:0036094 | L | MF | small molecule binding |
| 0.769 | GO:0031981 | L | CC | nuclear lumen |
| 0.768 | GO:0006996 | L | BP | organelle organization |
| 0.764 | GO:0023052 | L | BP | signaling |
| 0.746 | GO:0016070 | L | BP | RNA metabolic process |
| 0.744 | GO:0007165 | L | BP | signal transduction |
| 0.742 | GO:0005654 | L | CC | nucleoplasm |
| 0.736 | GO:0005829 | L | CC | cytosol |
| 0.725 | GO:0010467 | L | BP | gene expression |
| 0.713 | GO:0006464 | L | BP | cellular protein modification process |
| 0.708 | GO:0007154 | L | BP | cell communication |
| 0.7 | GO:0050790 | L | BP | regulation of catalytic activity |
| 0.678 | GO:0009893 | L | BP | positive regulation of metabolic process |
| 0.634 | GO:0043234 | L | CC | protein complex |
| 0.621 | GO:0016772 | L | MF | transferase activity, transferring phosphorus-containing groups |
| 0.609 | GO:0007275 | L | BP | multicellular organismal development |
| 0.607 | GO:0019904 | L | MF | protein domain specific binding |
| 0.606 | GO:0032403 | L | MF | protein complex binding |
| 0.59 | GO:0048856 | L | BP | anatomical structure development |
| 0.551 | GO:0009966 | L | BP | regulation of signal transduction |
| 0.542 | GO:0010033 | L | BP | response to organic substance |
| 0.524 | GO:0042325 | L | BP | regulation of phosphorylation |
| 0.503 | GO:0005102 | L | MF | receptor binding |

**Blast**

| **BLAST Matched UniProt ID & name** | | | **Identity** | **E Value** | **Score** |
| --- | --- | --- | --- | --- | --- |
| P16470 | RCI2_ECOLX | Shufflon-specific DNA recombinase | 29.801 | 2.17E-24 | 105 |
| P10487 | RCI1_ECOLX | Shufflon-specific DNA recombinase | 29.801 | 2.26E-24 | 105 |
| P24218 | INTD_ECOLI | Prophage DLP12 integrase | 23.175 | 5.30E-05 | 48.5 |
| P04890 | VINT_BPP22 | Integrase | 26.752 | 0.004 | 42.4 |
| Q7ZAJ4 | XERC_STAES | Tyrosine recombinase XerC | 37.778 | 0.02 | 40 |

**Protein name: phage integrase family protein**

**Predicted results:**

**SVMProt**

| **Protein Family Name** | **GO Category** | **SVM** | **PNN** | **KNN** |
| --- | --- | --- | --- | --- |
| **Molecular Function** | | | | |
| EC6.1 Ligases - Forming Carbon-Oxygen Bonds | - | 82.2 | - | Y |
| All DNA-binding | GO:0003677 DNA binding | 98.6 | ? | ? |
| Iron-binding | GO:0005506 iron binding | 98.4 | - | - |
| Zinc-binding | GO:0008270 zinc binding | 90.3 | - | - |
| Metal-binding | GO:0046872 metal ion binding | 86.8 | - | - |
| All lipid-binding proteins | GO:0008289 lipid binding | 83.9 | - | - |
| Calcium-binding | - | 58.6 | - | Y |
| EC4.1 Lyases - Carbon-Carbon Lyases | - | 58.6 | - | - |
| **Biological Process** | | | | |
| DNA integration | GO:0015074 DNA integration | 98.8 | ? | ? |
| DNA recombination | GO:0006310 DNA recombination | 98.8 | ? | ? |
| DNA repair | GO:0006281 DNA repair | 58.6 | ? | ? |
| Lipid degradation | GO:0016042 lipid catabolic process | 58.6 | ? | ? |
| **Broadly Defined Function** | | | | |
| Photosystem I | GO:0009522 photosystem I | 58.6 | ? | ? |

**FFPred**

| **Score** | **GO term** | **RL** | **Domain** | **Description** |
| --- | --- | --- | --- | --- |
| 0.925 | GO:0003824 | H | MF | catalytic activity |
| 0.923 | GO:0016740 | H | MF | transferase activity |
| 0.883 | GO:0000166 | H | MF | nucleotide binding |
| 0.876 | GO:0008092 | H | MF | cytoskeletal protein binding |
| 0.84 | GO:0001883 | H | MF | purine nucleoside binding |
| 0.838 | GO:0015631 | H | MF | tubulin binding |
| 0.813 | GO:0017076 | H | MF | purine nucleotide binding |
| 0.812 | GO:0032549 | H | MF | ribonucleoside binding |
| 0.796 | GO:0001882 | H | MF | nucleoside binding |
| 0.794 | GO:0003676 | H | MF | nucleic acid binding |
| 0.772 | GO:0006082 | H | BP | organic acid metabolic process |
| 0.748 | GO:0005524 | H | MF | ATP binding |
| 0.745 | GO:0003779 | H | MF | actin binding |
| 0.735 | GO:0035639 | H | MF | purine ribonucleoside triphosphate binding |
| 0.731 | GO:0019222 | H | BP | regulation of metabolic process |
| 0.717 | GO:0044281 | H | BP | small molecule metabolic process |
| 0.696 | GO:0006414 | H | BP | translational elongation |
| 0.686 | GO:0016310 | H | BP | phosphorylation |
| 0.681 | GO:0006810 | H | BP | transport |
| 0.672 | GO:0016817 | H | MF | hydrolase activity, acting on acid anhydrides |
| 0.667 | GO:0046914 | H | MF | transition metal ion binding |
| 0.667 | GO:0006796 | H | BP | phosphate-containing compound metabolic process |
| 0.667 | GO:0003723 | H | MF | RNA binding |
| 0.633 | GO:0005730 | H | CC | nucleolus |
| 0.627 | GO:0034645 | H | BP | cellular macromolecule biosynthetic process |
| 0.624 | GO:0016301 | H | MF | kinase activity |
| 0.61 | GO:0030234 | H | MF | enzyme regulator activity |
| 0.605 | GO:0051641 | H | BP | cellular localization |
| 0.597 | GO:0030554 | H | MF | adenyl nucleotide binding |
| 0.593 | GO:0019900 | H | MF | kinase binding |
| 0.591 | GO:0044822 | H | MF | poly(A) RNA binding |
| 0.588 | GO:0031267 | H | MF | small GTPase binding |
| 0.587 | GO:0051020 | H | MF | GTPase binding |
| 0.564 | GO:0009059 | H | BP | macromolecule biosynthetic process |
| 0.564 | GO:0005198 | H | MF | structural molecule activity |
| 0.554 | GO:1903506 | H | BP | regulation of nucleic acid-templated transcription |
| 0.541 | GO:0009056 | H | BP | catabolic process |
| 0.537 | GO:0048037 | H | MF | cofactor binding |
| 0.536 | GO:0008017 | H | MF | microtubule binding |
| 0.536 | GO:0010468 | H | BP | regulation of gene expression |
| 0.531 | GO:0016020 | H | CC | membrane |
| 0.529 | GO:0051649 | H | BP | establishment of localization in cell |
| 0.528 | GO:0046907 | H | BP | intracellular transport |
| 0.52 | GO:0055114 | H | BP | oxidation-reduction process |
| 0.518 | GO:0016567 | H | BP | protein ubiquitination |
| 0.517 | GO:0019901 | H | MF | protein kinase binding |
| 0.513 | GO:0008104 | H | BP | protein localization |
| 0.513 | GO:0051171 | H | BP | regulation of nitrogen compound metabolic process |
| 0.513 | GO:0045184 | H | BP | establishment of protein localization |
| 0.506 | GO:0016818 | H | MF | hydrolase activity, acting on acid anhydrides, in phosphorus-containing anhydrides |
| 0.952 | GO:0044237 | L | BP | cellular metabolic process |
| 0.952 | GO:0043229 | L | CC | intracellular organelle |
| 0.946 | GO:0005737 | L | CC | cytoplasm |
| 0.936 | GO:0006807 | L | BP | nitrogen compound metabolic process |
| 0.925 | GO:0006139 | L | BP | nucleobase-containing compound metabolic process |
| 0.902 | GO:0008152 | L | BP | metabolic process |
| 0.899 | GO:0097159 | L | MF | organic cyclic compound binding |
| 0.873 | GO:0009058 | L | BP | biosynthetic process |
| 0.862 | GO:0043169 | L | MF | cation binding |
| 0.854 | GO:0005634 | L | CC | nucleus |
| 0.849 | GO:0036094 | L | MF | small molecule binding |
| 0.848 | GO:0046872 | L | MF | metal ion binding |
| 0.844 | GO:0050896 | L | BP | response to stimulus |
| 0.838 | GO:0044267 | L | BP | cellular protein metabolic process |
| 0.832 | GO:0043231 | L | CC | intracellular membrane-bounded organelle |
| 0.83 | GO:0034641 | L | BP | cellular nitrogen compound metabolic process |
| 0.823 | GO:0046483 | L | BP | heterocycle metabolic process |
| 0.82 | GO:0051716 | L | BP | cellular response to stimulus |
| 0.805 | GO:0032502 | L | BP | developmental process |
| 0.801 | GO:0006725 | L | BP | cellular aromatic compound metabolic process |
| 0.796 | GO:0019538 | L | BP | protein metabolic process |
| 0.787 | GO:0031981 | L | CC | nuclear lumen |
| 0.786 | GO:0005829 | L | CC | cytosol |
| 0.775 | GO:0032991 | L | CC | macromolecular complex |
| 0.767 | GO:0050790 | L | BP | regulation of catalytic activity |
| 0.763 | GO:0006996 | L | BP | organelle organization |
| 0.743 | GO:0005654 | L | CC | nucleoplasm |
| 0.739 | GO:0023052 | L | BP | signaling |
| 0.716 | GO:0016070 | L | BP | RNA metabolic process |
| 0.704 | GO:0010467 | L | BP | gene expression |
| 0.7 | GO:0019904 | L | MF | protein domain specific binding |
| 0.693 | GO:0007165 | L | BP | signal transduction |
| 0.648 | GO:0006464 | L | BP | cellular protein modification process |
| 0.646 | GO:0007154 | L | BP | cell communication |
| 0.637 | GO:0016787 | L | MF | hydrolase activity |
| 0.634 | GO:0009893 | L | BP | positive regulation of metabolic process |
| 0.61 | GO:0048856 | L | BP | anatomical structure development |
| 0.609 | GO:0007275 | L | BP | multicellular organismal development |
| 0.597 | GO:0016772 | L | MF | transferase activity, transferring phosphorus-containing groups |
| 0.583 | GO:0032403 | L | MF | protein complex binding |
| 0.582 | GO:0005856 | L | CC | cytoskeleton |
| 0.579 | GO:0009966 | L | BP | regulation of signal transduction |
| 0.532 | GO:0043234 | L | CC | protein complex |
| 0.508 | GO:0010033 | L | BP | response to organic substance |

**Blast**

| **BLAST Matched UniProt ID & name** | | | **Identity** | **E Value** | **Score** |
| --- | --- | --- | --- | --- | --- |
| P16470 | RCI2_ECOLX | Shufflon-specific DNA recombinase | 33.333 | 3.13E-28 | 117 |
| P10487 | RCI1_ECOLX | Shufflon-specific DNA recombinase | 33.333 | 3.64E-28 | 117 |
| P76056 | INTR_ECOLI | Putative lambdoid prophage Rac integrase | 25.926 | 0.003 | 43.1 |

**Protein name: dusA-associated integrases**

**Predicted results:**

**SVMProt**

| **Protein Family Name** | **GO Category** | **SVM** | **PNN** | **KNN** |
| --- | --- | --- | --- | --- |
| **Molecular Function** | | | | |
| All DNA-binding | GO:0003677 DNA binding | 99 | ? | ? |
| EC2.7 Transferases - Transferring Phosphorus-Containing Groups | - | 78.4 | - | Y |
| Iron-binding | GO:0005506 iron binding | 91.3 | - | - |
| Metal-binding | GO:0046872 metal ion binding | 85.4 | - | - |
| All lipid-binding proteins | GO:0008289 lipid binding | 80.4 | - | - |
| EC4.1 Lyases - Carbon-Carbon Lyases | - | 71.3 | - | - |
| Biological Process: |  |  |  |  |
| DNA recombination | GO:0006310 DNA recombination | 98.9 | ? | ? |
| DNA integration | GO:0015074 DNA integration | 91.3 | ? | ? |
| DNA repair | GO:0006281 DNA repair | 58.6 | ? | ? |
| **Broadly Defined Function** | | | | |
| Photosystem I | GO:0009522 photosystem I | 58.6 | ? | ? |

**FFPred**

| **Score** | **GO term** | **RL** | **Domain** | **Description** |
| --- | --- | --- | --- | --- |
| 0.889 | GO:0003824 | H | MF | catalytic activity |
| 0.86 | GO:0016740 | H | MF | transferase activity |
| 0.857 | GO:0000166 | H | MF | nucleotide binding |
| 0.845 | GO:0019222 | H | BP | regulation of metabolic process |
| 0.819 | GO:0008092 | H | MF | cytoskeletal protein binding |
| 0.802 | GO:0015631 | H | MF | tubulin binding |
| 0.797 | GO:0046395 | H | BP | carboxylic acid catabolic process |
| 0.785 | GO:0004674 | H | MF | protein serine/threonine kinase activity |
| 0.762 | GO:0005739 | H | CC | mitochondrion |
| 0.757 | GO:0017076 | H | MF | purine nucleotide binding |
| 0.753 | GO:0016310 | H | BP | phosphorylation |
| 0.749 | GO:0001883 | H | MF | purine nucleoside binding |
| 0.743 | GO:0032549 | H | MF | ribonucleoside binding |
| 0.737 | GO:0006796 | H | BP | phosphate-containing compound metabolic process |
| 0.736 | GO:0003676 | H | MF | nucleic acid binding |
| 0.728 | GO:0001882 | H | MF | nucleoside binding |
| 0.71 | GO:0004672 | H | MF | protein kinase activity |
| 0.697 | GO:0030554 | H | MF | adenyl nucleotide binding |
| 0.696 | GO:0009059 | H | BP | macromolecule biosynthetic process |
| 0.687 | GO:0009056 | H | BP | catabolic process |
| 0.686 | GO:0005524 | H | MF | ATP binding |
| 0.685 | GO:0044281 | H | BP | small molecule metabolic process |
| 0.679 | GO:0035639 | H | MF | purine ribonucleoside triphosphate binding |
| 0.676 | GO:0006810 | H | BP | transport |
| 0.65 | GO:0019901 | H | MF | protein kinase binding |
| 0.634 | GO:0019900 | H | MF | kinase binding |
| 0.633 | GO:0051641 | H | BP | cellular localization |
| 0.625 | GO:0010468 | H | BP | regulation of gene expression |
| 0.617 | GO:0016773 | H | MF | phosphotransferase activity, alcohol group as acceptor |
| 0.614 | GO:0030234 | H | MF | enzyme regulator activity |
| 0.612 | GO:0051649 | H | BP | establishment of localization in cell |
| 0.607 | GO:0016301 | H | MF | kinase activity |
| 0.598 | GO:0035556 | H | BP | intracellular signal transduction |
| 0.589 | GO:0003723 | H | MF | RNA binding |
| 0.583 | GO:0019439 | H | BP | aromatic compound catabolic process |
| 0.581 | GO:0044822 | H | MF | poly(A) RNA binding |
| 0.581 | GO:0006082 | H | BP | organic acid metabolic process |
| 0.578 | GO:0016020 | H | CC | membrane |
| 0.57 | GO:0016462 | H | MF | pyrophosphatase activity |
| 0.57 | GO:1903506 | H | BP | regulation of nucleic acid-templated transcription |
| 0.567 | GO:0003779 | H | MF | actin binding |
| 0.563 | GO:0016817 | H | MF | hydrolase activity, acting on acid anhydrides |
| 0.556 | GO:0043547 | H | BP | positive regulation of GTPase activity |
| 0.553 | GO:0046907 | H | BP | intracellular transport |
| 0.546 | GO:0017111 | H | MF | nucleoside-triphosphatase activity |
| 0.545 | GO:0034645 | H | BP | cellular macromolecule biosynthetic process |
| 0.544 | GO:0043241 | H | BP | protein complex disassembly |
| 0.533 | GO:0017016 | H | MF | Ras GTPase binding |
| 0.526 | GO:0043087 | H | BP | regulation of GTPase activity |
| 0.522 | GO:0051171 | H | BP | regulation of nitrogen compound metabolic process |
| 0.518 | GO:2001141 | H | BP | regulation of RNA biosynthetic process |
| 0.506 | GO:0019752 | H | BP | carboxylic acid metabolic process |
| 0.506 | GO:0051020 | H | MF | GTPase binding |
| 0.502 | GO:0031988 | H | CC | membrane-bounded vesicle |
| 0.5 | GO:0006351 | H | BP | transcription, DNA-templated |
| 0.955 | GO:0043229 | L | CC | intracellular organelle |
| 0.942 | GO:0044237 | L | BP | cellular metabolic process |
| 0.932 | GO:0097159 | L | MF | organic cyclic compound binding |
| 0.922 | GO:0005737 | L | CC | cytoplasm |
| 0.883 | GO:0007165 | L | BP | signal transduction |
| 0.881 | GO:0006139 | L | BP | nucleobase-containing compound metabolic process |
| 0.864 | GO:0009058 | L | BP | biosynthetic process |
| 0.861 | GO:0016787 | L | MF | hydrolase activity |
| 0.86 | GO:0043231 | L | CC | intracellular membrane-bounded organelle |
| 0.856 | GO:0006807 | L | BP | nitrogen compound metabolic process |
| 0.845 | GO:0050896 | L | BP | response to stimulus |
| 0.844 | GO:0008152 | L | BP | metabolic process |
| 0.839 | GO:0036094 | L | MF | small molecule binding |
| 0.837 | GO:0005634 | L | CC | nucleus |
| 0.83 | GO:0023052 | L | BP | signaling |
| 0.829 | GO:0044267 | L | BP | cellular protein metabolic process |
| 0.825 | GO:0019538 | L | BP | protein metabolic process |
| 0.825 | GO:0046872 | L | MF | metal ion binding |
| 0.819 | GO:0051716 | L | BP | cellular response to stimulus |
| 0.815 | GO:0034641 | L | BP | cellular nitrogen compound metabolic process |
| 0.81 | GO:0007154 | L | BP | cell communication |
| 0.809 | GO:0046483 | L | BP | heterocycle metabolic process |
| 0.792 | GO:0032502 | L | BP | developmental process |
| 0.787 | GO:0006725 | L | BP | cellular aromatic compound metabolic process |
| 0.777 | GO:0006996 | L | BP | organelle organization |
| 0.77 | GO:0019904 | L | MF | protein domain specific binding |
| 0.762 | GO:0050790 | L | BP | regulation of catalytic activity |
| 0.76 | GO:0005829 | L | CC | cytosol |
| 0.757 | GO:0043169 | L | MF | cation binding |
| 0.757 | GO:0031981 | L | CC | nuclear lumen |
| 0.722 | GO:0005654 | L | CC | nucleoplasm |
| 0.689 | GO:0032991 | L | CC | macromolecular complex |
| 0.681 | GO:0016070 | L | BP | RNA metabolic process |
| 0.681 | GO:0009966 | L | BP | regulation of signal transduction |
| 0.675 | GO:0010467 | L | BP | gene expression |
| 0.626 | GO:0006464 | L | BP | cellular protein modification process |
| 0.625 | GO:0005102 | L | MF | receptor binding |
| 0.616 | GO:0009893 | L | BP | positive regulation of metabolic process |
| 0.61 | GO:0007275 | L | BP | multicellular organismal development |
| 0.59 | GO:0048856 | L | BP | anatomical structure development |
| 0.554 | GO:0043234 | L | CC | protein complex |
| 0.538 | GO:0016772 | L | MF | transferase activity, transferring phosphorus-containing groups |
| 0.534 | GO:0032403 | L | MF | protein complex binding |
| 0.531 | GO:0010033 | L | BP | response to organic substance |
| 0.527 | GO:0071310 | L | BP | cellular response to organic substance |
| 0.516 | GO:0043085 | L | BP | positive regulation of catalytic activity |

**Blast**

| **BLAST Matched UniProt ID & name** | | | **Identity** | **E Value** | **Score** |
| --- | --- | --- | --- | --- | --- |
| P10487 | RCI1_ECOLX | Shufflon-specific DNA recombinase | 24.917 | 9.77E-13 | 72.4 |
| P16470 | RCI2_ECOLX | Shufflon-specific DNA recombinase | 24.917 | 9.86E-13 | 72.4 |
| P24218 | INTD_ECOLI | Prophage DLP12 integrase | 38 | 8.82E-04 | 44.7 |
| P04890 | VINT_BPP22 | Integrase | 39.623 | 0.002 | 43.1 |

**Protein name: dusA-associated integrases**

**Predicted results:**

**SVMProt**

| **Protein Family Name** | **GO Category** | **SVM** | **PNN** | **KNN** |
| --- | --- | --- | --- | --- |
| **Molecular Function** | | | | |
| Zinc-binding | GO:0008270 zinc binding | 99.1 | - | - |
| All DNA-binding | GO:0003677 DNA binding | 97 | ? | ? |
| EC2.7 Transferases - Transferring Phosphorus-Containing Groups | - | 62.2 | - | Y |
| Metal-binding | GO:0046872 metal ion binding | 82.2 | - | - |
| Magnesium-binding | GO:0000287 magnesium binding | 58.6 | ? | ? |
| **Biological Process** | | | | |
| DNA recombination | GO:0006310 DNA recombination | 97.7 | ? | ? |
| DNA repair | GO:0006281 DNA repair | 58.6 | ? | ? |

**FFPred**

| **Score** | **GO term** | **RL** | **Domain** | **Description** |
| --- | --- | --- | --- | --- |
| 0.954 | GO:0003824 | H | MF | catalytic activity |
| 0.865 | GO:0000166 | H | MF | nucleotide binding |
| 0.857 | GO:0044281 | H | BP | small molecule metabolic process |
| 0.848 | GO:0006082 | H | BP | organic acid metabolic process |
| 0.843 | GO:0016740 | H | MF | transferase activity |
| 0.842 | GO:0008092 | H | MF | cytoskeletal protein binding |
| 0.836 | GO:0019222 | H | BP | regulation of metabolic process |
| 0.835 | GO:0006810 | H | BP | transport |
| 0.821 | GO:0046395 | H | BP | carboxylic acid catabolic process |
| 0.813 | GO:0001883 | H | MF | purine nucleoside binding |
| 0.808 | GO:0032549 | H | MF | ribonucleoside binding |
| 0.802 | GO:0017076 | H | MF | purine nucleotide binding |
| 0.797 | GO:0001882 | H | MF | nucleoside binding |
| 0.78 | GO:0003676 | H | MF | nucleic acid binding |
| 0.773 | GO:0016310 | H | BP | phosphorylation |
| 0.769 | GO:0045184 | H | BP | establishment of protein localization |
| 0.76 | GO:0005739 | H | CC | mitochondrion |
| 0.749 | GO:0016301 | H | MF | kinase activity |
| 0.744 | GO:0035639 | H | MF | purine ribonucleoside triphosphate binding |
| 0.727 | GO:0051649 | H | BP | establishment of localization in cell |
| 0.713 | GO:0005524 | H | MF | ATP binding |
| 0.71 | GO:0003779 | H | MF | actin binding |
| 0.691 | GO:0030554 | H | MF | adenyl nucleotide binding |
| 0.664 | GO:0015631 | H | MF | tubulin binding |
| 0.652 | GO:0051641 | H | BP | cellular localization |
| 0.647 | GO:0030234 | H | MF | enzyme regulator activity |
| 0.637 | GO:0009059 | H | BP | macromolecule biosynthetic process |
| 0.635 | GO:0017111 | H | MF | nucleoside-triphosphatase activity |
| 0.624 | GO:0009056 | H | BP | catabolic process |
| 0.623 | GO:0006091 | H | BP | generation of precursor metabolites and energy |
| 0.617 | GO:0046907 | H | BP | intracellular transport |
| 0.615 | GO:0006796 | H | BP | phosphate-containing compound metabolic process |
| 0.614 | GO:0016817 | H | MF | hydrolase activity, acting on acid anhydrides |
| 0.609 | GO:0010468 | H | BP | regulation of gene expression |
| 0.601 | GO:0043547 | H | BP | positive regulation of GTPase activity |
| 0.594 | GO:0016462 | H | MF | pyrophosphatase activity |
| 0.59 | GO:0008104 | H | BP | protein localization |
| 0.588 | GO:0019901 | H | MF | protein kinase binding |
| 0.584 | GO:0005083 | H | MF | small GTPase regulator activity |
| 0.579 | GO:0046914 | H | MF | transition metal ion binding |
| 0.578 | GO:0016818 | H | MF | hydrolase activity, acting on acid anhydrides, in phosphorus-containing anhydrides |
| 0.576 | GO:0034645 | H | BP | cellular macromolecule biosynthetic process |
| 0.564 | GO:0015031 | H | BP | protein transport |
| 0.552 | GO:0019900 | H | MF | kinase binding |
| 0.536 | GO:0005730 | H | CC | nucleolus |
| 0.535 | GO:0016773 | H | MF | phosphotransferase activity, alcohol group as acceptor |
| 0.531 | GO:0016020 | H | CC | membrane |
| 0.525 | GO:0055114 | H | BP | oxidation-reduction process |
| 0.521 | GO:0043087 | H | BP | regulation of GTPase activity |
| 0.515 | GO:0019439 | H | BP | aromatic compound catabolic process |
| 0.515 | GO:0044255 | H | BP | cellular lipid metabolic process |
| 0.514 | GO:0035556 | H | BP | intracellular signal transduction |
| 0.507 | GO:0051056 | H | BP | regulation of small GTPase mediated signal transduction |
| 0.501 | GO:0005576 | H | CC | extracellular region |
| 0.962 | GO:0043229 | L | CC | intracellular organelle |
| 0.94 | GO:0044237 | L | BP | cellular metabolic process |
| 0.929 | GO:0005737 | L | CC | cytoplasm |
| 0.919 | GO:0006139 | L | BP | nucleobase-containing compound metabolic process |
| 0.892 | GO:0097159 | L | MF | organic cyclic compound binding |
| 0.891 | GO:0043231 | L | CC | intracellular membrane-bounded organelle |
| 0.876 | GO:0016787 | L | MF | hydrolase activity |
| 0.873 | GO:0006807 | L | BP | nitrogen compound metabolic process |
| 0.855 | GO:0007165 | L | BP | signal transduction |
| 0.843 | GO:0050896 | L | BP | response to stimulus |
| 0.84 | GO:0005634 | L | CC | nucleus |
| 0.839 | GO:0008152 | L | BP | metabolic process |
| 0.838 | GO:0023052 | L | BP | signaling |
| 0.836 | GO:0032502 | L | BP | developmental process |
| 0.834 | GO:0009058 | L | BP | biosynthetic process |
| 0.817 | GO:0051716 | L | BP | cellular response to stimulus |
| 0.816 | GO:0034641 | L | BP | cellular nitrogen compound metabolic process |
| 0.816 | GO:0007154 | L | BP | cell communication |
| 0.809 | GO:0046483 | L | BP | heterocycle metabolic process |
| 0.807 | GO:0019538 | L | BP | protein metabolic process |
| 0.804 | GO:0036094 | L | MF | small molecule binding |
| 0.796 | GO:0044267 | L | BP | cellular protein metabolic process |
| 0.789 | GO:0006725 | L | BP | cellular aromatic compound metabolic process |
| 0.789 | GO:0006996 | L | BP | organelle organization |
| 0.765 | GO:0043169 | L | MF | cation binding |
| 0.755 | GO:0032991 | L | CC | macromolecular complex |
| 0.753 | GO:0005829 | L | CC | cytosol |
| 0.753 | GO:0031981 | L | CC | nuclear lumen |
| 0.75 | GO:0050790 | L | BP | regulation of catalytic activity |
| 0.72 | GO:0005654 | L | CC | nucleoplasm |
| 0.695 | GO:0019904 | L | MF | protein domain specific binding |
| 0.69 | GO:0016070 | L | BP | RNA metabolic process |
| 0.688 | GO:0043234 | L | CC | protein complex |
| 0.684 | GO:0010467 | L | BP | gene expression |
| 0.667 | GO:0016772 | L | MF | transferase activity, transferring phosphorus-containing groups |
| 0.64 | GO:0046872 | L | MF | metal ion binding |
| 0.612 | GO:0048856 | L | BP | anatomical structure development |
| 0.61 | GO:0007275 | L | BP | multicellular organismal development |
| 0.596 | GO:0009893 | L | BP | positive regulation of metabolic process |
| 0.591 | GO:0005102 | L | MF | receptor binding |
| 0.569 | GO:0071310 | L | BP | cellular response to organic substance |
| 0.569 | GO:0006464 | L | BP | cellular protein modification process |
| 0.547 | GO:0010033 | L | BP | response to organic substance |
| 0.54 | GO:0009966 | L | BP | regulation of signal transduction |
| 0.534 | GO:0032403 | L | MF | protein complex binding |
| 0.524 | GO:0030659 | L | CC | cytoplasmic vesicle membrane |
| 0.513 | GO:0031410 | L | CC | cytoplasmic vesicle |

**Blast**

| **BLAST Matched UniProt ID & name** | | | **Identity** | **E Value** | **Score** |
| --- | --- | --- | --- | --- | --- |
| P10487 | RCI1_ECOLX | Shufflon-specific DNA recombinase | 24.595 | 3.86E-11 | 67.4 |
| P16470 | RCI2_ECOLX | Shufflon-specific DNA recombinase | 24.595 | 3.86E-11 | 67.4 |
| P04890 | VINT_BPP22 | Integrase | 24.468 | 1.98E-04 | 46.6 |
| P9WMB2 | INT2_MYCTO | Putative prophage phiRv2 integrase | 31.707 | 1.70E-02 | 40.4 |
| P24218 | INTD_ECOLI | Prophage DLP12 integrase | 36.735 | 1.70E-02 | 40.4 |

**Protein name: SimC7**

**Predicted results:**

**SVMProt**

| **Protein Family Name** | **GO Category** | **SVM** | **PNN** | **KNN** |
| --- | --- | --- | --- | --- |
| **Molecular Function** | | | | |
| EC2.5 Transferases - Transferring Alkyl or Aryl Groups, Other than Methyl Groups | - | 97.5 | - | Y |
| EC6.3 Ligases - Forming Carbon-Nitrogen Bonds | - | 78.4 | - | Y |
| EC2.3 Transferases - Acyltransferases | - | 95.2 | - | - |
| EC4.1 Lyases - Carbon-Carbon Lyases | - | 71.3 | - | Y |
| EC4.2 Lyases - Carbon-Oxygen Lyases | - | 68.5 | - | Y |
| EC2.1 Transferases - Transferring One-Carbon Groups | - | 58.6 | - | Y |
| Magnesium-binding | GO:0000287 magnesium binding | 58.6 | ? | ? |
| Calcium-binding | - | 58.6 | - | - |

**FFPred**

| **Score** | **GO term** | **RL** | **Domain** | **Description** |
| --- | --- | --- | --- | --- |
| 0.986 | GO:0003824 | H | MF | catalytic activity |
| 0.973 | GO:0019752 | H | BP | carboxylic acid metabolic process |
| 0.957 | GO:0016491 | H | MF | oxidoreductase activity |
| 0.955 | GO:0032787 | H | BP | monocarboxylic acid metabolic process |
| 0.947 | GO:0005739 | H | CC | mitochondrion |
| 0.942 | GO:0044281 | H | BP | small molecule metabolic process |
| 0.937 | GO:0009056 | H | BP | catabolic process |
| 0.928 | GO:0055114 | H | BP | oxidation-reduction process |
| 0.925 | GO:0031966 | H | CC | mitochondrial membrane |
| 0.91 | GO:0046395 | H | BP | carboxylic acid catabolic process |
| 0.895 | GO:0005576 | H | CC | extracellular region |
| 0.885 | GO:0005740 | H | CC | mitochondrial envelope |
| 0.86 | GO:0055086 | H | BP | nucleobase-containing small molecule metabolic process |
| 0.859 | GO:0005975 | H | BP | carbohydrate metabolic process |
| 0.847 | GO:0006796 | H | BP | phosphate-containing compound metabolic process |
| 0.844 | GO:0006631 | H | BP | fatty acid metabolic process |
| 0.835 | GO:1901605 | H | BP | alpha-amino acid metabolic process |
| 0.835 | GO:0006082 | H | BP | organic acid metabolic process |
| 0.831 | GO:0017076 | H | MF | purine nucleotide binding |
| 0.825 | GO:0032549 | H | MF | ribonucleoside binding |
| 0.823 | GO:0070062 | H | CC | extracellular vesicular exosome |
| 0.814 | GO:0031988 | H | CC | membrane-bounded vesicle |
| 0.802 | GO:0009117 | H | BP | nucleotide metabolic process |
| 0.8 | GO:0000166 | H | MF | nucleotide binding |
| 0.792 | GO:0006629 | H | BP | lipid metabolic process |
| 0.772 | GO:0009165 | H | BP | nucleotide biosynthetic process |
| 0.765 | GO:0001882 | H | MF | nucleoside binding |
| 0.762 | GO:0048037 | H | MF | cofactor binding |
| 0.76 | GO:0035639 | H | MF | purine ribonucleoside triphosphate binding |
| 0.756 | GO:0044255 | H | BP | cellular lipid metabolic process |
| 0.751 | GO:0031982 | H | CC | vesicle |
| 0.737 | GO:0001883 | H | MF | purine nucleoside binding |
| 0.731 | GO:0005524 | H | MF | ATP binding |
| 0.725 | GO:0006163 | H | BP | purine nucleotide metabolic process |
| 0.718 | GO:0019637 | H | BP | organophosphate metabolic process |
| 0.711 | GO:0016740 | H | MF | transferase activity |
| 0.649 | GO:0016788 | H | MF | hydrolase activity, acting on ester bonds |
| 0.635 | GO:0005759 | H | CC | mitochondrial matrix |
| 0.627 | GO:0009116 | H | BP | nucleoside metabolic process |
| 0.601 | GO:0016817 | H | MF | hydrolase activity, acting on acid anhydrides |
| 0.597 | GO:0008610 | H | BP | lipid biosynthetic process |
| 0.594 | GO:0009259 | H | BP | ribonucleotide metabolic process |
| 0.583 | GO:0051186 | H | BP | cofactor metabolic process |
| 0.581 | GO:0007005 | H | BP | mitochondrion organization |
| 0.577 | GO:0005996 | H | BP | monosaccharide metabolic process |
| 0.571 | GO:0050662 | H | MF | coenzyme binding |
| 0.56 | GO:0005743 | H | CC | mitochondrial inner membrane |
| 0.535 | GO:0016462 | H | MF | pyrophosphatase activity |
| 0.526 | GO:0006520 | H | BP | cellular amino acid metabolic process |
| 0.522 | GO:0030554 | H | MF | adenyl nucleotide binding |
| 0.517 | GO:0017111 | H | MF | nucleoside-triphosphatase activity |
| 0.516 | GO:0000287 | H | MF | magnesium ion binding |
| 0.51 | GO:0019222 | H | BP | regulation of metabolic process |
| 0.974 | GO:0008152 | L | BP | metabolic process |
| 0.963 | GO:0005737 | L | CC | cytoplasm |
| 0.959 | GO:0044237 | L | BP | cellular metabolic process |
| 0.959 | GO:0009058 | L | BP | biosynthetic process |
| 0.954 | GO:0006807 | L | BP | nitrogen compound metabolic process |
| 0.947 | GO:0043229 | L | CC | intracellular organelle |
| 0.903 | GO:0043231 | L | CC | intracellular membrane-bounded organelle |
| 0.869 | GO:0050896 | L | BP | response to stimulus |
| 0.868 | GO:0043169 | L | MF | cation binding |
| 0.832 | GO:0051716 | L | BP | cellular response to stimulus |
| 0.826 | GO:0016787 | L | MF | hydrolase activity |
| 0.822 | GO:0046872 | L | MF | metal ion binding |
| 0.82 | GO:0036094 | L | MF | small molecule binding |
| 0.817 | GO:0034641 | L | BP | cellular nitrogen compound metabolic process |
| 0.803 | GO:0005634 | L | CC | nucleus |
| 0.793 | GO:0046483 | L | BP | heterocycle metabolic process |
| 0.769 | GO:0006725 | L | BP | cellular aromatic compound metabolic process |
| 0.733 | GO:0005829 | L | CC | cytosol |
| 0.702 | GO:0031981 | L | CC | nuclear lumen |
| 0.684 | GO:0006996 | L | BP | organelle organization |
| 0.683 | GO:0044267 | L | BP | cellular protein metabolic process |
| 0.649 | GO:0005654 | L | CC | nucleoplasm |
| 0.642 | GO:0010467 | L | BP | gene expression |
| 0.622 | GO:0032502 | L | BP | developmental process |
| 0.621 | GO:0097159 | L | MF | organic cyclic compound binding |
| 0.601 | GO:0007275 | L | BP | multicellular organismal development |
| 0.596 | GO:0016070 | L | BP | RNA metabolic process |
| 0.574 | GO:0006139 | L | BP | nucleobase-containing compound metabolic process |
| 0.56 | GO:0019538 | L | BP | protein metabolic process |
| 0.548 | GO:0023052 | L | BP | signaling |
| 0.522 | GO:0007154 | L | BP | cell communication |
| 0.508 | GO:0044262 | L | BP | cellular carbohydrate metabolic process |
| 0.501 | GO:0006399 | L | BP | tRNA metabolic process |
| 0.5 | GO:0048856 | L | BP | anatomical structure development |

**Blast**

| **BLAST Matched UniProt ID & name** | | | **Identity** | **E Value** | **Score** |
| --- | --- | --- | --- | --- | --- |
| Q8KU07 | AZOB_XENAZ | NAD | 39.194 | 3.89E-34 | 130 |
| Q54LW0 | PADA_DICDI | Prestalk A differentiation protein A | 23.368 | 1.44E-17 | 83.6 |
| Q9S9N9 | CCR1_ARATH | Cinnamoyl-CoA reductase 1 | 33.071 | 4.09E-07 | 53.9 |
| Q9SAH9 | CCR2_ARATH | Cinnamoyl-CoA reductase 2 | 32.54 | 1.77E-06 | 52 |
| Q94EG6 | Y5224_ARATH | Uncharacterized protein At5g02240 | 25 | 7.99E-05 | 46.6 |
| O80934 | Y2766_ARATH | Uncharacterized protein At2g37660, chloroplastic | 26.977 | 1.86E-04 | 45.8 |
| P51105 | DFRA_GERHY | Dihydroflavonol-4-reductase | 33.75 | 0.003 | 42.4 |
| P51103 | DFRA_CALCH | Dihydroflavonol-4-reductase | 30.579 | 5.00E-03 | 41.6 |
| P51102 | DFRA_ARATH | Dihydroflavonol-4-reductase | 25.698 | 5.00E-03 | 41.2 |
| P51104 | DFRA_DIACA | Dihydroflavonol-4-reductase | 27.068 | 1.30E-02 | 40 |
| P14720 | DFRA_PETHY | Dihydroflavonol-4-reductase | 33.75 | 1.70E-02 | 39.7 |
| Q8SKU2 | TIC62_PEA | Protein TIC 62, chloroplastic | 27.011 | 2.90E-02 | 39.3 |
| P14721 | DFRA_ANTMA | Dihydroflavonol-4-reductase | 33.333 | 3.40E-02 | 38.9 |
| Q2SYI1 | RMLD_BURTA | dTDP-4-dehydrorhamnose reductase | 47.541 | 4.70E-02 | 38.1 |

**Protein name: RecD**

**Predicted results:**

**SVMProt**

| **Protein Family Name** | **GO Category** | **SVM** | **PNN** | **KNN** |
| --- | --- | --- | --- | --- |
| **Molecular Function** | | | | |
| EC2.7 Transferases - Transferring Phosphorus-Containing Groups | - | 78.4 | Y | Y |
| Zinc-binding | GO:0008270 zinc binding | 99.1 | - | - |
| All DNA-binding | GO:0003677 DNA binding | 88.1 | ? | ? |
| EC2.1 Transferases - Transferring One-Carbon Groups | - | 58.6 | - | Y |
| Iron-binding | GO:0005506 iron binding | 78.4 | - | - |
| Magnesium-binding | GO:0000287 magnesium binding | 58.6 | ? | ? |

**FFPred**

| **Score** | **GO term** | **RL** | **Domain** | **Description** |
| --- | --- | --- | --- | --- |
| 0.934 | GO:0031966 | H | CC | mitochondrial membrane |
| 0.923 | GO:0003824 | H | MF | catalytic activity |
| 0.921 | GO:0005739 | H | CC | mitochondrion |
| 0.904 | GO:0055114 | H | BP | oxidation-reduction process |
| 0.901 | GO:0019752 | H | BP | carboxylic acid metabolic process |
| 0.893 | GO:0016491 | H | MF | oxidoreductase activity |
| 0.872 | GO:0044281 | H | BP | small molecule metabolic process |
| 0.867 | GO:0005743 | H | CC | mitochondrial inner membrane |
| 0.866 | GO:0005740 | H | CC | mitochondrial envelope |
| 0.839 | GO:0009056 | H | BP | catabolic process |
| 0.832 | GO:0006082 | H | BP | organic acid metabolic process |
| 0.799 | GO:0000166 | H | MF | nucleotide binding |
| 0.791 | GO:0006790 | H | BP | sulfur compound metabolic process |
| 0.781 | GO:0032787 | H | BP | monocarboxylic acid metabolic process |
| 0.78 | GO:0016020 | H | CC | membrane |
| 0.779 | GO:0016740 | H | MF | transferase activity |
| 0.77 | GO:0001882 | H | MF | nucleoside binding |
| 0.762 | GO:0001883 | H | MF | purine nucleoside binding |
| 0.74 | GO:0017076 | H | MF | purine nucleotide binding |
| 0.739 | GO:0046395 | H | BP | carboxylic acid catabolic process |
| 0.734 | GO:0032549 | H | MF | ribonucleoside binding |
| 0.714 | GO:0035639 | H | MF | purine ribonucleoside triphosphate binding |
| 0.683 | GO:0009117 | H | BP | nucleotide metabolic process |
| 0.665 | GO:0044255 | H | BP | cellular lipid metabolic process |
| 0.644 | GO:0012505 | H | CC | endomembrane system |
| 0.643 | GO:0006629 | H | BP | lipid metabolic process |
| 0.639 | GO:0006520 | H | BP | cellular amino acid metabolic process |
| 0.624 | GO:0019222 | H | BP | regulation of metabolic process |
| 0.621 | GO:0009059 | H | BP | macromolecule biosynthetic process |
| 0.621 | GO:0005789 | H | CC | endoplasmic reticulum membrane |
| 0.616 | GO:0005783 | H | CC | endoplasmic reticulum |
| 0.614 | GO:0055086 | H | BP | nucleobase-containing small molecule metabolic process |
| 0.606 | GO:0070062 | H | CC | extracellular vesicular exosome |
| 0.591 | GO:1901605 | H | BP | alpha-amino acid metabolic process |
| 0.583 | GO:0015980 | H | BP | energy derivation by oxidation of organic compounds |
| 0.573 | GO:0006631 | H | BP | fatty acid metabolic process |
| 0.571 | GO:0005975 | H | BP | carbohydrate metabolic process |
| 0.562 | GO:0034645 | H | BP | cellular macromolecule biosynthetic process |
| 0.555 | GO:0005524 | H | MF | ATP binding |
| 0.555 | GO:0019637 | H | BP | organophosphate metabolic process |
| 0.543 | GO:0016462 | H | MF | pyrophosphatase activity |
| 0.53 | GO:0016746 | H | MF | transferase activity, transferring acyl groups |
| 0.519 | GO:0016874 | H | MF | ligase activity |
| 0.507 | GO:0008610 | H | BP | lipid biosynthetic process |
| 0.504 | GO:0031982 | H | CC | vesicle |
| 0.503 | GO:0016818 | H | MF | hydrolase activity, acting on acid anhydrides, in phosphorus-containing anhydrides |
| 0.963 | GO:0008152 | L | BP | metabolic process |
| 0.954 | GO:0044237 | L | BP | cellular metabolic process |
| 0.95 | GO:0005737 | L | CC | cytoplasm |
| 0.927 | GO:0009058 | L | BP | biosynthetic process |
| 0.89 | GO:0050896 | L | BP | response to stimulus |
| 0.889 | GO:0043229 | L | CC | intracellular organelle |
| 0.879 | GO:0019538 | L | BP | protein metabolic process |
| 0.861 | GO:0043231 | L | CC | intracellular membrane-bounded organelle |
| 0.852 | GO:0006807 | L | BP | nitrogen compound metabolic process |
| 0.843 | GO:0036094 | L | MF | small molecule binding |
| 0.821 | GO:0051716 | L | BP | cellular response to stimulus |
| 0.806 | GO:0043169 | L | MF | cation binding |
| 0.804 | GO:0046872 | L | MF | metal ion binding |
| 0.797 | GO:0044267 | L | BP | cellular protein metabolic process |
| 0.775 | GO:0006139 | L | BP | nucleobase-containing compound metabolic process |
| 0.769 | GO:0006464 | L | BP | cellular protein modification process |
| 0.732 | GO:0023052 | L | BP | signaling |
| 0.712 | GO:0097159 | L | MF | organic cyclic compound binding |
| 0.695 | GO:0032502 | L | BP | developmental process |
| 0.695 | GO:0007165 | L | BP | signal transduction |
| 0.652 | GO:0034641 | L | BP | cellular nitrogen compound metabolic process |
| 0.644 | GO:0009966 | L | BP | regulation of signal transduction |
| 0.617 | GO:0007154 | L | BP | cell communication |
| 0.596 | GO:0007275 | L | BP | multicellular organismal development |
| 0.571 | GO:0005634 | L | CC | nucleus |
| 0.566 | GO:0046483 | L | BP | heterocycle metabolic process |
| 0.554 | GO:0010033 | L | BP | response to organic substance |
| 0.548 | GO:0006725 | L | BP | cellular aromatic compound metabolic process |
| 0.546 | GO:0009893 | L | BP | positive regulation of metabolic process |
| 0.54 | GO:0032991 | L | CC | macromolecular complex |
| 0.521 | GO:0043234 | L | CC | protein complex |

**Blast**

| **BLAST Matched UniProt ID & name** | | | **Identity** | **E Value** | **Score** |
| --- | --- | --- | --- | --- | --- |
| A0QS28 | RECD_MYCS2 | RecBCD enzyme subunit RecD | 69.573 | 0.00E+00 | 640 |
| P04993 | RECD_ECOLI | RecBCD enzyme subunit RecD | 36.661 | 7.10E-73 | 250 |
| Q89AB2 | RECD_BUCBP | RecBCD enzyme subunit RecD | 23.486 | 3.67E-56 | 204 |
| P45158 | RECD_HAEIN | RecBCD enzyme subunit RecD | 31.504 | 1.32E-51 | 192 |
| P57530 | RECD_BUCAI | RecBCD enzyme subunit RecD | 23.952 | 3.54E-36 | 145 |
| Q8K9A8 | RECD_BUCAP | RecBCD enzyme subunit RecD | 20.611 | 1.81E-33 | 137 |
| Q9RT63 | RECDL_DEIRA | ATP-dependent RecD-like DNA helicase | 31.991 | 9.02E-33 | 137 |
| O34481 | RECDL_BACSU | ATP-dependent RecD-like DNA helicase | 26.173 | 1.28E-19 | 96.7 |
| Q8NG08 | HELB_HUMAN | DNA helicase B | 29.767 | 5.33E-11 | 69.3 |
| Q5UQ92 | YR530_MIMIV | Uncharacterized protein R530 | 25.658 | 3.40E-09 | 62.8 |
| P55418 | TRAA_RHISN | Probable conjugal transfer protein TraA | 27.981 | 2.02E-08 | 60.8 |
| Q58914 | Y1519_METJA | Uncharacterized protein MJ1519 | 41.818 | 3.12E-07 | 57 |
| Q6NVF4 | HELB_MOUSE | DNA helicase B | 29.187 | 3.41E-07 | 57 |
| Q44349 | TRAA_AGRFC | Conjugal transfer protein TraA | 31.138 | 7.44E-04 | 46.2 |
| Q44363 | TRAA_RHIRD | Conjugal transfer protein TraA | 30.539 | 2.00E-03 | 44.7 |

**Protein name: CLA-ER**

**Predicted results:**

**SVMProt**

| **Protein Family Name** | **GO Category** | **SVM** | **PNN** | **KNN** |
| --- | --- | --- | --- | --- |
| **Molecular Function** | | | | |
| EC4.1 Lyases - Carbon-Carbon Lyases | - | 90.3 | - | Y |
| EC2.7 Transferases - Transferring Phosphorus-Containing Groups | - | 83.9 | - | Y |
| Iron-binding | GO:0005506 iron binding | 92.9 | - | - |
| All lipid-binding proteins | GO:0008289 lipid binding | 92.1 | - | - |
| EC3.1 Hydrolases - Acting on Ester Bonds | - | 71.3 | - | - |
| EC2.3 Transferases - Acyltransferases | - | 65.4 | - | - |
| EC4.2 Lyases - Carbon-Oxygen Lyases | - | 65.4 | - | - |
| Calcium-binding | - | 58.6 | - | - |
| Magnesium-binding | GO:0000287 magnesium binding | 58.6 | ? | ? |
| **Broadly Defined Function** | | | | |
| Photosystem I | GO:0009522 photosystem I | 58.6 | ? | ? |

**FFPred**

| **Score** | **GO term** | **RL** | **Domain** | **Description** |
| --- | --- | --- | --- | --- |
| 0.936 | GO:0003824 | H | MF | catalytic activity |
| 0.891 | GO:0005576 | H | CC | extracellular region |
| 0.827 | GO:0006810 | H | BP | transport |
| 0.825 | GO:0031982 | H | CC | vesicle |
| 0.812 | GO:0055114 | H | BP | oxidation-reduction process |
| 0.8 | GO:0009116 | H | BP | nucleoside metabolic process |
| 0.798 | GO:0006082 | H | BP | organic acid metabolic process |
| 0.798 | GO:0070062 | H | CC | extracellular vesicular exosome |
| 0.769 | GO:0015980 | H | BP | energy derivation by oxidation of organic compounds |
| 0.764 | GO:0019222 | H | BP | regulation of metabolic process |
| 0.759 | GO:0010468 | H | BP | regulation of gene expression |
| 0.746 | GO:0016740 | H | MF | transferase activity |
| 0.728 | GO:0005739 | H | CC | mitochondrion |
| 0.69 | GO:0031988 | H | CC | membrane-bounded vesicle |
| 0.689 | GO:0051171 | H | BP | regulation of nitrogen compound metabolic process |
| 0.684 | GO:0008092 | H | MF | cytoskeletal protein binding |
| 0.683 | GO:0044281 | H | BP | small molecule metabolic process |
| 0.683 | GO:0003779 | H | MF | actin binding |
| 0.674 | GO:0051252 | H | BP | regulation of RNA metabolic process |
| 0.67 | GO:0000166 | H | MF | nucleotide binding |
| 0.665 | GO:0006412 | H | BP | translation |
| 0.657 | GO:1903506 | H | BP | regulation of nucleic acid-templated transcription |
| 0.654 | GO:0006355 | H | BP | regulation of transcription, DNA-templated |
| 0.65 | GO:0034645 | H | BP | cellular macromolecule biosynthetic process |
| 0.649 | GO:0030554 | H | MF | adenyl nucleotide binding |
| 0.642 | GO:0006091 | H | BP | generation of precursor metabolites and energy |
| 0.641 | GO:0009059 | H | BP | macromolecule biosynthetic process |
| 0.64 | GO:2001141 | H | BP | regulation of RNA biosynthetic process |
| 0.624 | GO:0016020 | H | CC | membrane |
| 0.624 | GO:0006796 | H | BP | phosphate-containing compound metabolic process |
| 0.601 | GO:0045333 | H | BP | cellular respiration |
| 0.599 | GO:0055086 | H | BP | nucleobase-containing small molecule metabolic process |
| 0.589 | GO:0003676 | H | MF | nucleic acid binding |
| 0.588 | GO:0016817 | H | MF | hydrolase activity, acting on acid anhydrides |
| 0.586 | GO:0051641 | H | BP | cellular localization |
| 0.572 | GO:0001883 | H | MF | purine nucleoside binding |
| 0.554 | GO:0031090 | H | CC | organelle membrane |
| 0.545 | GO:0009117 | H | BP | nucleotide metabolic process |
| 0.519 | GO:0017076 | H | MF | purine nucleotide binding |
| 0.518 | GO:0046907 | H | BP | intracellular transport |
| 0.515 | GO:0034613 | H | BP | cellular protein localization |
| 0.512 | GO:0035556 | H | BP | intracellular signal transduction |
| 0.512 | GO:0009056 | H | BP | catabolic process |
| 0.509 | GO:0035639 | H | MF | purine ribonucleoside triphosphate binding |
| 0.504 | GO:0005215 | H | MF | transporter activity |
| 0.502 | GO:0016818 | H | MF | hydrolase activity, acting on acid anhydrides, in phosphorus-containing anhydrides |
| 0.5 | GO:0017111 | H | MF | nucleoside-triphosphatase activity |
| 0.94 | GO:0005737 | L | CC | cytoplasm |
| 0.939 | GO:0097159 | L | MF | organic cyclic compound binding |
| 0.933 | GO:0043229 | L | CC | intracellular organelle |
| 0.929 | GO:0008152 | L | BP | metabolic process |
| 0.911 | GO:0043231 | L | CC | intracellular membrane-bounded organelle |
| 0.897 | GO:0044237 | L | BP | cellular metabolic process |
| 0.87 | GO:0019538 | L | BP | protein metabolic process |
| 0.861 | GO:0050896 | L | BP | response to stimulus |
| 0.846 | GO:0023052 | L | BP | signaling |
| 0.844 | GO:0009058 | L | BP | biosynthetic process |
| 0.828 | GO:0051716 | L | BP | cellular response to stimulus |
| 0.827 | GO:0005634 | L | CC | nucleus |
| 0.822 | GO:0046872 | L | MF | metal ion binding |
| 0.82 | GO:0032502 | L | BP | developmental process |
| 0.818 | GO:0034641 | L | BP | cellular nitrogen compound metabolic process |
| 0.801 | GO:0007165 | L | BP | signal transduction |
| 0.796 | GO:0046483 | L | BP | heterocycle metabolic process |
| 0.787 | GO:0050790 | L | BP | regulation of catalytic activity |
| 0.786 | GO:0032991 | L | CC | macromolecular complex |
| 0.776 | GO:0006725 | L | BP | cellular aromatic compound metabolic process |
| 0.773 | GO:0005829 | L | CC | cytosol |
| 0.77 | GO:0043169 | L | MF | cation binding |
| 0.743 | GO:0006807 | L | BP | nitrogen compound metabolic process |
| 0.736 | GO:0031981 | L | CC | nuclear lumen |
| 0.733 | GO:0043234 | L | CC | protein complex |
| 0.727 | GO:0044267 | L | BP | cellular protein metabolic process |
| 0.721 | GO:0006996 | L | BP | organelle organization |
| 0.703 | GO:0007154 | L | BP | cell communication |
| 0.7 | GO:0036094 | L | MF | small molecule binding |
| 0.687 | GO:0009893 | L | BP | positive regulation of metabolic process |
| 0.672 | GO:0010467 | L | BP | gene expression |
| 0.666 | GO:0005654 | L | CC | nucleoplasm |
| 0.644 | GO:0016070 | L | BP | RNA metabolic process |
| 0.641 | GO:0005102 | L | MF | receptor binding |
| 0.64 | GO:0048856 | L | BP | anatomical structure development |
| 0.592 | GO:0007275 | L | BP | multicellular organismal development |
| 0.578 | GO:0006139 | L | BP | nucleobase-containing compound metabolic process |
| 0.568 | GO:0016787 | L | MF | hydrolase activity |
| 0.547 | GO:0006464 | L | BP | cellular protein modification process |
| 0.538 | GO:0009966 | L | BP | regulation of signal transduction |
| 0.534 | GO:0043085 | L | BP | positive regulation of catalytic activity |
| 0.519 | GO:0051246 | L | BP | regulation of protein metabolic process |
| 0.515 | GO:0010033 | L | BP | response to organic substance |
| 0.505 | GO:0007049 | L | BP | cell cycle |

**Blast**

| **BLAST Matched UniProt ID & name** | | | **Identity** | **E Value** | **Score** |
| --- | --- | --- | --- | --- | --- |
| P96707 | YDGI_BACSU | Putative NAD | 38.776 | 1.10E-43 | 151 |
| P81102 | YODC_BACSU | Putative NAD | 35.602 | 7.21E-30 | 115 |
| Q55233 | DRGA_SYNY3 | Protein DrgA | 25.654 | 1.37E-14 | 72.8 |
| Q8CN23 | Y2073_STAES | Putative NAD | 25.455 | 1.46E-12 | 67 |
| Q5HLA1 | Y2086_STAEQ | Putative NAD | 25.359 | 4.81E-12 | 65.9 |
| P15888 | NFSB_SALTY | Oxygen-insensitive NAD | 27.919 | 8.79E-11 | 62 |
| Q5XCB9 | Y809_STRP6 | Putative NAD | 27.835 | 2.71E-10 | 60.5 |
| P46072 | FRA1_ALIFS | Major NAD | 24.731 | 2.09E-08 | 55.8 |
| Q4L920 | Y546_STAHJ | Putative NAD | 23.223 | 6.33E-08 | 54.3 |
| Q4A087 | Y379_STAS1 | Putative NAD | 26.066 | 6.88E-08 | 54.3 |
| Q5HD30 | Y2534_STAAC | Putative NAD | 23.902 | 1.63E-07 | 53.1 |
| Q6G6F2 | Y2409_STAAS | Putative NAD | 23.415 | 5.82E-07 | 51.6 |
| O34475 | YFKO_BACSU | Putative NAD | 31.579 | 1.12E-06 | 50.8 |
| O28017 | Y2267_ARCFU | Putative NADH dehydrogenase/NAD | 33.696 | 1.51E-06 | 49.7 |
| B3Q6V6 | Y4764_RHOPT | Putative NADH dehydrogenase/NAD | 28.8 | 3.32E-05 | 46.6 |
| B4SHM0 | Y358_STRM5 | Putative NADH dehydrogenase/NAD | 28.472 | 3.78E-04 | 43.5 |
| Q60049 | NOX_THET8 | NADH dehydrogenase | 28.723 | 7.12E-04 | 42.7 |
| B2FKV6 | Y482_STRMK | Putative NADH dehydrogenase/NAD | 27.083 | 3.00E-03 | 40.8 |
| Q5REW1 | IYD1_PONAB | Iodotyrosine dehalogenase 1 | 30.769 | 9.00E-03 | 39.7 |
| Q6PHW0 | IYD1_HUMAN | Iodotyrosine dehalogenase 1 | 31.746 | 1.70E-02 | 38.9 |
| B0T152 | Y018_CAUSK | Putative NADH dehydrogenase/NAD | 24.8 | 4.10E-02 | 37.4 |

**Protein name: FadE34**

**Predicted results:**

**SVMProt**

| **Protein Family Name** | **GO Category** | **SVM** | **PNN** | **KNN** |
| --- | --- | --- | --- | --- |
| **Molecular Function** | | | | |
| EC3.5 Hydrolases - Acting on Carbon-Nitrogen Bonds, other than Peptide Bonds | - | 80.4 | - | Y |
| All lipid-binding proteins | GO:0008289 lipid binding | 99 | - | - |
| Zinc-binding | GO:0008270 zinc binding | 97 | - | - |
| Manganese-binding | GO:0030145 manganese binding | 96.4 | ? | ? |
| All DNA-binding | GO:0003677 DNA binding | 92.1 | ? | ? |
| EC6.3 Ligases - Forming Carbon-Nitrogen Bonds | - | 68.5 | - | Y |
| Magnesium-binding | GO:0000287 magnesium binding | 58.6 | ? | ? |
| **Biological Process** | | | | |
| DNA replication | GO:0006260 DNA replication | 85.4 | ? | ? |
| DNA repair | GO:0006281 DNA repair | 58.6 | ? | ? |

**FFPred**

| **Score** | **GO term** | **RL** | **Domain** | **Description** |
| --- | --- | --- | --- | --- |
| 0.957 | GO:0003824 | H | MF | catalytic activity |
| 0.878 | GO:0006082 | H | BP | organic acid metabolic process |
| 0.865 | GO:0000166 | H | MF | nucleotide binding |
| 0.845 | GO:0032549 | H | MF | ribonucleoside binding |
| 0.84 | GO:0017076 | H | MF | purine nucleotide binding |
| 0.831 | GO:0001883 | H | MF | purine nucleoside binding |
| 0.805 | GO:0044281 | H | BP | small molecule metabolic process |
| 0.799 | GO:0055114 | H | BP | oxidation-reduction process |
| 0.793 | GO:0016817 | H | MF | hydrolase activity, acting on acid anhydrides |
| 0.774 | GO:0001882 | H | MF | nucleoside binding |
| 0.766 | GO:0035639 | H | MF | purine ribonucleoside triphosphate binding |
| 0.746 | GO:0032787 | H | BP | monocarboxylic acid metabolic process |
| 0.736 | GO:0016740 | H | MF | transferase activity |
| 0.726 | GO:0006810 | H | BP | transport |
| 0.719 | GO:0070062 | H | CC | extracellular vesicular exosome |
| 0.696 | GO:0005524 | H | MF | ATP binding |
| 0.694 | GO:0005576 | H | CC | extracellular region |
| 0.679 | GO:0016818 | H | MF | hydrolase activity, acting on acid anhydrides, in phosphorus-containing anhydrides |
| 0.679 | GO:0016462 | H | MF | pyrophosphatase activity |
| 0.679 | GO:0016491 | H | MF | oxidoreductase activity |
| 0.654 | GO:0017111 | H | MF | nucleoside-triphosphatase activity |
| 0.644 | GO:0031982 | H | CC | vesicle |
| 0.64 | GO:0019752 | H | BP | carboxylic acid metabolic process |
| 0.631 | GO:0009056 | H | BP | catabolic process |
| 0.579 | GO:0005739 | H | CC | mitochondrion |
| 0.576 | GO:0030554 | H | MF | adenyl nucleotide binding |
| 0.554 | GO:0016020 | H | CC | membrane |
| 0.54 | GO:0009116 | H | BP | nucleoside metabolic process |
| 0.524 | GO:0051020 | H | MF | GTPase binding |
| 0.521 | GO:0006631 | H | BP | fatty acid metabolic process |
| 0.516 | GO:0031988 | H | CC | membrane-bounded vesicle |
| 0.96 | GO:0005737 | L | CC | cytoplasm |
| 0.909 | GO:0097159 | L | MF | organic cyclic compound binding |
| 0.906 | GO:0036094 | L | MF | small molecule binding |
| 0.905 | GO:0044237 | L | BP | cellular metabolic process |
| 0.891 | GO:0008152 | L | BP | metabolic process |
| 0.889 | GO:0006807 | L | BP | nitrogen compound metabolic process |
| 0.861 | GO:0050896 | L | BP | response to stimulus |
| 0.835 | GO:0009058 | L | BP | biosynthetic process |
| 0.827 | GO:0051716 | L | BP | cellular response to stimulus |
| 0.825 | GO:0046872 | L | MF | metal ion binding |
| 0.817 | GO:0005634 | L | CC | nucleus |
| 0.796 | GO:0043169 | L | MF | cation binding |
| 0.795 | GO:0034641 | L | BP | cellular nitrogen compound metabolic process |
| 0.793 | GO:0043229 | L | CC | intracellular organelle |
| 0.778 | GO:0046483 | L | BP | heterocycle metabolic process |
| 0.772 | GO:0006725 | L | BP | cellular aromatic compound metabolic process |
| 0.767 | GO:0043231 | L | CC | intracellular membrane-bounded organelle |
| 0.761 | GO:0005829 | L | CC | cytosol |
| 0.734 | GO:0019538 | L | BP | protein metabolic process |
| 0.732 | GO:0031981 | L | CC | nuclear lumen |
| 0.73 | GO:0032502 | L | BP | developmental process |
| 0.713 | GO:0016787 | L | MF | hydrolase activity |
| 0.699 | GO:0032991 | L | CC | macromolecular complex |
| 0.699 | GO:0006996 | L | BP | organelle organization |
| 0.663 | GO:0006139 | L | BP | nucleobase-containing compound metabolic process |
| 0.655 | GO:0043234 | L | CC | protein complex |
| 0.654 | GO:0005654 | L | CC | nucleoplasm |
| 0.649 | GO:0010467 | L | BP | gene expression |
| 0.644 | GO:0009966 | L | BP | regulation of signal transduction |
| 0.606 | GO:0048856 | L | BP | anatomical structure development |
| 0.601 | GO:0016070 | L | BP | RNA metabolic process |
| 0.592 | GO:0044267 | L | BP | cellular protein metabolic process |
| 0.579 | GO:0007275 | L | BP | multicellular organismal development |
| 0.568 | GO:0009893 | L | BP | positive regulation of metabolic process |
| 0.567 | GO:0007154 | L | BP | cell communication |
| 0.552 | GO:0005102 | L | MF | receptor binding |
| 0.534 | GO:0006464 | L | BP | cellular protein modification process |
| 0.505 | GO:0051246 | L | BP | regulation of protein metabolic process |

**Blast**

| **BLAST Matched UniProt ID & name** | | | **Identity** | **E Value** | **Score** |
| --- | --- | --- | --- | --- | --- |
| Q2LQP0 | CHCOA_SYNAS | Cyclohexane-1-carbonyl-CoA dehydrogenase | 34.314 | 2.11E-21 | 100 |
| Q9KJE8 | BBSG_THAAR | (R)-benzylsuccinyl-CoA dehydrogenase | 35.503 | 1.02E-19 | 95.5 |
| Q9FS88 | MBCD_SOLTU | 2-methylacyl-CoA dehydrogenase, mitochondrial | 33.333 | 2.01E-18 | 91.7 |
| Q9FS88 | MBCD_SOLTU | 2-methylacyl-CoA dehydrogenase, mitochondrial | 31.746 | 5.49E-05 | 49.7 |
| Q9FS87 | IVD_SOLTU | Isovaleryl-CoA dehydrogenase, mitochondrial | 32.609 | 1.07E-17 | 89.4 |
| Q9FS87 | IVD_SOLTU | Isovaleryl-CoA dehydrogenase, mitochondrial | 35.294 | 9.78E-07 | 55.1 |
| Q75IM9 | IVD_ORYSJ | Isovaleryl-CoA dehydrogenase, mitochondrial | 33.333 | 1.26E-17 | 89 |
| Q75IM9 | IVD_ORYSJ | Isovaleryl-CoA dehydrogenase, mitochondrial | 23.592 | 4.06E-06 | 53.1 |
| P41367 | ACADM_PIG | Medium-chain specific acyl-CoA dehydrogenase, mitochondrial | 31.336 | 7.37E-17 | 87 |
| Q5RAS0 | ACADS_PONAB | Short-chain specific acyl-CoA dehydrogenase, mitochondrial | 29.825 | 7.93E-17 | 86.7 |
| P16219 | ACADS_HUMAN | Short-chain specific acyl-CoA dehydrogenase, mitochondrial | 29.825 | 2.43E-16 | 85.1 |
| Q9SWG0 | IVD_ARATH | Isovaleryl-CoA dehydrogenase, mitochondrial | 29.67 | 5.76E-16 | 84 |
| Q9SWG0 | IVD_ARATH | Isovaleryl-CoA dehydrogenase, mitochondrial | 32.558 | 8.66E-05 | 48.9 |
| P63428 | ACDP_MYCBO | Probable acyl-CoA dehydrogenase fadE25 | 31.602 | 6.45E-16 | 83.6 |
| P63428 | ACDP_MYCBO | Probable acyl-CoA dehydrogenase fadE25 | 33.613 | 1.17E-09 | 64.3 |
| P11310 | ACADM_HUMAN | Medium-chain specific acyl-CoA dehydrogenase, mitochondrial | 30.651 | 8.42E-16 | 83.6 |
| Q8HXY8 | ACADM_MACFA | Medium-chain specific acyl-CoA dehydrogenase, mitochondrial | 30.268 | 9.38E-16 | 83.6 |
| C3UVB0 | ACD_DESML | Glutaryl-CoA dehydrogenase | 27.757 | 1.00E-15 | 83.2 |
| C3UVB0 | ACD_DESML | Glutaryl-CoA dehydrogenase | 26.42 | 3.24E-09 | 63.2 |
| A5A6I0 | ACADM_PANTR | Medium-chain specific acyl-CoA dehydrogenase, mitochondrial | 30.651 | 1.30E-15 | 83.2 |
| Q07417 | ACADS_MOUSE | Short-chain specific acyl-CoA dehydrogenase, mitochondrial | 26.962 | 3.07E-15 | 82 |
| P15651 | ACADS_RAT | Short-chain specific acyl-CoA dehydrogenase, mitochondrial | 26.962 | 4.77E-15 | 81.3 |
| P45954 | ACDSB_HUMAN | Short/branched chain specific acyl-CoA dehydrogenase, mitochondrial | 32.653 | 6.70E-15 | 80.9 |
| Q5RF40 | ACDSB_PONAB | Short/branched chain specific acyl-CoA dehydrogenase, mitochondrial | 32.653 | 9.40E-15 | 80.5 |
| P51174 | ACADL_MOUSE | Long-chain specific acyl-CoA dehydrogenase, mitochondrial | 34.194 | 1.21E-14 | 80.1 |
| Q3ZBF6 | ACADS_BOVIN | Short-chain specific acyl-CoA dehydrogenase, mitochondrial | 27.986 | 1.61E-14 | 79.7 |
| Q3ZBF6 | ACADS_BOVIN | Short-chain specific acyl-CoA dehydrogenase, mitochondrial | 34.211 | 1.17E-05 | 52 |
| P15650 | ACADL_RAT | Long-chain specific acyl-CoA dehydrogenase, mitochondrial | 32.716 | 3.63E-14 | 78.6 |
| P46703 | ACDP_MYCLE | Probable acyl-CoA dehydrogenase fadE25 | 30.736 | 3.88E-14 | 78.2 |
| P46703 | ACDP_MYCLE | Probable acyl-CoA dehydrogenase fadE25 | 30.252 | 5.79E-07 | 55.8 |
| P45952 | ACADM_MOUSE | Medium-chain specific acyl-CoA dehydrogenase, mitochondrial | 29.126 | 7.63E-14 | 77.8 |
| Q3SZB4 | ACADM_BOVIN | Medium-chain specific acyl-CoA dehydrogenase, mitochondrial | 32.124 | 7.70E-14 | 77.8 |
| P70584 | ACDSB_RAT | Short/branched chain specific acyl-CoA dehydrogenase, mitochondrial | 27.599 | 1.17E-13 | 77 |
| P28330 | ACADL_HUMAN | Long-chain specific acyl-CoA dehydrogenase, mitochondrial | 31.818 | 3.70E-13 | 75.5 |
| P79273 | ACADS_PIG | Short-chain specific acyl-CoA dehydrogenase, mitochondrial | 26.871 | 3.96E-13 | 75.5 |
| Q9VSA3 | ACADM_DROME | Probable medium-chain specific acyl-CoA dehydrogenase, mitochondrial | 30.573 | 4.12E-13 | 75.5 |
| P08503 | ACADM_RAT | Medium-chain specific acyl-CoA dehydrogenase, mitochondrial | 28.077 | 5.93E-13 | 74.7 |
| Q9DBL1 | ACDSB_MOUSE | Short/branched chain specific acyl-CoA dehydrogenase, mitochondrial | 31.544 | 6.61E-13 | 74.7 |
| P45857 | ACDB_BACSU | Acyl-CoA dehydrogenase | 24.731 | 1.08E-12 | 73.9 |
| P79274 | ACADL_PIG | Long-chain specific acyl-CoA dehydrogenase, mitochondrial | 32.484 | 2.01E-12 | 73.2 |
| Q5EAD4 | ACDSB_BOVIN | Short/branched chain specific acyl-CoA dehydrogenase, mitochondrial | 27.545 | 2.66E-12 | 72.8 |
| Q8K370 | ACD10_MOUSE | Acyl-CoA dehydrogenase family member 10 | 27.679 | 1.88E-11 | 71.2 |
| P34275 | IVD_CAEEL | Probable acyl-CoA dehydrogenase 6 | 22.485 | 2.33E-11 | 69.7 |
| P45867 | ACDA_BACSU | Acyl-CoA dehydrogenase | 25.067 | 3.49E-11 | 68.9 |
| Q60HI0 | ACADL_MACFA | Long-chain specific acyl-CoA dehydrogenase, mitochondrial | 31.169 | 4.20E-11 | 68.9 |
| P9WQG2 | ACDC_MYCTO | Acyl-CoA dehydrogenase fadE12 | 28.431 | 9.18E-11 | 67.8 |
| Q96329 | ACOX4_ARATH | Acyl-coenzyme A oxidase 4, peroxisomal | 28 | 9.67E-11 | 68.2 |
| Q7U0Y2 | ACDC_MYCBO | Acyl-CoA dehydrogenase fadE12 | 28.431 | 9.68E-11 | 67.8 |
| Q2LQN9 | CH1CO_SYNAS | Cyclohex-1-ene-1-carbonyl-CoA dehydrogenase | 26.906 | 2.82E-10 | 66.6 |
| Q2LQN9 | CH1CO_SYNAS | Cyclohex-1-ene-1-carbonyl-CoA dehydrogenase | 27.692 | 3.66E-07 | 56.6 |
| P52042 | ACDS_CLOAB | Acyl-CoA dehydrogenase, short-chain specific | 24.67 | 4.53E-10 | 65.5 |
| P52042 | ACDS_CLOAB | Acyl-CoA dehydrogenase, short-chain specific | 25.767 | 3.00E-03 | 44.3 |
| Q54IM8 | ACAD8_DICDI | Isobutyryl-CoA dehydrogenase, mitochondrial | 27.957 | 7.06E-10 | 65.1 |
| Q8RWZ3 | IBR3_ARATH | Probable acyl-CoA dehydrogenase IBR3 | 31.373 | 8.47E-10 | 65.9 |
| Q0NXR6 | ACAD8_BOVIN | Isobutyryl-CoA dehydrogenase, mitochondrial | 24.39 | 1.05E-09 | 64.7 |
| Q9UKU7 | ACAD8_HUMAN | Isobutyryl-CoA dehydrogenase, mitochondrial | 24.407 | 3.04E-09 | 63.2 |
| Q9D7B6 | ACAD8_MOUSE | Isobutyryl-CoA dehydrogenase, mitochondrial | 24.39 | 5.93E-09 | 62.4 |
| P63430 | Y897_MYCBO | Probable acyl-CoA dehydrogenase FadE10 | 35.669 | 6.50E-09 | 62.8 |
| Q6JQN1 | ACD10_HUMAN | Acyl-CoA dehydrogenase family member 10 | 26.364 | 7.45E-09 | 62.8 |
| Q80XL6 | ACD11_MOUSE | Acyl-CoA dehydrogenase family member 11 | 23.345 | 3.05E-08 | 60.8 |
| Q06319 | ACDS_MEGEL | Acyl-CoA dehydrogenase, short-chain specific | 32.743 | 3.15E-08 | 59.7 |
| O34421 | ACDC_BACSU | Probable acyl-CoA dehydrogenase YngJ | 36.842 | 1.11E-07 | 58.2 |
| O34421 | ACDC_BACSU | Probable acyl-CoA dehydrogenase YngJ | 27.922 | 1.80E-07 | 57.4 |
| Q54RR5 | ACDSB_DICDI | Probable short/branched chain specific acyl-CoA dehydrogenase | 31.532 | 3.59E-07 | 56.6 |
| Q2KHZ9 | GCDH_BOVIN | Glutaryl-CoA dehydrogenase, mitochondrial | 25.564 | 7.10E-06 | 52.4 |
| Q92947 | GCDH_HUMAN | Glutaryl-CoA dehydrogenase, mitochondrial | 25.581 | 3.08E-05 | 50.4 |
| Q60759 | GCDH_MOUSE | Glutaryl-CoA dehydrogenase, mitochondrial | 24.812 | 3.90E-05 | 50.1 |
| Q8HXX8 | GCDH_MACFA | Glutaryl-CoA dehydrogenase, mitochondrial | 26.623 | 5.62E-05 | 49.7 |
| P81140 | GCDH_PIG | Glutaryl-CoA dehydrogenase, mitochondrial | 26.154 | 1.48E-04 | 48.1 |
| P50544 | ACADV_MOUSE | Very long-chain specific acyl-CoA dehydrogenase, mitochondrial | 29.73 | 3.00E-03 | 44.7 |

**Protein name: FadE34**

**Predicted results:**

**SVMProt**

| **Protein Family Name** | **GO Category** | **SVM** | **PNN** | **KNN** |
| --- | --- | --- | --- | --- |
| **Molecular Function** | | | | |
| EC2.3 Transferases - Acyltransferases | - | 94.2 | - | Y |
| EC1.1 Oxidoreductases - Acting on the CH-OH group of donors | - | 83.9 | - | Y |
| All lipid-binding proteins | GO:0008289 lipid binding | 97.5 | - | - |
| Zinc-binding | GO:0008270 zinc binding | 95.7 | - | - |
| EC2.7 Transferases - Transferring Phosphorus-Containing Groups | - | 68.5 | Y | - |
| EC3.5 Hydrolases - Acting on Carbon-Nitrogen Bonds, other than Peptide Bonds | - | 65.4 | - | Y |
| EC6.3 Ligases - Forming Carbon-Nitrogen Bonds | - | 62.2 | - | - |
| **Broadly Defined Function** | | | | |
| Photosystem I | GO:0009522 photosystem I | 58.6 | ? | ? |

**FFPred**

| **Score** | **GO term** | **RL** | **Domain** | **Description** |
| --- | --- | --- | --- | --- |
| 0.912 | GO:0003824 | H | MF | catalytic activity |
| 0.898 | GO:0032549 | H | MF | ribonucleoside binding |
| 0.879 | GO:0017076 | H | MF | purine nucleotide binding |
| 0.861 | GO:0000166 | H | MF | nucleotide binding |
| 0.848 | GO:0001882 | H | MF | nucleoside binding |
| 0.838 | GO:0001883 | H | MF | purine nucleoside binding |
| 0.829 | GO:0035639 | H | MF | purine ribonucleoside triphosphate binding |
| 0.801 | GO:0016817 | H | MF | hydrolase activity, acting on acid anhydrides |
| 0.734 | GO:0006082 | H | BP | organic acid metabolic process |
| 0.725 | GO:0044281 | H | BP | small molecule metabolic process |
| 0.7 | GO:0005524 | H | MF | ATP binding |
| 0.687 | GO:0015031 | H | BP | protein transport |
| 0.686 | GO:0006810 | H | BP | transport |
| 0.686 | GO:0016818 | H | MF | hydrolase activity, acting on acid anhydrides, in phosphorus-containing anhydrides |
| 0.677 | GO:0005739 | H | CC | mitochondrion |
| 0.663 | GO:0051020 | H | MF | GTPase binding |
| 0.662 | GO:0045184 | H | BP | establishment of protein localization |
| 0.643 | GO:0031267 | H | MF | small GTPase binding |
| 0.635 | GO:0016462 | H | MF | pyrophosphatase activity |
| 0.629 | GO:0009056 | H | BP | catabolic process |
| 0.627 | GO:0017111 | H | MF | nucleoside-triphosphatase activity |
| 0.622 | GO:0032787 | H | BP | monocarboxylic acid metabolic process |
| 0.588 | GO:0030554 | H | MF | adenyl nucleotide binding |
| 0.577 | GO:0070062 | H | CC | extracellular vesicular exosome |
| 0.562 | GO:0019222 | H | BP | regulation of metabolic process |
| 0.556 | GO:0017016 | H | MF | Ras GTPase binding |
| 0.555 | GO:0031982 | H | CC | vesicle |
| 0.535 | GO:0016020 | H | CC | membrane |
| 0.518 | GO:0019752 | H | BP | carboxylic acid metabolic process |
| 0.96 | GO:0005737 | L | CC | cytoplasm |
| 0.908 | GO:0044237 | L | BP | cellular metabolic process |
| 0.878 | GO:0036094 | L | MF | small molecule binding |
| 0.849 | GO:0050896 | L | BP | response to stimulus |
| 0.836 | GO:0097159 | L | MF | organic cyclic compound binding |
| 0.822 | GO:0005634 | L | CC | nucleus |
| 0.82 | GO:0051716 | L | BP | cellular response to stimulus |
| 0.803 | GO:0043229 | L | CC | intracellular organelle |
| 0.794 | GO:0034641 | L | BP | cellular nitrogen compound metabolic process |
| 0.79 | GO:0006807 | L | BP | nitrogen compound metabolic process |
| 0.782 | GO:0046483 | L | BP | heterocycle metabolic process |
| 0.779 | GO:0008152 | L | BP | metabolic process |
| 0.778 | GO:0006725 | L | BP | cellular aromatic compound metabolic process |
| 0.77 | GO:0005829 | L | CC | cytosol |
| 0.745 | GO:0043231 | L | CC | intracellular membrane-bounded organelle |
| 0.741 | GO:0031981 | L | CC | nuclear lumen |
| 0.715 | GO:0032502 | L | BP | developmental process |
| 0.71 | GO:0006996 | L | BP | organelle organization |
| 0.702 | GO:0009966 | L | BP | regulation of signal transduction |
| 0.693 | GO:0019538 | L | BP | protein metabolic process |
| 0.689 | GO:0043234 | L | CC | protein complex |
| 0.682 | GO:0007154 | L | BP | cell communication |
| 0.674 | GO:0044267 | L | BP | cellular protein metabolic process |
| 0.666 | GO:0005654 | L | CC | nucleoplasm |
| 0.666 | GO:0016787 | L | MF | hydrolase activity |
| 0.663 | GO:0032991 | L | CC | macromolecular complex |
| 0.654 | GO:0010467 | L | BP | gene expression |
| 0.634 | GO:0009058 | L | BP | biosynthetic process |
| 0.614 | GO:0016070 | L | BP | RNA metabolic process |
| 0.611 | GO:0006139 | L | BP | nucleobase-containing compound metabolic process |
| 0.606 | GO:0023052 | L | BP | signaling |
| 0.591 | GO:0009893 | L | BP | positive regulation of metabolic process |
| 0.591 | GO:0046872 | L | MF | metal ion binding |
| 0.576 | GO:0007275 | L | BP | multicellular organismal development |
| 0.538 | GO:0048856 | L | BP | anatomical structure development |
| 0.501 | GO:0043169 | L | MF | cation binding |

**Blast**

| **BLAST Matched UniProt ID & name** | | | **Identity** | **E Value** | **Score** |
| --- | --- | --- | --- | --- | --- |
| P45867 | ACDA_BACSU | Acyl-CoA dehydrogenase | 26.786 | 1.62E-25 | 113 |
| P45867 | ACDA_BACSU | Acyl-CoA dehydrogenase | 26.23 | 3.27E-09 | 63.2 |
| P45857 | ACDB_BACSU | Acyl-CoA dehydrogenase | 27.82 | 6.55E-25 | 111 |
| Q5RAS0 | ACADS_PONAB | Short-chain specific acyl-CoA dehydrogenase, mitochondrial | 28.819 | 1.99E-18 | 91.7 |
| Q2LQN9 | CH1CO_SYNAS | Cyclohex-1-ene-1-carbonyl-CoA dehydrogenase | 25.5 | 6.85E-18 | 90.1 |
| P16219 | ACADS_HUMAN | Short-chain specific acyl-CoA dehydrogenase, mitochondrial | 28.819 | 7.16E-18 | 90.1 |
| P79273 | ACADS_PIG | Short-chain specific acyl-CoA dehydrogenase, mitochondrial | 27.506 | 1.05E-17 | 89.4 |
| O34421 | ACDC_BACSU | Probable acyl-CoA dehydrogenase YngJ | 25.316 | 2.60E-17 | 87.8 |
| O34421 | ACDC_BACSU | Probable acyl-CoA dehydrogenase YngJ | 32.895 | 1.35E-08 | 61.2 |
| Q9FS88 | MBCD_SOLTU | 2-methylacyl-CoA dehydrogenase, mitochondrial | 30.882 | 2.40E-16 | 85.5 |
| Q9FS88 | MBCD_SOLTU | 2-methylacyl-CoA dehydrogenase, mitochondrial | 22.959 | 1.42E-09 | 64.3 |
| P15651 | ACADS_RAT | Short-chain specific acyl-CoA dehydrogenase, mitochondrial | 27.526 | 3.57E-16 | 84.7 |
| Q07417 | ACADS_MOUSE | Short-chain specific acyl-CoA dehydrogenase, mitochondrial | 27.397 | 5.05E-16 | 84.3 |
| P45954 | ACDSB_HUMAN | Short/branched chain specific acyl-CoA dehydrogenase, mitochondrial | 28.402 | 5.35E-16 | 84.7 |
| P45954 | ACDSB_HUMAN | Short/branched chain specific acyl-CoA dehydrogenase, mitochondrial | 22.564 | 3.17E-14 | 79 |
| P28330 | ACADL_HUMAN | Long-chain specific acyl-CoA dehydrogenase, mitochondrial | 33.333 | 5.57E-16 | 84.3 |
| P28330 | ACADL_HUMAN | Long-chain specific acyl-CoA dehydrogenase, mitochondrial | 20.981 | 5.83E-08 | 59.3 |
| Q5RF40 | ACDSB_PONAB | Short/branched chain specific acyl-CoA dehydrogenase, mitochondrial | 28.402 | 6.35E-16 | 84.3 |
| Q5RF40 | ACDSB_PONAB | Short/branched chain specific acyl-CoA dehydrogenase, mitochondrial | 22.564 | 2.11E-14 | 79.7 |
| P12007 | IVD_RAT | Isovaleryl-CoA dehydrogenase, mitochondrial | 24.311 | 7.10E-16 | 84 |
| P46703 | ACDP_MYCLE | Probable acyl-CoA dehydrogenase fadE25 | 31.621 | 9.32E-16 | 83.2 |
| P34275 | IVD_CAEEL | Probable acyl-CoA dehydrogenase 6 | 27.941 | 1.14E-15 | 83.2 |
| Q3ZBF6 | ACADS_BOVIN | Short-chain specific acyl-CoA dehydrogenase, mitochondrial | 27.604 | 1.17E-15 | 83.2 |
| Q9KJE8 | BBSG_THAAR | (R)-benzylsuccinyl-CoA dehydrogenase | 34.014 | 1.53E-15 | 82.8 |
| P63428 | ACDP_MYCBO | Probable acyl-CoA dehydrogenase fadE25 | 30.159 | 4.28E-15 | 81.3 |
| Q75IM9 | IVD_ORYSJ | Isovaleryl-CoA dehydrogenase, mitochondrial | 30.288 | 5.98E-15 | 81.3 |
| Q75IM9 | IVD_ORYSJ | Isovaleryl-CoA dehydrogenase, mitochondrial | 23.018 | 4.91E-11 | 68.9 |
| Q9JHI5 | IVD_MOUSE | Isovaleryl-CoA dehydrogenase, mitochondrial | 24.81 | 6.14E-15 | 81.3 |
| C3UVB0 | ACD_DESML | Glutaryl-CoA dehydrogenase | 33.333 | 6.62E-15 | 80.9 |
| C3UVB0 | ACD_DESML | Glutaryl-CoA dehydrogenase | 29.252 | 1.00E-03 | 45.4 |
| Q5EAD4 | ACDSB_BOVIN | Short/branched chain specific acyl-CoA dehydrogenase, mitochondrial | 22.475 | 6.62E-15 | 81.3 |
| Q5EAD4 | ACDSB_BOVIN | Short/branched chain specific acyl-CoA dehydrogenase, mitochondrial | 25.656 | 7.81E-12 | 71.6 |
| P79274 | ACADL_PIG | Long-chain specific acyl-CoA dehydrogenase, mitochondrial | 34.416 | 6.66E-15 | 81.3 |
| P79274 | ACADL_PIG | Long-chain specific acyl-CoA dehydrogenase, mitochondrial | 22.826 | 1.45E-08 | 61.2 |
| Q9SWG0 | IVD_ARATH | Isovaleryl-CoA dehydrogenase, mitochondrial | 30.864 | 7.36E-15 | 80.9 |
| Q9SWG0 | IVD_ARATH | Isovaleryl-CoA dehydrogenase, mitochondrial | 27.815 | 5.31E-06 | 53.1 |
| Q5RBD5 | IVD_PONAB | Isovaleryl-CoA dehydrogenase, mitochondrial | 23.559 | 8.19E-15 | 80.9 |
| Q9FS87 | IVD_SOLTU | Isovaleryl-CoA dehydrogenase, mitochondrial | 31.481 | 8.38E-15 | 80.5 |
| Q9FS87 | IVD_SOLTU | Isovaleryl-CoA dehydrogenase, mitochondrial | 22.564 | 2.21E-07 | 57.4 |
| Q8HXY8 | ACADM_MACFA | Medium-chain specific acyl-CoA dehydrogenase, mitochondrial | 29.612 | 9.67E-15 | 80.5 |
| Q8HXY8 | ACADM_MACFA | Medium-chain specific acyl-CoA dehydrogenase, mitochondrial | 22.277 | 1.17E-11 | 70.9 |
| P26440 | IVD_HUMAN | Isovaleryl-CoA dehydrogenase, mitochondrial | 22.556 | 1.81E-14 | 79.7 |
| P51174 | ACADL_MOUSE | Long-chain specific acyl-CoA dehydrogenase, mitochondrial | 31.818 | 2.08E-14 | 79.7 |
| Q3SZI8 | IVD_BOVIN | Isovaleryl-CoA dehydrogenase, mitochondrial | 24.304 | 2.41E-14 | 79.3 |
| B7M0D6 | CAIA_ECO8A | Crotonobetainyl-CoA dehydrogenase | 27.606 | 6.26E-14 | 77.8 |
| B7M0D6 | CAIA_ECO8A | Crotonobetainyl-CoA dehydrogenase | 26.667 | 1.12E-08 | 61.2 |
| P41367 | ACADM_PIG | Medium-chain specific acyl-CoA dehydrogenase, mitochondrial | 29.353 | 6.47E-14 | 78.2 |
| Q60HI0 | ACADL_MACFA | Long-chain specific acyl-CoA dehydrogenase, mitochondrial | 32.68 | 6.62E-14 | 78.2 |
| Q60HI0 | ACADL_MACFA | Long-chain specific acyl-CoA dehydrogenase, mitochondrial | 22.703 | 5.21E-08 | 59.3 |
| P11310 | ACADM_HUMAN | Medium-chain specific acyl-CoA dehydrogenase, mitochondrial | 30.412 | 7.08E-14 | 77.8 |
| P11310 | ACADM_HUMAN | Medium-chain specific acyl-CoA dehydrogenase, mitochondrial | 23.232 | 1.80E-12 | 73.6 |
| P15650 | ACADL_RAT | Long-chain specific acyl-CoA dehydrogenase, mitochondrial | 31.169 | 8.55E-14 | 77.8 |
| A5A6I0 | ACADM_PANTR | Medium-chain specific acyl-CoA dehydrogenase, mitochondrial | 30.412 | 1.03E-13 | 77.4 |
| A5A6I0 | ACADM_PANTR | Medium-chain specific acyl-CoA dehydrogenase, mitochondrial | 23.232 | 2.51E-12 | 73.2 |
| A8WP91 | ACAD2_CAEBR | Probable medium-chain specific acyl-CoA dehydrogenase 2, mitochondrial | 26.538 | 1.08E-13 | 77 |
| P60584 | CAIA_ECOLI | Crotonobetainyl-CoA dehydrogenase | 27.324 | 1.29E-13 | 76.6 |
| P60584 | CAIA_ECOLI | Crotonobetainyl-CoA dehydrogenase | 26.667 | 1.08E-08 | 61.2 |
| P0A9V0 | YDIO_ECO57 | Probable acyl-CoA dehydrogenase YdiO | 24.339 | 1.35E-13 | 76.6 |
| P0A9V0 | YDIO_ECO57 | Probable acyl-CoA dehydrogenase YdiO | 29.5 | 8.43E-12 | 71.2 |
| Q0TLV0 | CAIA_ECOL5 | Crotonobetainyl-CoA dehydrogenase | 27.324 | 1.65E-13 | 76.3 |
| Q0TLV0 | CAIA_ECOL5 | Crotonobetainyl-CoA dehydrogenase | 26.667 | 1.07E-08 | 61.2 |
| Q2LQP0 | CHCOA_SYNAS | Cyclohexane-1-carbonyl-CoA dehydrogenase | 34.416 | 1.94E-13 | 76.3 |
| Q9DBL1 | ACDSB_MOUSE | Short/branched chain specific acyl-CoA dehydrogenase, mitochondrial | 26.384 | 2.15E-13 | 76.3 |
| Q9DBL1 | ACDSB_MOUSE | Short/branched chain specific acyl-CoA dehydrogenase, mitochondrial | 22.739 | 5.12E-12 | 72 |
| Q0T8F5 | CAIA_SHIF8 | Crotonobetainyl-CoA dehydrogenase | 26.593 | 2.15E-13 | 75.9 |
| Q0T8F5 | CAIA_SHIF8 | Crotonobetainyl-CoA dehydrogenase | 26.667 | 1.06E-08 | 61.2 |
| P70584 | ACDSB_RAT | Short/branched chain specific acyl-CoA dehydrogenase, mitochondrial | 26.71 | 2.37E-13 | 76.3 |
| P70584 | ACDSB_RAT | Short/branched chain specific acyl-CoA dehydrogenase, mitochondrial | 21.851 | 5.14E-11 | 68.9 |
| Q8HXY7 | ACADV_MACFA | Very long-chain specific acyl-CoA dehydrogenase, mitochondrial | 25.879 | 6.20E-13 | 75.9 |
| Q9VSA3 | ACADM_DROME | Probable medium-chain specific acyl-CoA dehydrogenase, mitochondrial | 34.641 | 6.44E-13 | 74.7 |
| Q06319 | ACDS_MEGEL | Acyl-CoA dehydrogenase, short-chain specific | 33.333 | 6.74E-13 | 74.3 |
| P08503 | ACADM_RAT | Medium-chain specific acyl-CoA dehydrogenase, mitochondrial | 29.897 | 7.11E-13 | 74.7 |
| Q8GB20 | CAIA_PROSL | Crotonobetainyl-CoA dehydrogenase | 26 | 1.06E-12 | 73.9 |
| Q8GB20 | CAIA_PROSL | Crotonobetainyl-CoA dehydrogenase | 24.667 | 2.60E-07 | 57 |
| Q54RR5 | ACDSB_DICDI | Probable short/branched chain specific acyl-CoA dehydrogenase | 23.98 | 1.32E-12 | 73.9 |
| A9MQH5 | CAIA_SALAR | Crotonobetainyl-CoA dehydrogenase | 27.019 | 1.43E-12 | 73.6 |
| A9MQH5 | CAIA_SALAR | Crotonobetainyl-CoA dehydrogenase | 27.273 | 3.98E-08 | 59.7 |
| P49748 | ACADV_HUMAN | Very long-chain specific acyl-CoA dehydrogenase, mitochondrial | 25.278 | 1.70E-12 | 74.3 |
| Q3SZB4 | ACADM_BOVIN | Medium-chain specific acyl-CoA dehydrogenase, mitochondrial | 29.897 | 1.93E-12 | 73.6 |
| P45952 | ACADM_MOUSE | Medium-chain specific acyl-CoA dehydrogenase, mitochondrial | 29.381 | 3.48E-12 | 72.4 |
| Q8ZRX2 | CAIA_SALTY | Crotonobetainyl-CoA dehydrogenase | 27.042 | 3.94E-12 | 72 |
| Q8ZRX2 | CAIA_SALTY | Crotonobetainyl-CoA dehydrogenase | 26.667 | 3.00E-09 | 63.2 |
| A8ALR4 | CAIA_CITK8 | Crotonobetainyl-CoA dehydrogenase | 26.966 | 4.35E-12 | 72 |
| A8ALR4 | CAIA_CITK8 | Crotonobetainyl-CoA dehydrogenase | 26.667 | 5.96E-09 | 62 |
| A8XNF0 | ACAD1_CAEBR | Probable medium-chain specific acyl-CoA dehydrogenase 1, mitochondrial | 28.272 | 4.46E-12 | 72.4 |
| P52042 | ACDS_CLOAB | Acyl-CoA dehydrogenase, short-chain specific | 29.53 | 9.16E-12 | 70.9 |
| Q8Z9L2 | CAIA_SALTI | Crotonobetainyl-CoA dehydrogenase | 26.761 | 1.23E-11 | 70.5 |
| Q8Z9L2 | CAIA_SALTI | Crotonobetainyl-CoA dehydrogenase | 26.19 | 2.00E-09 | 63.5 |
| Q22347 | ACADM_CAEEL | Probable medium-chain specific acyl-CoA dehydrogenase 10, mitochondrial | 27.749 | 1.37E-11 | 70.9 |
| Q7U0Y2 | ACDC_MYCBO | Acyl-CoA dehydrogenase fadE12 | 26.108 | 1.52E-11 | 70.5 |
| Q7U0Y2 | ACDC_MYCBO | Acyl-CoA dehydrogenase fadE12 | 29.213 | 1.85E-10 | 67 |
| P9WQG2 | ACDC_MYCTO | Acyl-CoA dehydrogenase fadE12 | 26.355 | 1.63E-11 | 70.1 |
| P9WQG2 | ACDC_MYCTO | Acyl-CoA dehydrogenase fadE12 | 29.213 | 1.68E-10 | 67 |
| Q9UKU7 | ACAD8_HUMAN | Isobutyryl-CoA dehydrogenase, mitochondrial | 27.731 | 2.40E-11 | 70.1 |
| P48818 | ACADV_BOVIN | Very long-chain specific acyl-CoA dehydrogenase, mitochondrial | 25.469 | 4.44E-11 | 69.7 |
| Q9D7B6 | ACAD8_MOUSE | Isobutyryl-CoA dehydrogenase, mitochondrial | 25.135 | 7.67E-11 | 68.2 |
| Q54IM8 | ACAD8_DICDI | Isobutyryl-CoA dehydrogenase, mitochondrial | 27.807 | 1.03E-10 | 67.8 |
| P45953 | ACADV_RAT | Very long-chain specific acyl-CoA dehydrogenase, mitochondrial | 23.989 | 1.03E-10 | 68.6 |
| P50544 | ACADV_MOUSE | Very long-chain specific acyl-CoA dehydrogenase, mitochondrial | 23.81 | 1.12E-10 | 68.6 |
| Q96329 | ACOX4_ARATH | Acyl-coenzyme A oxidase 4, peroxisomal | 33.594 | 1.25E-10 | 67.8 |
| O32176 | FADE_BACSU | Probable acyl-CoA dehydrogenase | 19.551 | 1.02E-09 | 65.5 |
| Q9H845 | ACAD9_HUMAN | Acyl-CoA dehydrogenase family member 9, mitochondrial | 23.77 | 1.08E-08 | 62 |
| Q2KHZ9 | GCDH_BOVIN | Glutaryl-CoA dehydrogenase, mitochondrial | 28.283 | 8.88E-08 | 58.5 |
| Q92947 | GCDH_HUMAN | Glutaryl-CoA dehydrogenase, mitochondrial | 29.054 | 1.57E-07 | 57.8 |
| B3DMA2 | ACD11_RAT | Acyl-CoA dehydrogenase family member 11 | 31.034 | 8.56E-07 | 56.2 |
| Q8HXX8 | GCDH_MACFA | Glutaryl-CoA dehydrogenase, mitochondrial | 34.146 | 1.13E-06 | 55.1 |
| Q20772 | GCDH_CAEEL | Probable glutaryl-CoA dehydrogenase, mitochondrial | 29.787 | 1.93E-06 | 54.3 |
| Q60759 | GCDH_MOUSE | Glutaryl-CoA dehydrogenase, mitochondrial | 35.366 | 3.35E-06 | 53.5 |
| Q80XL6 | ACD11_MOUSE | Acyl-CoA dehydrogenase family member 11 | 29.231 | 3.58E-06 | 53.9 |
| Q54R47 | GCDH_DICDI | Glutaryl-CoA dehydrogenase, mitochondrial | 36.047 | 2.27E-05 | 50.8 |
| Q8Z937 | FADE_SALTI | Acyl-coenzyme A dehydrogenase | 25.368 | 2.00E-02 | 42 |
| Q8ZRJ7 | FADE_SALTY | Acyl-coenzyme A dehydrogenase | 25.368 | 2.30E-02 | 41.6 |

**Protein name: Ras3**

**Predicted results:**

**SVMProt**

| **Protein Family Name** | **GO Category** | **SVM** | **PNN** | **KNN** |
| --- | --- | --- | --- | --- |
| **Molecular Function** | | | | |
| Zinc-binding | GO:0008270 zinc binding | 92.1 | - | - |
| All lipid-binding proteins | GO:0008289 lipid binding | 89.3 | - | - |
| RNA-binding proteins | GO:0003723 RNA binding | 58.6 | ? | ? |
| Calcium-binding | - | 58.6 | - | - |
| Actin binding | GO:0003779 actin binding | 58.6 | ? | ? |
| Magnesium-binding | GO:0000287 magnesium binding | 58.6 | ? | ? |
| Metal-binding | GO:0046872 metal ion binding | 58.6 | - | - |
| EC2.1 Transferases - Transferring One-Carbon Groups | - | 58.6 | - | - |
| mRNA-binding proteins | GO:0003729 mRNA binding | 58.6 | - | - |
| Copper-binding | GO:0005507 copper binding | 58.6 | - | - |
| **Broadly Defined Function** | | | | |
| Outer membrane | GO:0009279 cell outer membrane | 58.6 | - | - |

**FFPred**

| **Score** | **GO term** | **RL** | **Domain** | **Description** |
| --- | --- | --- | --- | --- |
| 0.752 | GO:0000166 | H | MF | nucleotide binding |
| 0.734 | GO:0035639 | H | MF | purine ribonucleoside triphosphate binding |
| 0.729 | GO:0019222 | H | BP | regulation of metabolic process |
| 0.709 | GO:0006810 | H | BP | transport |
| 0.699 | GO:0035556 | H | BP | intracellular signal transduction |
| 0.698 | GO:0017076 | H | MF | purine nucleotide binding |
| 0.693 | GO:0001882 | H | MF | nucleoside binding |
| 0.682 | GO:0016310 | H | BP | phosphorylation |
| 0.677 | GO:0032549 | H | MF | ribonucleoside binding |
| 0.662 | GO:0003824 | H | MF | catalytic activity |
| 0.653 | GO:0016740 | H | MF | transferase activity |
| 0.618 | GO:0016301 | H | MF | kinase activity |
| 0.604 | GO:0001883 | H | MF | purine nucleoside binding |
| 0.592 | GO:0051641 | H | BP | cellular localization |
| 0.571 | GO:0005516 | H | MF | calmodulin binding |
| 0.566 | GO:0006796 | H | BP | phosphate-containing compound metabolic process |
| 0.564 | GO:0016192 | H | BP | vesicle-mediated transport |
| 0.545 | GO:0032561 | H | MF | guanyl ribonucleotide binding |
| 0.536 | GO:0005730 | H | CC | nucleolus |
| 0.535 | GO:0051171 | H | BP | regulation of nitrogen compound metabolic process |
| 0.534 | GO:0004674 | H | MF | protein serine/threonine kinase activity |
| 0.527 | GO:0016020 | H | CC | membrane |
| 0.512 | GO:0051649 | H | BP | establishment of localization in cell |
| 0.979 | GO:0043229 | L | CC | intracellular organelle |
| 0.938 | GO:0043231 | L | CC | intracellular membrane-bounded organelle |
| 0.92 | GO:0005737 | L | CC | cytoplasm |
| 0.916 | GO:0044237 | L | BP | cellular metabolic process |
| 0.914 | GO:0008152 | L | BP | metabolic process |
| 0.894 | GO:0097159 | L | MF | organic cyclic compound binding |
| 0.849 | GO:0019538 | L | BP | protein metabolic process |
| 0.848 | GO:0007165 | L | BP | signal transduction |
| 0.839 | GO:0023052 | L | BP | signaling |
| 0.828 | GO:0050896 | L | BP | response to stimulus |
| 0.823 | GO:0005634 | L | CC | nucleus |
| 0.812 | GO:0034641 | L | BP | cellular nitrogen compound metabolic process |
| 0.809 | GO:0051716 | L | BP | cellular response to stimulus |
| 0.807 | GO:0046483 | L | BP | heterocycle metabolic process |
| 0.805 | GO:0044267 | L | BP | cellular protein metabolic process |
| 0.8 | GO:0007154 | L | BP | cell communication |
| 0.795 | GO:0031981 | L | CC | nuclear lumen |
| 0.785 | GO:0006725 | L | BP | cellular aromatic compound metabolic process |
| 0.752 | GO:0005829 | L | CC | cytosol |
| 0.752 | GO:0006464 | L | BP | cellular protein modification process |
| 0.743 | GO:0046872 | L | MF | metal ion binding |
| 0.72 | GO:0032991 | L | CC | macromolecular complex |
| 0.714 | GO:0006996 | L | BP | organelle organization |
| 0.71 | GO:0005654 | L | CC | nucleoplasm |
| 0.673 | GO:0032502 | L | BP | developmental process |
| 0.665 | GO:0010467 | L | BP | gene expression |
| 0.639 | GO:0048856 | L | BP | anatomical structure development |
| 0.632 | GO:0036094 | L | MF | small molecule binding |
| 0.631 | GO:0009893 | L | BP | positive regulation of metabolic process |
| 0.63 | GO:0016070 | L | BP | RNA metabolic process |
| 0.629 | GO:0007275 | L | BP | multicellular organismal development |
| 0.598 | GO:0043234 | L | CC | protein complex |
| 0.588 | GO:0043169 | L | MF | cation binding |
| 0.549 | GO:0010008 | L | CC | endosome membrane |
| 0.54 | GO:0009966 | L | BP | regulation of signal transduction |
| 0.527 | GO:0031325 | L | BP | positive regulation of cellular metabolic process |
| 0.523 | GO:0000209 | L | BP | protein polyubiquitination |

**Blast**

| **BLAST Matched UniProt ID & name** | | | **Identity** | **E Value** | **Score** |
| --- | --- | --- | --- | --- | --- |
| P22279 | RAS2_MUCCL | Ras-like protein 2 | 33.846 | 6.67E-31 | 119 |
| P34729 | RAS1_PHYPO | Ras-like protein 1 | 38.378 | 2.65E-29 | 115 |
| P15064 | RASG_DICDI | Ras-like protein rasG | 37.297 | 2.27E-28 | 112 |
| Q55CB7 | RASY_DICDI | Ras-like protein rasY | 34.146 | 8.97E-28 | 111 |
| P22278 | RAS1_MUCCL | Ras-like protein 1 | 34.314 | 4.99E-27 | 109 |
| Q05058 | RASL_COPCI | 24 kDa Ras-like protein | 35.135 | 5.10E-27 | 109 |
| P08645 | RAS3_DROME | Ras-like protein 3 | 33.846 | 8.56E-27 | 108 |
| Q55CA9 | RASZ_DICDI | Ras-like protein rasZ | 34.634 | 1.07E-26 | 108 |
| P28775 | RAS_LENED | Ras-like protein | 35.135 | 1.29E-26 | 108 |
| P32253 | RASC_DICDI | Ras-like protein rasC | 33.838 | 1.34E-26 | 107 |
| Q18246 | RAP1_CAEEL | Ras-related protein Rap-1 | 33.838 | 1.43E-26 | 107 |
| P32254 | RASS_DICDI | Ras-like protein rasS | 33.333 | 1.53E-26 | 107 |
| P34726 | RAS2_PHYPO | Ras-like protein 2 | 35.676 | 1.60E-26 | 107 |
| Q59XU5 | RAS1_CANAL | Ras-like protein 1 | 36.216 | 1.83E-26 | 109 |
| C4YKT4 | RAS1_CANAW | Ras-like protein 1 | 36.216 | 2.04E-26 | 109 |
| P0CY32 | RAS1_CANAX | Ras-like protein 1 | 36.216 | 2.09E-26 | 109 |
| Q55CC0 | RASW_DICDI | Ras-like protein rasW | 33.663 | 2.88E-26 | 107 |
| P08647 | RAS_SCHPO | Ras-like protein 1 | 36.216 | 3.21E-26 | 107 |
| Q12526 | RAS_EMENI | Ras-like protein | 35.135 | 4.43E-26 | 107 |
| Q55CB0 | RASU_DICDI | Ras-like protein rasU | 33.168 | 6.74E-26 | 106 |
| O93856 | RAS_LACBI | Ras-like protein | 32.328 | 1.02E-25 | 105 |
| Q55CB8 | RASX_DICDI | Ras-like protein rasX | 34.466 | 1.71E-25 | 105 |
| O42785 | RASL_COLTR | Ras-like protein | 32.42 | 1.83E-25 | 105 |
| Q01387 | RAS2_NEUCR | Protein ras-2 | 31.527 | 1.94E-25 | 105 |
| P03967 | RASD_DICDI | Ras-like protein rasD | 35.079 | 3.12E-25 | 103 |
| P01120 | RAS2_YEAST | Ras-like protein 2 | 35.484 | 5.86E-25 | 105 |
| P87018 | RAS_BOTFU | Ras-like protein | 34.054 | 7.62E-25 | 103 |
| Q94694 | RAP1_PHYPO | Ras-related protein Rap-1 | 32.178 | 7.77E-25 | 102 |
| Q99578 | RIT2_HUMAN | GTP-binding protein Rit2 | 30.348 | 9.27E-25 | 103 |
| P22280 | RAS3_MUCCL | Ras-like protein 3 | 34.054 | 9.50E-25 | 103 |
| P70425 | RIT2_MOUSE | GTP-binding protein Rit2 | 29.808 | 1.61E-24 | 102 |
| Q5BJQ5 | RIT2_RAT | GTP-binding protein Rit2 | 29.808 | 3.60E-24 | 101 |
| P70426 | RIT1_MOUSE | GTP-binding protein Rit1 | 30.332 | 4.55E-24 | 101 |
| P18613 | RAPA_DICDI | Ras-related protein rapA | 32.178 | 8.90E-24 | 99.8 |
| Q7ZXH7 | RAP1B_XENLA | Ras-related protein Rap-1b | 32.653 | 9.62E-24 | 99.8 |
| Q6TEN1 | RAP1B_DANRE | Ras-related protein Rap-1b | 32.653 | 1.05E-23 | 99.8 |
| Q92963 | RIT1_HUMAN | GTP-binding protein Rit1 | 30.097 | 1.34E-23 | 100 |
| Q9YH37 | RAP1B_CYPCA | Ras-related protein Rap-1b | 32.653 | 1.71E-23 | 99 |
| Q95KD9 | DIRA2_MACFA | GTP-binding protein Di-Ras2 | 32.524 | 3.07E-23 | 98.6 |
| P18262 | RAS_ARTSA | Ras-like protein | 32.804 | 3.11E-23 | 98.2 |
| P23175 | RASH_MSVNS | GTPase HRas | 30.653 | 5.08E-23 | 97.8 |
| P01117 | RASK_MSVKI | GTPase KRas | 30.653 | 5.89E-23 | 97.8 |
| Q99JI6 | RAP1B_MOUSE | Ras-related protein Rap-1b | 32.143 | 5.94E-23 | 97.4 |
| Q62636 | RAP1B_RAT | Ras-related protein Rap-1b | 32.143 | 7.90E-23 | 97.1 |
| Q96HU8 | DIRA2_HUMAN | GTP-binding protein Di-Ras2 | 32.039 | 8.06E-23 | 97.4 |
| Q5R6S2 | DIRA2_PONAB | GTP-binding protein Di-Ras2 | 32.039 | 9.90E-23 | 97.4 |
| P0CQ43 | RAS_CRYNB | Ras-like protein | 33.514 | 1.15E-22 | 97.4 |
| P01113 | RASH_MSVMO | GTPase HRas | 30.151 | 1.26E-22 | 96.7 |
| P01119 | RAS1_YEAST | Ras-like protein 1 | 33.333 | 1.54E-22 | 98.6 |
| P22123 | RAPA_DIPOM | Ras-related protein O-Krev | 32.143 | 1.62E-22 | 96.3 |
| P01116 | RASK_HUMAN | GTPase KRas | 32.275 | 2.05E-22 | 96.3 |
| P01115 | RASH_MSVHA | Transforming protein p29 [Cleaved into: Transforming protein p21] | 30.151 | 2.12E-22 | 97.1 |
| Q5PR73 | DIRA2_MOUSE | GTP-binding protein Di-Ras2 | 31.553 | 2.21E-22 | 96.3 |
| P62836 | RAP1A_RAT | Ras-related protein Rap-1A | 32.143 | 2.84E-22 | 95.5 |
| P05774 | RAS_CARAU | Ras-like protein | 32.275 | 3.29E-22 | 95.5 |
| Q91806 | RASN_XENLA | GTPase NRas | 32.039 | 5.10E-22 | 95.1 |
| Q550Q4 | RHEB_DICDI | GTP-binding protein Rheb homolog | 29.744 | 7.19E-22 | 94.4 |
| P01114 | RASH_RRASV | Transforming protein p29 [Cleaved into: Transforming protein p21] | 30.151 | 7.29E-22 | 95.9 |
| P79800 | RASK_MELGA | GTPase KRas | 31.053 | 8.08E-22 | 94.4 |
| P32883 | RASK_MOUSE | GTPase KRas | 31.746 | 9.08E-22 | 94.4 |
| A6NIZ1 | RP1BL_HUMAN | Ras-related protein Rap-1b-like protein | 31.633 | 1.00E-21 | 94 |
| P13856 | RSR1_YEAST | Ras-related protein RSR1 | 30.303 | 1.42E-21 | 95.1 |
| Q9YH38 | RASK_CYPCA | GTPase KRas | 31.892 | 1.85E-21 | 93.6 |
| P32252 | RASB_DICDI | Ras-like protein rasB | 30.811 | 5.58E-21 | 92.4 |
| O42277 | RASK_ORYLA | GTPase KRas | 31.351 | 6.79E-21 | 91.7 |
| P01112 | RASH_HUMAN | GTPase HRas | 30.688 | 8.31E-21 | 91.7 |
| Q07983 | RASK_MONDO | GTPase KRas | 31.053 | 1.15E-20 | 91.3 |
| P08642 | RASH_CHICK | GTPase HRas | 30.688 | 1.50E-20 | 90.9 |
| Q05147 | RASK_XENLA | GTPase KRas | 30.526 | 2.01E-20 | 90.5 |
| Q04970 | RASN_RAT | GTPase NRas | 30.097 | 7.00E-20 | 89 |
| P01111 | RASN_HUMAN | GTPase NRas | 30.688 | 8.40E-20 | 88.6 |
| Q55CB9 | RASV_DICDI | Ras-like protein rasV | 30.622 | 8.53E-20 | 89.4 |
| P12825 | RASN_CAVPO | GTPase NRas | 30.688 | 8.72E-20 | 88.6 |
| P08646 | RAS1_DROME | Ras-like protein 1 | 29.954 | 9.42E-20 | 88.6 |
| P08556 | RASN_MOUSE | GTPase NRas | 30.688 | 9.88E-20 | 88.6 |
| Q5F352 | RASN_CHICK | GTPase NRas | 30.688 | 1.15E-19 | 88.2 |
| Q95ME4 | RASN_MONDO | GTPase NRas | 30.688 | 1.16E-19 | 88.2 |
| P10301 | RRAS_HUMAN | Ras-related protein R-Ras | 30.645 | 1.26E-19 | 88.6 |
| B4NJ72 | RAS1_DROWI | Ras-like protein 1 | 29.493 | 1.66E-19 | 87.8 |
| P04388 | RAS2_DROME | Ras-like protein 2 | 29.016 | 2.09E-19 | 87.8 |
| Q5EFX7 | RASK_KRYMA | GTPase KRas | 30.811 | 3.04E-19 | 87 |
| Q295X7 | RAS1_DROPS | Ras-like protein 1 | 29.493 | 3.06E-19 | 87 |
| P79737 | RASN_DANRE | GTPase NRas | 30.688 | 4.05E-19 | 86.7 |
| B4JFU8 | RAS1_DROGR | Ras-like protein 1 | 30.729 | 4.24E-19 | 86.7 |
| B4LY29 | RAS1_DROVI | Ras-like protein 1 | 31.351 | 4.66E-19 | 86.7 |
| Q5RD87 | RASN_PONAB | GTPase NRas | 30.159 | 6.15E-19 | 86.3 |
| P22126 | RAS1_NEUCR | Protein ras-1 | 30.256 | 8.36E-19 | 86.3 |
| P62070 | RRAS2_HUMAN | Ras-related protein R-Ras2 | 29.73 | 9.23E-19 | 85.9 |
| P22981 | LET60_CAEEL | Ras protein let-60 | 31.053 | 1.14E-18 | 85.5 |
| P62820 | RAB1A_HUMAN | Ras-related protein Rab-1A | 27.136 | 1.79E-18 | 85.1 |
| O94363 | RHB1_SCHPO | GTP-binding protein rhb1 | 28.205 | 1.90E-18 | 84.7 |
| O76173 | RAB1C_DICDI | Ras-related protein Rab-1C | 30.303 | 2.14E-18 | 84.7 |
| P38976 | RAS2_HYDVU | Ras-like protein RAS2 | 29.189 | 2.25E-18 | 84.7 |
| P51539 | RAS1_HYDVU | Ras-like protein RAS1 | 31.053 | 2.56E-18 | 84.3 |
| P10833 | RRAS_MOUSE | Ras-related protein R-Ras | 30.108 | 3.14E-18 | 84.7 |
| P33723 | YPT1_NEUCR | GTP-binding protein ypt1 | 29.146 | 3.64E-18 | 84.3 |
| P22125 | RAB1_DIPOM | Ras-related protein ORAB-1 | 27.136 | 3.73E-18 | 84.3 |
| Q39433 | RB1BV_BETVU | Ras-related protein RAB1BV | 30.093 | 3.94E-18 | 84.3 |
| B4KB60 | RAS1_DROMO | Ras-like protein 1 | 29.524 | 4.76E-18 | 83.6 |
| Q08E00 | RASLC_BOVIN | Ras-like protein family member 12 | 29.218 | 4.94E-18 | 84.7 |
