# Supplementary material for: SVM-Prot 2016: A Web-Server for Machine Learning Prediction of Protein Functional Families from Sequence Irrespective of Similarity
Source: PLoS One. 2016 Aug 15;11(8):e0155290. doi: 10.1371/journal.pone.0155290 (PMC4985167; doi:10.1371/journal.pone.0155290)
Supplement: S5 Table — (DOCX) [file pone.0155290.s005.docx]

**Table S5.** 10 representative protein functional families covered by SVM-Prot and the prediction performance of the LibD3C, SVM, kNN and PNN models on the independent testing sets. The prediction results are given in Sensitivity SE=TP/(TP+FN), Specificity SP=TN/(TN+FP), Precision PR=TP/(TP + FP), where TP=true positive, FN=false negative, TN=true negative, and FP=false positive respectively.

| **Family Name** | **GO Id** | **SVM** | | | **LibD3C** | | | **KNN** | | | **PNN** | | |
| --- | --- | --- | --- | --- | --- | --- | --- | --- | --- | --- | --- | --- | --- |
|  |  | **SE (%)** | **SP (%)** | **PR (%)** | **SE (%)** | **SP (%)** | **PR (%)** | **SE (%)** | **SP (%)** | **PR (%)** | **SE (%)** | **SP (%)** | **PR (%)** |
| Actin capping | GO:0051693 | 95.1 | 99.99 | 93.3 | 97.06 | 99.89 | 71.74 | 73.3 | 99.9 | 55.0 | 91.2 | 99.9 | 71.0 |
| DNA recombination | GO:0006310 | 85.7 | 97.4 | 92.1 | 87.11 | 98.75 | 76.52 | 67.5 | 99.3 | 80.3 | 77.6 | 98.9 | 77.0 |
| DNA repair | GO:0006281 | 88.7 | 96.8 | 85.9 | 83.56 | 97.1 | 74.66 | 67.6 | 96.8 | 68.0 | 64.3 | 99.3 | 90.4 |
| EC1.5 Oxidoreductases (CH-NH donors) | GO:0016645 | 58.6 | 99.6 | 66.1 | 89.65 | 97.53 | 85.36 | 84.5 | 95.8 | 76.0 | 64.2 | 99.2 | 92.6 |
| EC2.9 Transferases (selenium-containing) | GO:0016785 | 96.0 | 99.99 | 99.3 | 99.03 | 99.63 | 81.9 | 83.7 | 99.7 | 81.4 | 92.4 | 99.9 | 92.5 |
| EC4.4 Carbon-sulfur lyases | GO:0016846 | 60.3 | 99.9 | 83.3 | 92.06 | 98.87 | 83.93 | 77.0 | 99.0 | 82.7 | 83.8 | 99.2 | 86.8 |
| EC5.1 Racemases and Epimerases | GO:0016854 | 53.0 | 99.4 | 53.9 | 93.2 | 96.86 | 90.51 | 80.7 | 93.8 | 80.0 | 69.3 | 98.7 | 94.5 |
| Elongation factor activity | GO:0003746 | 97.5 | 99.99 | 98.8 | 95.73 | 99.15 | 73.72 | 95.8 | 99.6 | 83.7 | 84.1 | 99.9 | 94.0 |
| G protein coupled receptors | GO:0004930 | 95.6 | 98.1 | 94.5 | 98.56 | 98.65 | 52.39 | 96.6 | 98.9 | 64.1 | 94.1 | 99.9 | 93.4 |
| Lipid-binding | GO:0008289 | 84.4 | 99.9 | 93.4 | 79.23 | 99.59 | 67.32 | 72.8 | 99.6 | 71.2 | 66.9 | 99.7 | 72.1 |
